# Electrochemical Fluorination of Partially Fluorinated Dialkylethers. Synthesis of Sevoflurane Derivatives (CF_3_)_2_CHOCHF_2_, (CF_3_)_2_CHOCF_3_, and (CF_3_)_2_CFOCF_3_

**DOI:** 10.1002/chem.71589

**Published:** 2026-08-19

**Authors:** Tanja Knuplez, Leon N. Schneider, Erik R. Csapo, Peer Kirsch, Nikolai V. Ignat'ev, Maik Finze

**Affiliations:** ^1^ Julius Maximilian University Würzburg, Institute of Inorganic Chemistry, Institute for Sustainable Chemistry & Catalysis with Boron (ICB) Würzburg Germany; ^2^ Merck Electronics KGaA Darmstadt Germany

**Keywords:** electrochemical fluorination, electrosynthesis, ether, structure elucidation

## Abstract

Electrochemical fluorination in anhydrous hydrogen fluoride (ECF in aHF) according to the Simons process was used for the fluorination of the anesthetic sevoflurane (CF_3_)_2_CHOCH_2_F to yield (CF_3_)_2_CHOCHF_2_, (CF_3_)_2_CHOCF_3_, and (CF_3_)_2_CFOCF_3_. The related ethers R^F^OCH_3_ (R^F^ = *n*C_3_F_7_, a mixture of *n*C_4_F_9_/*i*C_4_F_9_, *i*C_4_F_9_, *t*C_4_F_9_) were fluorinated to result in the respective mono‐, di‐, and trifluoromethyl ethers. The vapor pressure curves of selected fluorinated ethers, including (CF_3_)_2_CHOCHF_2_, (CF_3_)_2_CHOCF_3_, and (CF_3_)_2_CFOCF_3_, were determined, and the Antoine fit parameters were used for the extrapolation of the normal boiling points and the enthalpies of vaporization. In addition, the crystal structures of sevoflurane, (CF_3_)_2_CHOCH_3_, (CF_3_)_2_CHOCHF_2_, (CF_3_)_2_CHOCF_3_, and (CF_3_)_2_CFOCF_3_ were determined by x‐ray diffraction, providing a detailed insight into trends of the bond parameters and the intermolecular interactions in the solid state. The interpretation of the bond parameters is supported by calculated values (density functional theory, DFT).

## Graphical abstract

Electrochemical fluorination (ECF, Simons process) is a valuable method for the fluorination of partially fluorinated dialkyl ethers, providing an inexpensive, scalable, and safe access to partially fluorinated ethers with a higher fluorine content and the perfluorinated analogues that are hardly accessible using other fluorination techniques. Physicochemical and structural data provide detailed insight into the selected properties of the selected ethers.

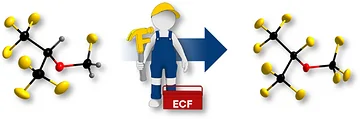

## 1 Introduction

The electrochemical fluorination (ECF) of organic substances in anhydrous hydrogen fluoride (aHF) was invented by the American scientist Joseph H. Simons in the early forties of the last century. In 1948, Minnesota Mining & Manufacturing Company (3M Company) patented Simons’ invention [1] and implemented the new technology for the synthesis of fluorochemicals. Nowadays, many fluorochemicals are manufactured via the Simons process or are follow‐up products of fluorinated compounds prepared via ECF. Takashi Abe, who greatly contributed to the development of the Simons process, described ECF as “a locomotive for the development of fluorine chemistry” [2]. ECF is the key method for the preparation of many high‐performance materials indispensable for modern applications of high relevance, for example, in medicine, the semiconductor industry, and energy production, conversion, and storage. Throughout its 80 year history, the Simons process was applied for the fluorination of compounds containing C─O, C─N, C─S, C─P, and S─N bonds [3, 4, 5, 6, 7, 8]. During the last 30 years, the focus shifted toward the synthesis of molecules with short‐chain fluoroalkyl substituents, which are, for example, used as building blocks in organic chemistry [4].

ECF in anhydrous HF provides many advantages compared to other fluorination methods using fluorinating reagents such as alkali metal fluorides, SbF_5_, XeF_2_, SF_4_, elemental fluorine, and so forth. One important advantage is that it requires only cheap reagents, that is, aHF and electricity. In addition, the cell design is relatively simple, particularly because an undivided electrochemical cell is used, which allows high current densities and thus ensures a high productivity per cell volume. Furthermore, the Simons process proceeds at mild conditions, for example, at a temperature range of −5 to 15°C and typically at atmospheric pressure. The product isolation from the cell is straightforward since the highly fluorinated products exhibit low solubilities in aHF and thus, they either escape from the cell as gas (low‐boiling fluorinated products) or separate inside the cell as bottom phase (high‐boiling fluorinated products). For the cathode, iron or nickel can be used, with nickel exhibiting a higher corrosion resistance. On the anode side, a metal fluoride layer that is insoluble in aHF is required. So, only nickel can be used, as a sufficiently adhesive layer of electrochemically generated high‐valent Ni fluorides (NiF_3_/NiF_4_) is formed [4, 9, 10, 11, 12]. The starting materials used in ECF need to be stable and soluble in aHF at a concentration of ca. 4–10 wt%. Hydrogen is the only byproduct, which is formed at the cathode (Scheme 1). Nevertheless, since an undivided electrochemical cell is used, the electrochemical reduction of fluorinated compounds at the cathode has to be considered [13].

**SCHEME 1:**
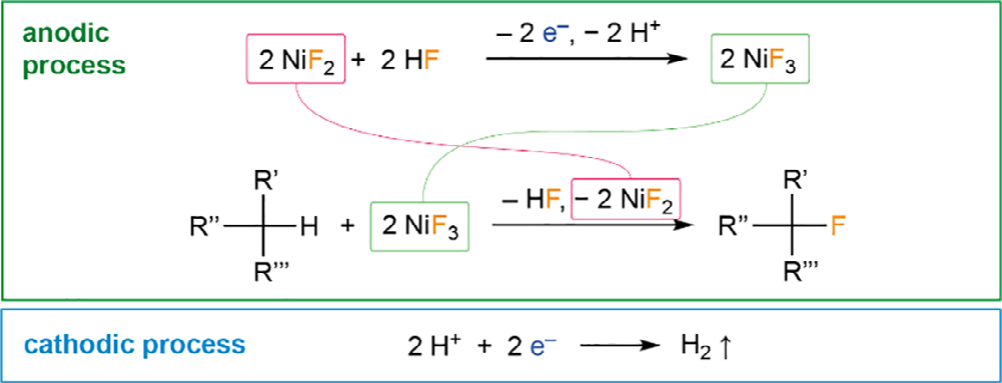
Electrochemical fluorination (Simons process) of organic compounds.

Since its discovery, the Simons process has been used for the electrochemical fluorination of, for example, sulfonic acid derivatives (mostly sulfonyl chlorides and sulfonyl fluorides), acetic acid derivatives (acyl chlorides and acyl fluorides, anhydrides, esters, etc), trialkyl amines, and phosphines on an industrial scale [4, 5, 6, 14, 15, 16, 17, 18, 19, 20, 21]. Many other substrates such as alcohols, heterocycles, sulfides, and disulfides were tested, as well. However, only a few examples of the ECF of dialkyl ethers were described in the literature. In 1950, J. H. Simons reported the electrochemical fluorination of di‐*n*‐butyl ether to give C_4_F_9_OC_4_F_9_ [1], but large amounts of C_4_F_10_ and other fluorocarbons of lower molecular weight were formed as side products. The ECF of a few other dialkyl ethers, that are, dimethyl, diethyl, diamyl, and dihexyl ethers, yielding the corresponding perfluorinated dialkyl ethers, was mentioned in a table in the same patent [1]. In another patent entitled “Fluorocarbon Ethers,” J. H. Simons described the electrochemical preparation of CF_3_OCF_3_, C_2_F_5_OC_2_F_5_, (*n*C_3_F_7_)_2_O, (*n*C_4_F_9_)_2_O, (*n*C_5_F_11_)_2_O, and (*n*C_6_F_13_)_2_O from the corresponding dialkyl ethers [22]. The formation of various side products with shorter fluorinated alkyl chains compared to the starting compounds was observed, which was rationalized by C─O bond cleavage in the course of ECF. In general, ECF of dialkyl ethers gave the corresponding perfluorodialkyl ethers in low yields, only [14]. In particular, for long carbon chain dialkyl ethers, degradation processes were observed. For example, ECF of (*n*C_5_H_11_)_2_O and (*n*C_6_H_13_)_2_O provided the respective perfluorinated substances in low yields of 14 and 10%, respectively [23]. Nagase et al. employed dialkyl ethers as starting material for the synthesis of perfluorocarboxylic acids, and ECF of diethyl, di‐*n*‐propyl, ethyl‐*n*‐butyl, and di‐*n*‐butyl ether gave the respective perfluorocarboxylic acid (isolated as Na^+^ salt) in 22%–36% yield [24]. Perfluorinated dialkyl ethers are formed as side products. Similar results were reported by Berenblit et al. for the ECF of dimethoxyethane (monoglyme) that gave CF_3_OCF_2_CF_2_OCF_3_ in 14%, CF_3_OCF_2_C(O)F in 18%, and CF_3_C(O)F in 2.5% yield [25].

Electrochemical fluorination of chlorine‐containing ethers was studied by Okazaki et al. [26]. ECF of CH_2_ClOCH_3_, CH_2_ClOCH_2_Cl, CHCl_2_OCH_2_Cl, CCl_3_OCH_3_, and CHCl_2_OCHCl_2_ resulted in per‐ and partially fluorinated ethers in low yield, for example, CF_3_OCF_3_, CF_3_OCHF_2_, and CHF_2_OCHF_2_ [26]. Only two chlorine‐containing fluoroethers, namely CHFClOCHF_2_ from dichloromethyl–chloromethyl ether and CF_2_ClOCH_3_ from trichloromethyl methyl ether, were isolated after ECF in 0.6% and 13.2% yield. Presumably, the high content of partially fluorinated dimethyl ethers in the product mixtures obtained from ECF of chlorine‐containing dimethyl ethers is a consequence of the low boiling point and low solubility of partially fluorinated dimethyl ethers formed by replacement of chlorine with fluorine, which facilitates their release into the gas phase. In contrast, chlorine atoms in β‐position to oxygen in chloroethyl methyl ethers are resistant to replacement by fluorine with aHF. Hence, a number of methyl ethyl ethers containing fluorine and chlorine were obtained by ECF of CH_3_OCH_2_CCl_3_, CH_3_OCHClCCl_3_, and CH_3_OCCl_2_CCl_3_. In addition, side reactions led to the formation of acid fluorides that, after work‐up with caustic soda, resulted in Na{CF_3_C(O)O}, Na{CClF_2_C(O)O}, and Na{CCl_2_FC(O)O} [26].

In previous publications, we demonstrated that the presence of fluorine or trifluoromethyl substituents at heteroatoms preserves the decomposition of the starting material during ECF in aHF, leading to higher yields of perfluorinated and partially fluorinated molecules, which are valuable synthons for the introduction of fluorinated groups into organic molecules [4, 13, 27, 28, 29, 30, 31]. So far, only two articles and one patent have been published that describe the ECF of partially fluorinated dialkyl ethers. In 1990, Chambers et al. described the ECF of {CF_3_CHFCF_2_CH(CH_3_)}_2_O [32]. The corresponding perfluoro‐di(1‐methylbutyl)ether {CF_3_CF_2_CF_2_CF(CF_3_)}_2_O was obtained in 8% yield only (Scheme 2A). This low yield was explained by the parallel formation of a complex mixture of partially fluorinated side products. ECF of *n*C_6_H_13_C(CF_3_)_2_OCH_3_ studied by Ono et al. yielded the perfluorinated ether *n*C_6_F_13_C(CF_3_)_2_OCF_3_ in 27% [33], and cyclic perfluorinated ethers (oxolane and oxane) were observed as side products in 7.7% and 1% yield (Scheme 2B). The patent application entitled “Preparation of F‐alkyl F‐isobutyl ethers by electrochemical fluorination” described the ECF of a compound mixture obtained from the addition of alcohols to perfluoro‐*iso*butylene [34]. For example, ECF of a mixture of (CF_3_)_2_CHCF_2_OCH_3_ (74 wt.%), (CF_3_)_2_C═CFOCH_3_ (15 wt.%), and CF_2_═C(CF_3_)CF_2_OCH_3_ (8 wt.%) gave the fluorinated ethers (CF_3_)_2_CFCF_2_OCF_3_ and (CF_3_)_2_CHCF_2_OCF_3_ in 50% and 5% yield, respectively (Scheme 2C). The presence of the side products COF_2_, CF_4_, C_3_F_8_, *iso*‐C_4_F_10_, and (CF_3_)_2_CFC(O)F in the reaction mixture showed the degradation during ECF [34].

**SCHEME 2:**
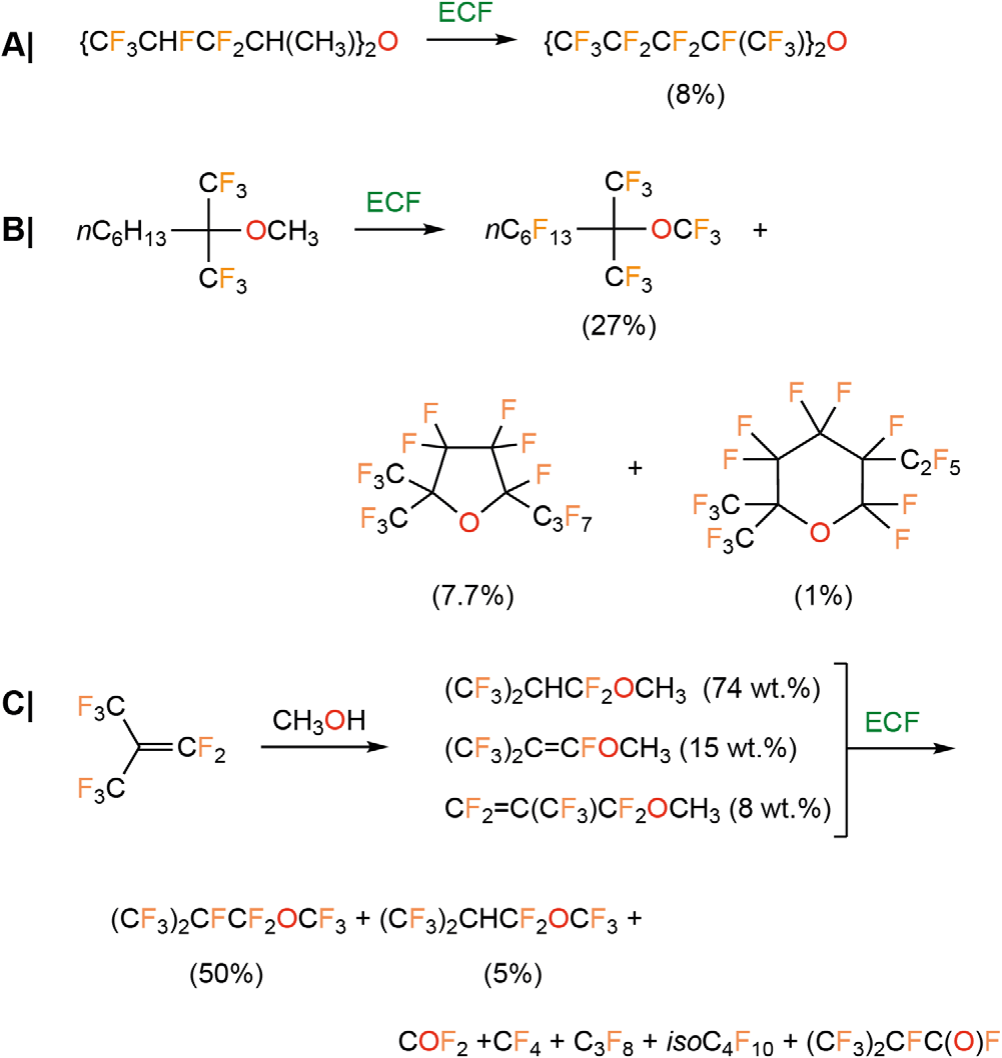
ECF of partially fluorinated dialkyl ethers.

Fluorinated dialkyl ethers are important compounds relevant for a number of different applications [14, 35, 36], such as refrigerants [37], blowing agents [37], cleaning solvents [37], materials for the production and operation of electronic devices such as OLEDs [38], and anesthetics and other medicinal applications [39]. Fluranes such as sevoflurane are examples of fluorinated dialkyl ethers that are widely used. Sevoflurane (CF_3_)_2_CHOCH_2_F (**1**), which contains a fluoromethoxy group bonded to a hexafluoro‐*iso*‐propyl residue, is a well‐known volatile anesthetic that is used in surgical operations [40, 41, 42, 43]. In addition, it was recently employed as a solvent in lithium‐ion batteries (LIB) [44]. The related ether (CF_3_)_2_CHOCH_3_ (HFPM, isoflurothyl, HFE‐356mmz), which is also an anesthetic [45], was, for example, used for the design of nonflammable battery electrolytes [46, 47, 48] and as a refrigerant [49]. Furthermore, it serves as a starting compound for the synthesis of sevoflurane [42]. Its heptafluoroisopropyl analogue (CF_3_)_2_CFOCH_3_ [50, 51] is synthetically easily accessible [51] and an industrial product. The sevoflurane derivatives (CF_3_)_2_CHOCHF_2_ (**2**), (CF_3_)_2_CHOCF_3_ (**3**), and (CF_3_)_2_CFOCF_3_ (**4**) have been less investigated and applied than sevoflurane or (CF_3_)_2_CHOCH_3_ and (CF_3_)_2_CFOCH_3_, presumably because their synthesis is more complex, provides them in relatively low yield, and requires laborious isolation procedures. The synthesis of ether **2** was described in a patent application as early as 1969 [52]. A number of syntheses of **2** were described, which mostly rely on dechlorofluorinations [53], for example, of (CF_3_)_2_CHOCHCl_2_ [52]. Similarly, **3** is accessible via chlorination and subsequent chlorine–fluorine exchange [54, 55]. For both, (CF_3_)_2_CHOCH_3_ is the key precursor for the synthesis. In contrast, perfluoro‐2‐methoxy propane **4** was reported as the minor product of the addition of CF_3_OF to hexafluoropropene CF_3_CF═CF_2_ and it was only obtained in a mixture with the main product CF_3_CF_2_CF_2_OCF_3_ [56, 57].

In this contribution, we report the electrochemical fluorination (ECF, Simons process) of sevoflurane (**1**) to yield the fluorinated 2‐methoxy propanes **2**–**4** that were characterized in detail, including single crystal x‐ray diffraction (SCXRD). ECF of a series of perfluoroalkyl methyl ethers complements the fluorination study of **1**.

## 2 Results and Discussion

### 2.1 Electrochemical Fluorination of Sevoflurane

The electrochemical fluorination of sevoflurane (CF_3_)_2_CHOCH_2_F (**1**) resulted in a mixture of (CF_3_)_2_CHOCHF_2_ (**2**), (CF_3_)_2_CHOCF_3_ (**3**), and (CF_3_)_2_CFOCF_3_ (**4**) (Scheme 3), which were separated by fractional distillation and characterized by multinuclear NMR spectroscopy, vibrational (Raman and IR) spectroscopy, mass spectrometry, and SCXRD (vide infra).

**SCHEME 3:**
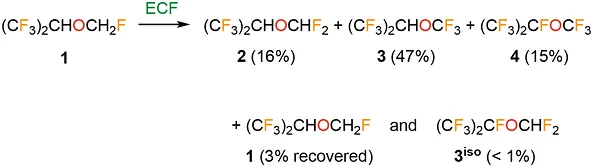
Electrochemical fluorination of sevoflurane (**1**) to yield **2**–**4** and trace amounts of (CF_3_)_2_CFOCHF_2_ (**3^iso^**) (yields determined by ^19^F NMR spectroscopy of the crude product mixture).

The major product of the ECF of sevoflurane was (CF_3_)_2_CHOCF_3_ (**3**) (Scheme 3). Presumably, the hydrogen atom in the *iso*‐position is to some extent protected by the two neighboring CF_3_ groups against fluorination at the high‐valent nickel fluoride layer on the Ni anode. Adsorption of the starting material on the surface of the Ni anode in the first step of the ECF process proceeded by orientation of **1** via the fluoromethyl group to the positively charged Ni anode, which explains the formation of (CF_3_)_2_CHOCHF_2_ (**2**) (Scheme 4). ECF is known to be a stepwise process [4], and so **2** was converted into (CF_3_)_2_CHOCF_3_ (**3**) in the second fluorination step. Each reaction step required two electrons and one molecule of HF (Scheme 1). The further fluorination at the *iso*‐position of the ─CH(CF_3_)_2_ moiety of **3** to result in the perfluorinated compound (CF_3_)_2_CFOCF_3_ (**4**) was slow, presumably, due to steric reasons as stated above. The stepwise fluorination followed the trend of decreasing energies of the HOMO orbitals (Figure 1).

**SCHEME 4:**
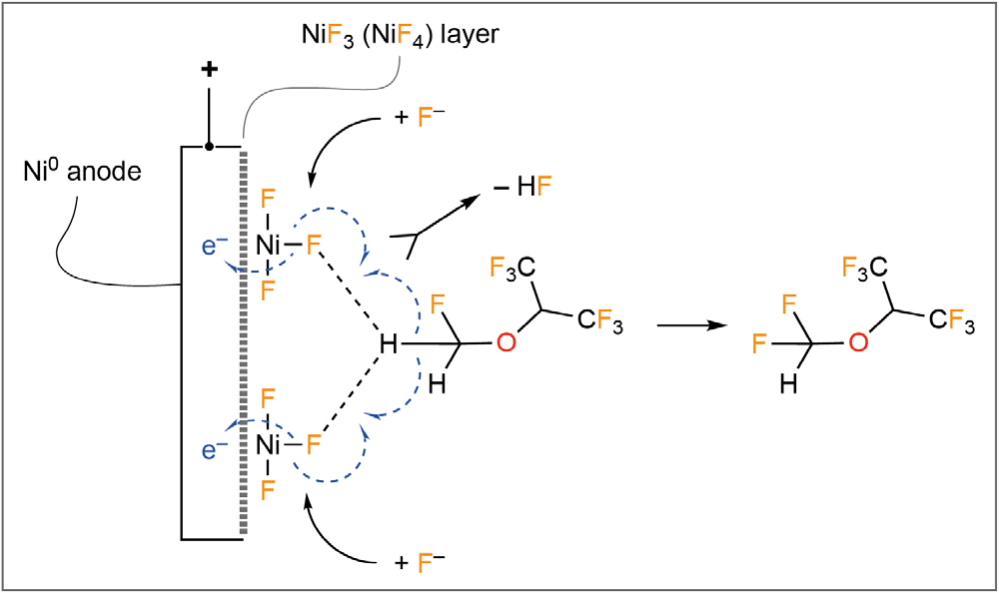
Electrochemical fluorination (ECF, Simons process) of sevoflurane (**1**) adsorbed on the surface of the nickel anode covered with NiF_3_/NiF_4_ to give (CF_3_)_2_CHOCHF_2_ (**2**).

**FIGURE 1:**
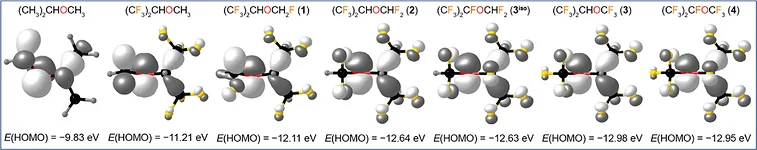
Plot of the HOMOs and HOMO energies of (CH_3_)_2_CHOCH_3_, (CF_3_)_2_CHOCH_3_, **1**–**4**, and **3^iso^** (ωB97X‐D4/def2‐TZVPP).

Electrochemical fluorination of sevoflurane (**1**) was carried out in an undivided stainless‐steel cell (Simons cell, *V* = 1.5 L) equipped with an array of nickel cathodes and anodes (effective anode area *S* = 15.2 dm^2^). The cell was filled with 1250 mL of anhydrous HF, which was pre‐electrolyzed at a constant voltage of 5.0 V overnight to remove residual water in HF and to form an active nickel fluoride layer on the surface of the Ni anode. At the start of the reaction, the cell was charged with 61.5 g of **1**, and during ECF portions of **1** were added using a syringe pump every hour. The portions of **1** added were adjusted according to the consumed electricity (Table S1). Altogether, 1646.1 g of **1** was added. The cell was operated at 5 to 8°C, a voltage range of 4.5–6.0 V, and an anodic current density of 0.14–0.26 A·dm^−2^. The electrolysis was stopped after the consumption of 1228 Ah of electricity. The gaseous products were collected during ECF in three subsequent PTFE traps maintained at −78°C, and in total, 1732.3 g of crude material was collected. Three main products **2**, **3**, and **4** were isolated from the material collected in the traps along with a small amount of the starting material **1** (Scheme 3). A few further minor components (0.9 g in the crude material, see Table S2), that is, hexafluoropropane CF_3_CHFCF_3_, heptafluoropropane CF_3_CF_2_CF_3_, trifluoromethyl hypofluorite CF_3_OF, carbonyl difluoride COF_2_, and the sevoflurane derivative (CF_3_)_2_CFOCHF_2_ (**3^iso^**), were identified by ^19^F NMR spectroscopy.

Distillation of collected product mixtures obtained during optimization of the ECF of **1** gave a fraction with a higher content of **3^iso^** that enabled the assignment of the ^1^H and ^19^F NMR signals (Figures S48 and S52). Most likely, the formation of only trace amounts of **3^iso^** was due to the steric shielding of the *iso*‐position of the ─CH(CF_3_)_2_ group of (CF_3_)_2_CHOCHF_2_ (**2**) (vide supra).

### 2.2 Electrochemical Fluorination of Selected Partially Fluorinated Dialkyl Ethers

To demonstrate the scope of the ECF of partially fluorinated dialkyl ethers, we investigated the electrochemical fluorination of R^F^OCH_3_ (R^F^ = *n*C_3_F_7_ (Novec7000), a mixture of *n*C_4_F_9_/*i*C_4_F_9_ (Novec7100), *i*C_4_F_9_, *t*C_4_F_9_) (Scheme 5 and Table 1). Although the starting compounds R^F^─OCH_3_ had only hydrogen atoms bonded to a primary carbon atom instead of two hydrogens and one fluorine as in **1**, in all cases the respective derivatives with a difluoromethoxy and a trifluoromethoxy group were obtained in good yield. In contrast to electrochemical fluorination of sevoflurane (Scheme 3), ECF of R^F^OCH_3_ gave the perfluorinated substance R^F^OCF_3_ as the main product (Table 1). All trifluoromethyl and difluoromethyl derivatives, and in addition *i*C_4_F_9_OCH_2_F, were isolated and spectroscopically characterized.

**SCHEME 5:**
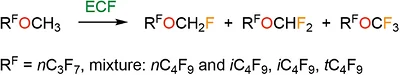
ECF of selected perfluoroalkyl methyl ethers.

**TABLE 1:** ECF of R^F^OCH_3_ (R^F^ = *n*C_3_F_7_, *n*/*i*C_4_F_9_, *i*C_4_F_9_, *t*C_4_F_9_) in aHF.

R^F^	*m*_reactant_ ^a^		*V*_cell_ ^b^	*Q*·*n* ^−1^	*z* ^c^	*U*_cell_	*I*_a_·S^−1^	*T*_cell_	*m*_product_ ^d^	R^F^OCH_3_ ^e^	R^F^OCH_2_F	R^F^OCHF_2_	R^F^OCF_3_
	(g)	(mol)	(mL)	(Ah·mol^−1^)		(V)	(A·dm^−2^)	(°C)	(g)	(%)^f^	(%)^f^	(%)^f^	(%)^f^
*n*C_3_F_7_ ^g^	314	1.57	430	161.7	6.0	4.6–6.0	0.1–0.4	2–4	325	6.8	1.1	26.6	50.3
*n/i*C_4_F_9_ ^h^	333	1.33	340	166.1	6.2	5.7–6.5	0.1–0.5	3	337	1.9	0.1	16.7	65.8
*i*C_4_F_9_	991	3.96	340	147.7	5.5	4.4–6.2	0.1–0.5	−5–7	904	7.1	3.5	20.5	46.8
*t*C_4_F_9_	178	0.69	290	174.3	6.5	5.3–6.0	0.3–0.4	0	163	15.1	2.4	17.5	44.5

^a^ The perfluoroalkylmethylether was added batchwise to the reaction mixture.

^b^ Volume of the ECF cell.

^c^ Electron equivalents per mole: *z* = *Q*·*n* ^−1^·F^−1^.

^d^ Combined product collected in the cold traps at the outlet of the cell and isolated as organic phase from the reaction mixture in the cell upon addition of water and ice.

^e^ Starting material recovered.

^f^ Yields determined by ^19^F NMR of the crude product before distillation.

^g^ Novec7000.

^h^ Mixture of *n*C_4_F_9_OCH_3_ and *i*C_4_F_9_OCH_3_ (Novec7100).

### 2.3 Vapor Pressure Curves and Boiling Points

The vapor pressure curves of most of the fluorinated dialkyl ethers studied were measured, and the Antoine fitting parameters are provided in Table 2 together with the enthalpies of vaporization (Δ_vap_ *H*) and the boiling points (*T*_bp_) derived from the fits of the vapor pressure. The Antoine fits of the data of (CF_3_)_2_CHOCH_3_, **1**–**4**, *n*C_3_F_7_OCH_3_, *n*C_3_F_7_OCHF_2_, and *n*C_3_F_7_OCF_3_ are depicted in Figure 2.

**TABLE 2:** Normal boiling point (*T*_bp_), enthalpy of vaporization (Δ_vap_H) at *T* = 298.15 K and Antoine parameters^a^ for *n*C_3_F_7_OCH_3_, *n*C_3_F_7_OCHF_2_, *n*C_3_F_7_OCF_3_, (CF_3_)_2_CHOCH_3_, (CF_3_)_2_CHOCH_2_F (**1**), (CF_3_)_2_CHOCHF_2_ (**2**), (CF_3_)_2_CHOCF_3_ (**3**), (CF_3_)_2_CFOCF_3_ (**4**), *i*C_4_F_9_OCH_3_, *i*C_4_F_9_OCH_2_F, *i*C_4_F_9_OCHF_2_, *i*C_4_F_9_OCF_3_, *t*C_4_F_9_OCH_3_, *t*C_4_F_9_OCHF_2_ and *t*C_4_F_9_OCF_3_, as well as for the mixtures *i_0.8_*/*n_0.2_* C_4_F_9_OCH_3_, *i_0.8_*/*n_0.2_* C_4_F_9_OCHF_2_ and *i_0.8_*/*n_0.2_* C_4_F_9_OCF_3_.

	*T*_bp_ (°C)		Δ_vap_ *H* (kJ·mol^−1^)		Antoine parameters^a^		
	Exptl	Literature data	Exptl	Literature data	*A*	*B*	*C*
(CF_3_)_2_CHOCH_3_	51.1	50.95 [58]	32.7	32.6 [58, 59]	3.991	1012.1	−53.478
(CF_3_)_2_CHOCH_2_F (**1**)	58.3	58.58 [58]	34.3	34.3^b^ [58]	4.042	993.94	−50.418
(CF_3_)_2_CHOCHF_2_ (**2**)	42.0	42.12 [58]	31.5	31.1 [58, 59]	3.942	900.81	−51.128
(CF_3_)_2_CHOCF_3_ (**3**)	16.0	19 [55]	26.9	n.r.	4.155	1102.6	−58.518
(CF_3_)_2_CFOCF_3_ (**4**)	7.0	n.r.	25.3	n.r.	4.335	1201.8	−53.803
*n*C_3_F_7_OCH_3_	34.3	34.23 [60]	28.8	28.4 [61]	4.043	1012.8	−64.252
*n*C_3_F_7_OCHF_2_	23.5	n.r.	27.6	n.r.	3.993	932.40	−55.284
*n*C_3_F_7_OCF_3_	6.8	6.78 [62]	25.1	n.r.	3.922	885.51	−53.983
*i*C_4_F_9_OCH_3_	59.9	n.r.	32.6	n.r.	4.006	1096.7	−58.935
*i*C_4_F_9_OCH_2_F	67.8	n.r.	34.7	n.r.	4.144	1167.7	−58.727
*i*C_4_F_9_OCHF_2_	49.8	n.r.	31.4	n.r.	4.053	1080.0	−56.093
*i*C_4_F_9_OCF_3_	34.7	n.r.	28.8	n.r.	4.195	1114.4	−41.861
*t*C_4_F_9_OCH_3_	53.6	53.64 [63]	31.5	n.r.	4.023	1089.3	−55.616
*t*C_4_F_9_OCHF_2_	47.2	n.r.	30.9	n.r.	4.027	1061.5	−56.347
*t*C_4_F_9_OCF_3_	36.3	n.r.	29.1	n.r.	3.949	994.93	−57.118
*i*_0.8_/*n*_0.2_C_4_F_9_OCH_3_ ^d^	59.8	n.r.	32.5	n.r.	4.049	1119.6	−56.071
*i*_0.8_/*n*_0.2_C_4_F_9_OCHF_2_	49.8	n.r.	31.1	n.r.	4.190	1156.5	−46.614
*i*_0.8_/*n*_0.2_C_4_F_9_OCF_3_	35.1	n.r.	29.1	n.r.	4.041	1032.2	−52.418

^a^ log_10_(*p*·bar^−1^) = *A* − *B*·[*T* + C]^−1^.

^b^ Δ_vap_ *H* was determined using Antoine parameters reported in the literature.

^c^ n.r. = not reported.

^d^ commercially purchased as Novec7100.

**FIGURE 2:**
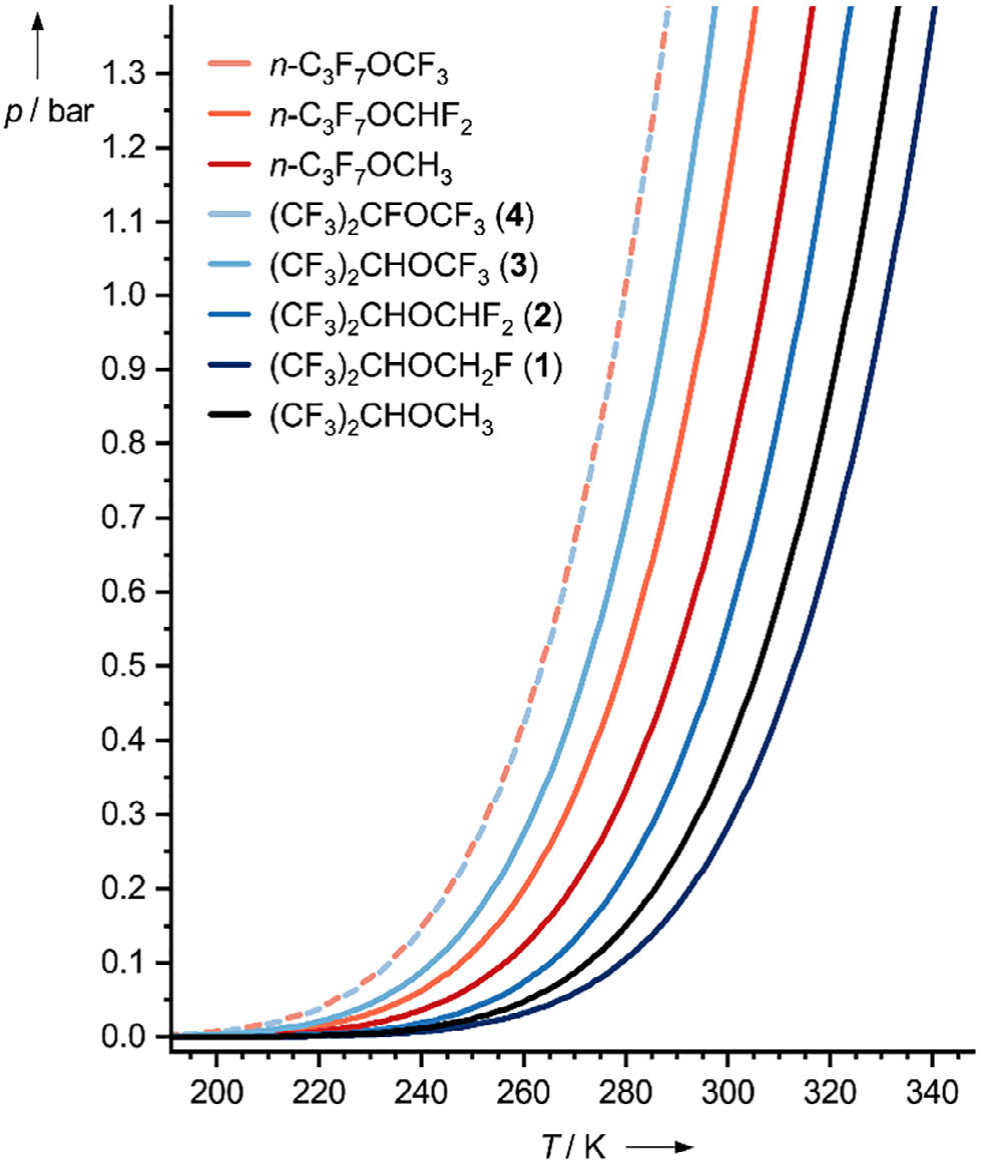
Vapor pressure curve (Antoine fit) of (CF_3_)_2_CHOCH_3_, **1**–**4**, *n*C_3_F_7_OCH_3_, *n*C_3_F_7_OCHF_2_, and *n*C_3_F_7_OCF_3_ (bottom).

The stepwise fluorination of sevoflurane (**1**) leads to a successive decrease of the boiling point in the order **1**> **2**> **3**> **4**, and the perfluorinated ether **4** reveals a boiling point of only 7.0°C (Table 2). This trend reflects a decrease in intermolecular interactions, which is often observed for compounds with an increasing degree of fluorination. In contrast, (CF_3_)_2_CHOCH_3_, which has fewer fluorine substituents, has a lower boiling point than **1** (Table 2). So, in the aforementioned series of ethers, **1** has the maximum of intermolecular interactions, which are mostly composed of hydrogen bonds, fluorine–fluorine, oxygen–fluorine, and electrostatic interactions in general (vide infra). Notably, the solubility of **1**, **2**, **3**, and **4** in aHF also decreases with an increasing number of fluorine atoms. This trend parallels the boiling points and the ability to participate in intermolecular interactions, that is, in hydrogen bonding with the solvent HF. The low solubility in combination with the high volatility facilitates the release of **2**, **3**, and **4** into the gas phase, preventing their decomposition during ECF and resulting in excellent yields of (CF_3_)_2_CHOCF_3_ (**3**) and (CF_3_)_2_CFOCF_3_ (**4**) (Scheme 3).

The vapor pressure curves of the two isomers **4** and *n*C_3_F_7_OCF_3_ are almost identical. The *n* isomer has a slightly lower boiling point of 6.8°C than its *iso* isomer (*T*_bp_ = 7.0°C). Similarly, the enthalpy of vaporization is slightly smaller for *n*C_3_F_7_OCF_3_ than for **4** (Table 2). Noteworthy, the boiling points of the related series of fluorinated ethers studied herein follow the general trend ─OCH_2_F > ─OCH_3_> ─OCHF_2_> ─OCF_3_ (Table 2).

### 2.4 Crystal Structures of Sevoflurane and Related Fluorinated Iso‐Propyl Methyl Ethers

Crystals of (CF_3_)_2_CHOCH_3_, (CF_3_)_2_CHOCH_2_F (**1**), (CF_3_)_2_CHOCHF_2_ (**2**), (CF_3_)_2_CHOCF_3_ (**3**), and (CF_3_)_2_CFOCF_3_ (**4**) were grown manually by in situ cryo‐crystallization [64, 65, 66, 67, 68, 69], and their structures were determined by SCXRD. Plots of the molecular structures of **1**–**4** derived from SCXRD and from DFT calculations, as well as plots of the electrostatic potentials (ESP), are depicted in Figure 3. Compounds **3** and **4** crystallize in the orthorhombic space group *Pnma* (*Z* = 4), and (CF_3_)_2_CHOCH_3_ in the orthorhombic space group *P*2_1_2_1_2_1_ (*Z* = 4). Compounds **1** and **2** crystallize in the monoclinic space groups *P*2_1_ (*Z* = 6) and *P*2_1_/*n* (*Z* = 4), respectively.

**FIGURE 3:**
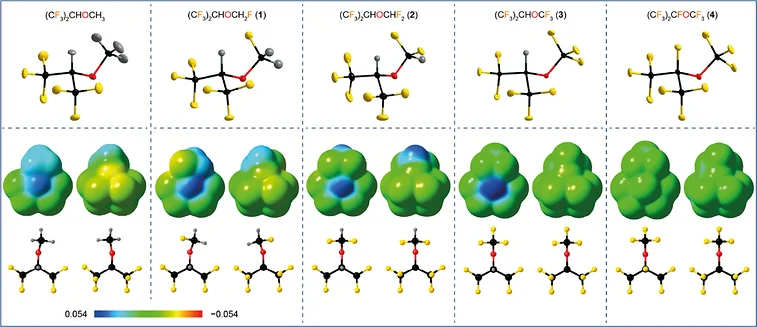
Crystal structures (top) and electrostatic potential (ESP) plots (bottom) of (CF_3_)_2_CHOCH_3_ and **1**–**4** (thermal ellipsoids set to the 25% probability level; H atoms of **1**–**3** were refined isotropically and of (CF_3_)_2_CHOCH_3_ anisotropically; DFT calculations: ωB97X‐D4/def2‐TZVPP).

Sevoflurane possesses *C*_1_ symmetry in the crystal, with the fluoromethoxy group revealing a *gauche* conformation, which agrees with calculated geometries and an earlier gas‐phase structure [70]. Both fluorine atoms of the ─CHF_2_ group in **2** are in a *gauche* position according to SCXRD and theory. DFT calculations predict a *gauche* conformation for (CF_3_)_2_CFOCHF_2_ (**3^iso^**), as well. The fluorine–*gauche* effect [36, 71, 72, 73], which is evident from the conformers of **1**, **2**, and **3^iso^**, is rationalized by an anomeric effect, that is, negative hyperconjugation. However, the importance of steric and electrostatic effects was also pointed out [74]. In the case of (CF_3_)_2_CHOCF_3_ (**3**), the influence of negative hyperconjugation of the trifluoromethoxy group can be deduced from the experimental and calculated C─F bond distances. The C─F distances of the fluorine atoms in the *gauche* position are significantly longer than the *d*(C─F) of the fluorine atom in the anti position (Table 3). The calculated C─F bond lengths for (CF_3_)_2_CFOCF_3_ (**4**) show that negative hyperconjugation is effective for the trifluoromethoxy group, but to a lesser extent than for **3**. Probably, the *iso*‐fluorine atom at the *iso*‐propyl group of **4** is the reason because it results in reduced electron density at the O atom. The C─F distances determined by SCXRD for the trifluoromethoxy group in **4** are very similar, and thus, the slightly shorter C─F distance found for the anti‐fluorine atom is not significant (Table 3).

**TABLE 3:** Selected experimental and calculated^a^ bond parameters^b^ of (CF_3_)_2_CHOCH_3_, (CF_3_)_2_CHOCH_2_F (**1**), (CF_3_)_2_CHOCHF_2_ (**2**), (CF_3_)_2_CHOCF_3_ (**3**), and (CF_3_)_2_CFOCF_3_ (**4**).

		sym	O─C^methyl^	O─C^propyl^	C^methyl^─F		C^methyl^─H^c^	C─CF_3_ ^c^	C^iso^─H/F	C─O─C	*τ*	
					gauche	anti					C─O─C─F^d^	C─C─O─C
(CH_3_)_2_CHOCH_3_	Calcd	*C*_1_	140.8	142.2	—	—	109.5	151.9^e^ 152.6^e^	110.1	114.4	—	166.7 −70.9
(CF_3_)_2_CHOCH_3_	SCXRD	*C*_1_	142.82(12)	139.10(9)	—	—	109.4(9)	152.22(12) 152.77(11)	107(2)	114.99(7)	—	113.23(8) −123.73(8)
	Calcd	*C*_1_	142.3	139.1	—	—	109.2	153.6 153.8	109.5	114.8	—	107.2 −128.3
**1**	SCXRD Mole1	*C*_1_	139.1(3)	141.0(3)	136.7(2)	—	111.1(4) 111.1(4)	152.8(3) 152.6(3)	109(3)	115.2(2)	−67.1(2)	128.1(2) −108.3(2)
	SCXRD Mole2	*C*_1_	139.2(3)	140.5(3)	137.7(3)	—	109(3) 108(4)	152.5(4) 152.8(3)	114(4)	115.7(2)	−73.0(3)	128.1(2) −109.2(2)
	SCXRD Mole3	*C*_1_	139.2(3)	140.3(2)	137.3(4)	—	111.1(3) 111.2(4)	153.0(4) 153.8(4)	111(4)	115.6(2)	74.3(3)	−129.1(2) 107.2(2)
	Gas^f^	*C*_1_	139.8(32)	139.0(76)	n.r.^g^	—	n.r.	153.2(34) 151.5(53)	n.r.	117(2)	−70(2)^h^	130.6(14) −102.3(10)
	Calcd	*C*_1_	138.9	140.3	137.9	—	108.9 109.3	153.7 153.8	109.3	115.5	−68.5	132.5 −103.4
**2**	SCXRD	*C*_1_	136.8(2)	141.2(2)	134.4(3) 135.0(2)	—	113(3)	153.1(3) 152.8(3)	111(2)	116.38(15)	56.9(2) −58.8(2)	117.6(2) −119.6(2)
	Calcd	*C*_s_	136.9	141.3	135.4	—	108.9	153.9	109.2	116.8	59.0	118.3
**3**	SCXRD	*C*_s_	136.1(3)	141.5(3)	132.9(2)	131.0(3)	—	153.1(2)	115(4)	116.28(17)	59.8(2)	118.68(16)
	Calcd	*C*_s_	136.1	141.4	133.9	132.1	—	154.0	109.1	116.9	60.1	118.2
**3^iso^**	Calcd	*C*_s_	138.2	139.3	134.6	—	108.9	155.8	135.1	121.9	60.1	118.4
**4**	SCXRD	*C*_s_	137.1(3)	140.2(3)	131.7(2)	132.4(4)	—	155.0(3)	133.7(3)	121.0(2)	61.3(3)	118.6(2)
	Calcd	*C*_s_	137.3	139.6	133.1	132.3	—	155.8	135.0	121.5	61.1	118.3

^a^ ωB97X‐D4/def2‐TZVPP.

^b^ Bond length in pm and angles in °.

^c^ Average values where applicable.

^d^ Fluorine atom in gauche orientation.

^e^ C─CH_3_.

^f^ Gas‐phase structure: Fourier‐transform microwave (FT‐MW) spectroscopy in a supersonic‐jet expansion [70].

^g^ n.r. = not refined.

^h^ Different fit than for the other values [70].

The experimental and calculated bond parameters listed in Table 3 reveal further distinct trends along the series of fluorinated methyl isopropyl ethers. Fluorination results, for example, in a shortening of the respective C─O bond; for example, the C─O^methyl^ distance decreases in the order (CF_3_)_2_CHOCH_3_> **1**> **2**> **3**. The C─O bond to the alkyl group that is not further fluorinated becomes longer in the same order. In line with this observation, the C─O^methyl^ distance in **3** is shorter than in **4**, and *d*(C─O^propyl^) is longer.

The highly fluorinated derivatives **2**–**4** and **3^iso^** are either *C*_s_ symmetrical or have a geometry close to *C*_s_ symmetry (Table 3). In contrast, the fluoromethyl group in sevoflurane is tilted. Similarly, the SCXRD and the calculated structure of (CF_3_)_2_CHOCH_3_ show a twist from the mirror plane. This distortion from *C*_s_ symmetry is predicted by theory to be even stronger for the non‐fluorinated parent ether (CH_3_)_2_CHOCH_3_ (Table 3).

The noncovalent intermolecular interactions in the crystals of (CF_3_)_2_CHOCH_3_ and **1**–**4** were evaluated by Hirshfeld surface analyses [75, 76, 77, 78, 79] using the program CrystalExplorer21 (Figure 4) [80, 81]. The 2D‐fingerprint plots [75, 76, 77, 78, 79, 80] of the fluorine⋯fluorine interactions [82, 83] show a steady increase with an increasing number of fluorine atoms. The bar charts summarizing all different contact types in Figure 4 visualize this trend and show a similar behavior for the oxygen⋯fluorine contacts. Noteworthy, the shortest O⋯F intermolecular distance in the crystal of the perfluorinated compound **4** of 289.2(3) pm is in the range of relatively short O⋯F contacts [84] (Figure 4).

**FIGURE 4:**
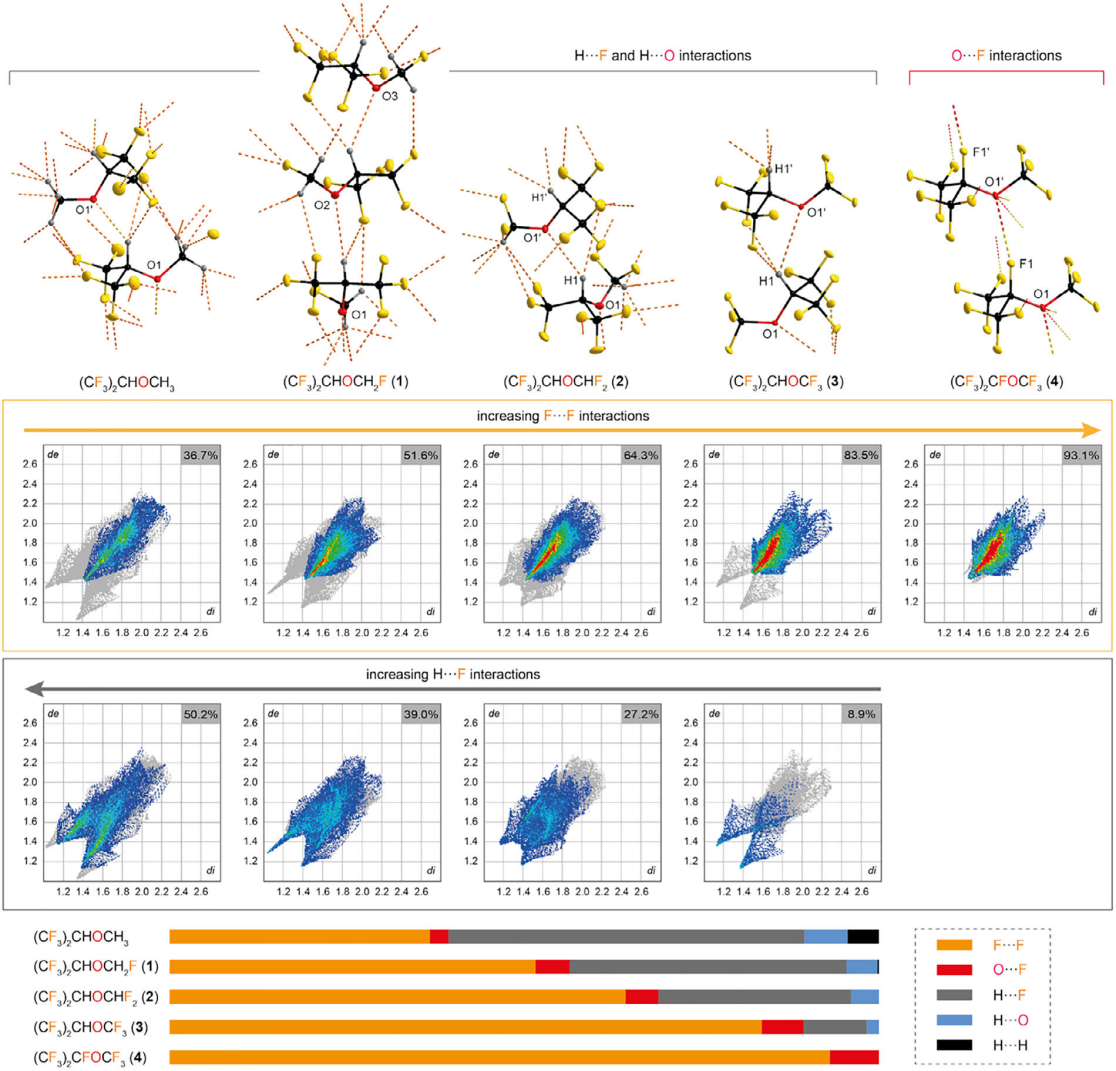
Excerpts of the crystal structures of (CF_3_)_2_CHOCH_3_ and **1**–**4** (thermal ellipsoids set to the 25% probability level; H atoms are depicted with arbitrary radii) highlighting the most relevant hydrogen bonds ((CF_3_)_2_CHOCH_3_ and **1**–**3**) or oxygen–fluorine interactions (**4**) (top), 2D‐fingerprint plots highlighting the intermolecular fluorine–fluorine interactions (F⋯F) and the hydrogen–fluorine bonds (H⋯F) including the Hirshfeld surface contribution of the respective interaction to the sum of the intermolecular interactions (in %) [80, 81] (middle), and bar chart summarizing the contributions of the different intermolecular interactions according to the 2D‐fingerprint analyses (bottom).

The 2D‐fingerprint plots of the H⋯F contacts and the bar charts (H⋯F and H⋯O contacts) reveal a decline in the hydrogen‐bonds with a decreasing number of hydrogen atoms in the series (CF_3_)_2_CHOCH_3_ and **1**–**3**. This analysis is supported by the excerpts of the crystal structures highlighting the most relevant intermolecular H⋯F and H⋯O contacts (Figure 4). The trend of decreasing relevance of hydrogen bonding agrees with the ESP plots in Figure 3 that show a decline in basic sites at the fluorine atoms and indicate a decrease in acidic hydrogen atoms. The general ability of **1** to take part in hydrogen bonding was assessed by theory earlier. This includes the formation of dimers of **1** [85] and (CF_3_)_2_CHOCH_2_F⋯benzene adducts in the gas phase [86] via hydrogen bonds.

## 3 Summary and Conclusions

Electrochemical fluorination in anhydrous HF (ECF, Simons process) provides a beneficial approach for the preparation of sevoflurane derivatives, that is, (CF_3_)_2_CFOCF_3_ (**4**), (CF_3_)_2_CHOCF_3_ (**3**), and (CF_3_)_2_CHOCHF_2_ (**2**). Since the Simons process is scalable and industrially established, these derivatives can be made accessible using the commercial anesthetic sevoflurane (**1**). The general applicability of ECF for the poly‐ and perfluorination using partially fluorinated ethers as starting compounds was demonstrated by the preparation of monofluoromethyl, difluoromethyl, and trifluoromethyl ethers starting from R^F^OCH_3_ (R^F^ = *n*C_3_F_7_, a mixture of *n*C_4_F_9_/*i*C_4_F_9_, *i*C_4_F_9_, *t*C_4_F_9_). Hence, ECF is a highly valuable tool for the preparation of partially as well as perfluorinated dialkyl ethers that are of high relevance for different applications.

The boiling point of sevoflurane and its analogues with a higher fluorine content decreases in the order **1**> **2**> **3**> **4**, that is, with increasing degree of fluorination. The boiling point, vapor pressure, and the enthalpy of vaporization (Δ_vap_ *H*) of (CF_3_)_2_CFOCF_3_ (**4**) and *n*C_3_F_7_OCF_3_ are very similar, showing that the intermolecular interactions of both perfluorinated isomers are close. The crystal structure analyses of (CF_3_)_2_CHOCH_3_ and **1**–**4** provide a unique insight into the bond parameters and the intermolecular interactions of a series of analogous compounds with a methoxy unit with different fluorine contents.

## Author Contributions

The synthetic work including the electrochemical fluorination (ECF) as well as the characterization was carried out by T.K. and L.N.S. The single‐crystal structure analyses were performed by T.K. DFT calculations were performed by E.R.C. The manuscript was written by T.K., N.V.I., and M.F. with contributions from all authors. The study was conceived by T.K., P.K., N.V.I., and M.F. All authors have given approval to the final version of the manuscript.

## Conflicts of Interest

The authors declare no conflicts of interest.

## Supporting information



The authors have cited additional references within the Supporting Information [87, 88, 89, 90, 91, 92, 93, 94, 95, 96, 97, 98, 99, 100, 101, 102, 103, 104]. Experimental procedures, spectra plots, details of the structural analyses, and DFT calculations can be found in the Supporting Information. Deposition Numbers 2565960 (**1**), 2565961 (**2**), 2565963 (**3**), 2565964 (**4**), 2565965 ((CF_3_)_2_CHOCH_3_), and 2565962 ((CF_3_)_2_CFCF_2_OCH_3_) contain the supplementary crystallographic data for this paper. These data are provided free of charge by the joint Cambridge Crystallographic Data Centre and Fachinformationszentrum Karlsruhe Access Structures service.
**Supporting File 1**: chem71589‐sup‐0001‐SuppMat.pdf

## Data Availability

The data that support the findings of this study are available in the Supporting Information of this article.
