# Supplementary material for: Electrochemical Fluorination of Partially Fluorinated Dialkylethers. Synthesis of Sevoflurane Derivatives (CF3)2CHOCHF2, (CF3)2CHOCF3, and (CF3)2CFOCF3
Source: Chemistry. 2026 Aug 19;32(41):e71589. doi: 10.1002/chem.71589 (PMC13729937; doi:10.1002/chem.71589)
Supplement: Supplementary file 1 [file CHEM-32-e71589-s001.pdf]

Supporting Information

Electrochemical Fluorination of Partially Fluorinated Dialkylethers. Synthesis of Sevoflurane Derivatives $(CF_3)_2CHOCHF_2$, $(CF_3)_2CHOCF_3$, and $(CF_3)_2CFOCF_3$

Tanja Knuplez,[a] Leon N. Schneider,[a] Erik R. Csapo, Peer Kirsch,[b] Nikolai V. Ignat'ev,[a] and Maik Finze*[a]

[a] Julius Maximilian University Würzburg, Institute of Inorganic Chemistry, Institute for Sustainable Chemistry & Catalysis with Boron (ICB), Am Hubland, 97074 Würzburg, Germany

[b] Merck Electronics KGaA, Frankfurter Str. 250, D-64293 Darmstadt, Germany

Contents

1. General S3
2. Chemicals S3
3. Synthesis and data S4
3.1. Electrochemical fluorination of sevoflurane (**1**) S4
3.2. Electrochemical fluorination of *n*-$C_3F_7OCH_3$ (Novec7000) S38
3.3. Characterization of $(CF_3)_2CHOCH_3$ S59
3.4. Electrochemical fluorination of $(CF_3)_3COCH_3$ S64
3.5. Electrochemical fluorination of $(CF_3)_2CFCF_2COCH_3$ S82
3.6. Electrochemical fluorination of $(CF_3)_2CFCF_2COCH_3/CF_3CF_2CF_2CF_2COCH_3$ (Novec7100) S102
4. Single crystal X-ray diffraction S122
5. References S124

1. General

^{1}H, ^{13}C, ^{17}O, and ^{19}F NMR spectra were recorded at 25 °C on solutions in $CDCl_3$ or on neat samples together with a closed capillary containing $(CD_3)_2CO$ and CCl_3F on a Bruker Avance NEO II 600 NMR spectrometer, a Bruker Avance NEO II 500 NMR spectrometer, or on a Bruker Avance NEO 400 NMR spectrometer. The NMR signals were referenced against TMS (^{1}H and ^{13}C), $CFCl_3$ (^{19}F), and H_2O (^{17}O) with $\Xi[^{17}O]$ = 13.556457, $\Xi[^{19}F]$ = 94.094011 MHz [1]. ^{1}H and ^{13}C chemical shifts were calibrated against the residual solvent signal ($CHCl_3$: $\delta(^{1}H)$ = 7.26 ppm, $(CHD_2)(CD_3)CO$: $\delta(^{1}H)$ = 2.05 ppm, $CDCl_3$: $\delta(^{13}C)$ = 77.16 ppm, $(CD_3)_2CO$: $\delta(^{13}C)$ = 29.84 and 206.26 ppm) [2]. ^{19}F chemical shifts were, in case of measurements with a capillary, calibrated against CCl_3F (CCl_3F: $\delta(^{19}F)$ = 0.00 ppm).

GC-MS analyses were performed using a Thermo Fisher Scientific Trace 1310 gas chromatograph (column: TG-SQC 5% phenyl methyl siloxane, 15 m, Ø 0.25 mm, film 0.25 µm; injector: 220 °C; oven: 40 °C (5 min), 40 °C to 280 °C (20 °C min^{-1}); carrier gas: He (1.2 mL min^{-1})) equipped with a Thermo Fisher Scientific AI 1310 autosampler/injector and a Thermo Fisher Scientific ISQ QD single quadrupole detector operating in EI (electron ionization) mode.

Thermal analyses were performed with a DSC 204 F1 Phoenix (Netzsch Gerätebau) in the temperature range of 0 to 550 °C with a heating rate of 10 K·min^{-1}. Measurements were performed in closed, sealed crucibles. In case of the more volatile compounds, the cooled substances were introduced into the crucibles under an inert argon atmosphere. Signals above 400 °C are due to the reaction of the substances with the aluminum crucible.

Vapor pressure measurements were carried out using a 1000 Torr *Baratron* absolute capacitance manometer from *MKS* combined with a PT100 thermometer of AA class made by *Greisinger*.

Density functional theory calculations (DFT) [3] were carried out using Orca 6.1 using the Resolution of Identity and Change of Sphere Exchange (RIJCOSX) methods with the def2/J auxiliary basis set on the standard defgrid2 hypersurface [4-5]. The functional ωB97X-D4 [6-8] and the def2-TZVPP [9-12] basis set were used for all atoms (level denoted as ωB97X-D4/def2-TZVPP). All calculated structures represent true minima without imaginary frequencies on the respective hypersurface. The computed frequencies were scaled [13] and convolved with a Gaussian-shaped function. Chemcraft (version 1.8) [14] was used for the analysis.

2. Chemicals

Anhydrous hydrogen fluoride (aHF) has been purchased from *GHC Gerling Holz & Co. Handels GmbH*. Sevoflurane was purchased from *AbbVie* and used without further purification. $(CF_3)_2CF_2OCH_3$ was purchased from *Carbosynth*, Novec 7100 (*i+n*-$C_4F_9OCH_3$) and $(CF_3)_2OCH_3$ from *TCI* and Novec7000 (*n*-$C_3F_7OCH_3$) from *Sigma Aldrich* and used without further purification. $(CF_3)_3COCH_3$ was received from Merck and distilled prior to use.

3. Synthesis and data

3.1. Electrochemical fluorination of sevoflurane (**1**)

A 1500 mL stainless steel cell with nickel electrodes (effective anode area *S* = 15.2 dm^2), the construction of which is described elsewhere [15] was filled with 1220 mL anhydrous hydrogen fluoride. A constant voltage of 5.0 V was applied overnight. $(CF_3)_2CHOCH_2F$ (1816.9 g, 9.082 mol) was added gradually to the pre-electrolyzed anhydrous HF. The gaseous products from the cell were collected in three subsequent PTFE traps maintained at −78 °C. All byproducts that were not collected in these PTFE traps, were allowed to pass through a $NaOH/H_2O$ scrubber to ensure decomposition of toxic gases, especially difluorophosgene and trifluoromethyl hypofluorite. The cell was operated at 4.5–6.0 V and the current was held between 2.1 and 4.0 A. The cell temperature was maintained at 5–8 °C and the temperature of the condenser was kept between −1 and −13 °C. After consumption of 1228 Ah the reaction was stopped. In total, 1732.3 g of crude material was collected from the cold traps during the experiment. Potassium fluoride (245.8 g, 4.23 mol) was suspended in the crude organic phase to remove the residual HF. The anhydrous HF from the cell was stored at −78 °C and subsequently poured onto ice (1200 g) but no organic layer formed.

The crude organic phase contained $(CF_3)_2CHOCH_2F$ (4%), $(CF_3)_2CHOCHF_2$ (20%), $(CF_3)_2CHOCF_3$ (58%), $(CF_3)_2CFOCF_3$ (18%), and other species in trace amounts. The separation of this mixture was carried out by distillation.

Table S1. Quantity of sevoflurane added according to passed electricity.

Electricity consumed: [Ah]	Quantity of Sevoflurane added [g]	Quantity of Sevoflurane added in total [g]
0	61.5	61.5
16.6	60.3	121.8
60.4	122.5	244.3
127.8	119.4	363.7
201.7	122.7	486.4
273.8	123.5	609.9
343.4	126.8	736.7
426.8	126.4	863.1
510.4	126.3	989.4
601.0	125.8	1115.2
695.8	125.5	1240.7
791.2	122.9	1363.6
892.1	129.6	1493.2
977.9	152.4	1646.1
1064.8	170.8	1816.9

Table S2. Composition of the crude material that was collected in the cold traps (1732.3 g) and yields with respect to the starting material sevoflurane (1816.9 g, 9.082 mol).

Compound	*m*(yield) [g]	*n*(yield) [mol]	yield [%]
$(CF_3)_2CHOCH_2F$ (**1**)	59.0	0.295	3.2
$(CF_3)_2CHOCHF_2$ (**2**)	322.0	1.477	16.3
$(CF_3)_2CHOCF_3$ (**3**)	1012.3	4.289	47.2
$(CF_3)_2CFOCF_3$ (**4**)	338.1	1.331	14.7
$(CF_3)_2CHOCH_2F$ (**1**) lost due to formation of byproducts during ECF incl. $(CF_3)_2CFOCHF_2$ (**3^{iso}**)		[1.690]	[18.6]

$(CF_3)_2CHOCH_2F$ (1) (sevoflurane)

Boiling point (vapor pressure): T_{bp} = 58.3 °C at 1013 mbar (Antoine parameter: A = 4.335; B = 1201.8; C = −53.803 for equation: $p = 10^{(A-B/(T+C))}$).

Enthalpy of vaporization (T = 298.15 K): $\Delta_{vap}H$ = 31.5 kJ mol^{-1} (for equation: $\Delta_{vap}H = \ln(10)\cdot R\cdot T^2\cdot(B/(T+C)^2)/1000$).

^{1}H NMR (600.2 MHz, $CDCl_3$): δ = 5.41 (d, 2H, $(CF_3)_2CHOC\mathbf{H}_2F$, $^2J(^{19}F, ^1H)$ = 53.5 Hz), 4.42 (sept, 1H, $(CF_3)_2C\mathbf{H}OCH_2F$, $^3J(^{19}F, ^1H)$ =5.81) ppm.

^{1}H{^{19}F} NMR (600.2 MHz, $CDCl_3$): δ = 5.41 (s, 2H, $(CF_3)_2CHOC\mathbf{H}_2F$), 4.42 (s, 1H, $(CF_3)_2C\mathbf{H}OCH_2F$) ppm.

^{13}C NMR (150.9 MHz, $CDCl_3$): δ = 120.9 (qm, 2C, $(\mathbf{C}F_3)_2CHOCH_2F$, $^1J(^{19}F,^{13}C)$ = 282.6 Hz), 102.9 9 (dtd, 1C, $(CF_3)_2CHO\mathbf{C}H_2F$, $^1J(^{19}F,^{13}C)$ = 226.2 Hz, $^1J(^{13}C, ^1H)$ = 178.0 Hz, $^3J(^{13}C, ^1H)$ = 6.4 Hz), 74.1 (dseptt, 1C, $(CF_3)_2\mathbf{C}HOCH_2F$, $^1J(^{13}C, ^1H)$ = 147.2 Hz, $^3J(^{19}F,^{13}C)$ = 33.6 Hz, $^3J(^{13}C, ^1H)$ = 6.9 Hz) ppm.

^{13}C{^{1}H} NMR (150.9 MHz, $CDCl_3$): δ = 120.9 (qm, 2C, $(\mathbf{C}F_3)_2CHOCH_2F$, $^1J(^{19}F,^{13}C)$ = 283.9 Hz), 102.9 (d, 1C, $(CF_3)_2CHO\mathbf{C}H_2F$, $^1J(^{19}F,^{13}C)$ = 224.0 Hz), 74.1 (sept, 1C, $(CF_3)_2\mathbf{C}HOCH_2F$, $^2J(^{19}F,^{13}C)$ = 33.5 Hz) ppm.

^{13}C{^{19}F} NMR (150.9 MHz, $CDCl_3$): δ = 120.9 (m, 2C, $(\mathbf{C}F_3)_2CHOCH_2F$), 102.9 (td, 1C, $(CF_3)_2CHO\mathbf{C}H_2F$, $^1J(^{13}C, ^1H)$ = 178.1 Hz, $^3J(^{13}C, ^1H)$ = 6.9 Hz), 74.1 (dt, 1C, $(CF_3)_2\mathbf{C}HOCH_2F$, $^1J(^{13}C, ^1H)$ = 146.9 Hz, $^3J(^{13}C, ^1H)$ = 6.9 Hz) ppm.

^{17}O NMR (81.4 MHz, $CDCl_3$): δ = 44.5 (s, 1O, $(CF_3)_2CH\mathbf{O}CH_2F$) ppm.

^{19}F NMR (564.7 MHz, $CDCl_3$): δ = −74.2 (dd, 1F, $(C\mathbf{F}_3)_2CHOCH_2F$, $^3J(^{19}F, ^1H)$ = 5.8 Hz, $^5J(^{19}F, ^{19}F)$ = 2.9 Hz), −154.7 (tsept, 1F, $(CF_3)_2CHOCH_2\mathbf{F}$, $^2J(^{19}F, ^1H)$ = 53.5 Hz, $^5J(^{19}F, ^{19}F)$ = 2.9 Hz) ppm.

^{19}F{^{1}H} NMR (564.7 MHz, $CDCl_3$): δ = −74.2 (d, 1F, $(C\mathbf{F}_3)_2CHOCH_2F$, $^5J(^{19}F, ^{19}F)$ = 2.9 Hz), −154.7 (sept, 1F, $(CF_3)_2CHOCH_2\mathbf{F}$, $^5J(^{19}F, ^{19}F)$ = 2.9 Hz) ppm.

GC-MS [t = 0.50 min] m/z$^+$: 199 [M]$^+$, 199 [M −H], 198 [M −H_2], 181 [M −F], 180 [M −HF], 151 [M −OCH_2F], 131 [M −CF_3], 69 [M −$F_3CCHOCH_2F$].

IR (gas phase, 20 °C, 1.5 mbar); wavenumber [cm^{-1}] (absorbance [%]): $\tilde{\nu}$ = 3030 (7), 2937 (8), 1383 (22), 1375 (31), 1372 (32), 1304 (70), 1274 (33), 1238 (77), 1225 (61), 1220 (69), 1185 (43), 1154 (8), 1135 (54), 1116 (52), 1037 (63), 1033 (52), 1027 (36), 907 (21), 880 (12), 744 (9), 693 (31).

Raman (liquid phase, 20 °C); wavenumber [cm^{-1}] (rel. Intensity [%]): $\tilde{\nu}$ = 3038 (6), 3012 (6), 2961 (13), 2943 (19), 2817 (6), 1500 (12), 1389 (6), 1372 (7), 1295 (7), 1284 (7), 1128 (6), 1110 (6), 1025 (11), 878 (42), 744 (100), 605 (9), 540 (6), 361 (12), 330 (32), 303 (16), 228 (7), 70 (6), 61 (6).

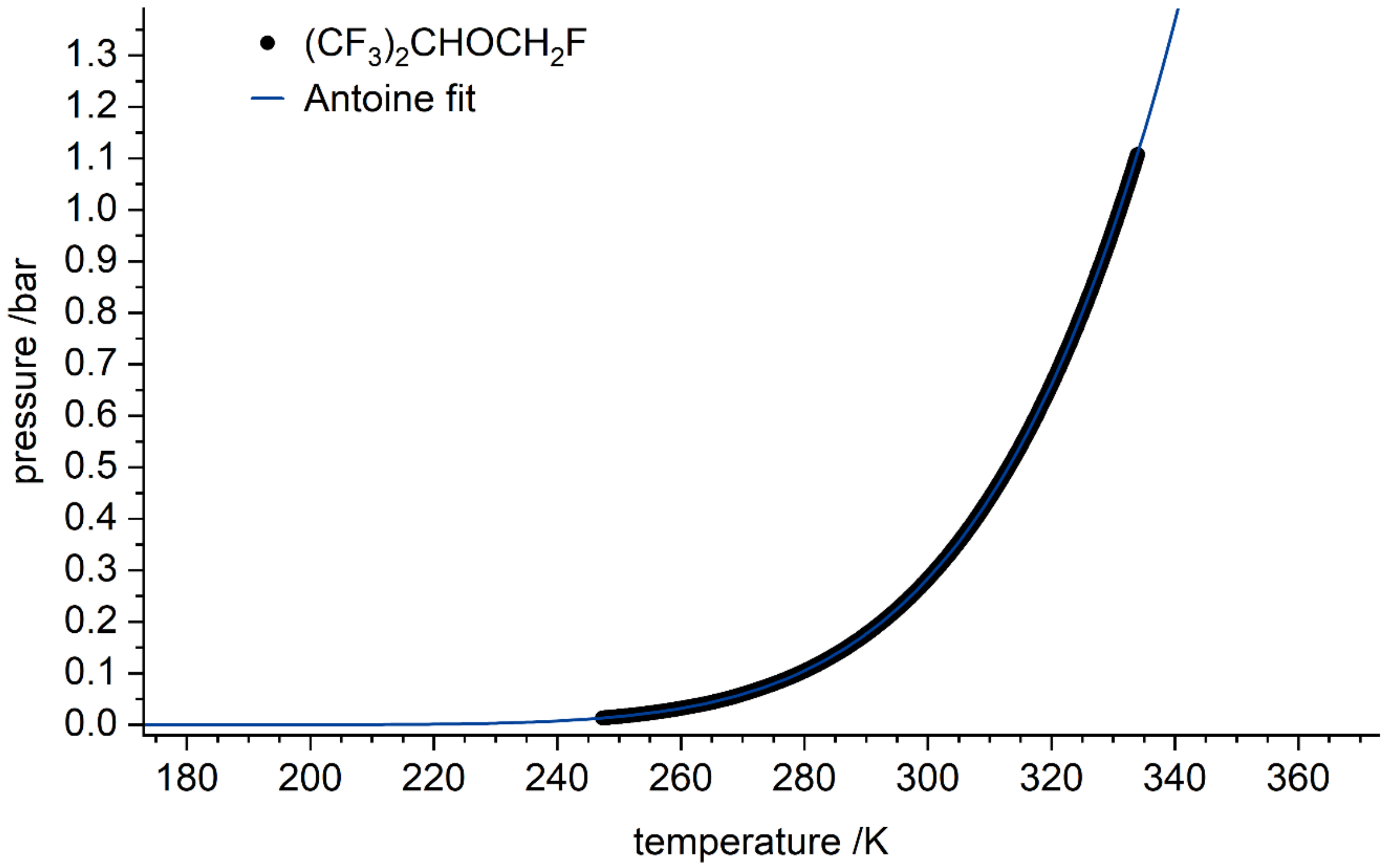

Figure S1. Vapor pressure measurement and Antoine fit of $(CF_3)_2CHOCH_2F$ (**1**) (Antoine parameter: A = 4.335; B = 1201.8; C = −53.803 for equation: $p = 10^{(A-B/(T+C))}$).

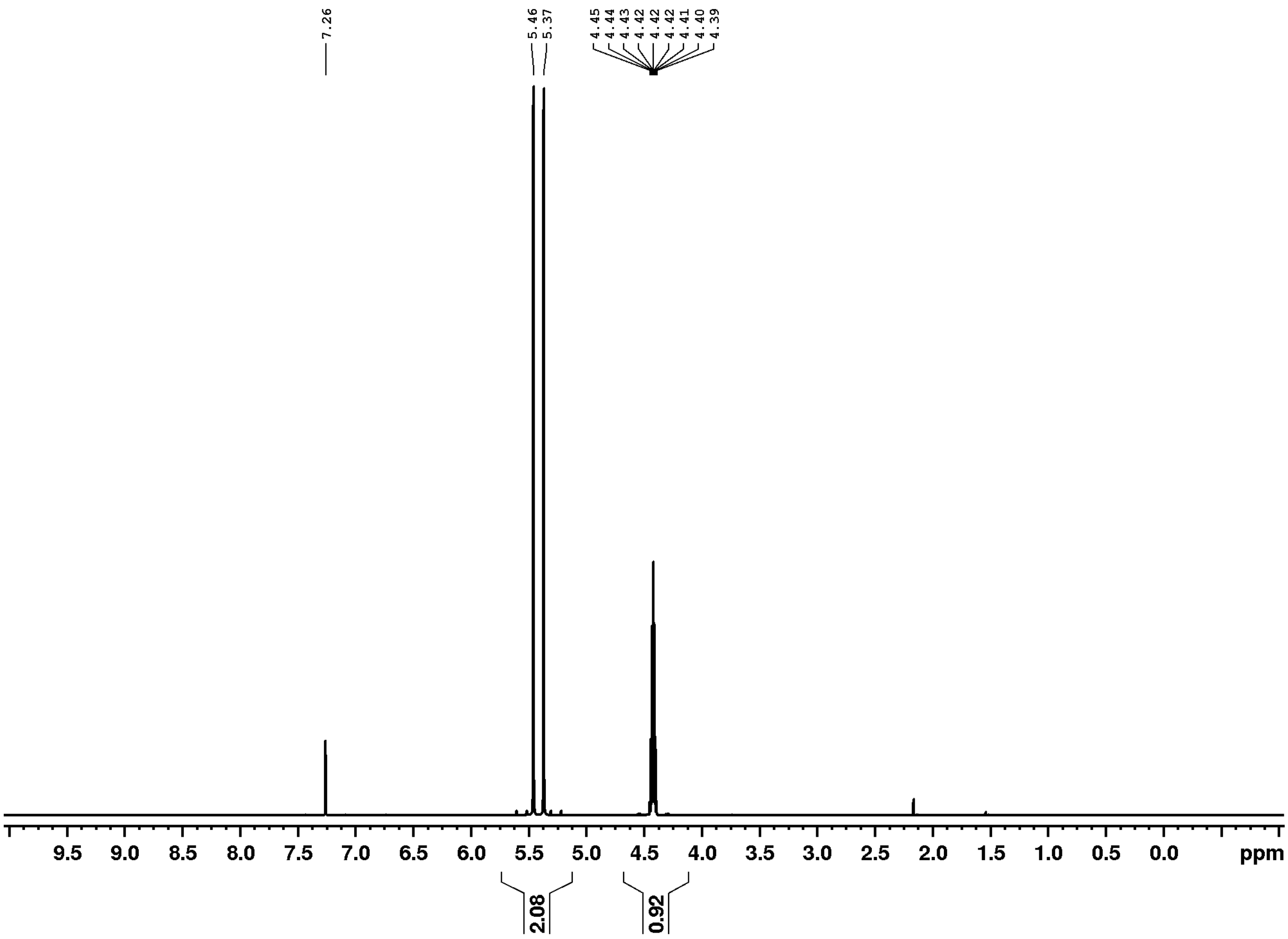

Figure S2. ^{1}H NMR of $(CF_3)_2CHOCH_2F$ (**1**) in $CDCl_3$.

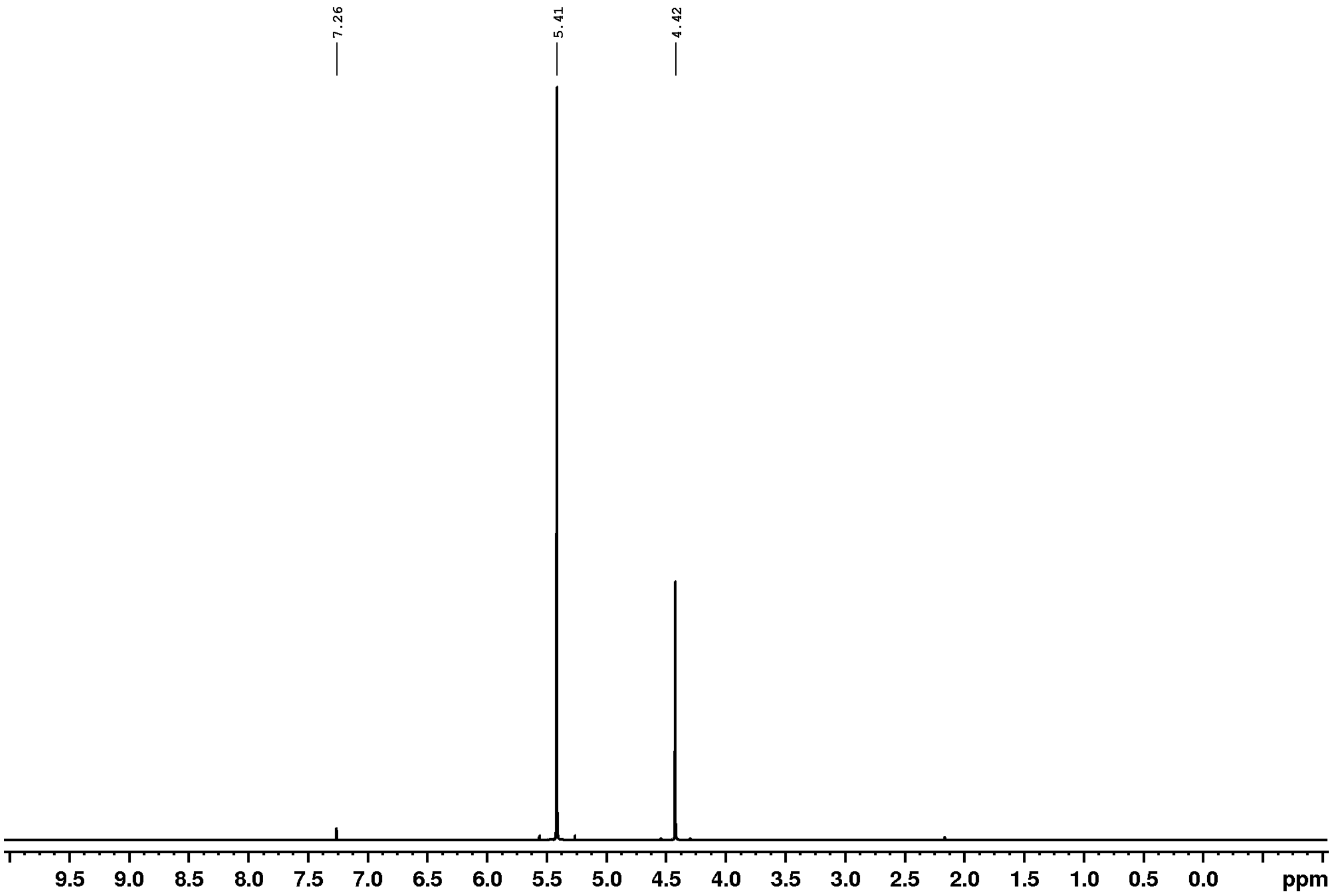

Figure S3. $^1H\{^{19}F\}$ NMR of $(CF_3)_2CHOCH_2F$ (**1**) in $CDCl_3$.

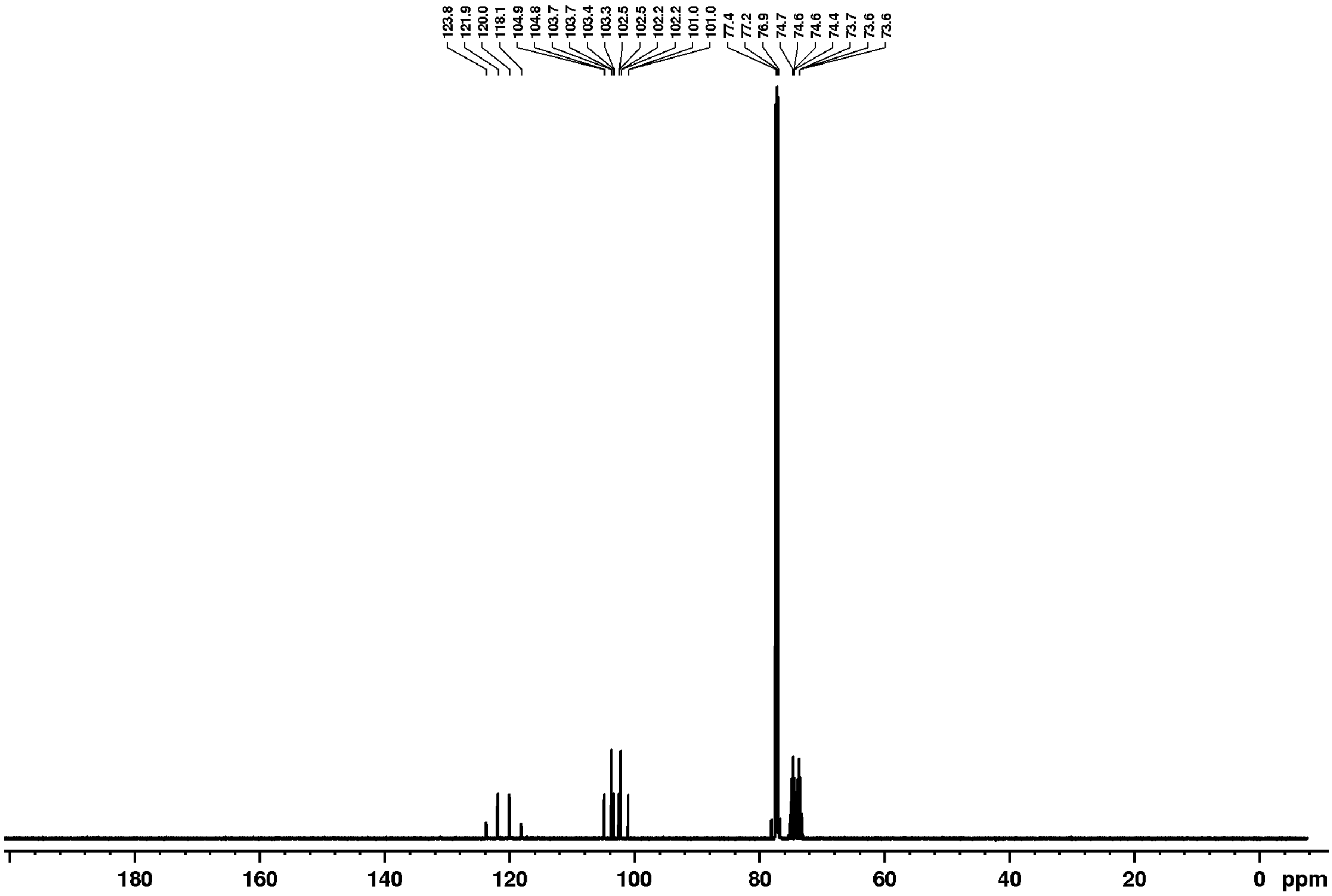

Figure S4. ^{13}C NMR of $(CF_3)_2CHOCH_2F$ (**1**) in $CDCl_3$.

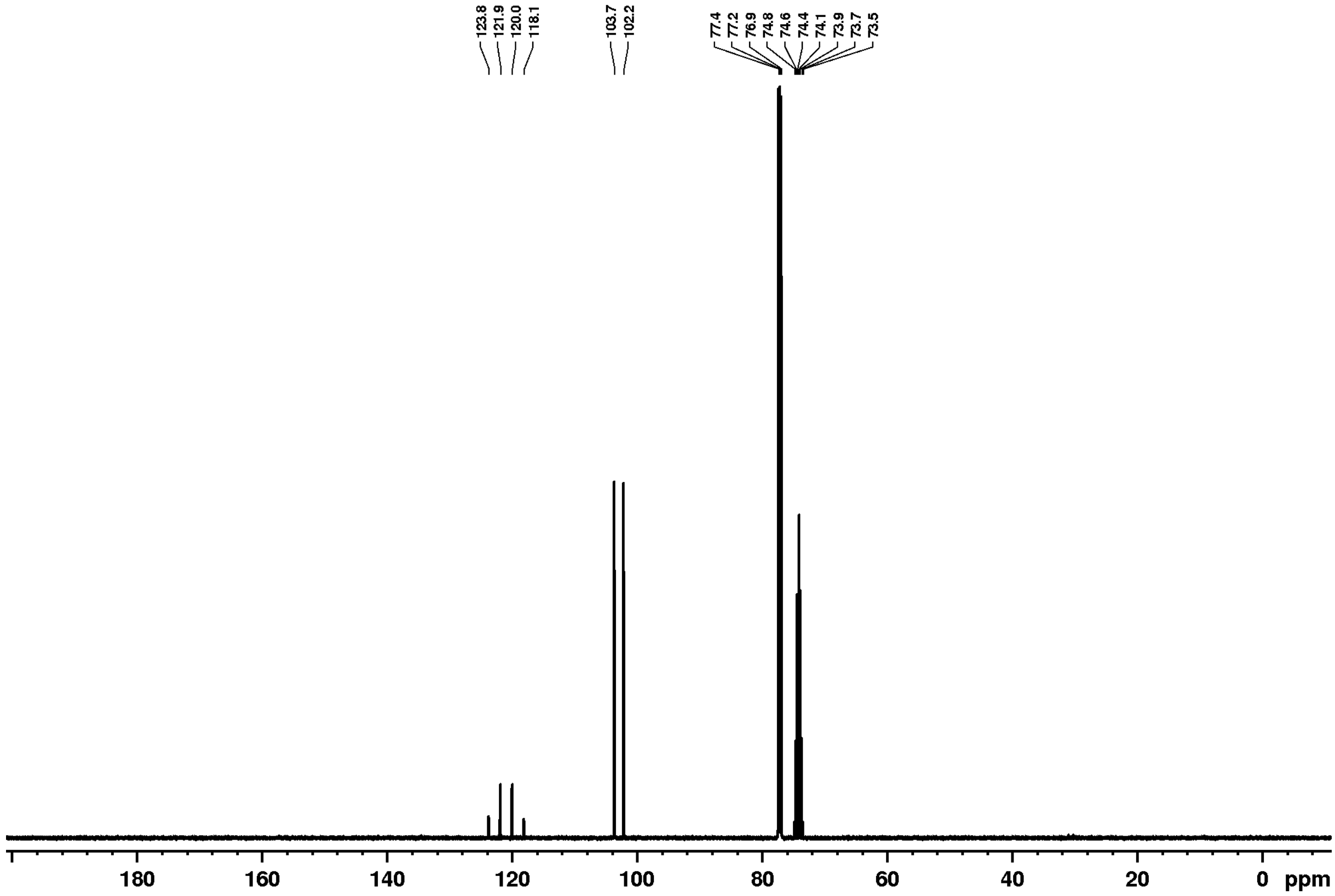

Figure S5. $^{13}C\{^{1}H\}$ NMR of $(CF_3)_2CHOCH_2F$ (**1**) in $CDCl_3$.

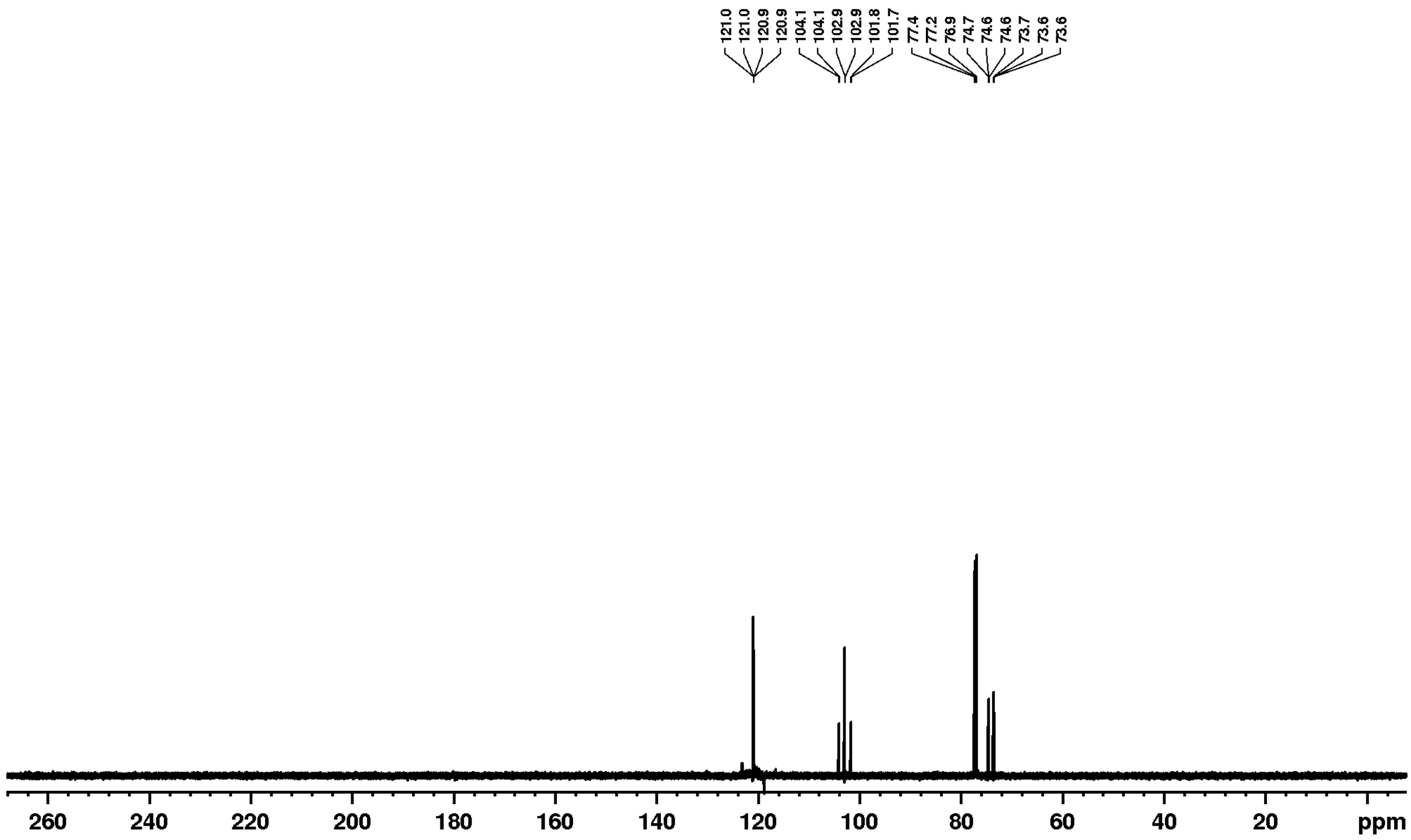

Figure S6. $^{13}C\{^{19}F\}$ NMR of $(CF_3)_2CHOCH_2F$ (**1**) in $CDCl_3$.

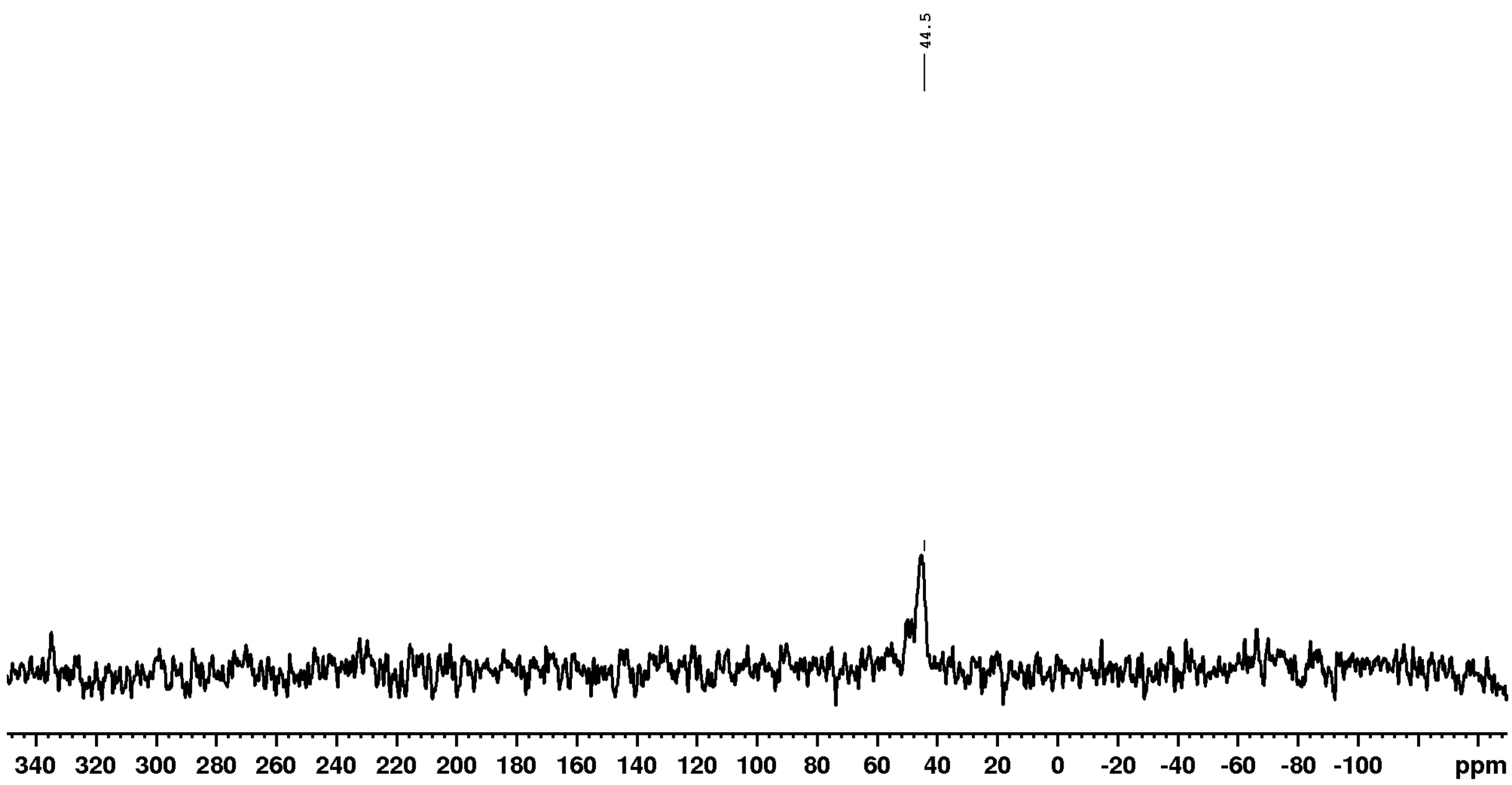

Figure S7. ^{17}O NMR of $(CF_3)_2CHOCH_2F$ (**1**) in $CDCl_3$.

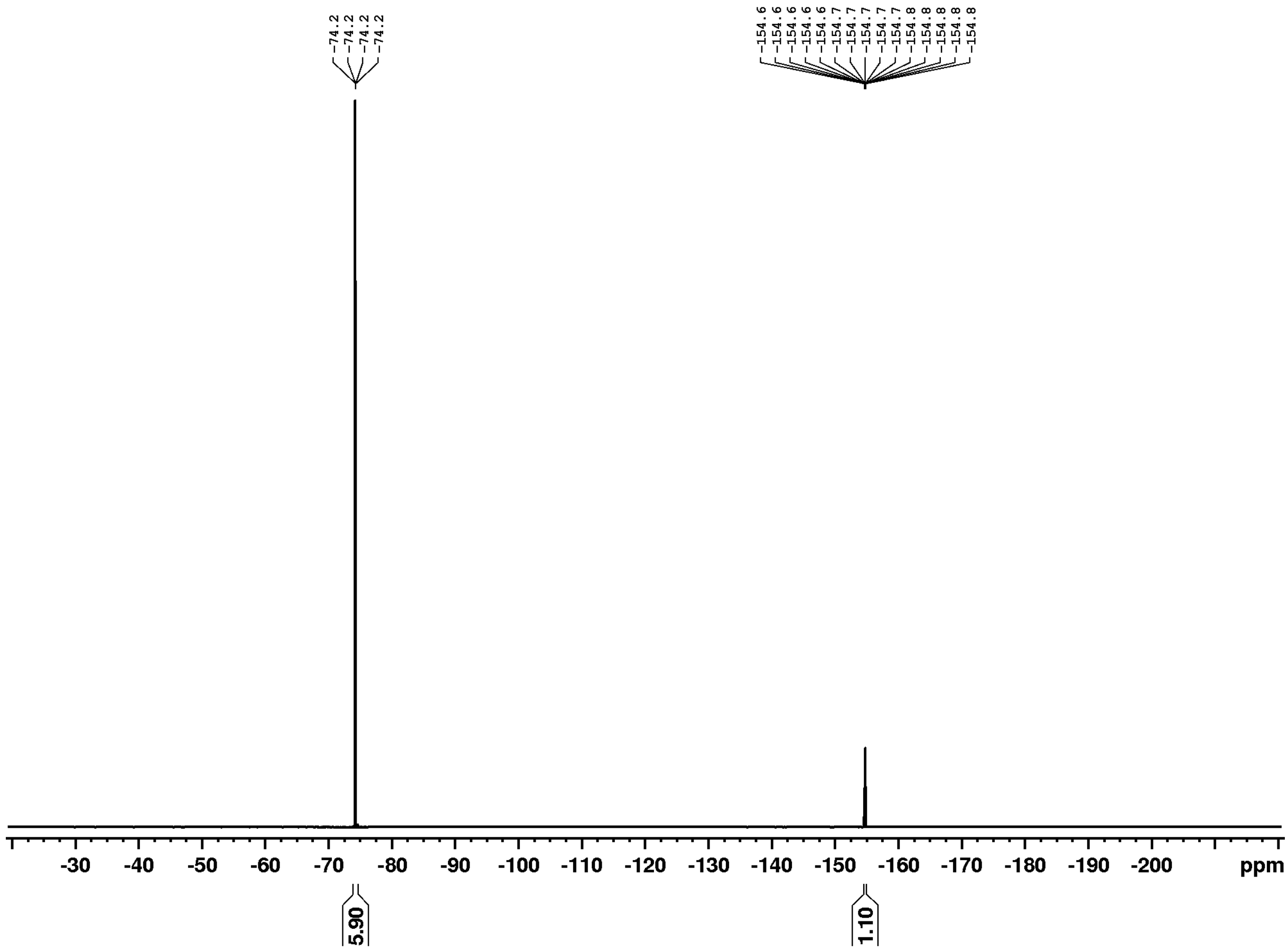

Figure S8. ^{19}F NMR of $(CF_3)_2CHOCH_2F$ (**1**) in $CDCl_3$.

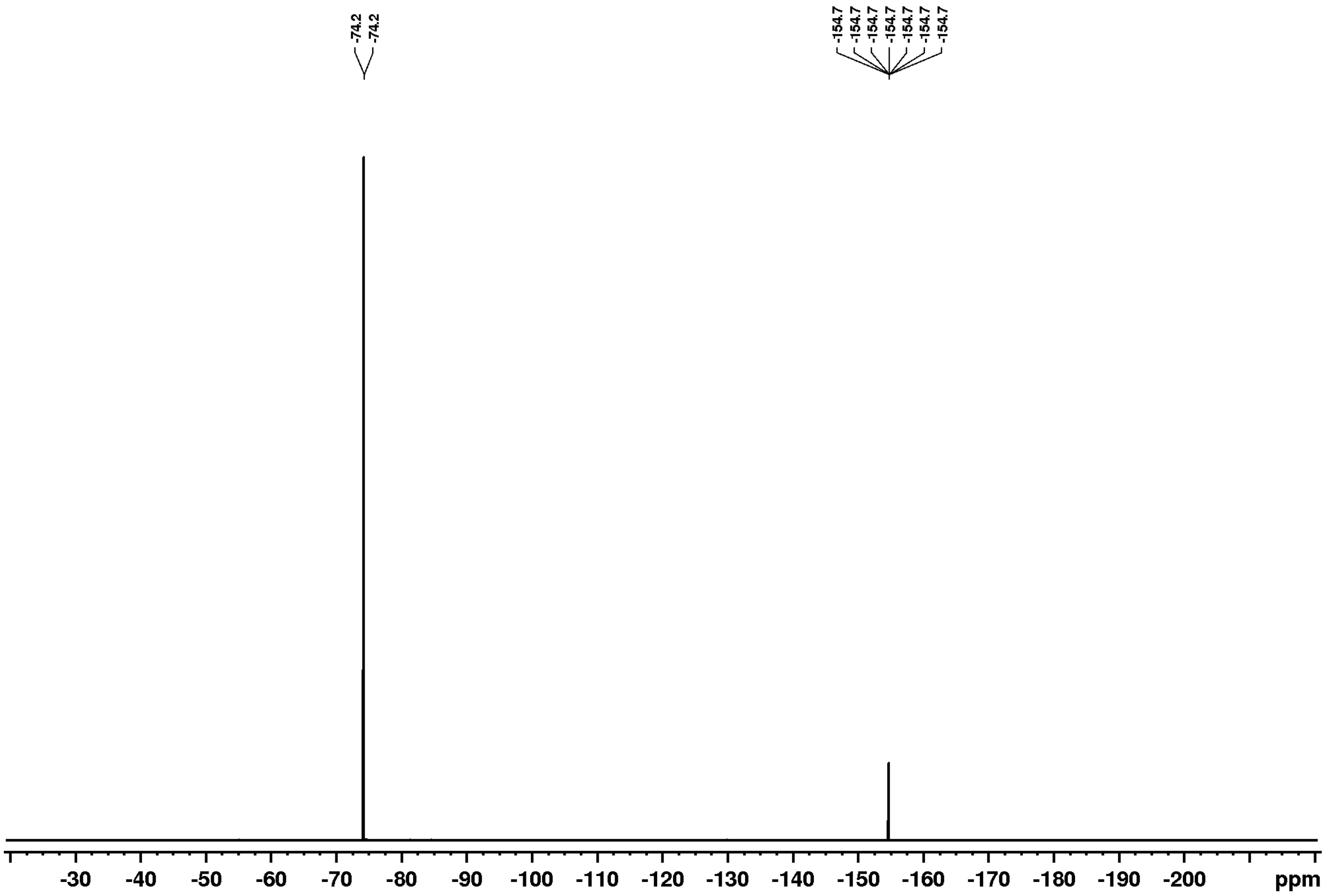

Figure S9. $^{19}F\{^{1}H\}$ NMR of $(CF_3)_2CHOCH_2F$ (**1**) in $CDCl_3$.

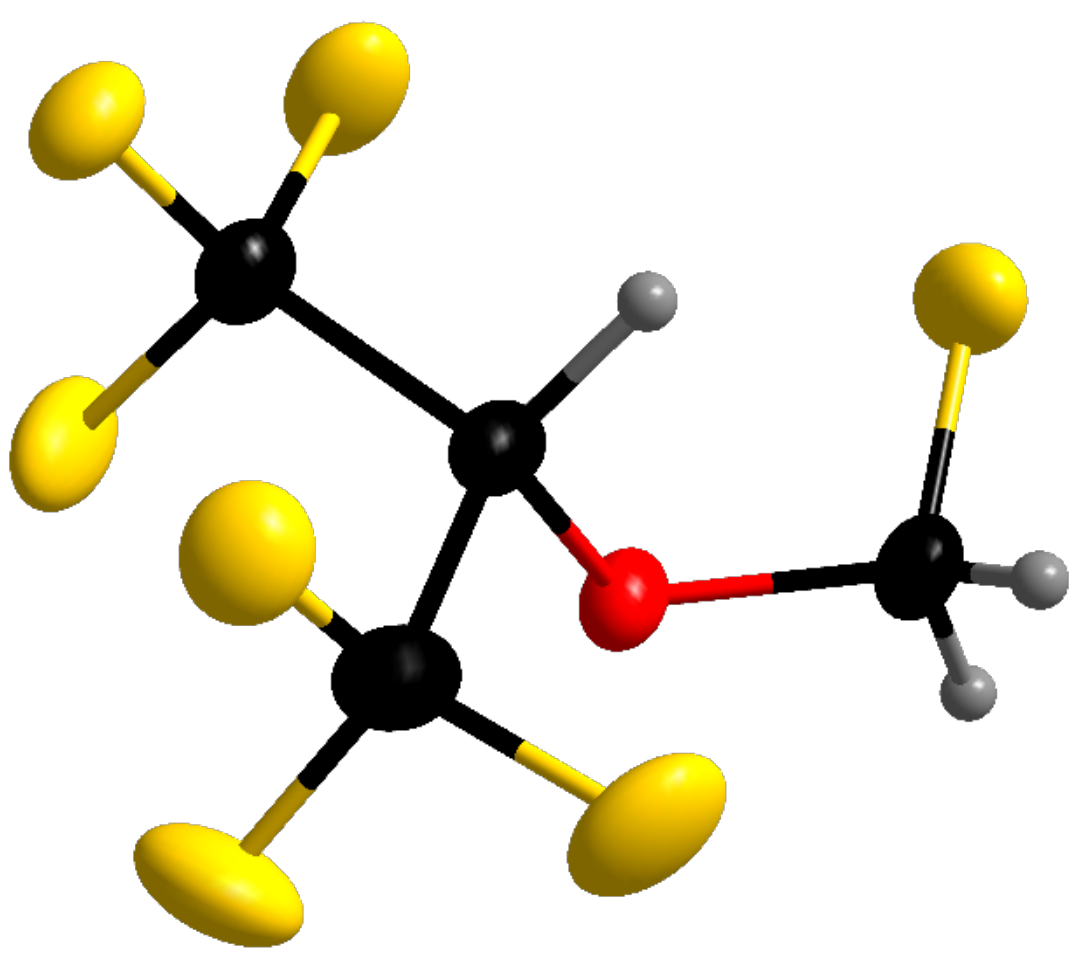

Figure S10. Solid state structure of $(CF_3)_2CHOCH_2F$ (**1**).

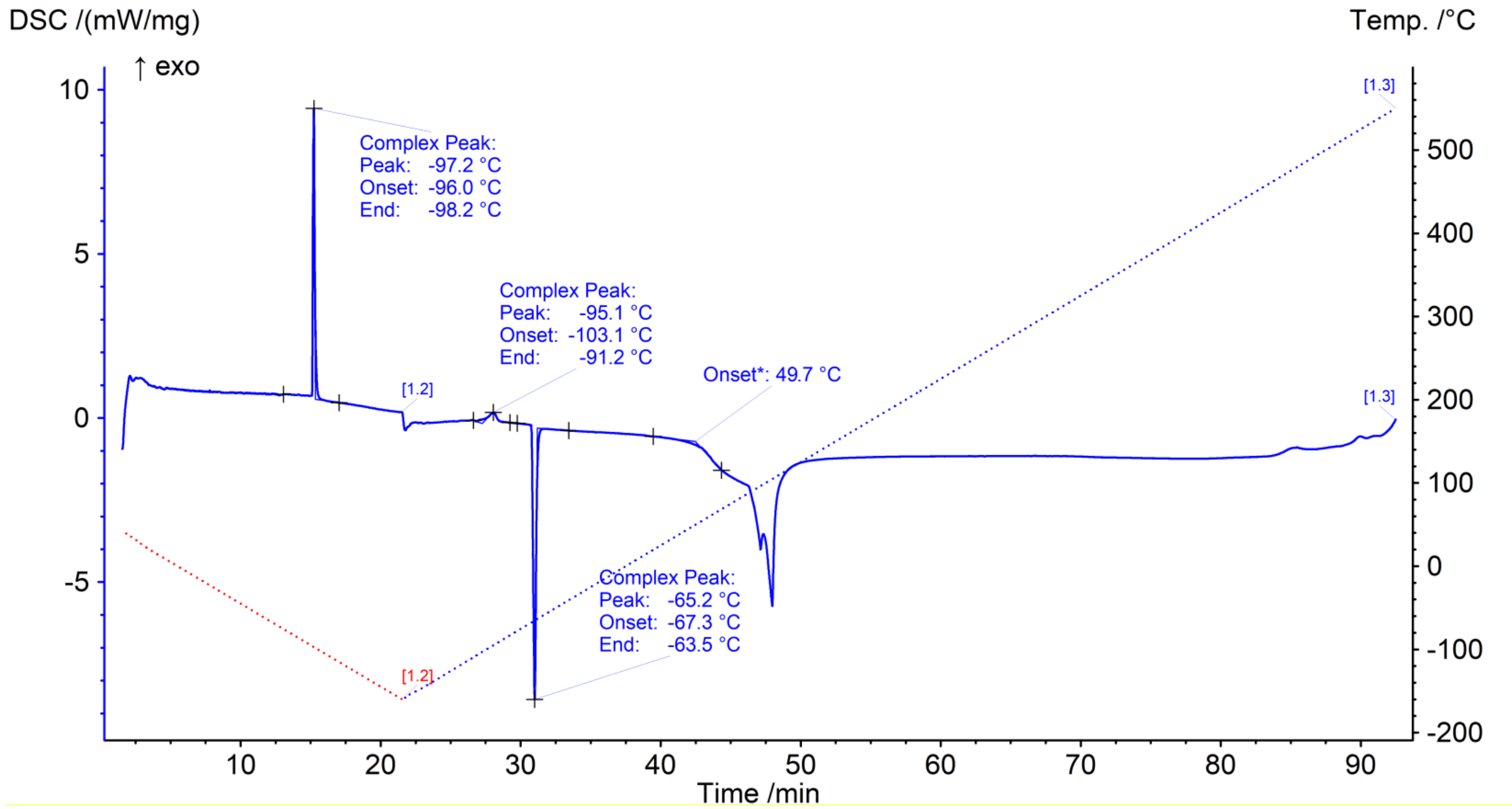

Figure S11. DSC curve of $(CF_3)_2CHOCH_2F$ (**1**).

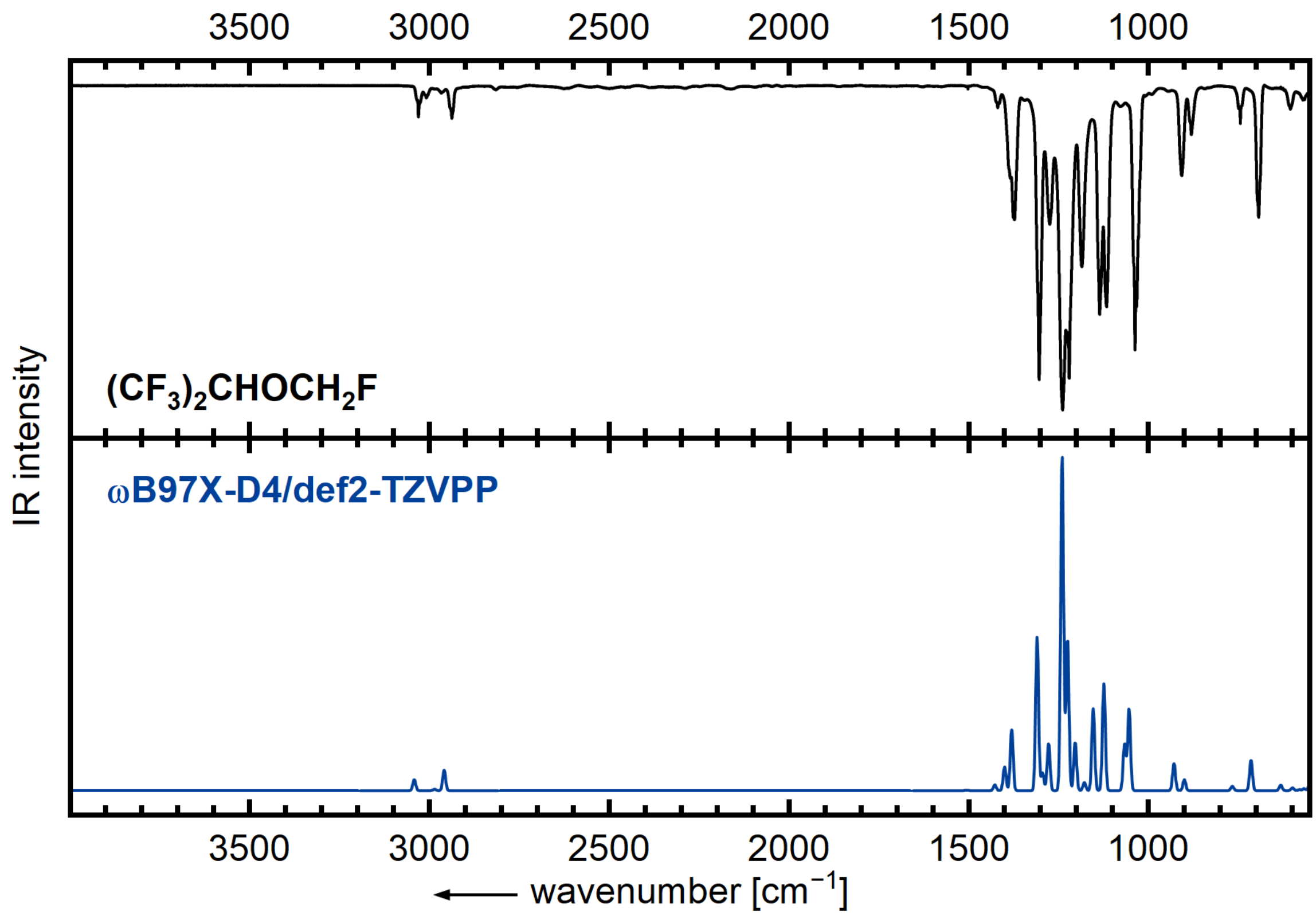

Figure S12. IR spectrum of $(CF_3)_2CHOCH_2F$ (**1**, gas phase): measured (top) vs. calculated (ωB97X-D4/def2-TZVPP, bottom).

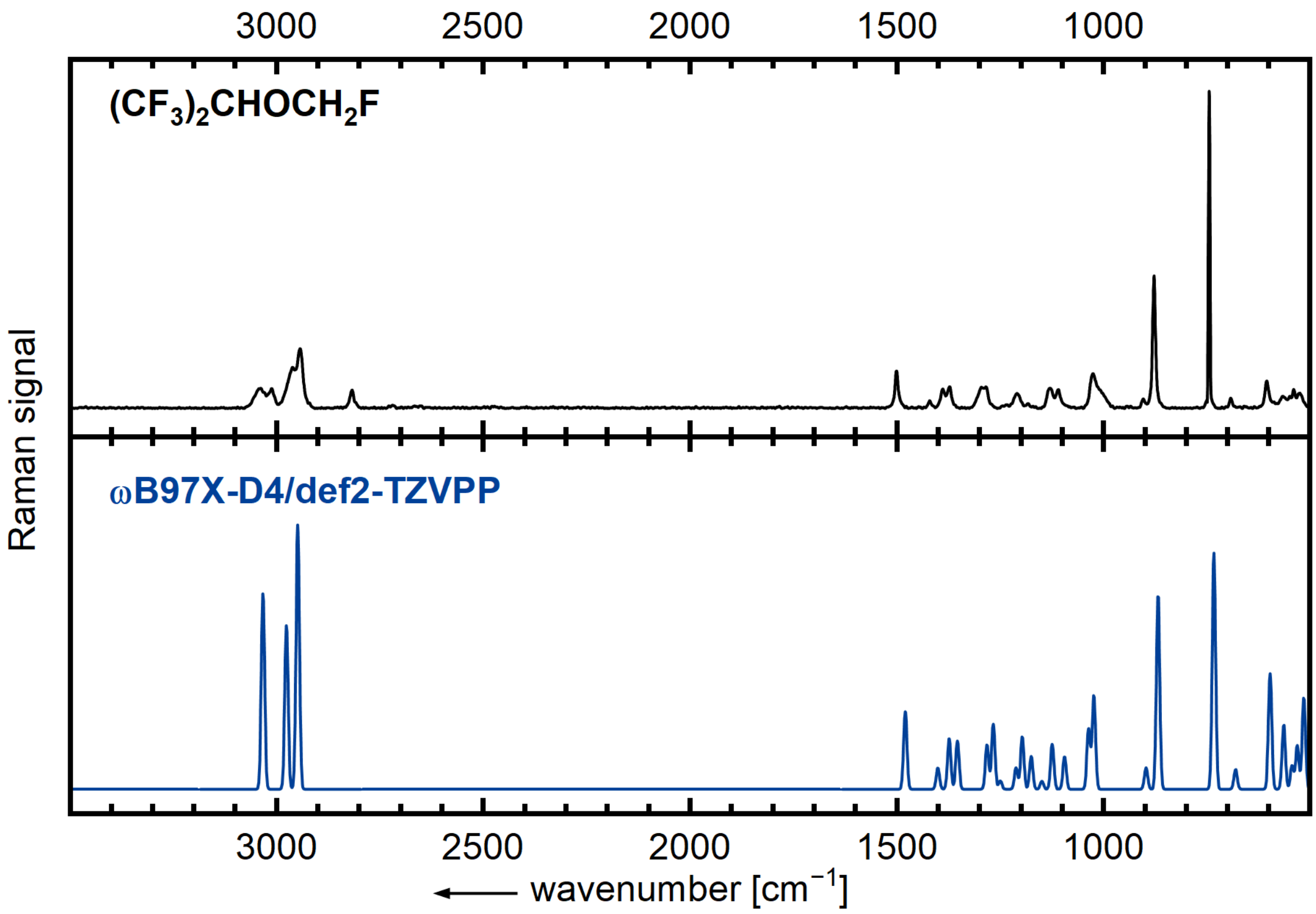

Figure S13. Raman spectrum of $(CF_3)_2CHOCH_2F$ (**1**, liquid phase): measured (top) vs. calculated (ωB97X-D4/def2-TZVPP, bottom).

$(CF_3)_2CHOCHF_2$ (2)

Boiling point (distillation): T_{bp} = 42 °C at 1000 mbar.

Boiling point (vapor pressure): T_{bp} = 42.0 °C at 1013 mbar (Antoine parameter: A = 4.043; B = 1012.8; C = −64.252 for equation: $p = 10^{(A-B/(T+C))}$).

Enthalpy of vaporization (T = 298.15 K): $\Delta_{vap}H$ = 34.3 kJ mol^{-1} (for equation: $\Delta_{vap}H = \ln(10)\cdot R\cdot T^2\cdot(B/(T+C)^2)/1000$).

^{1}H NMR (500.1 MHz, $CDCl_3$): δ = 6.42 (t, 1H, $(CF_3)_2CHOC\mathbf{H}F_2$, $^2J(^{19}F, ^1H)$ = 70.6 Hz), 4.83 (sept, 1H, $(CF_3)_2C\mathbf{H}OCHF_2$, $^3J(^{19}F, ^1H)$ = 5.6 Hz) ppm.

^{1}H{^{19}F} NMR (400.3 MHz, $CDCl_3$): δ = 6.42 (d, 1H, $(CF_3)_2CHOC\mathbf{H}F_2$, $^4J(^1H, ^1H)$ = 0.6 Hz), 4.83 (d, 1H, $(CF_3)_2C\mathbf{H}OCHF_2$, $^4J(^1H, ^1H)$ = 0.6 Hz) ppm.

^{13}C{^{1}H} NMR (125.8 MHz, $CDCl_3$): δ = 120.4 (qm, 2C, $(\mathbf{C}F_3)_2CHOCHF_2$, $^1J(^{19}F,^{13}C)$ = 282.8 Hz), 114.5 (t, 1C, $(CF_3)_2CHO\mathbf{C}HF_2$, $^1J(^{19}F,^{13}C)$ = 270.7 Hz), 68.6 (sept t, 1C, $(CF_3)_2\mathbf{C}HOCHF_2$, $^2J(^{19}F,^{13}C)$ = 35.1 Hz, $^3J(^{19}F,^{13}C)$ = 4.8 Hz) ppm.

^{13}C{^{19}F} NMR (125.8 MHz, $CDCl_3$): δ = 120.4 (d, 2C, $(\mathbf{C}F_3)_2CHOCHF_2$, $^2J(^{13}C, ^1H)$ = 6.0 Hz), 114.5 (dd, 1C, $(CF_3)_2CHO\mathbf{C}HF_2$, $^1J(^{13}C, ^1H)$ = 230.2 Hz, $^3J(^{13}C, ^1H)$ = 6.0 Hz), 68.6 (dd, 1C, $(CF_3)_2\mathbf{C}HOCHF_2$, $^1J(^{13}C, ^1H)$ = 148.9 Hz, $^3J(^{13}C, ^1H)$ = 5.4 Hz) ppm.

^{17}O NMR (54.3 MHz, $CDCl_3$): δ = 66.2 (s, 1O, $(CF_3)_2CH\mathbf{O}CHF_2$) ppm.

^{19}F NMR (470.6 MHz, $CDCl_3$): δ = −74.2 (dt, 6F, $(C\mathbf{F}_3)_2CHOCHF_2$, $^3J(^{19}F, ^1H)$ = 5.5 Hz, $^5J(^{19}F, ^{19}F)$ = 4.4 Hz)), −85.2 (dsept, 2F, $(CF_3)_2CHOCH\mathbf{F}_2$, $^2J(^{19}F, ^1H)$ = 70.6 Hz, $^5J(^{19}F, ^{19}F)$ = 4.4 Hz) ppm.

^{19}F{^{1}H} NMR (376.6 MHz, $CDCl_3$): δ = −74.2 (t, 6F, $(C\mathbf{F}_3)_2CHOCHF_2$, $^5J(^{19}F, ^{19}F)$ = 4.4 Hz), −85.1 (sept, 2F, $(CF_3)_2CHOCH\mathbf{F}_2$, $^5J(^{19}F, ^{19}F)$ = 4.4 Hz) ppm.

GC-MS [t = 0.47 min] m/z$^+$: 217 [M]$^+$, 199 [M −F], 151 [M −$OCHF_2$], 149 [M −CF_3], 69 [M −$CF_3CHOCHF_2$], 51 [M −$(CF_3)_2CHO$].

IR (gas phase, 20 °C, 1.6 mbar); wavenumber [cm^{-1}] (absorbance [%]): $\tilde{\nu}$ = 3048 (8), 2998 (3), 1405 (8), 1377 (59), 1304 (70), 1268 (36), 1267 (36), 1252 (86), 1221 (76), 1179 (78), 1157 (27), 1120 (87), 1089 (45), 1024 (8), 903 (27), 714 (29), 711 (24), 694 (35), 692 (34), 522 (9), 521 (8).

Raman (liquid phase, 20 °C); wavenumber [cm^{-1}] (rel. Intensity [%]): $\tilde{\nu}$ = 3055 (15), 2990 (20), 1386 (18), 1376 (21), 1300 (11), 1112 (11), 1021 (26), 895 (100), 878 (15), 768 (51), 752 (17), 714 (61), 595 (14), 538 (15), 520 (14), 437 (12), 331 (58), 320 (37), 231 (31).

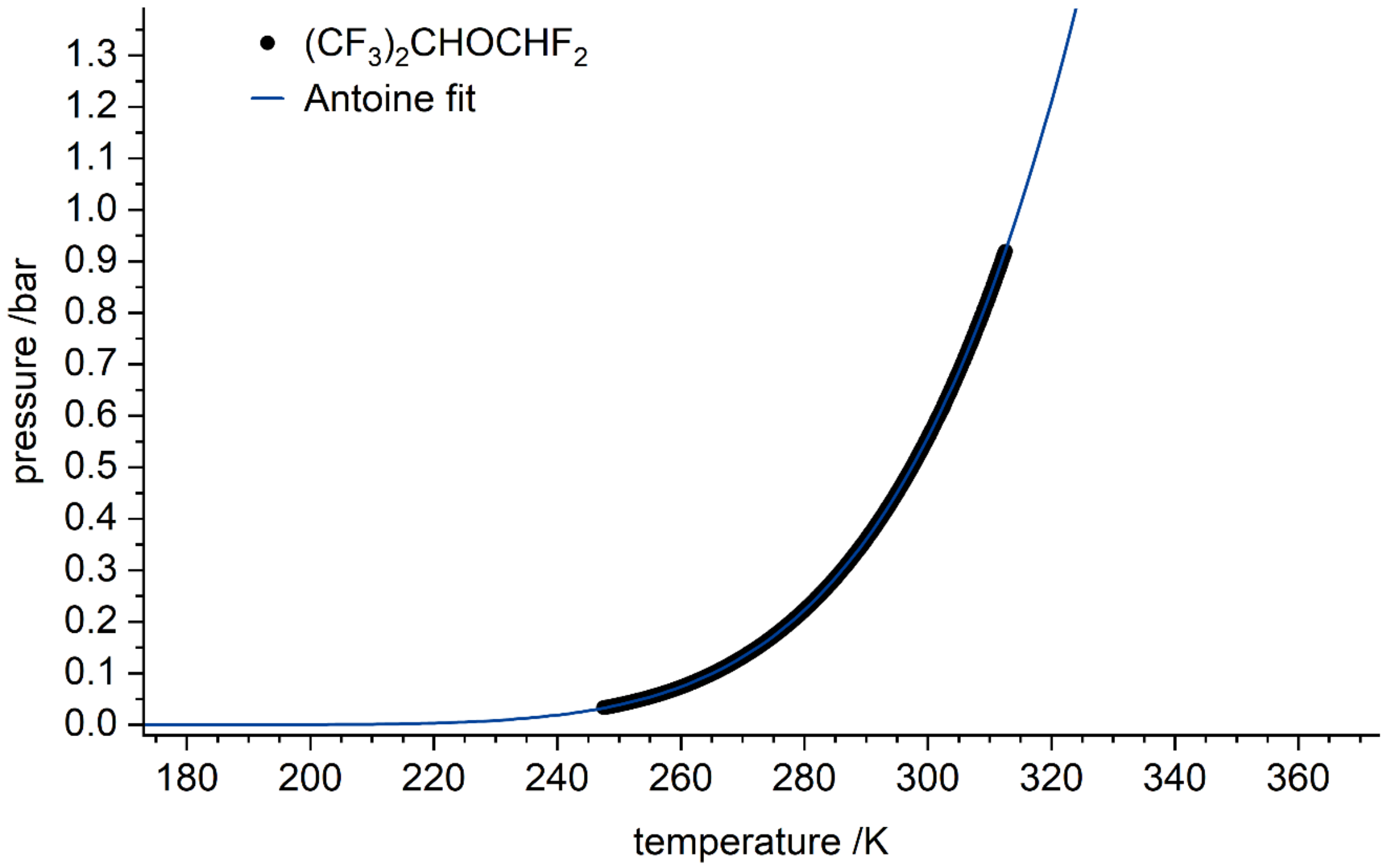

Figure S14. Vapor pressure measurement and Antoine fit of $(CF_3)_2CHOCHF_2$ (**2**) (Antoine parameter: A = 4.043; B = 1012.8; C = −64.252 for equation: $p = 10^{(A-B/(T+C))}$).

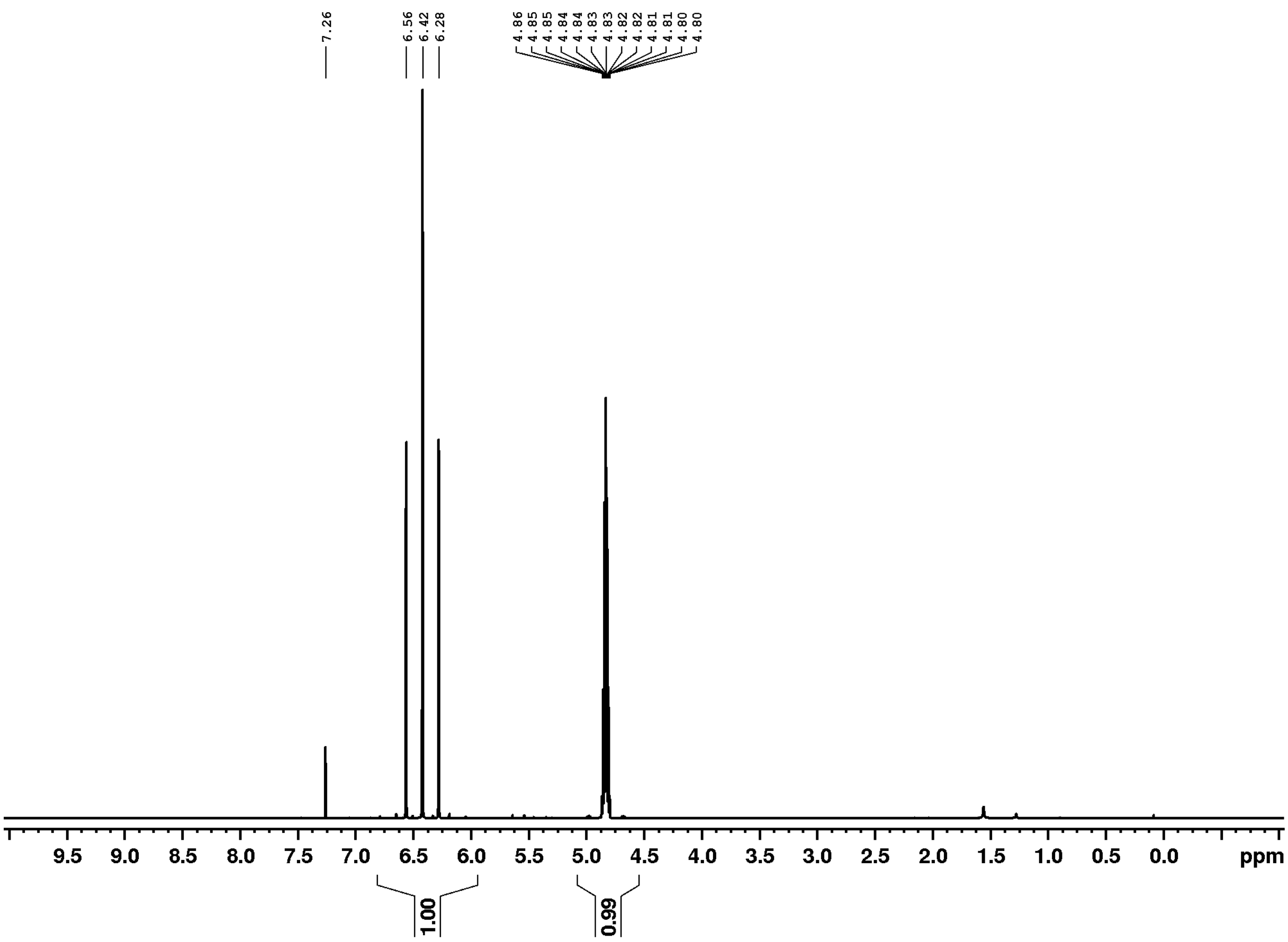

Figure S15. ^1H NMR of $(CF_3)_2CHOCHF_2$ (**2**) in $CDCl_3$.

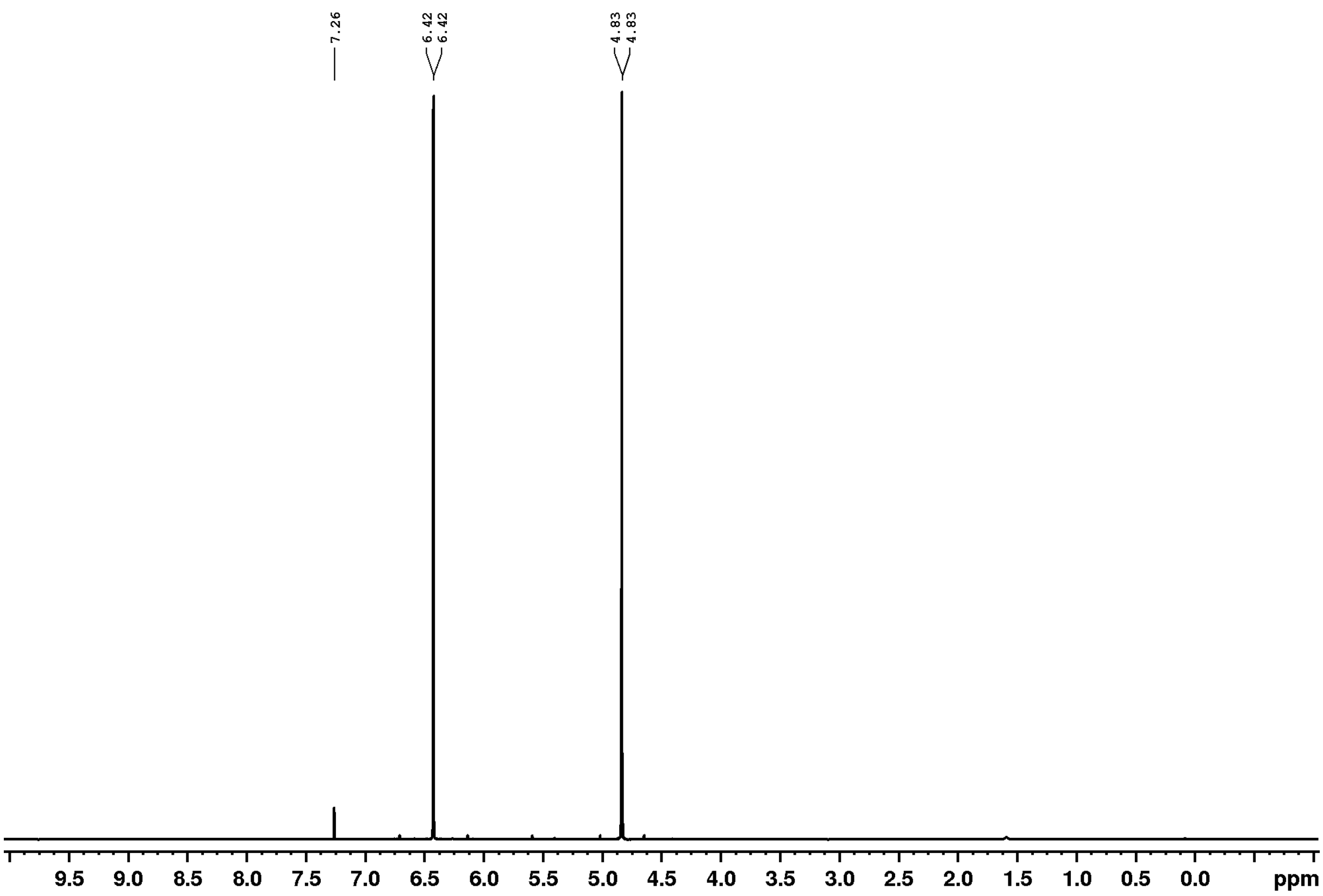

Figure S16. $^1H\{^{19}F\}$ NMR of $(CF_3)_2CHOCHF_2$ (**2**) in $CDCl_3$.

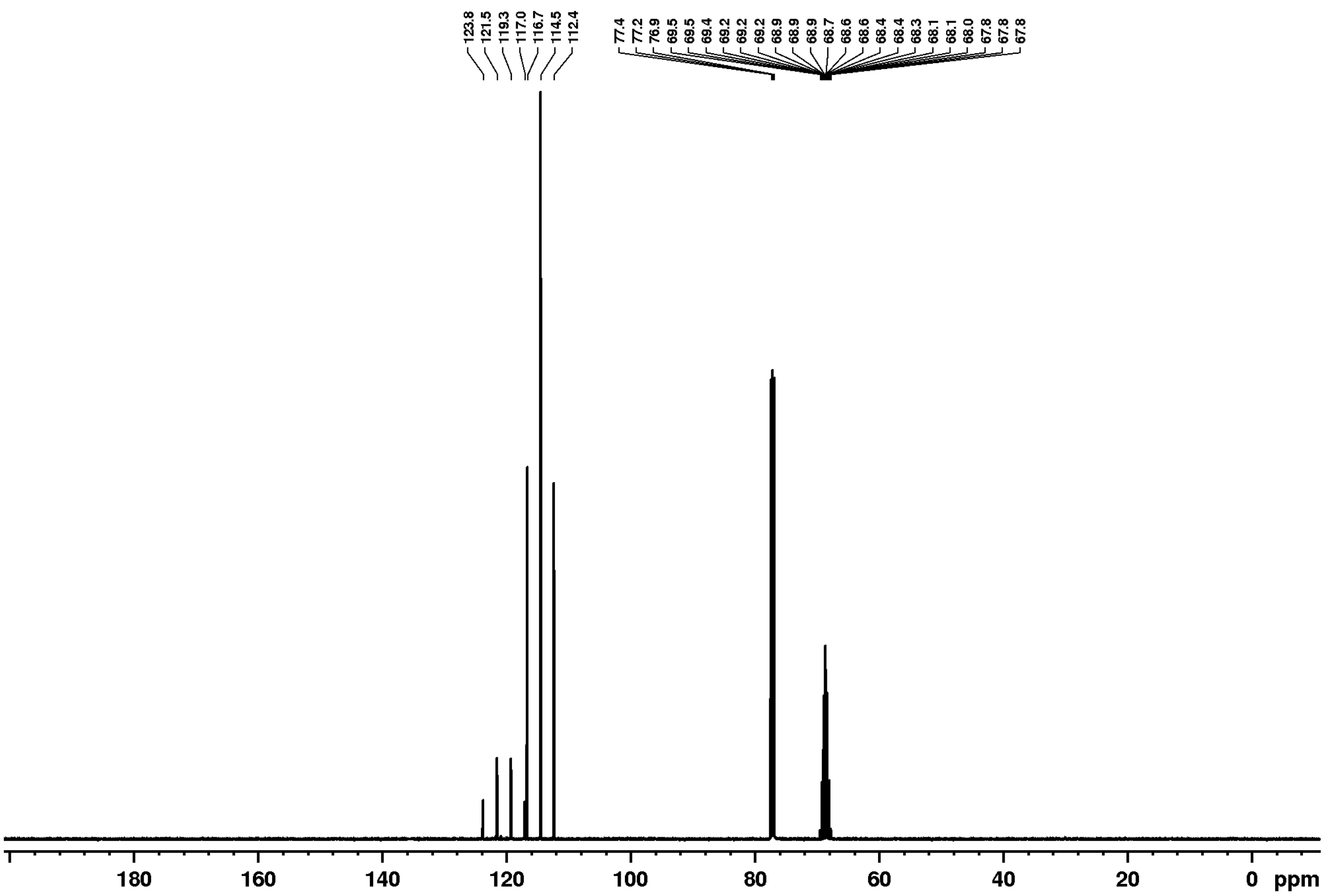

Figure S17. $^{13}C\{^1H\}$ NMR of $(CF_3)_2CHOCHF_2$ (**2**) in $CDCl_3$.

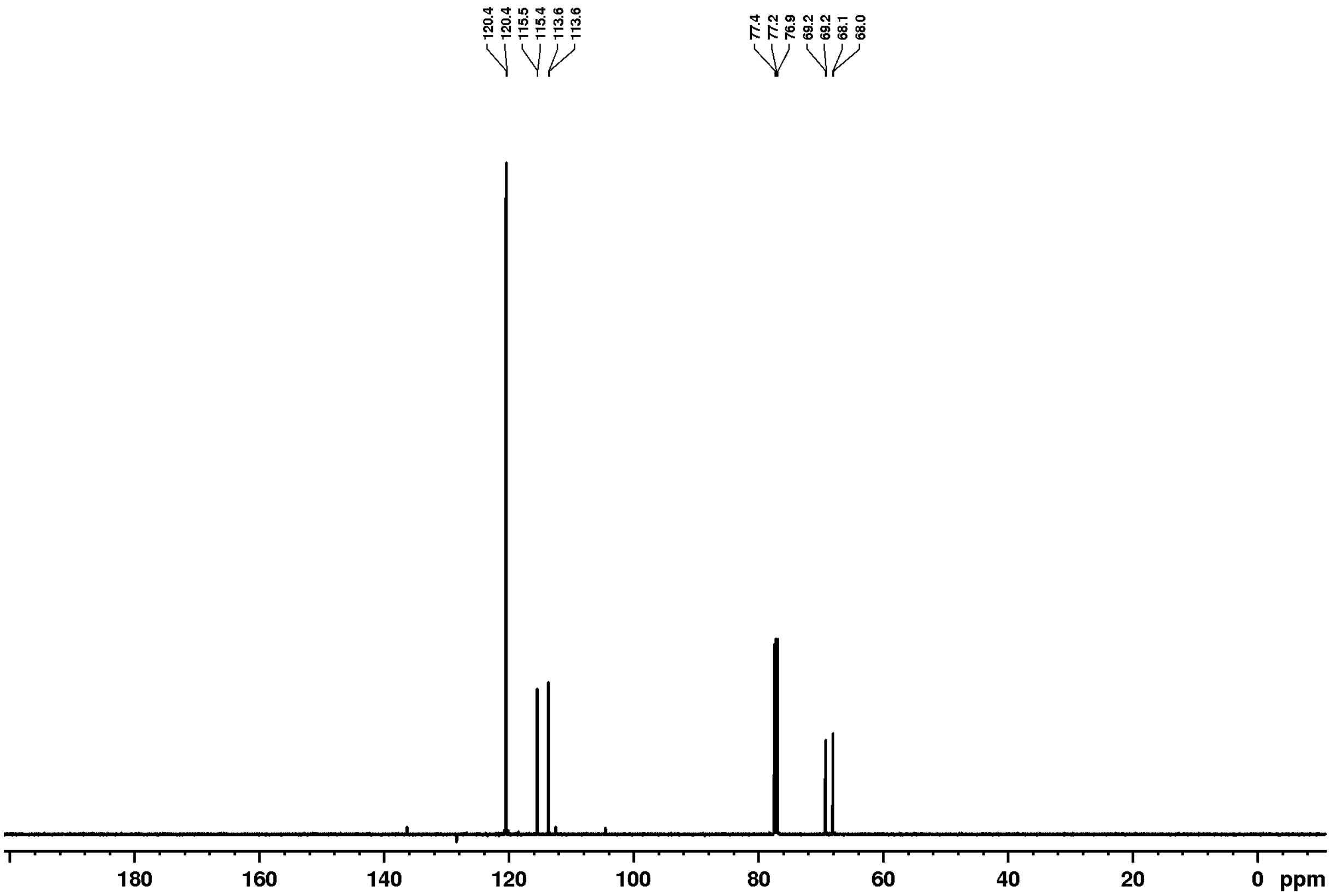

Figure S18. $^{13}C\{^{19}F\}$ NMR of $(CF_3)_2CHOCHF_2$ (**2**) in $CDCl_3$.

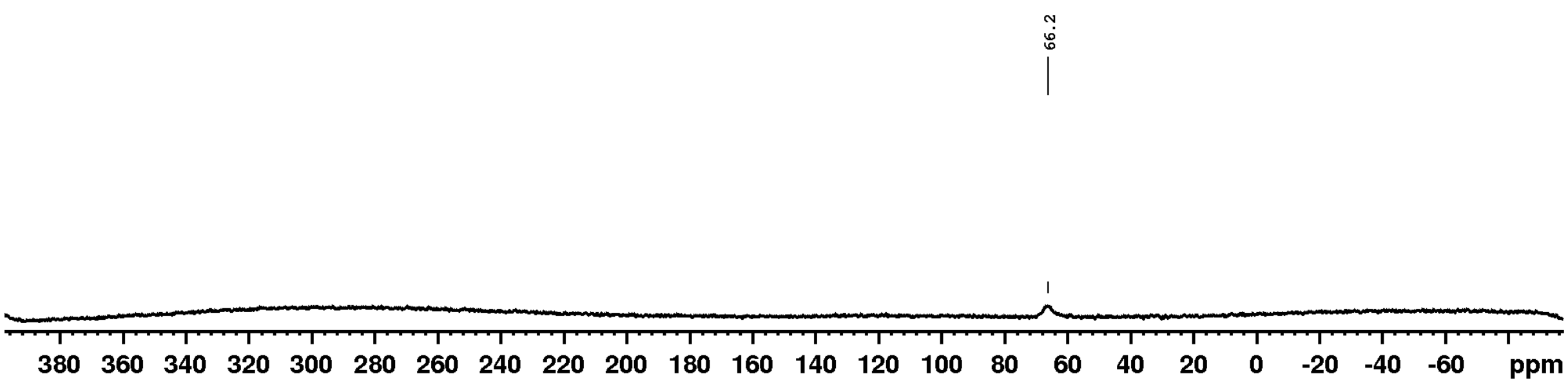

Figure S19. ^{17}O NMR of $(CF_3)_2CHOCHF_2$ (**2**) in $CDCl_3$.

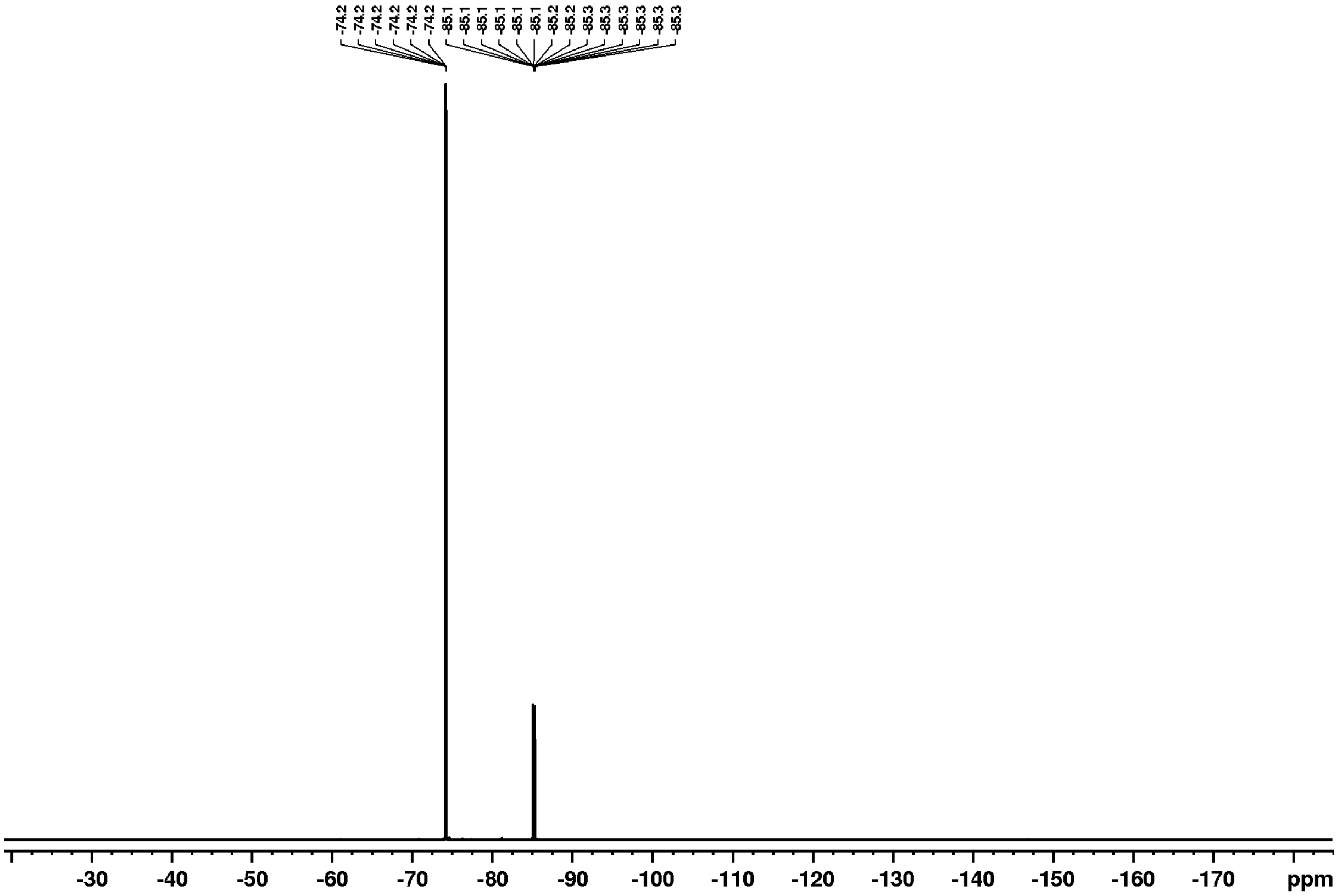

Figure S20. ^{19}F NMR of $(CF_3)_2CHOCHF_2$ (**2**) in $CDCl_3$.

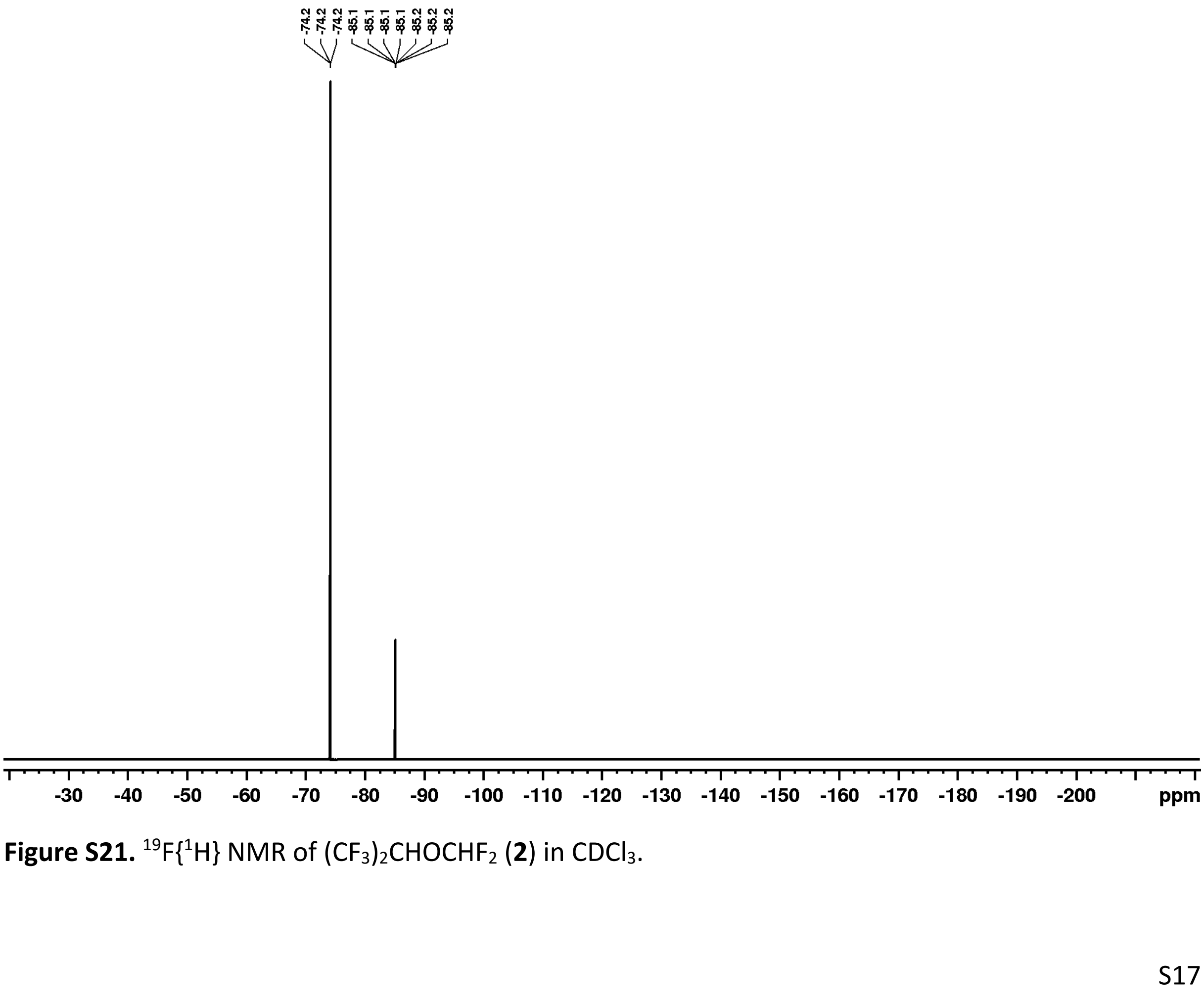

Figure S21. $^{19}F\{^{1}H\}$ NMR of $(CF_3)_2CHOCHF_2$ (**2**) in $CDCl_3$.

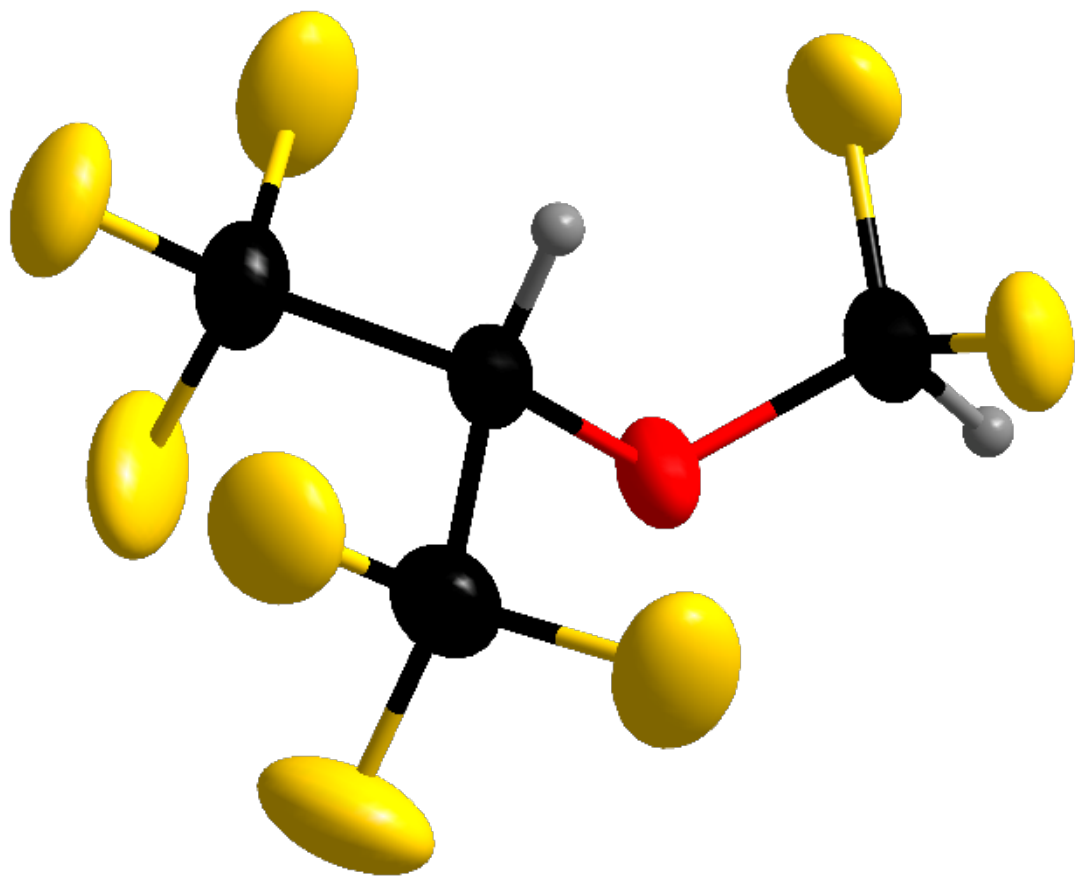

Figure S22. Solid state structure of $(CF_3)_2CHOCHF_2$ (**2**).

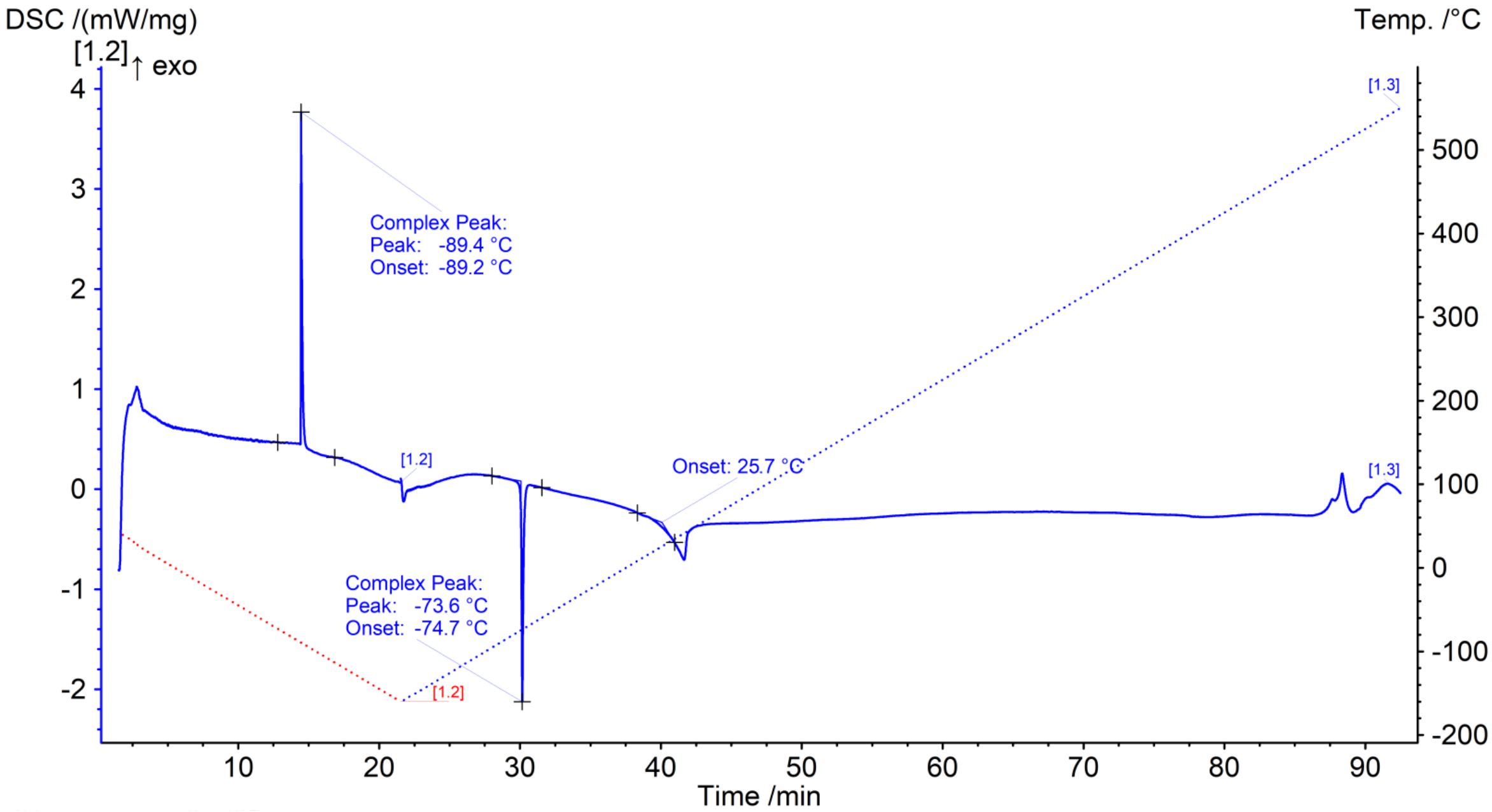

Figure S23. DSC curve of $(CF_3)_2CHOCHF_2$(**2**).

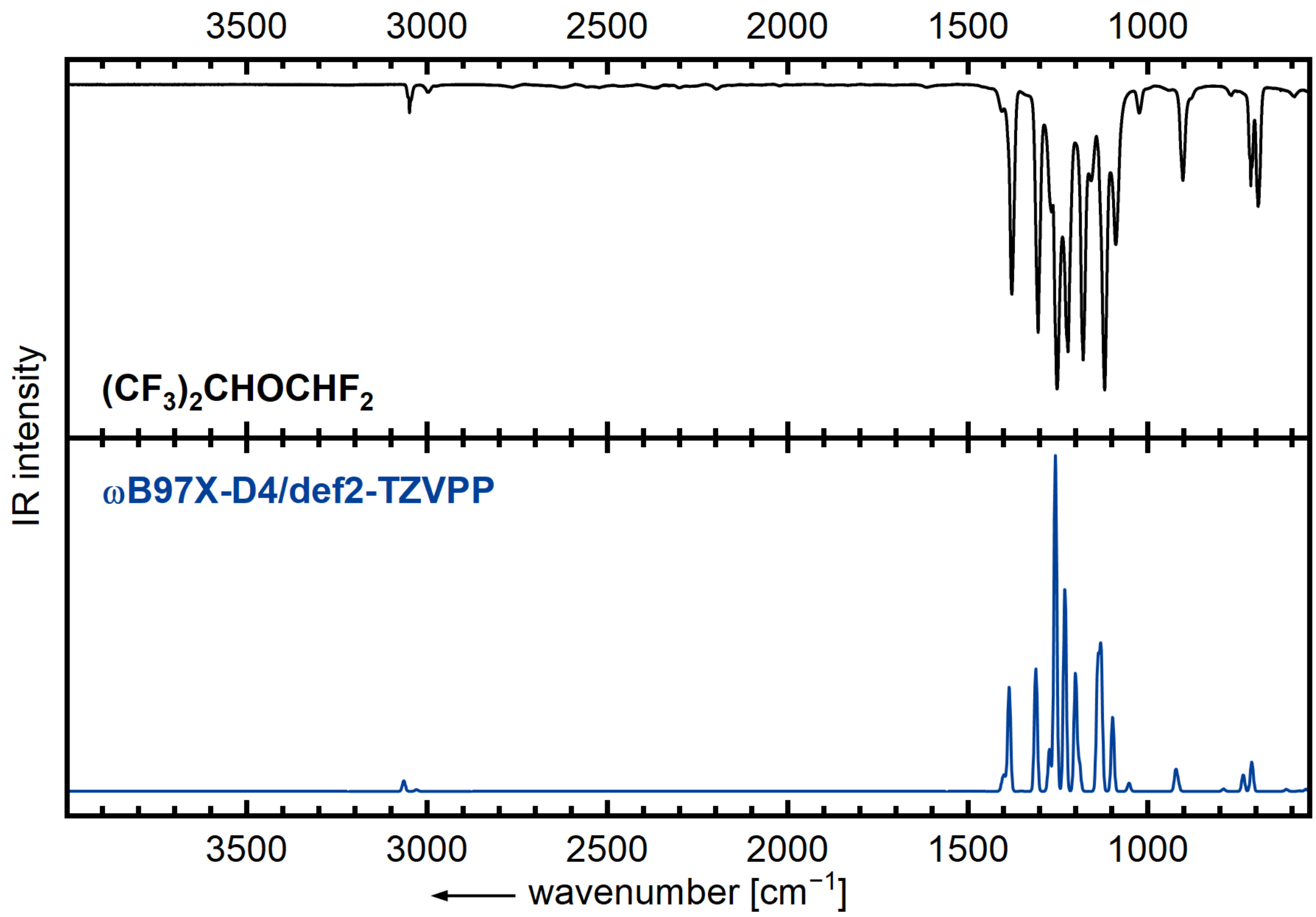

Figure S24. IR spectrum of $(CF_3)_2CHOCF_2H$ (**2**, gas phase): measured (top) vs. calculated (ωB97X-D4/def2-TZVPP, bottom).

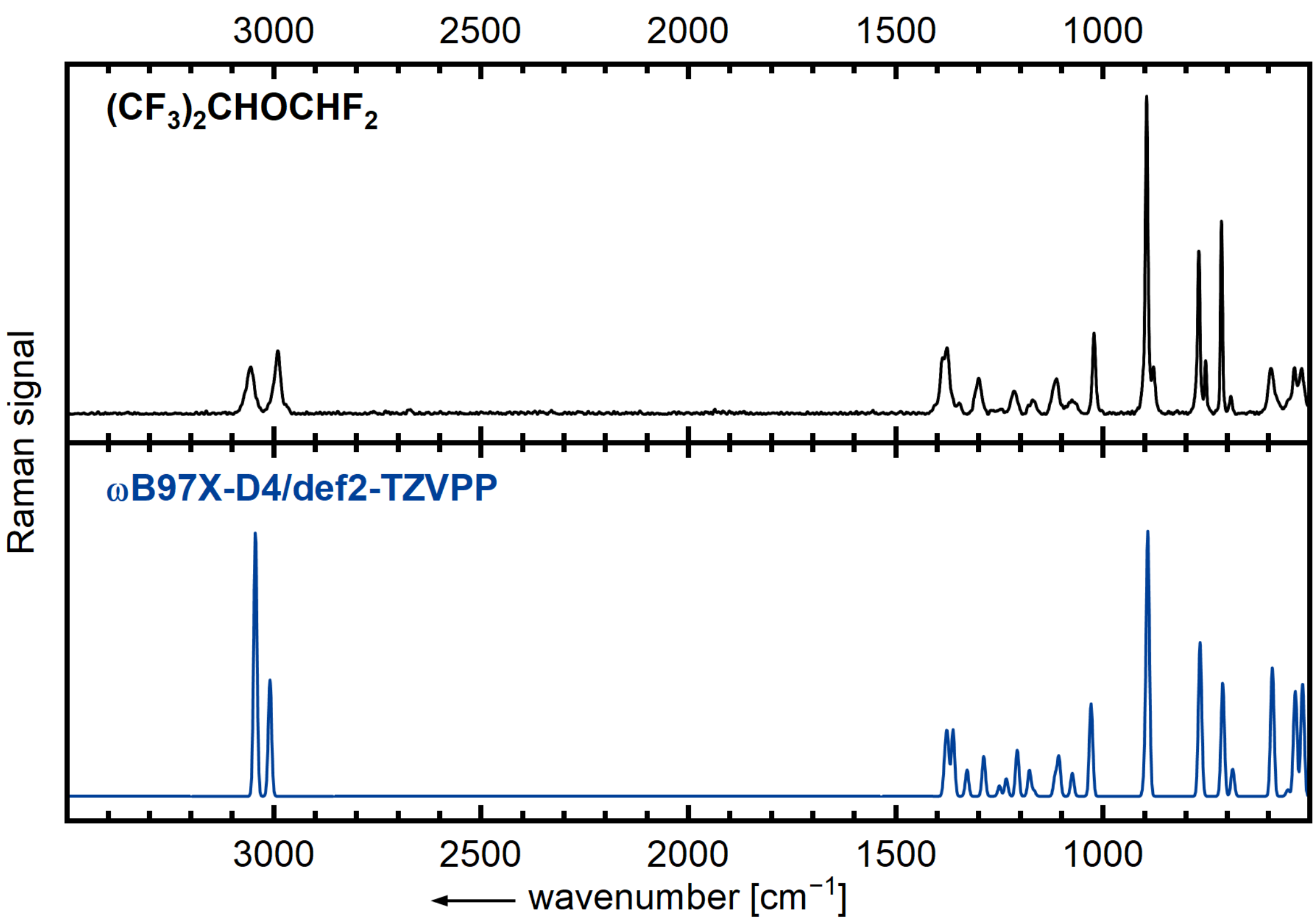

Figure S25. Raman spectrum of $(CF_3)_2CHOCF_2H$ (**2**, liquid phase): measured (top) vs. calculated (ωB97X-D4/def2-TZVPP, bottom).

$(CF_3)_2CHOCF_3$ (3)

Boiling point (distillation): T_{bp} = 12.9 °C at 863 mbar.

Boiling point (vapor pressure): T_{bp} = 16.0 °C at 1013 mbar (Antoine parameter: A = 3.993; B = 932.40; C = −55.284 for equation: $p = 10^{(A-B/(T+C))}$).

Enthalpy of vaporization (T = 298.15 K): $\Delta_{vap}H$ = 26.9 kJ mol^{-1} (for equation: $\Delta_{vap}H = \ln(10)\cdot R\cdot T^2\cdot(B/(T+C)^2)/1000$).

^{1}H NMR (500.1 MHz, $CDCl_3$): δ = 4.78 (septq, 1H, $(CF_3)_2C\mathbf{H}OCF_3$, $^3J(^{19}F, ^1H)$ = 5.4 Hz, $^4J(^{19}F, ^1H)$ = 0.5 Hz) ppm.

^{1}H{^{19}F} NMR (400.3 MHz, $CDCl_3$): δ = 4.77 (s, 1H, $(CF_3)_2C\mathbf{H}OCF_3$) ppm.

^{13}C NMR (125.8 MHz, $CDCl_3$): δ = 121.0 (qd, 1C, $(CF_3)_2CHO\mathbf{C}F_3$, $^1J(^{19}F,^{13}C)$ = 262.4 Hz, $^3J(^{13}C, ^1H)$ = 5.5 Hz), 119.8 (qm, 2C, $(\mathbf{C}F_3)_2CHOCF_3$, $^1J(^{19}F,^{13}C)$ = 281.9 Hz), 71.2 (dseptq, 1C, $(CF_3)_2\mathbf{C}HOCF_3$, $^1J(^{13}C, ^1H)$ = 149.6 Hz, $^2J(^{19}F,^{13}C)$ = 35.8 Hz, $^3J(^{19}F,^{13}C)$ = 3.1 Hz)

^{13}C{^{1}H} NMR (125.8 MHz, $CDCl_3$): δ = 121.0 (q, 1C, $(CF_3)_2CHO\mathbf{C}F_3$, $^1J(^{19}F,^{13}C)$ = 262.4 Hz), 119.8 (qm, 2C, $(\mathbf{C}F_3)_2CHOCF_3$, $^1J(^{19}F,^{13}C)$ = 281.9 Hz), 71.2 (septq, 1C, $(CF_3)_2\mathbf{C}HOCF_3$, $^2J(^{19}F,^{13}C)$ = 35.8 Hz, $^3J(^{19}F,^{13}C)$ = 3.1 Hz) ppm.

^{13}C{^{19}F} NMR (125.8 MHz, $CDCl_3$): δ = 121.1 (d, 1C, $(CF_3)_2CHO\mathbf{C}F_3$, $^3J(^{13}C, ^1H)$ = 5.5 Hz), 119.9 (d, 2C, $(\mathbf{C}F_3)_2CHOCF_3$, $^2J(^{13}C, ^1H)$ = 6.0 Hz), 71.5 (d, 1C, $(CF_3)_2\mathbf{C}HOCF_3$, $^1J(^{13}C, ^1H)$ = 149.9 Hz) ppm.

^{17}O NMR (81.4 MHz, $CDCl_3$): δ = 70.7 (s, 1O, $(CF_3)_2CH\mathbf{O}CF_2H$) ppm.

^{19}F NMR (470.6 MHz, $CDCl_3$): δ = −61.4 (sept, 3F, $(CF_3)_2CHOC\mathbf{F_3}$, $^5J(^{19}F, ^{19}F)$ = 3.0 Hz), −74.5 (m, 6F, $(C\mathbf{F_3})_2CHOCF_3$) ppm.

^{19}F{^{1}H} NMR (376.6 MHz, $CDCl_3$): δ = −60.9 (sept, 3F, $(CF_3)_2CHOC\mathbf{F_3}$, $^5J(^{19}F, ^{19}F)$ = 2.5 Hz), −74.1 (q, 6F, $(C\mathbf{F_3})_2CHOCF_3$, $^4J(^{19}F, ^{19}F)$ = 3.0 Hz) ppm.

GC-MS [t = 0.45 min] m/z$^+$: 236 $[M]^+$, 235 [M −H], 217 [M −F], 167 [M $-CF_3$], 151 [M $-OCF_3$], 69 [M $-(CF_3)_2CHO$].

IR (gas phase, 20 °C, 1.5 mbar); wavenumber [cm^{-1}] (absorbance [%]): $\tilde{\nu}$ = 2999 (2), 1389 (16), 1373 (57), 1313 (67), 1298 (76), 1262 (95), 1225 (91), 1215 (86), 1179 (20), 1121 (55), 906 (25), 876 (25), 694 (36), 691 (35), 682 (27), 678 (31), 676 (20).

Raman (liquid phase, 20 °C); wavenumber [cm^{-1}] (rel. Intensity [%]): $\tilde{\nu}$ = 2992 (7), 1392 (3), 1371 (4), 1312 (4), 1116 (4), 908 (45), 874 (5), 747 (100), 556 (9), 539 (3), 529 (5), 346 (15), 324 (29), 316 (17), 297 (4), 231 (11).

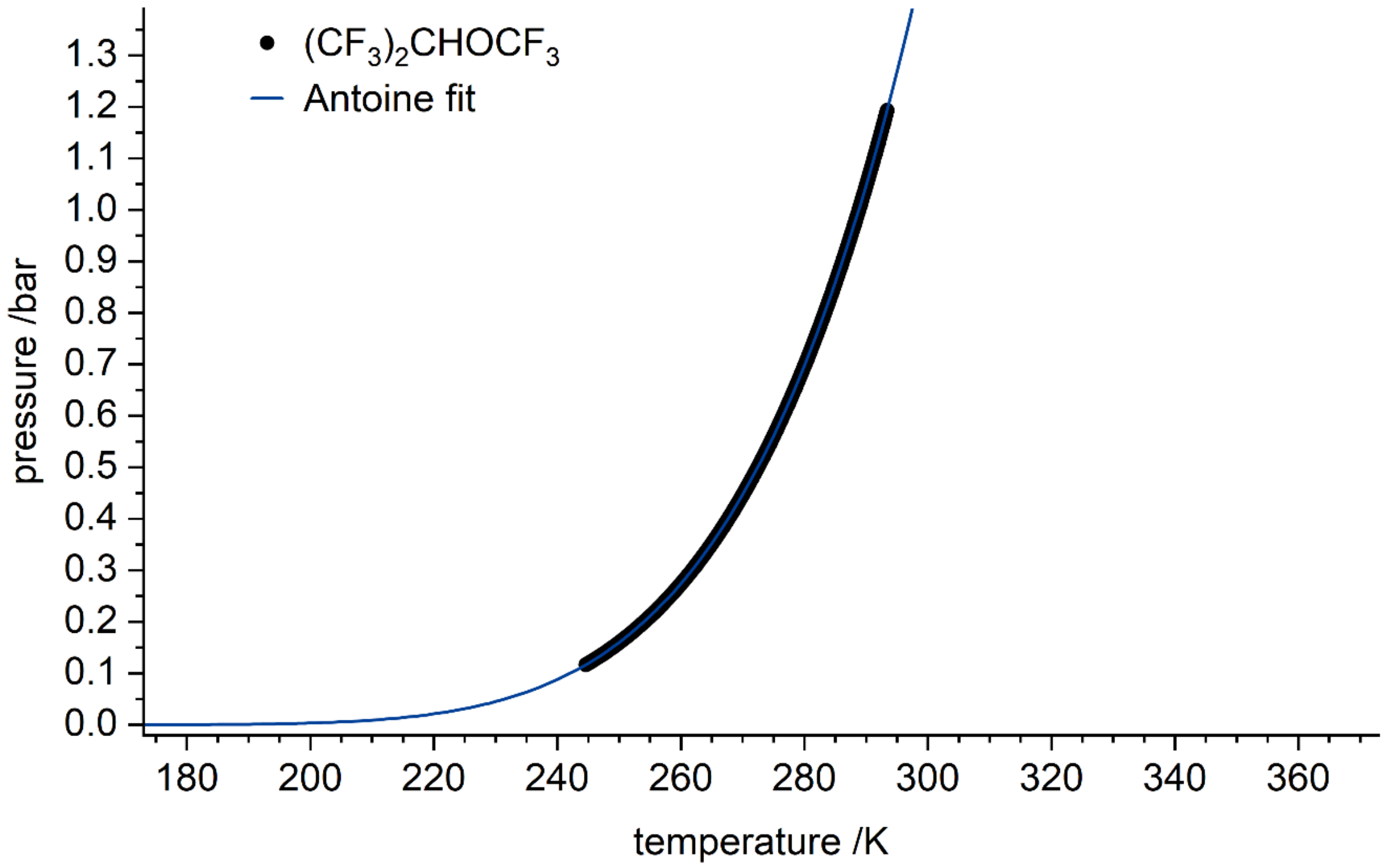

Figure S26. Vapor pressure measurement and Antoine fit of $(CF_3)_2CHOCF_3$ (**3**) (Antoine parameter: A = 3.993; B = 932.40; C = −55.284 for equation: p = 10(A−B/(T+C))).

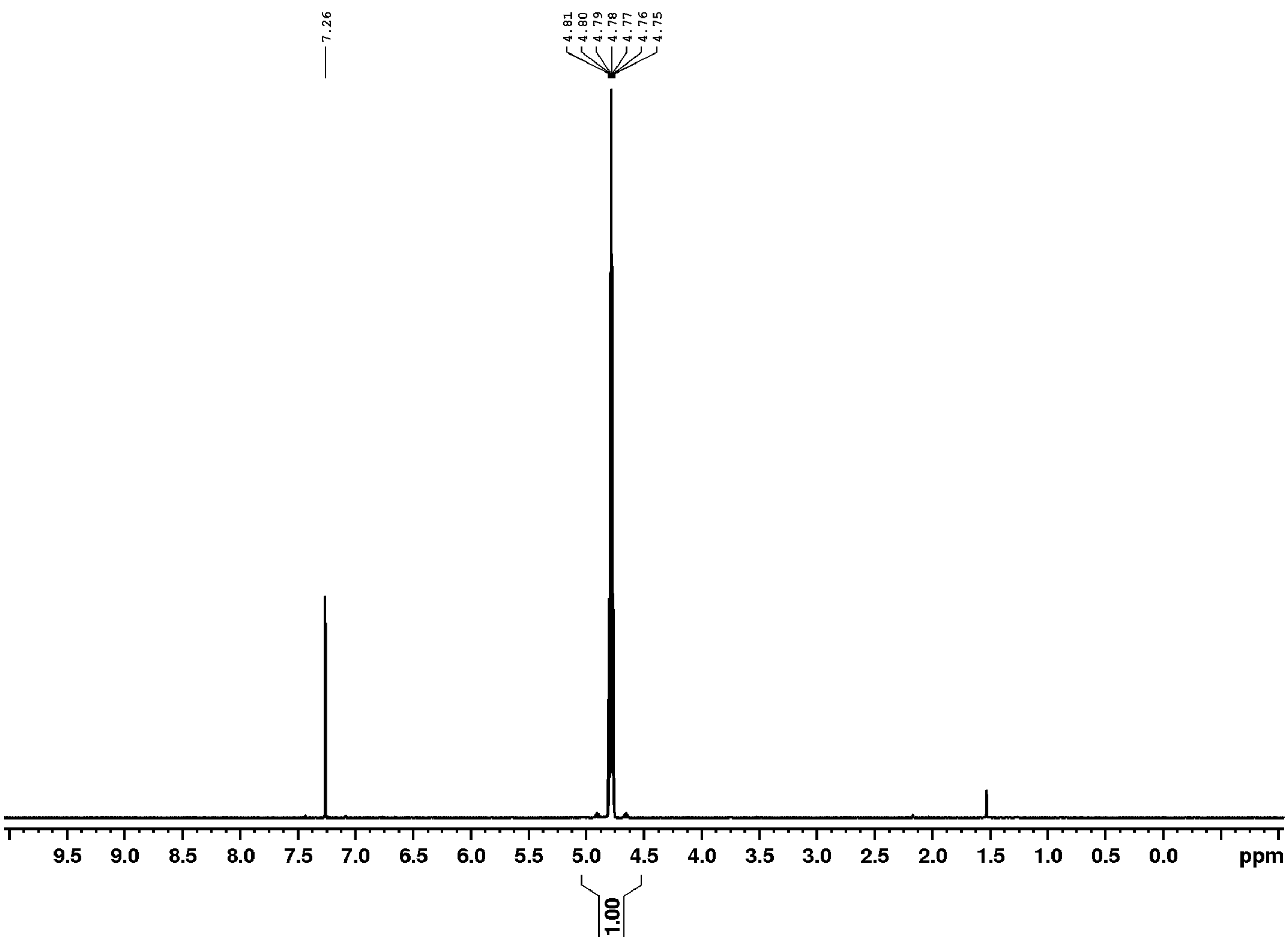

Figure S27. ^{1}H NMR of $(CF_3)_2CHOCF_3$ (**3**) in $CDCl_3$.

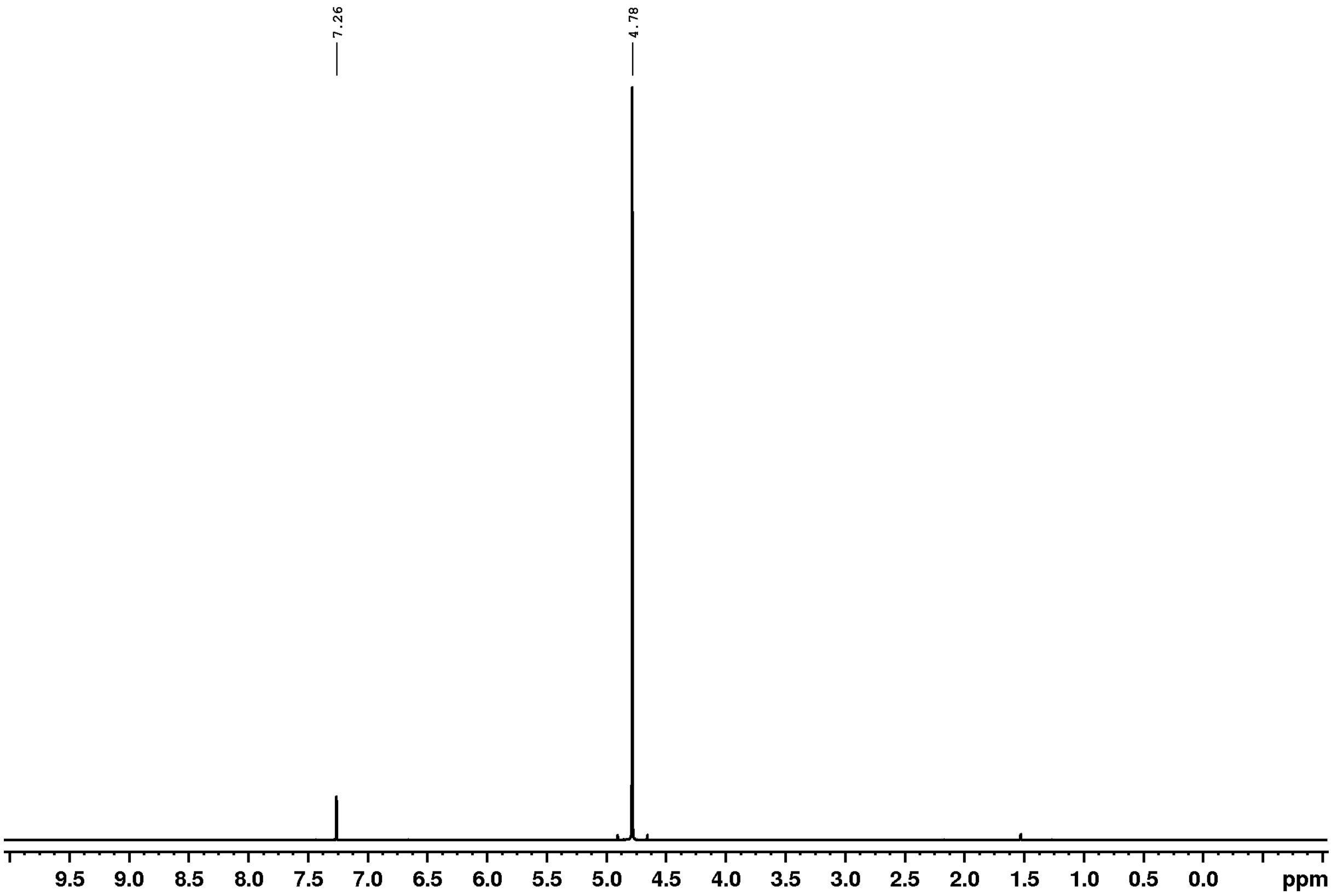

Figure S28. $^{1}H\{^{19}F\}$ NMR of $(CF_3)_2CHOCF_3$ (**3**) in $CDCl_3$.

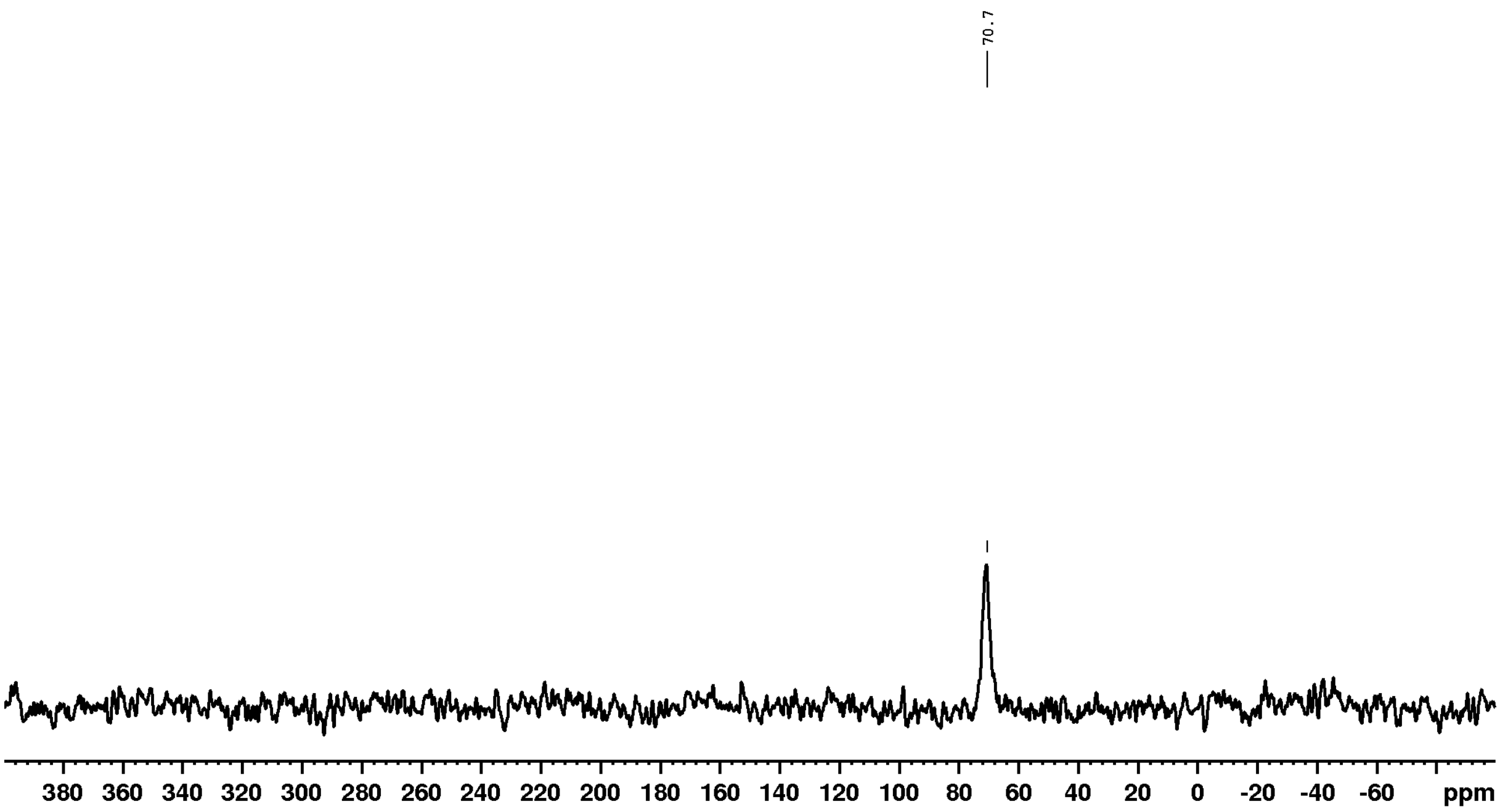

Figure S29. ^{17}O NMR of $(CF_3)_2CHOCF_3$ (**3**) in $CDCl_3$.

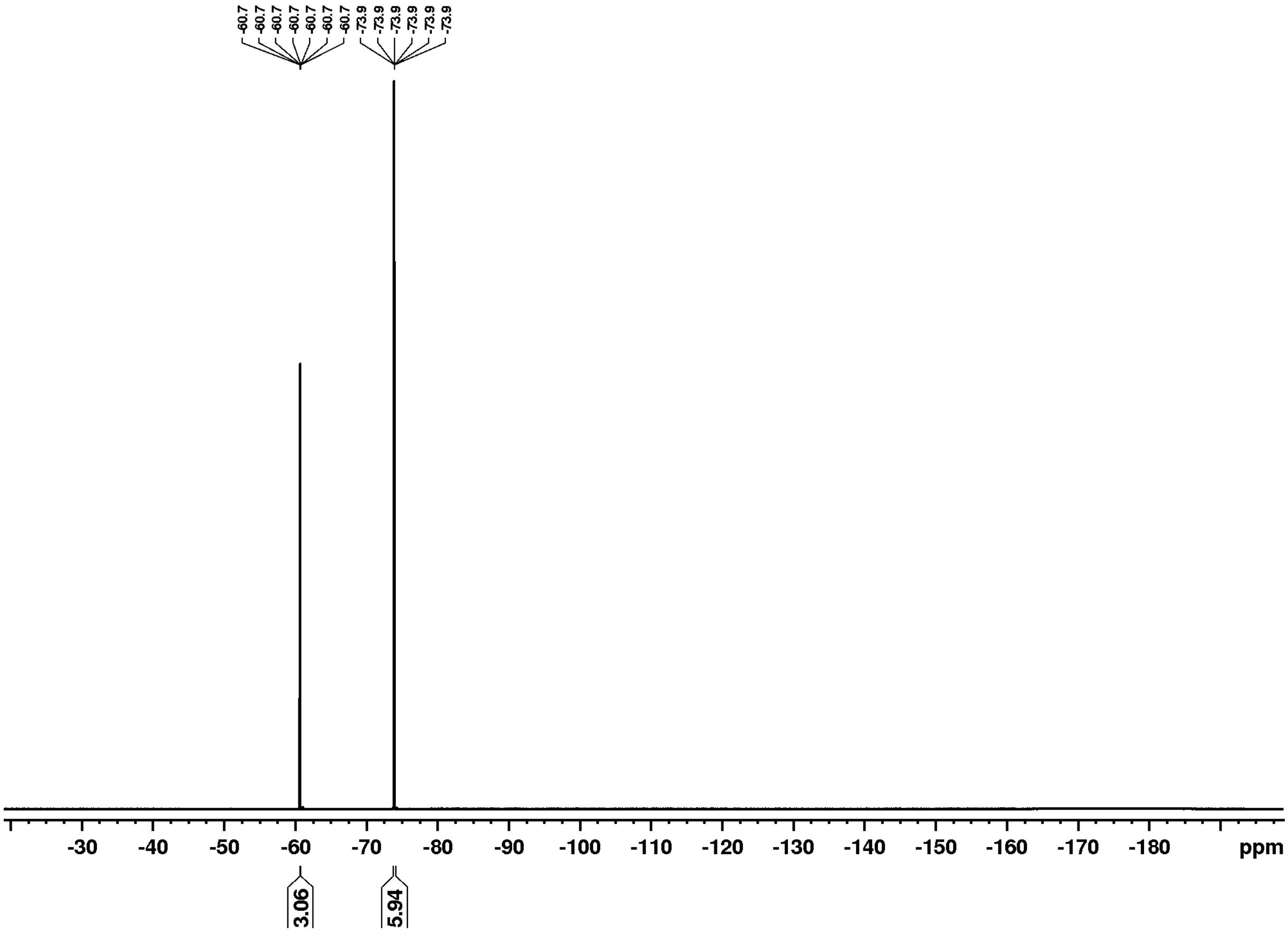

Figure S30. ^{19}F NMR of $(CF_3)_2CHOCF_3$ (**3**) in $CDCl_3$.

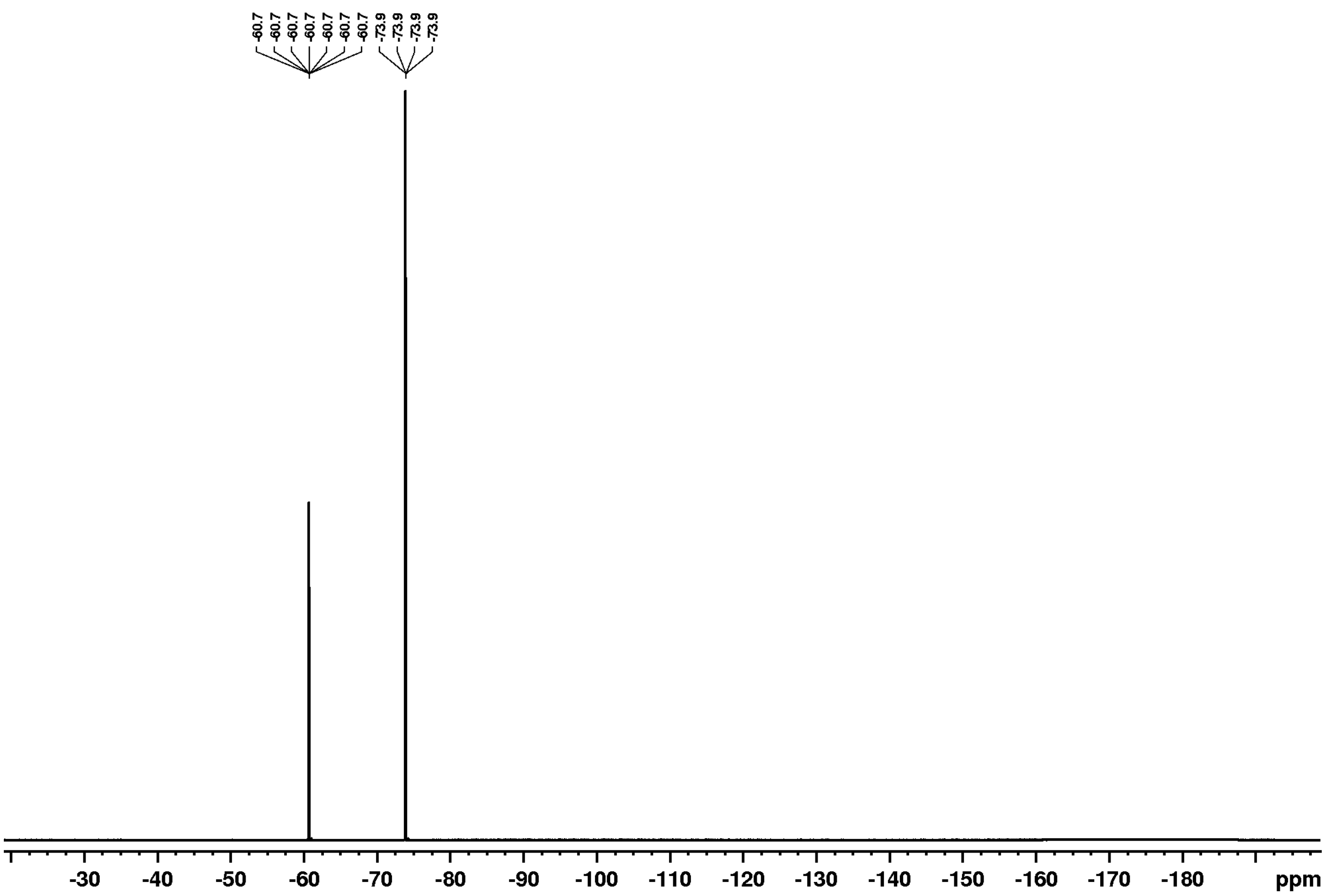

Figure S31. $^{19}F\{^1H\}$ NMR of $(CF_3)_2CHOCF_3$ (**3**) in $CDCl_3$.

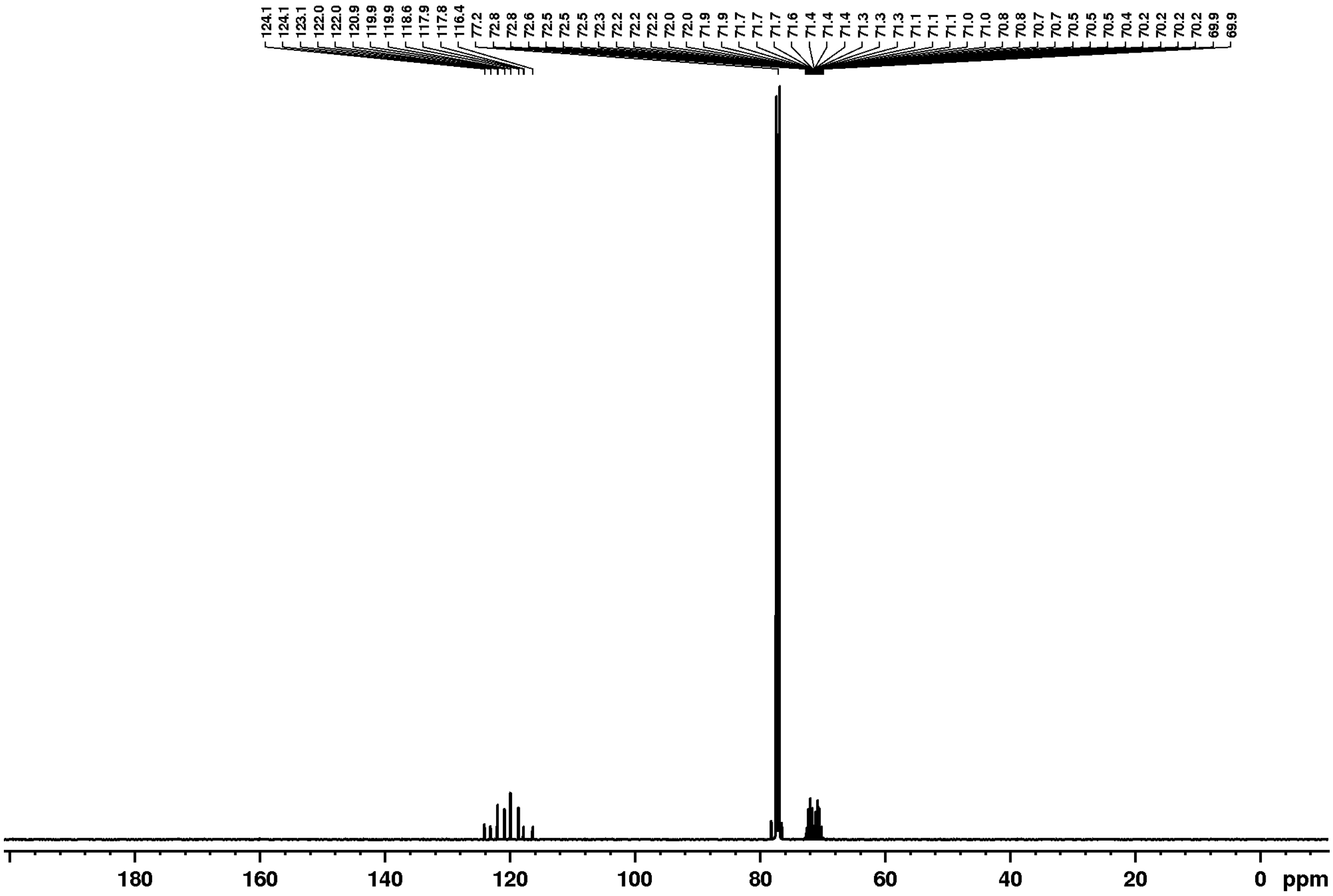

Figure S32. ^{13}C NMR of $(CF_3)_2CHOCF_3$ (**3**) in $CDCl_3$.

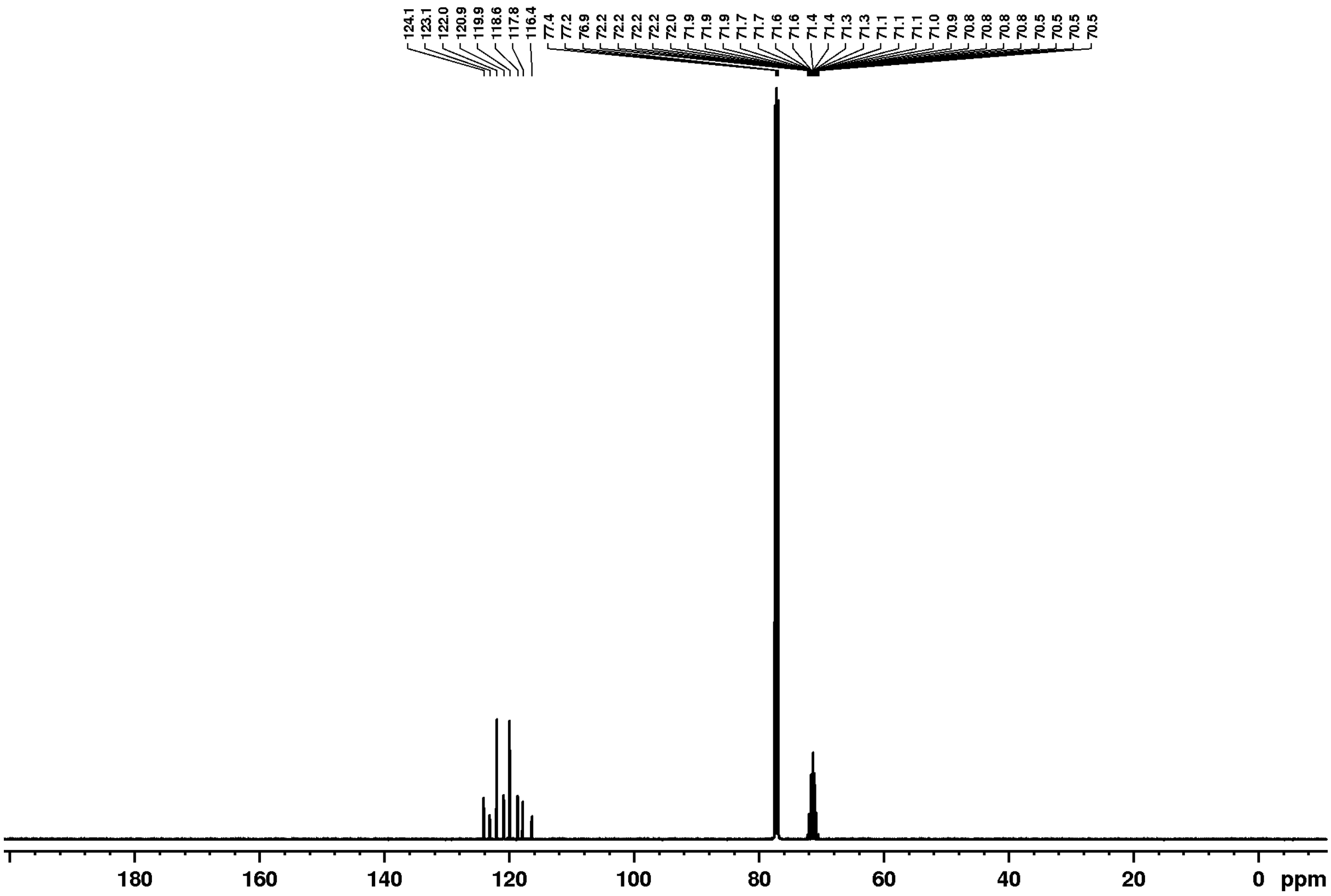

Figure S33. $^{13}C\{^1H\}$ NMR of $(CF_3)_2CHOCF_3$ (**3**) in $CDCl_3$.

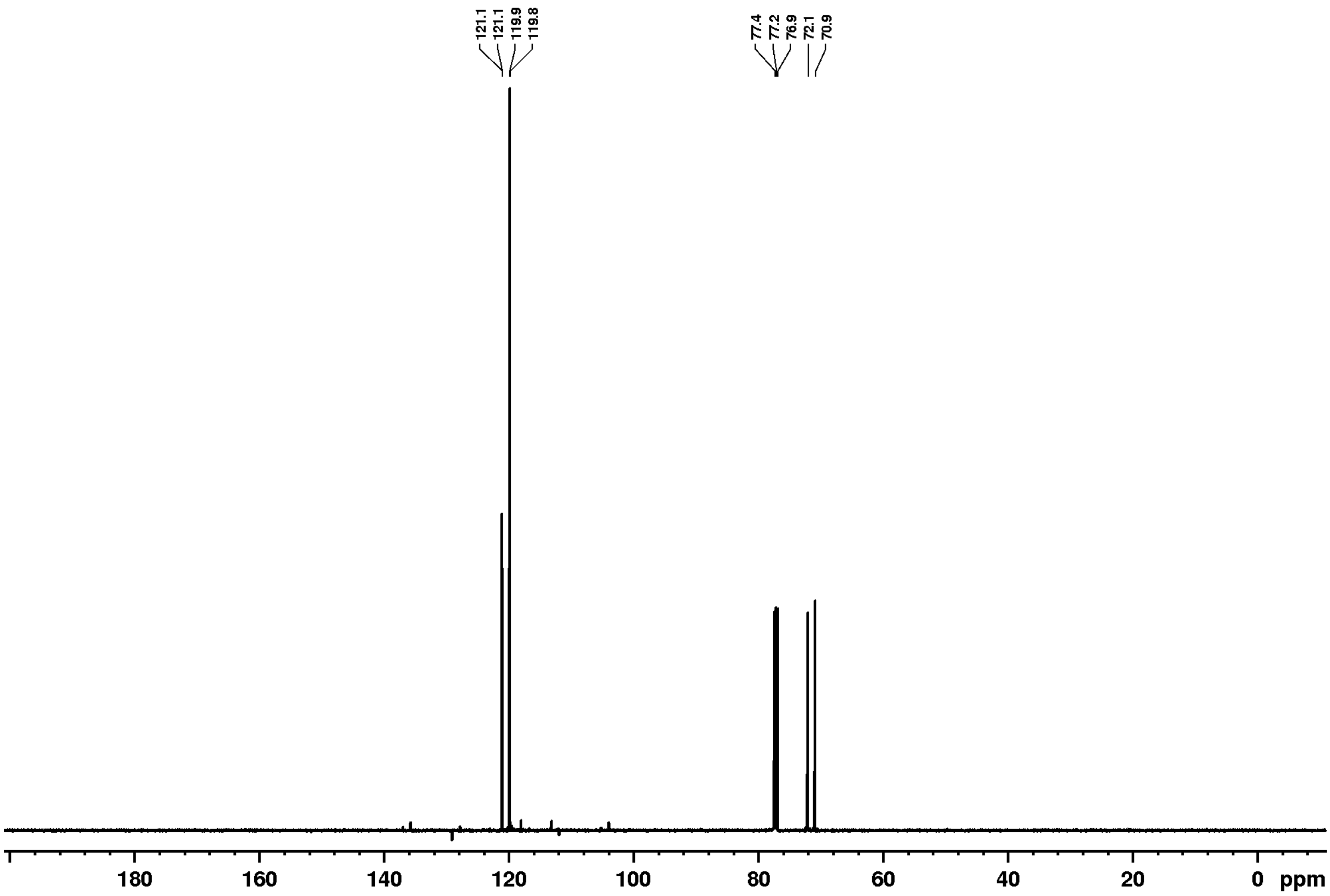

Figure S34. $^{13}C\{^{19}F\}$ NMR of $(CF_3)_2CHOCF_3$ (**3**) in $CDCl_3$.

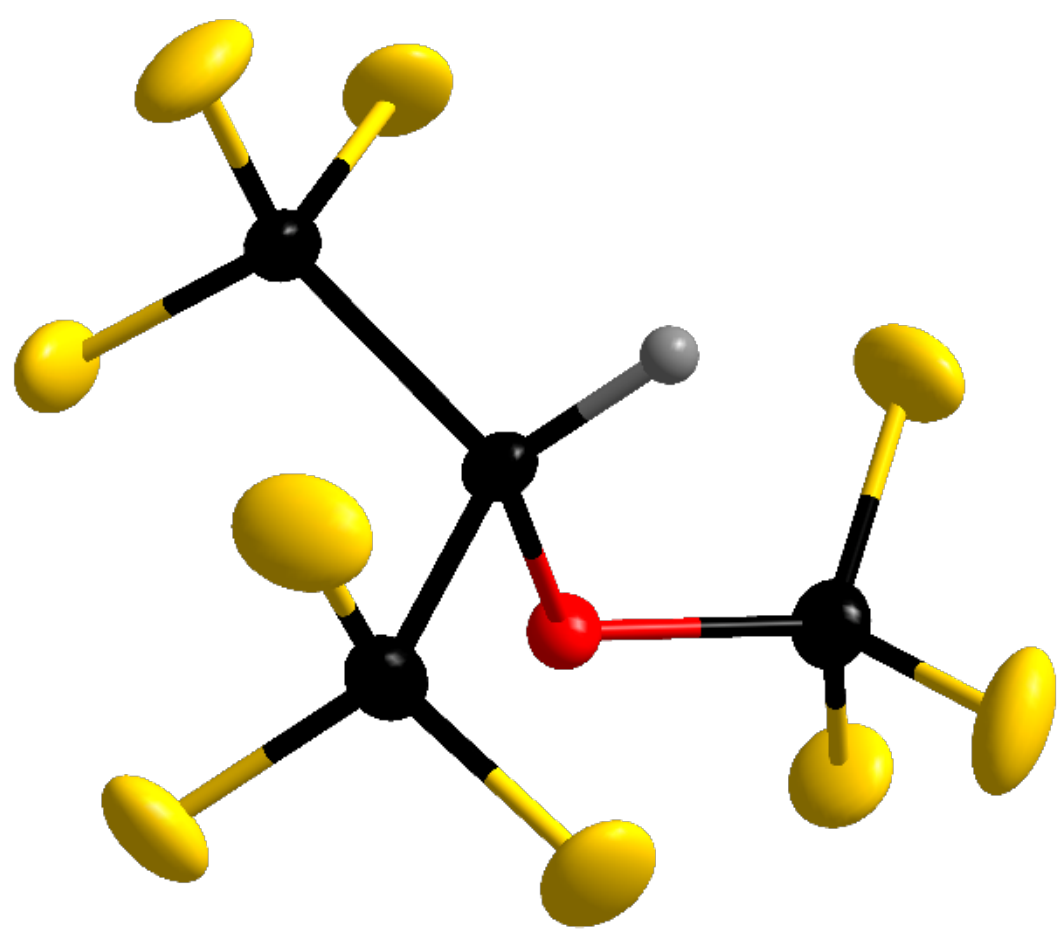

Figure S35. Solid state structure of $(CF_3)_2CHOCF_3$ (**3**).

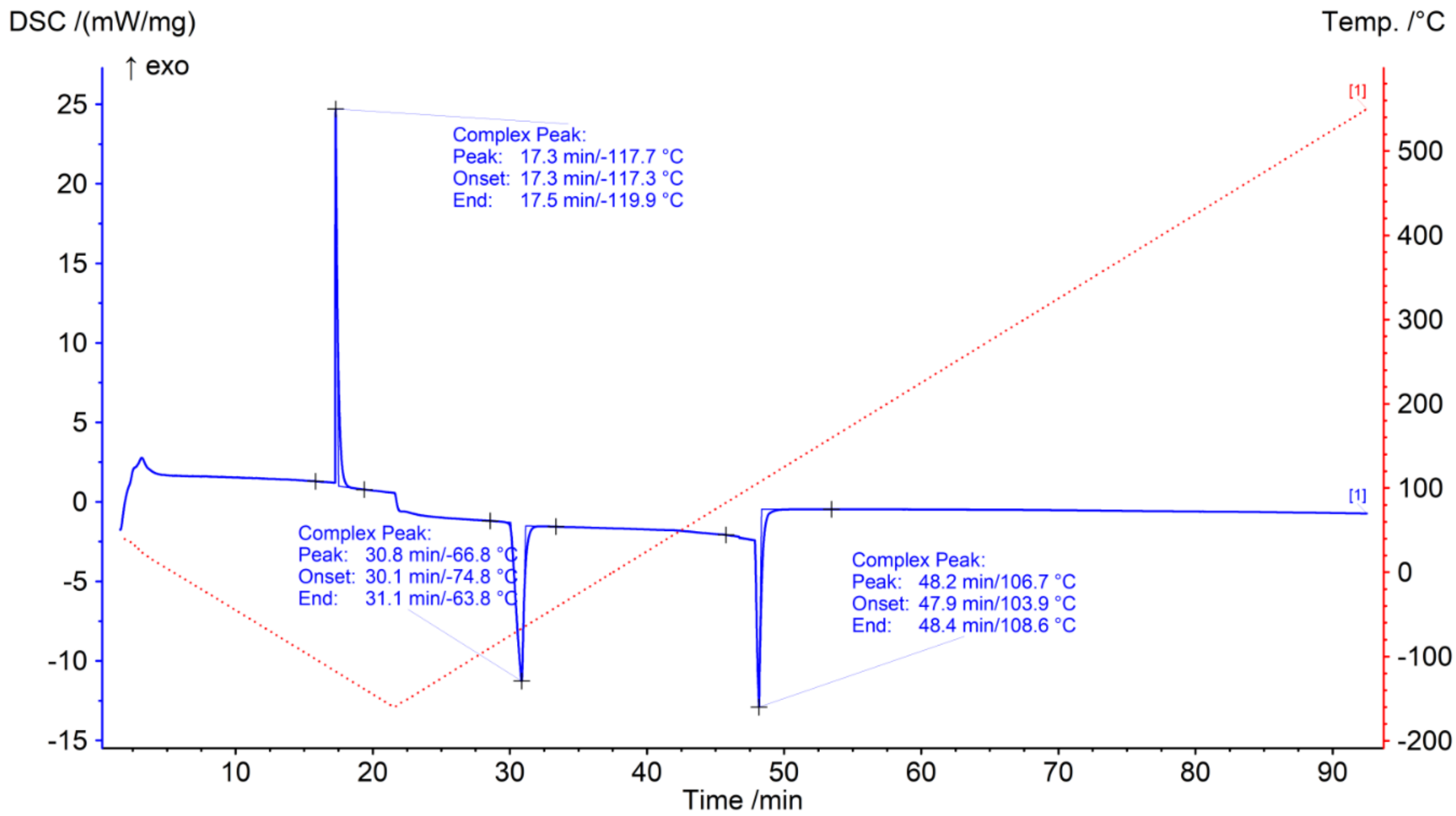

Figure S36. DSC curve of $(CF_3)_2CHOCF_3$ (**3**).

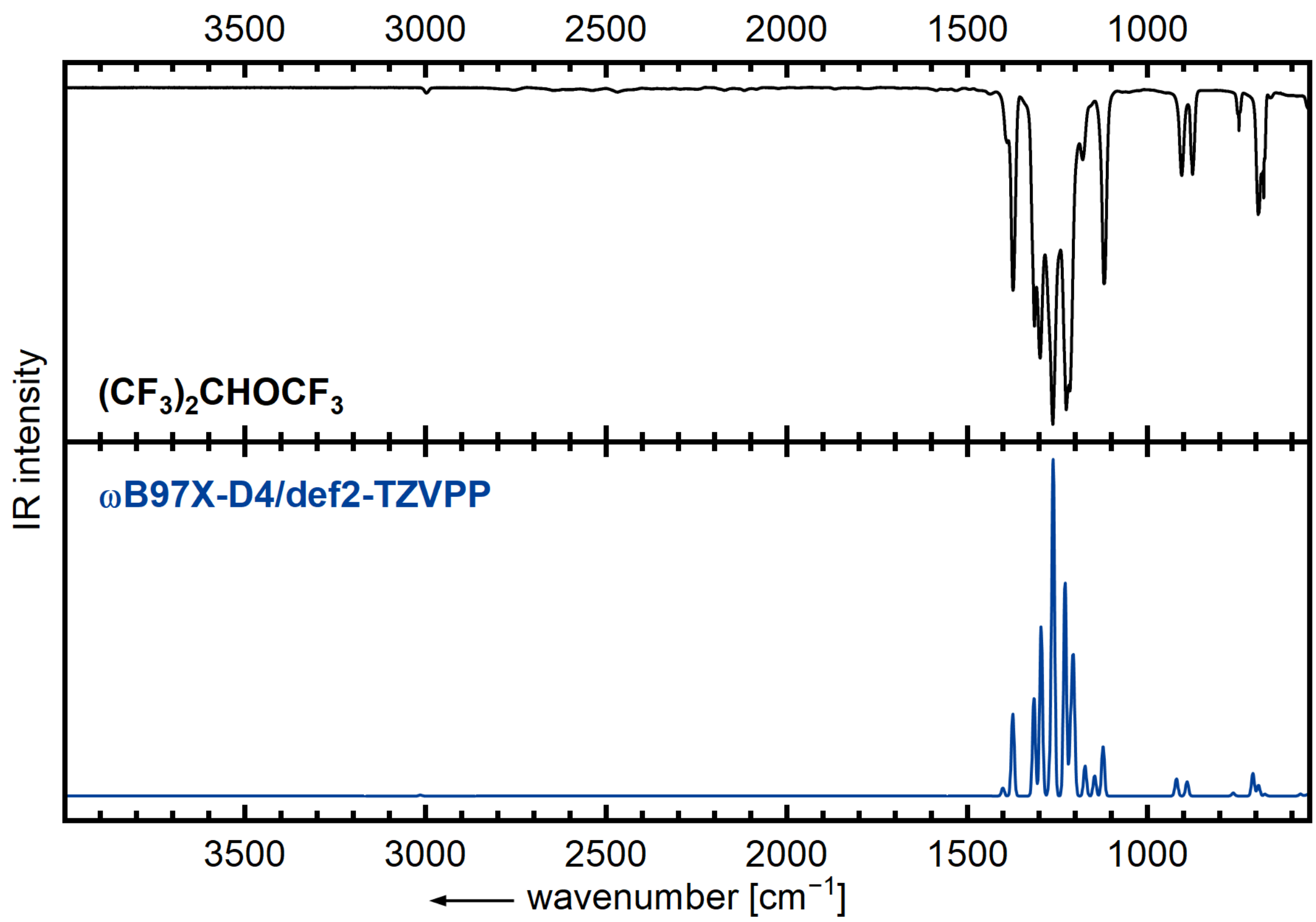

Figure S37. IR spectrum of $(CF_3)_2CHOCF_3$ (**3**, gas phase): measured (top) vs. calculated (ωB97X-D4/def2-TZVPP, bottom).

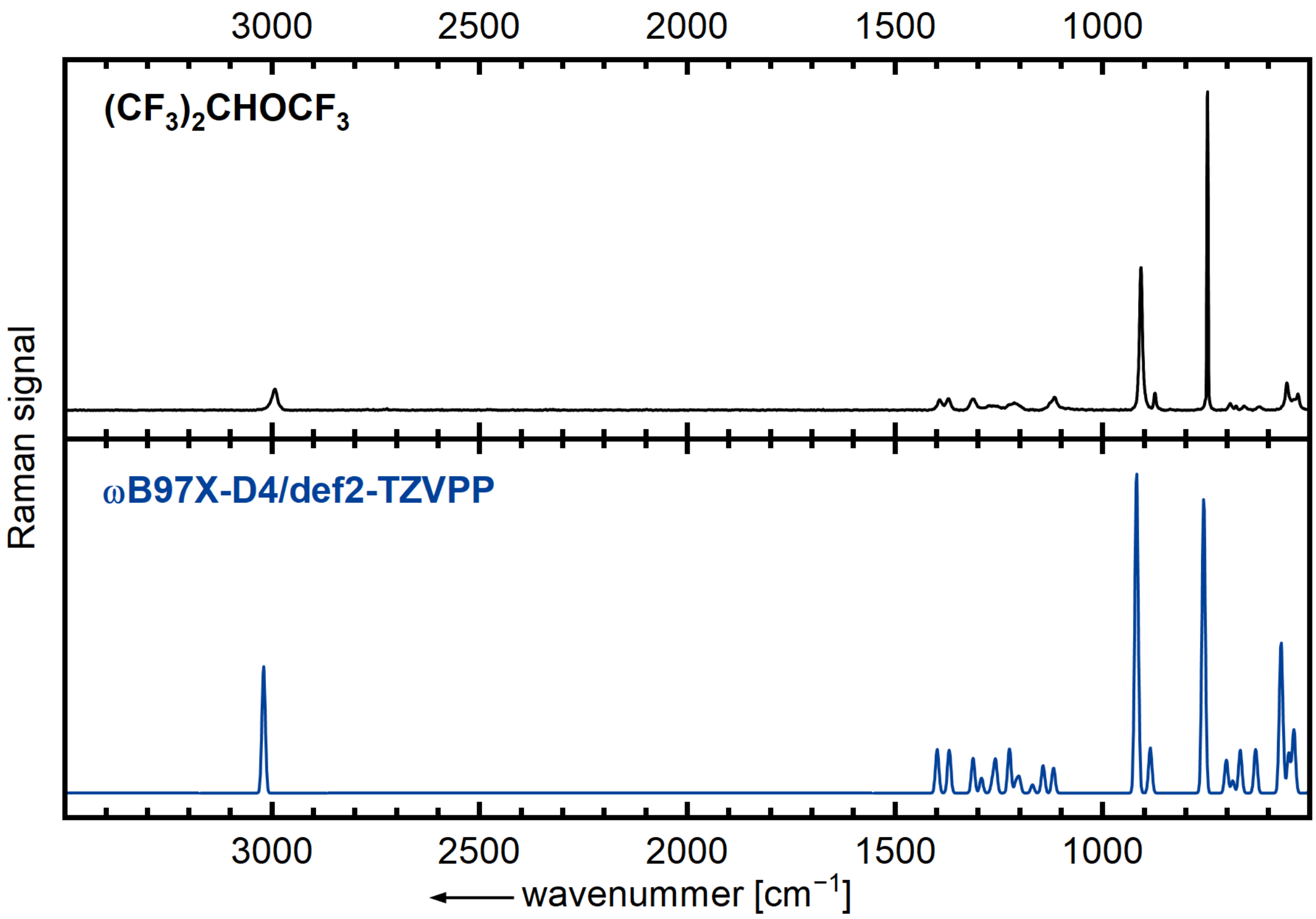

Figure S38. Raman spectrum of $(CF_3)_2CHOCF_3$ (**3**, liquid phase): measured (top) vs. calculated (ωB97X-D4/def2-TZVPP, bottom).

$(CF_3)_2CFOCF_3$ (4)

Boiling point (distillation): T_{bp} = 4.0 °C at 858 mbar.

Boiling point (vapor pressure): T_{bp} = 7.0 °C at 1013 mbar (Antoine parameter: A = 3.922; B = 885.51; C = −53.983 for equation: $p = 10^{(A-B/(T+C))}$).

Enthalpy of vaporization (T = 298.15 K): $\Delta_{vap}H$ = 25.3 kJ mol^{-1} (for equation: $\Delta_{vap}H = \ln(10)\cdot R\cdot T^2\cdot(B/(T+C)^2)/1000$).

$^{13}C\{^1H\}$ NMR (470.5 MHz, $CDCl_3$): δ = 119.4 (qd, 1C, $(CF_3)_2CFO\mathbf{C}F_3$, $^1J(^{13}C, ^{19}F)$ = 267.7 Hz, $^3J(^{13}C, ^{19}F)$ = 1.7 Hz), 117.6 (qd, 2C, $(\mathbf{C}F_3)_2CFOCF_3$, $^1J(^{19}F,^{13}C)$ = 288.3 Hz, $^2J(^{19}F,^{13}C)$ = 31.3 Hz), 100.7 (dsept, 1C, $(CF_3)_2\mathbf{C}FOCF_3$, $^1J(^{13}C, ^{19}F)$ = 267.3 Hz, $^2J(^{19}F,^{13}C)$ = 38.7 Hz) ppm.

$^{13}C\{^{19}F\}$ NMR (470.5 MHz, $CDCl_3$): δ = 119.4 (s, 1C, $(CF_3)_2CFO\mathbf{C}F_3$), 117.6 (s, 2C, $(\mathbf{C}F_3)_2CFOCF_3$), 100.7 (s, 1C, $(CF_3)_2\mathbf{C}FOCF_3$) ppm.

^{17}O NMR (54.3 MHz, $CDCl_3$): δ = 99.7 (s, 1O, $\mathbf{O}CF_3$) ppm.

^{19}F NMR (564.7 MHz, $CDCl_3$): δ = −53.6 (dsept, 3F, $(CF_3)_2CFOC\mathbf{F_3}$, $^4J(^{19}F, ^{19}F)$ = 14.7Hz, $^5J(^{19}F, ^{19}F)$ = 3.6 Hz), −80.4 (dq, 6F, $(C\mathbf{F_3})_2CFOCF_3$, $^3J(^{19}F, ^{19}F)$ = 3.6 Hz, $^5J(^{19}F, ^{19}F)$ = 2.4 Hz), −146.3 (qsept, 1F, $(CF_3)_2C\mathbf{F}OCF_3$, $^4J(^{19}F, ^{19}F)$ = 14.6 Hz, $^3J(^{19}F, ^{19}F)$ = 2.4 Hz) ppm.

GC-MS [t = 45 min] m/z$^+$: 235 $[M]^+$, 169 [M −OCF_3], 119 [M −CF_2OCF_3], 100 [M −CF_3OCF_3], 69 [M −$(CF_3)_2CFO$].

IR (gas phase, 20 °C, 1.5 mbar); wavenumber [cm^{-1}] (absorbance [%]): $\tilde{\nu}$ = 1368 (6), 541 (10), 749 (13), 1320 (49), 1308 (38), 1285 (89), 1272 (95), 1251 (48), 1241 (66), 1220 (60), 1167 (89), 1137 (28), 997 (57), 895 (20), 729 (26), 727 (26), 686 (16).

Raman (liquid phase, 20 °C); wavenumber [cm^{-1}] (rel. Intensity [%]): $\tilde{\nu}$ = 1363 (5), 1323 (6), 1294 (4), 1254 (5), 1159 (2), 1138 (3), 895 (18), 791 (100), 749 (20), 685 (3), 617 (10), 557 (12), 541 (9), 458 (3), 347 (36), 307 (59), 219 (15).

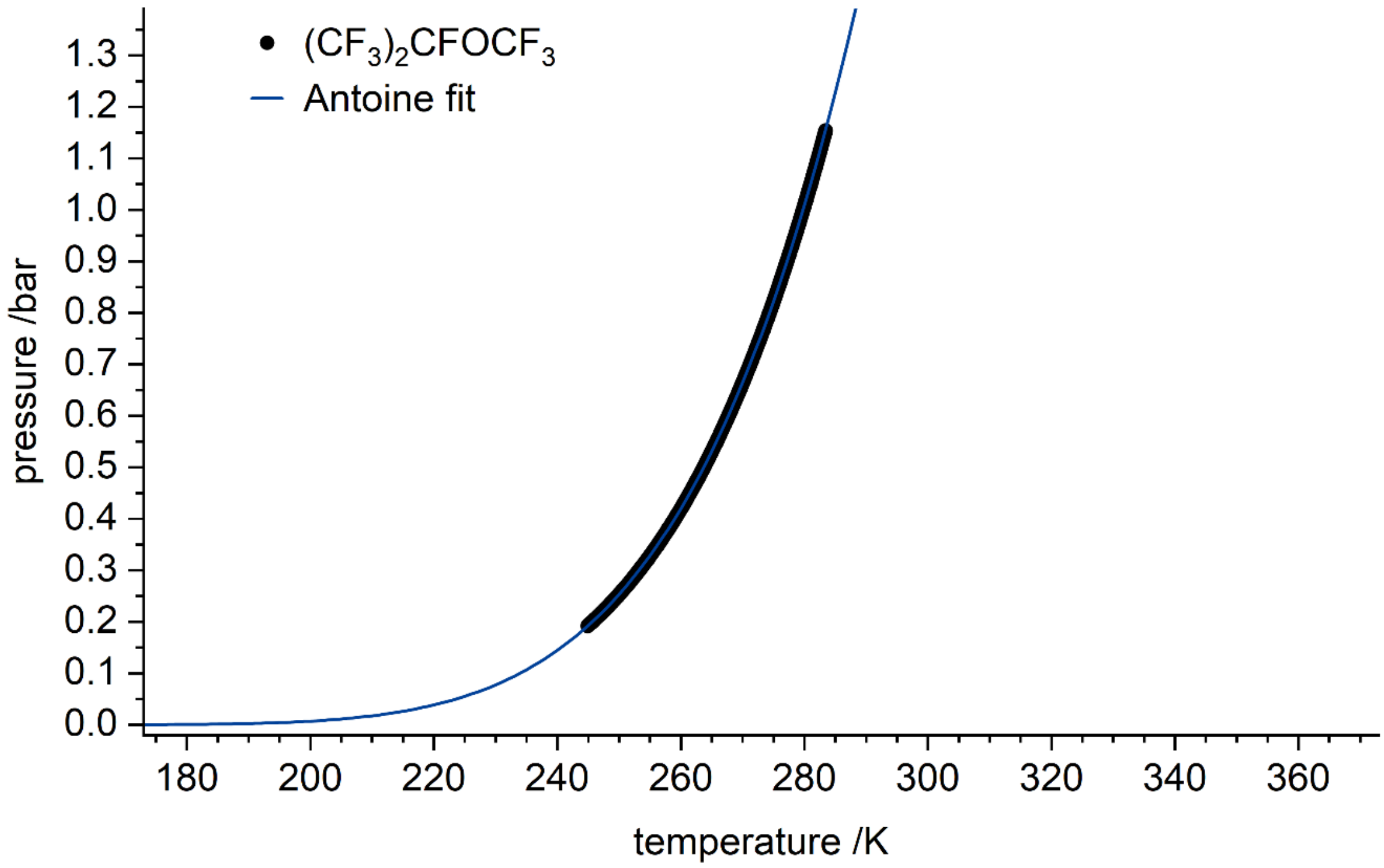

Figure S39. Vapor pressure measurement and Antoine fit of $(CF_3)_2CHOCF_3$ (**4**) (Antoine parameter: A = 3.922; B = 885.51; C = −53.983 for equation: $p = 10^{(A-B/(T+C))}$).

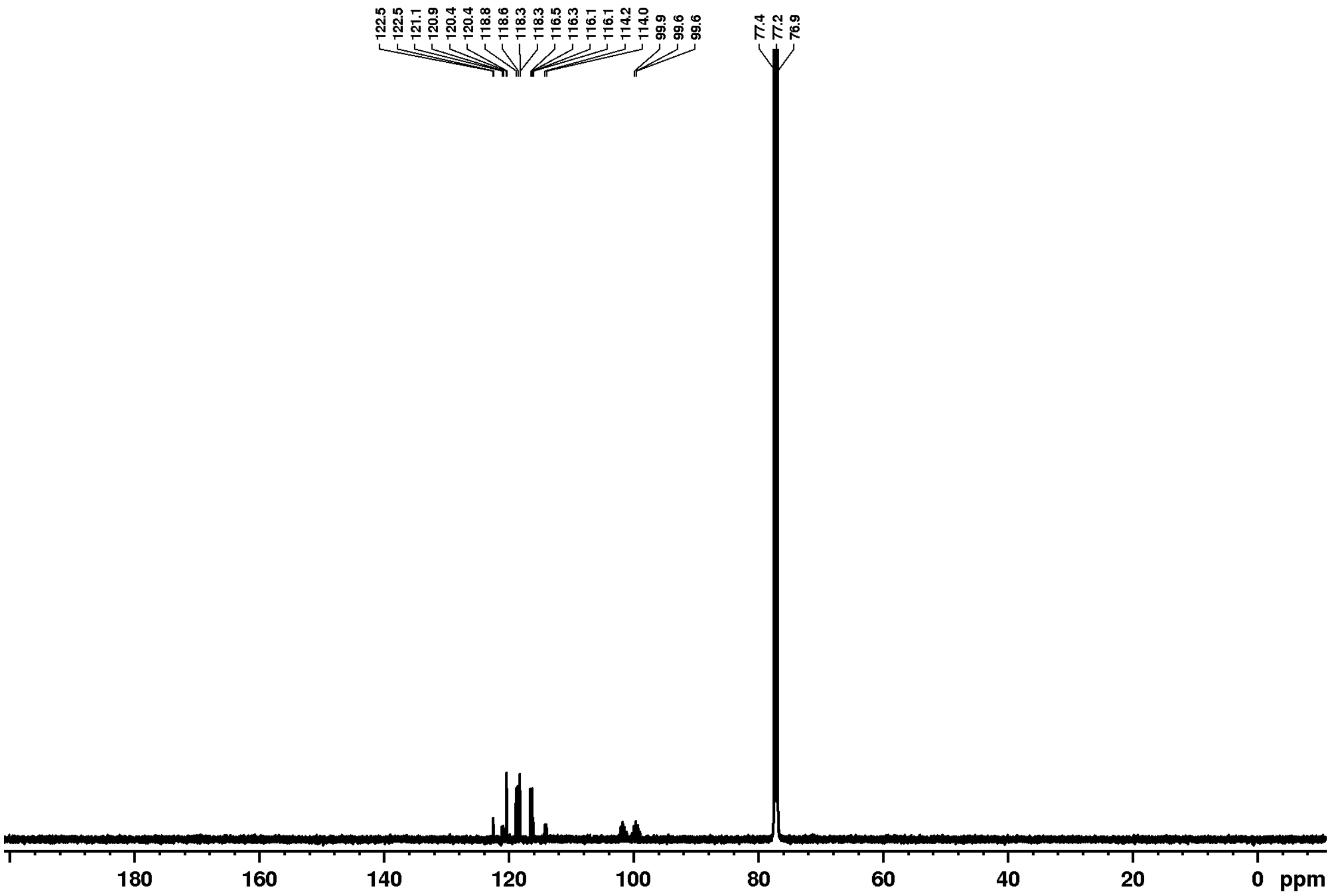

Figure S40. $^{13}C\{^1H\}$ NMR of $(CF_3)_2CHOCF_3$ (**4**) in $CDCl_3$.

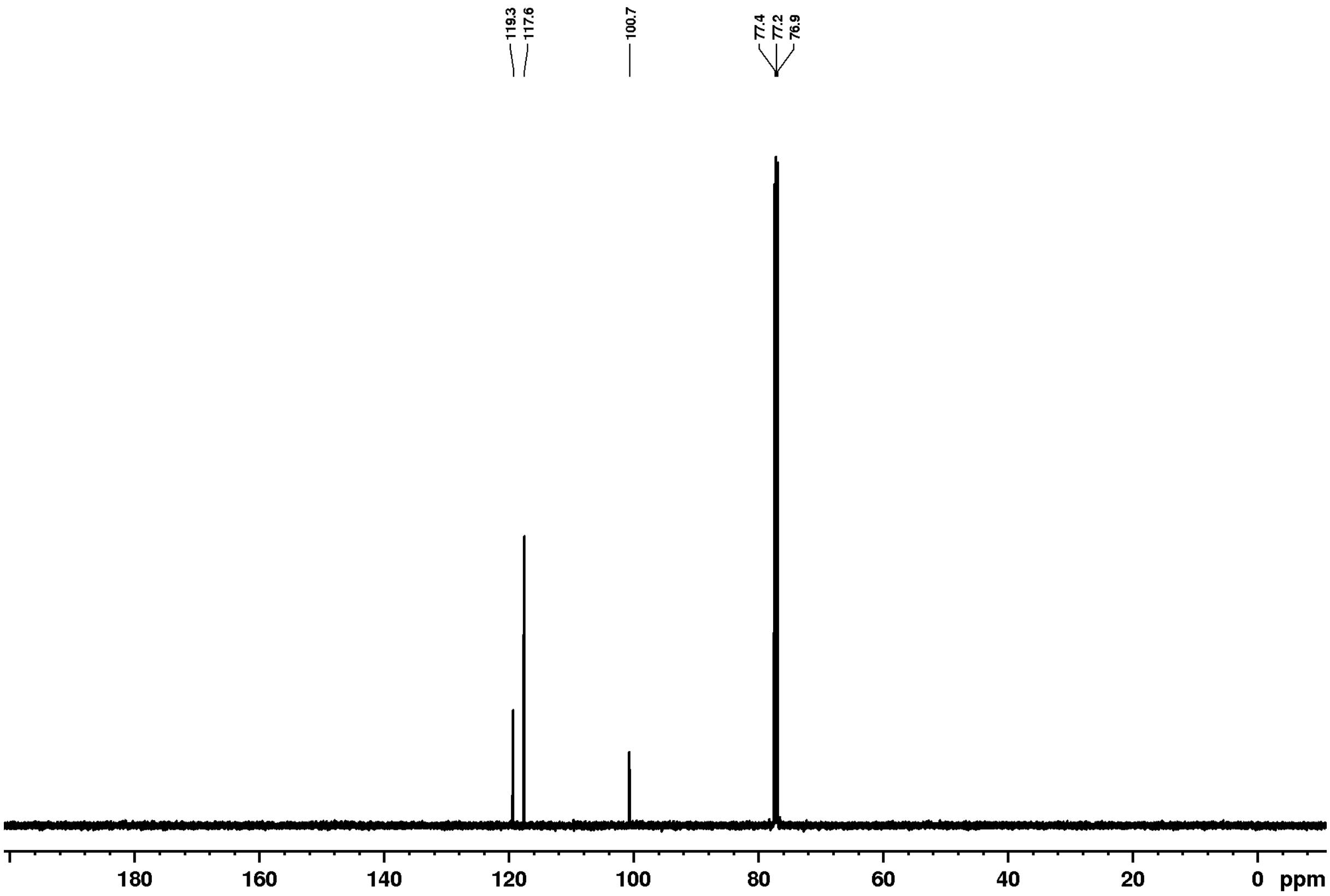

Figure S41. $^{13}C\{^{19}F\}$ NMR of $(CF_3)_2CHOCF_3$ (**4**) in $CDCl_3$.

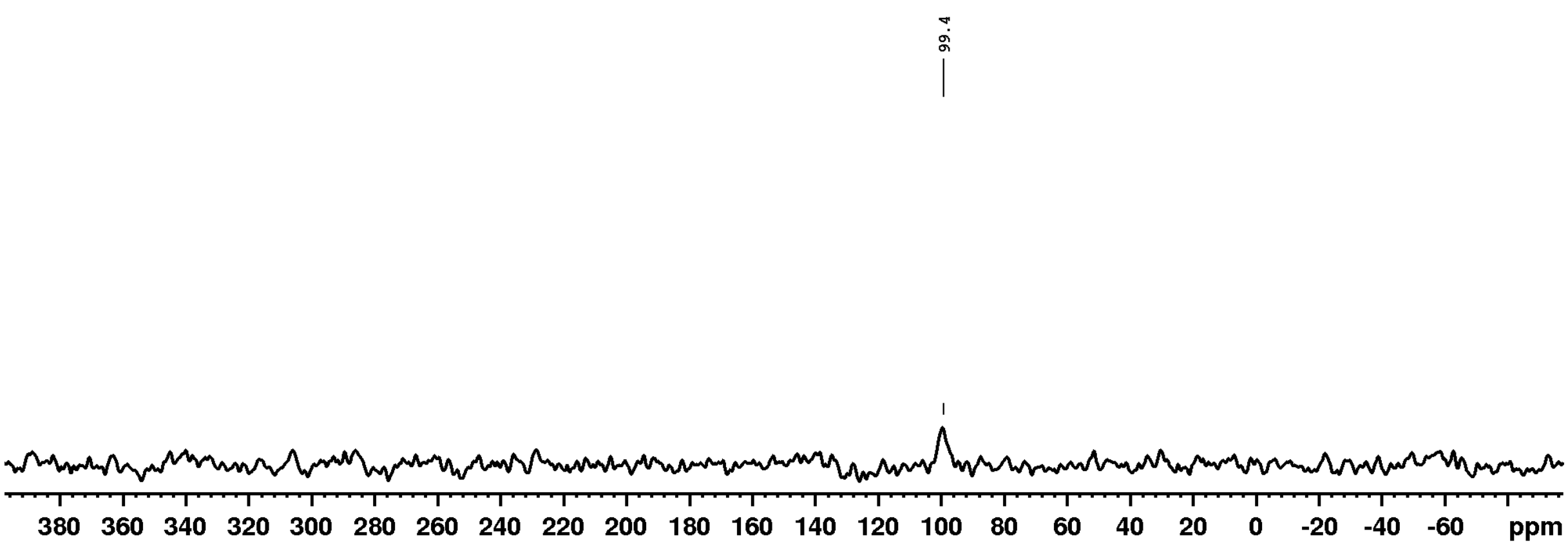

Figure S42. ^{17}O NMR of $(CF_3)_2CHOCF_3$ (**4**) in $CDCl_3$.

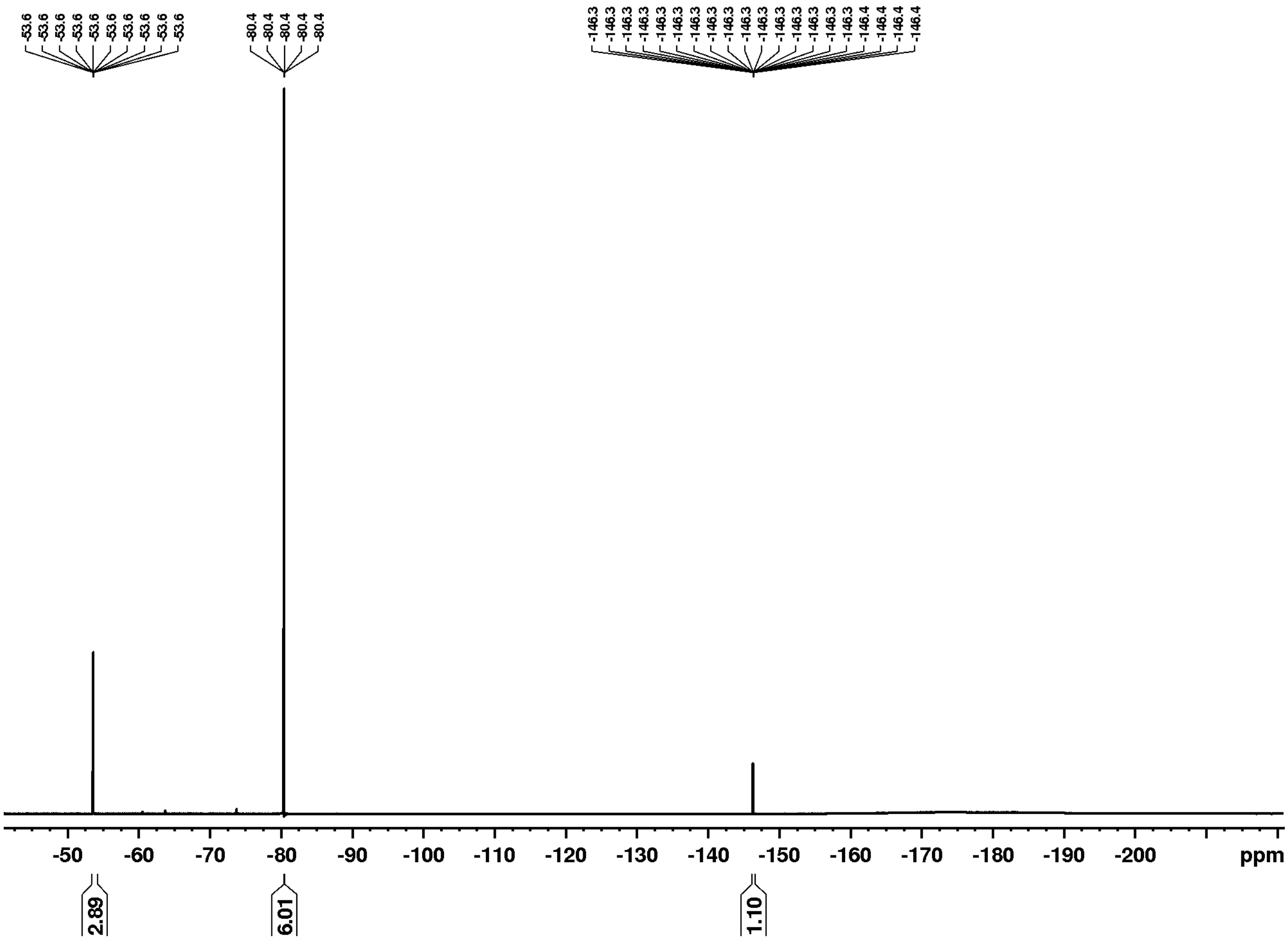

Figure S43. ^{19}F NMR of $(CF_3)_2CHOCF_3$ (**4**) in $CDCl_3$.

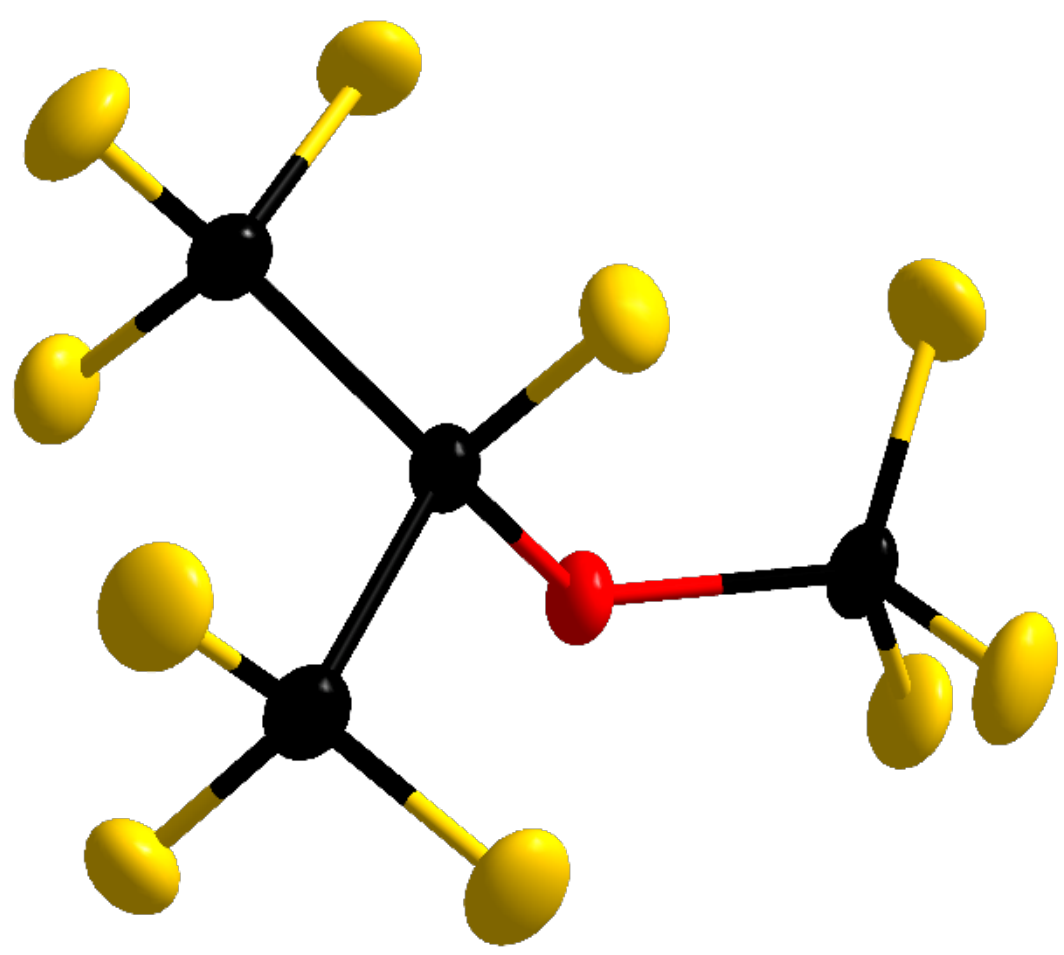

Figure S44. Solid state structure of $(CF_3)_2CFOCF_3$ (**4**).

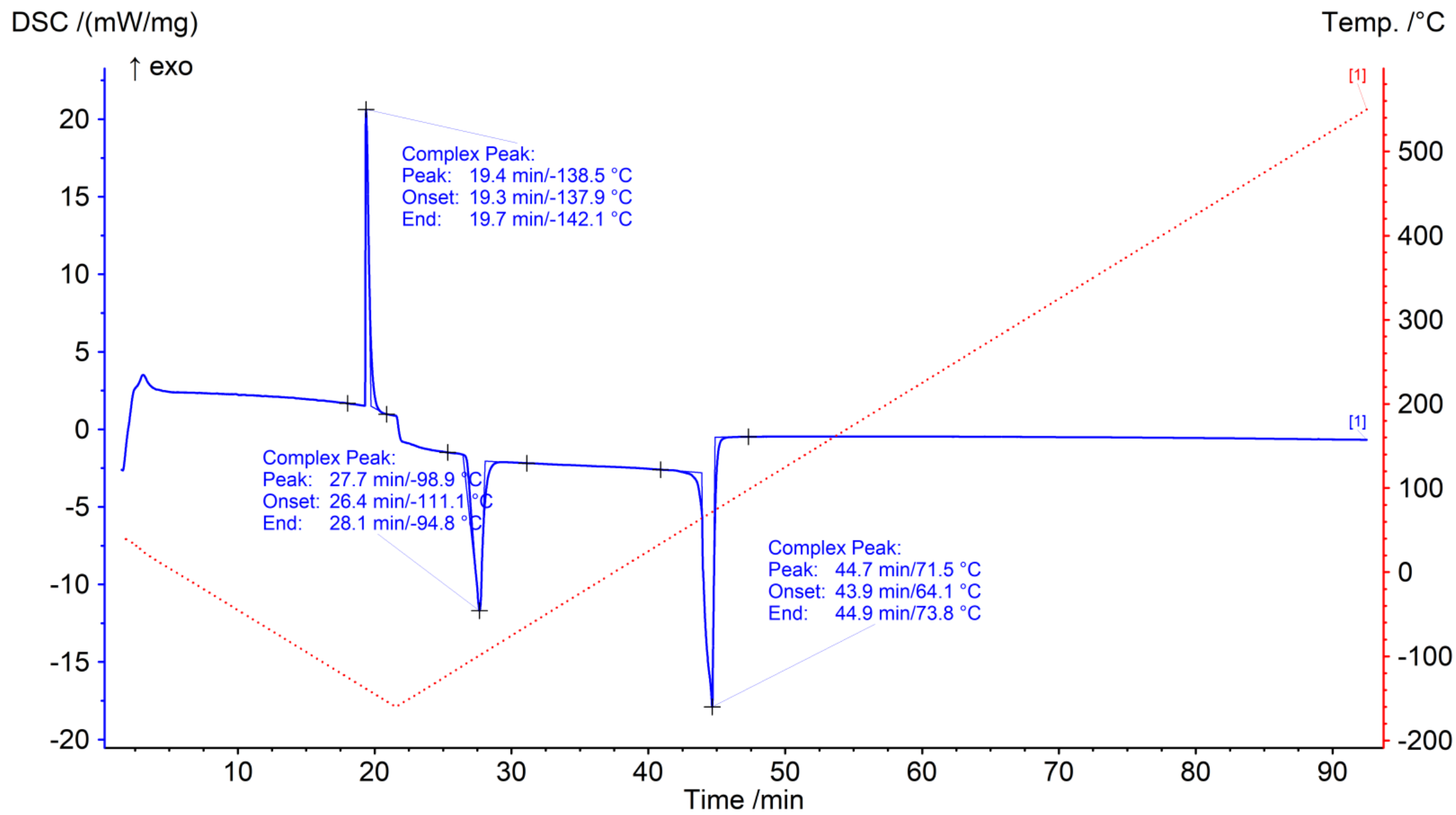

Figure S45. DSC curve of $(CF_3)_2CFOCF_3$ (**4**).

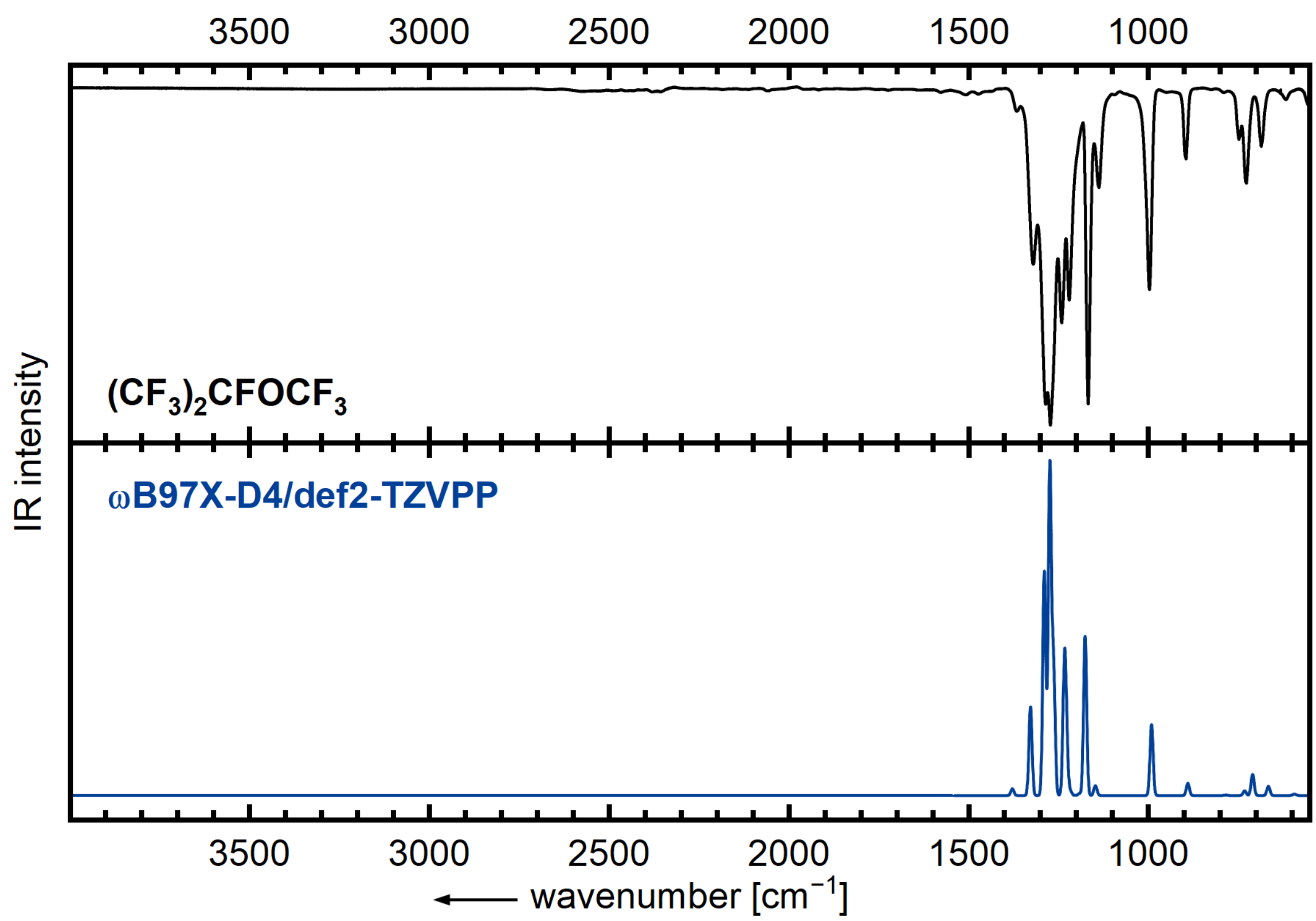

Figure S46. IR spectrum of $(CF_3)_2CFOCF_3$ (**4**, gas phase): measured (top) vs. calculated (ωB97X-D4/def2-TZVPP, bottom).

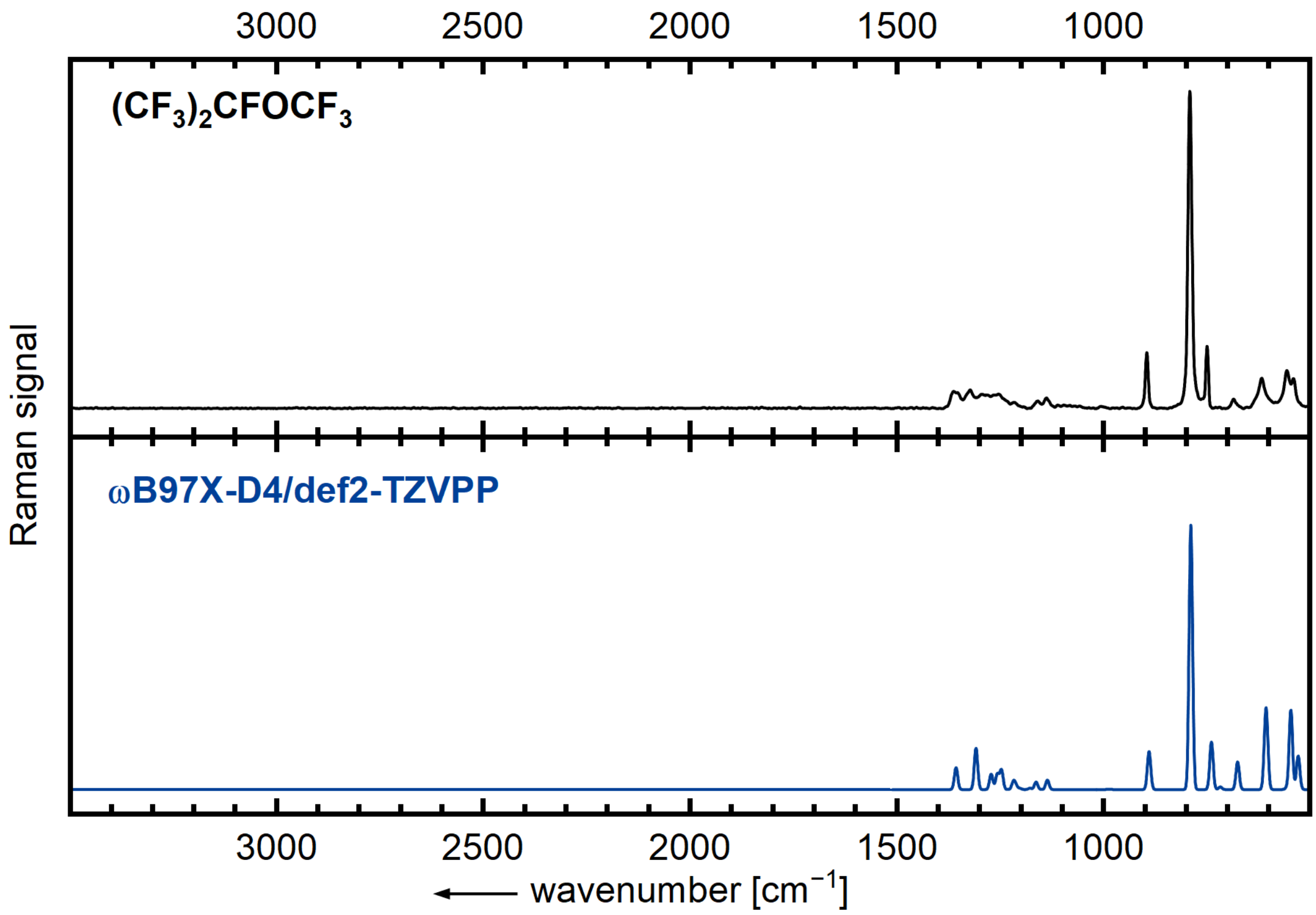

Figure S47. Raman spectrum of $(CF_3)_2CFOCF_3$ (**4**, liquid phase): measured (top) vs. calculated (ωB97X-D4/def2-TZVPP, bottom).

$(CF_3)_2CFOCHF_2$ (mixture with $(CF_3)_2CHOCF_3$, not isolated)

^{1}H NMR (400.3 MHz, $(CH_3)_2CO$): δ = 6.64 (t, 1H, $(CF_3)_2CFOC\mathbf{H}F_2$, $^2J(^{19}F, ^1H)$ = 69.1 Hz) ppm. **^{1}H{^{19}F} NMR** (400.3 MHz, $(CH_3)_2CO$): δ = 6.64 (s, 1H, $(CF_3)_2CFOC\mathbf{H}F_2$) ppm. **^{13}C NMR** (100.7 MHz, $(CH_3)_2CO$): δ = 117.8 (qm, 2C, $(\mathbf{C}F_3)_2CFOCHF_2$, $^1J(^{19}F, ^{13}C)$ = 289.1 Hz), 114.0 (tddsept, 1C, $(CF_3)_2CFO\mathbf{C}HF_2$, $^1J(^{19}F, ^{13}C)$ = 261.7 Hz, $^3J(^{19}F, ^{13}C)$ = 10.5 Hz, $^4J(^{19}F, ^{13}C)$ = 1.7 Hz), 100.7 (dsept, 1C, $(CF_3)_2\mathbf{C}FOCHF_2$, $^1J(^{19}F, ^{13}C)$ = 256.0 Hz, $^2J(^{19}F, ^{13}C)$ = 36.9 Hz) ppm.

^{13}C{^{1}H} NMR (100.7 MHz, $(CH_3)_2CO$): δ = 117.8 (qm, 2C, $(\mathbf{C}F_3)_2CFOCHF_2$, $^1J(^{19}F, ^{13}C)$ = 289.1 Hz), 114.0 (tdsept, 1C, $(CF_3)_2CFO\mathbf{C}HF_2$, $^1J(^{19}F, ^{13}C)$ = 261.7 Hz, $^1J(^{13}C, ^1H)$ = 224.8 Hz, $^3J(^{19}F, ^{13}C)$ = 10.5 Hz, $^4J(^{19}F, ^{13}C)$ = 1.7 Hz), 100.7 (dsept, 1C, $(CF_3)_2\mathbf{C}FOCHF_2$, $^1J(^{19}F, ^{13}C)$ = 256.0 Hz, $^2J(^{19}F, ^{13}C)$ = 36.9 Hz) ppm.

^{19}F NMR (470.6 MHz, $(CH_3)_2CO$): δ = −80.8 (dt, 6F, $(C\mathbf{F}_3)_2CFOCHF_2$, $^3J(^{19}F, ^{19}F)$ = 2.3 Hz, $^5J(^{19}F, ^{19}F)$ = 2.3 Hz), −82.9 (ddsept, 1F, $(CF_3)_2CFOCH\mathbf{F}_2$, $^2J(^{19}F, ^1H)$ = 69.2, $^4J(^{19}F, ^{19}F)$ = 4.4 Hz, $^5J(^{19}F, ^{19}F)$ = 2.3 Hz), −143.2 (m, 1F, $(CF_3)_2CFOCH\mathbf{F}_2$) ppm.

^{19}F{^{1}H} NMR (470.6 MHz, $(CH_3)_2CO$): δ = −80.8 (dt, 6F, $(C\mathbf{F}_3)_2CFOCHF_2$, $^3J(^{19}F, ^{19}F)$ = 2.3 Hz, $^5J(^{19}F, ^{19}F)$ = 2.3 Hz), −82.9 (dsept, 1F, $(CF_3)_2CFOCH\mathbf{F}_2$, $^4J(^{19}F, ^{19}F)$ = 4.4 Hz, $^5J(^{19}F, ^{19}F)$ = 2.3 Hz), −143.2 (m, 1F, $(CF_3)_2CFOCH\mathbf{F}_2$) ppm.

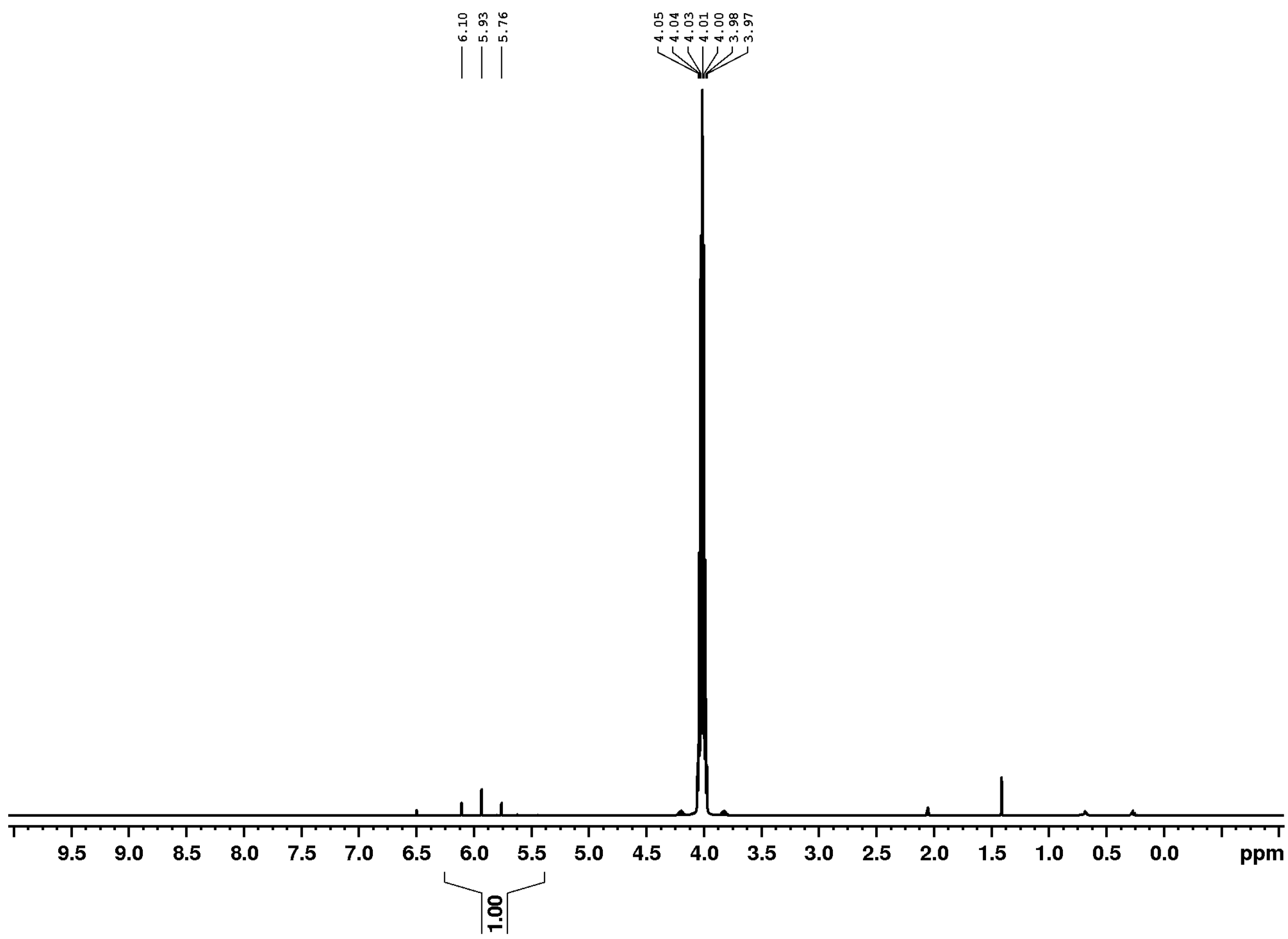

Figure S48. ^1H NMR of $(CF_3)_2CFOCHF_2$ (mixture with $(CF_3)_2CHOCF_3$) measured as neat substance with acetone-d_6 and CCl_3F as external standard.

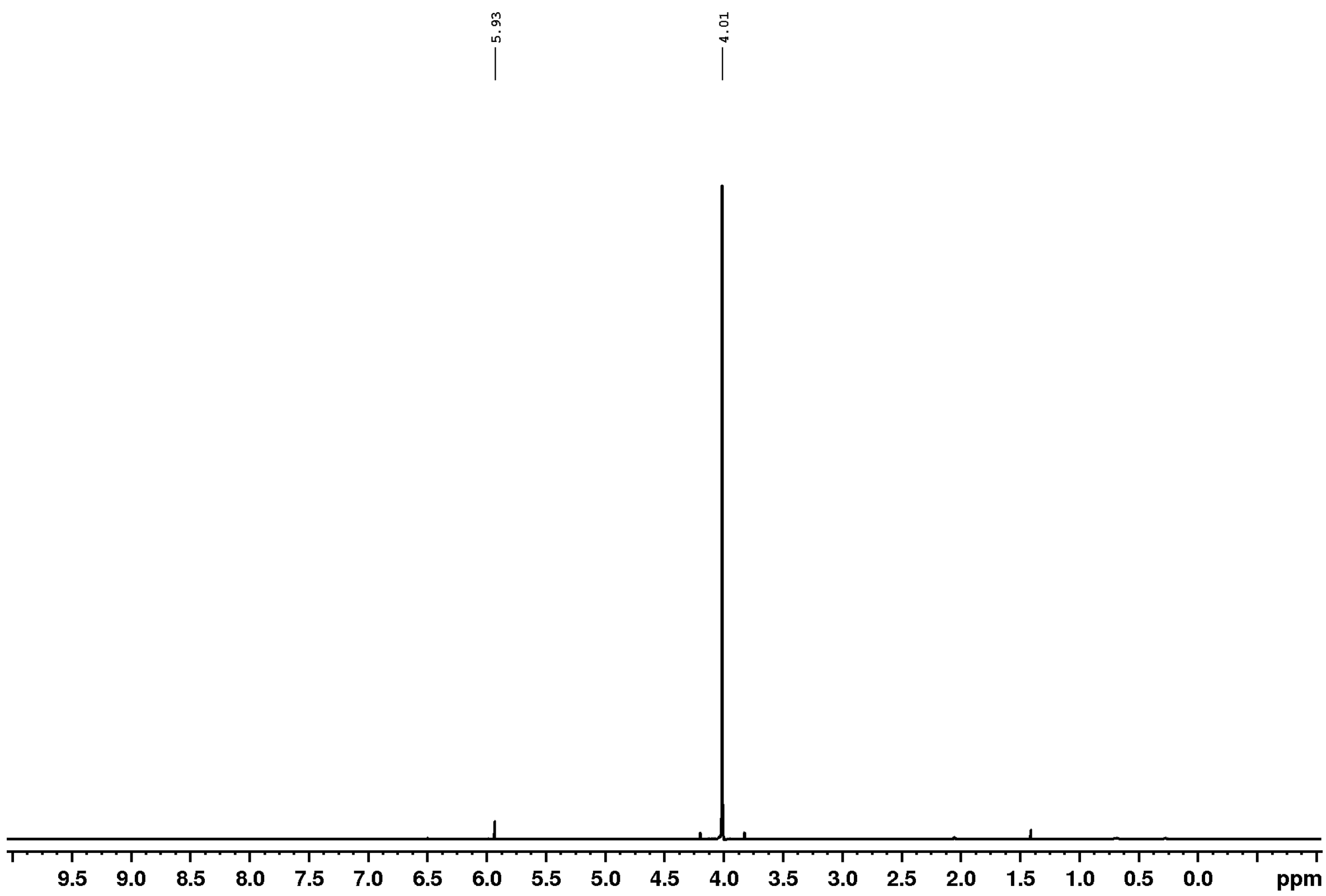

Figure S49. $^1H\{^{19}F\}$ NMR of $(CF_3)_2CFOCHF_2$ (mixture with $(CF_3)_2CHOCF_3$) measured as neat substance with acetone-d_6 and CCl_3F as external standard.

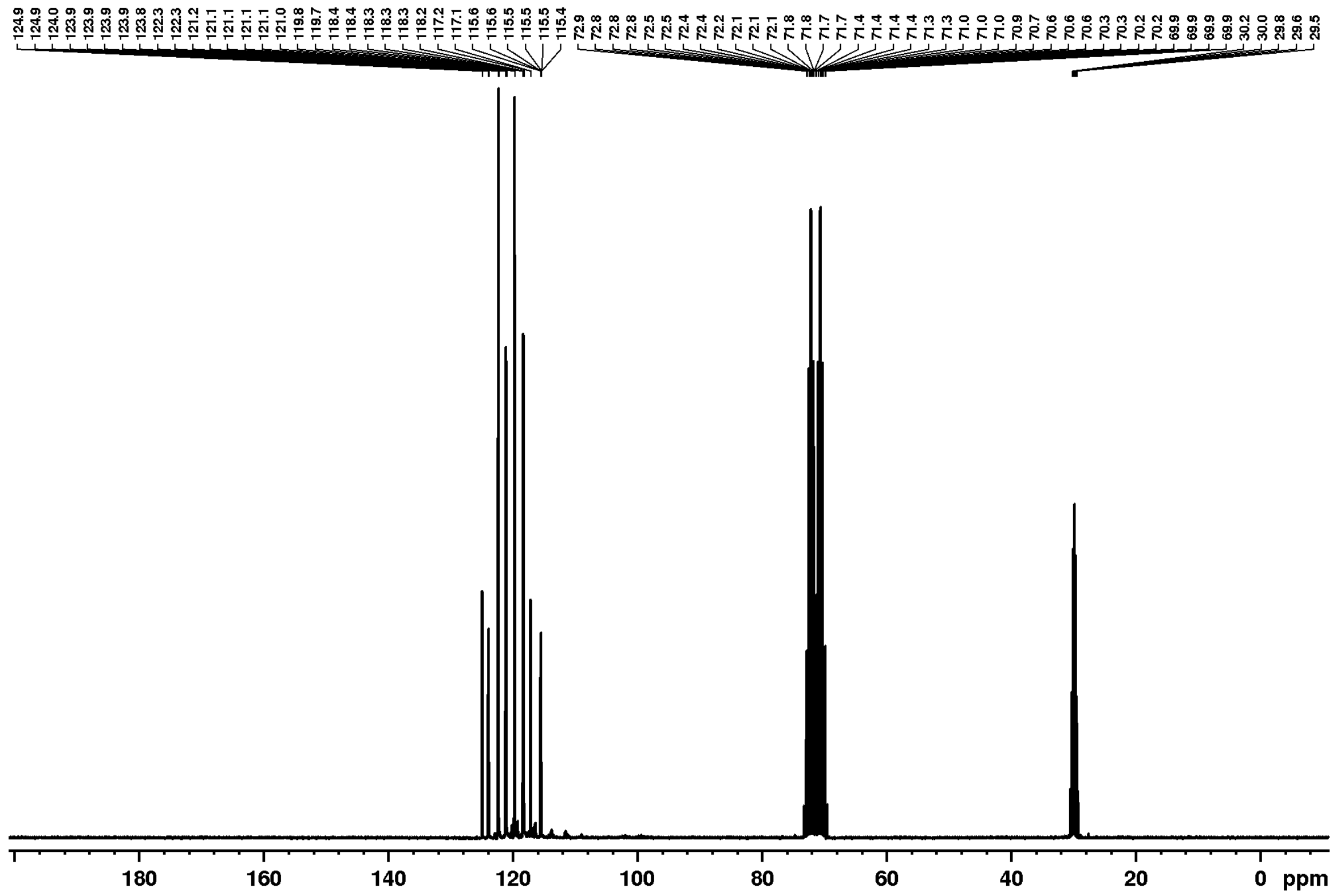

Figure S50. ^{13}C NMR of $(CF_3)_2CFOCHF_2$ (mixture with $(CF_3)_2CHOCF_3$) measured as neat substance with acetone-d_6 and CCl_3F as external standard.

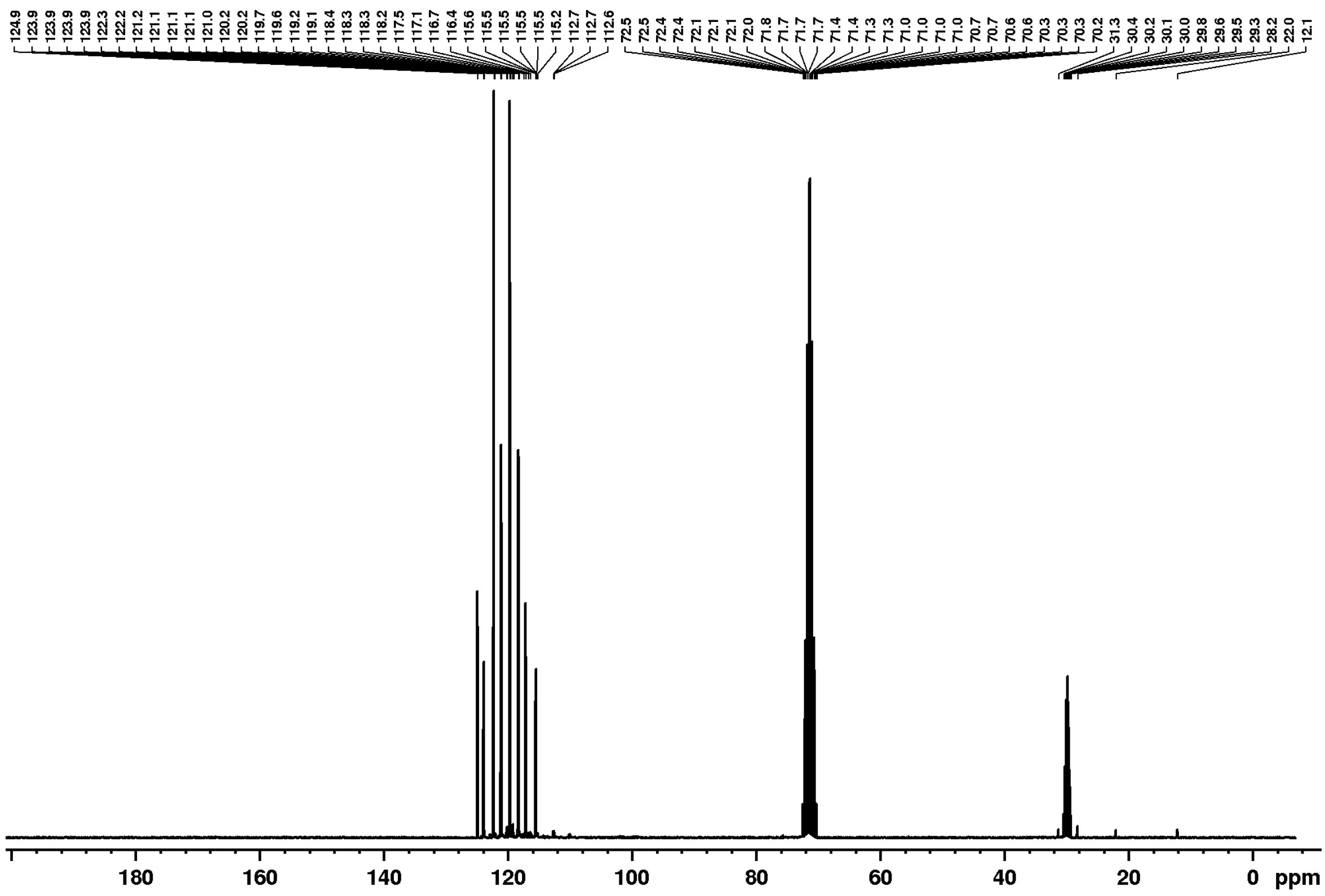

Figure S51. $^{13}C\{^1H\}$ NMR of $(CF_3)_2CFOCHF_2$ (mixture with $(CF_3)_2CHOCF_3$) measured as neat substance with acetone-d_6 and CCl_3F as external standard.

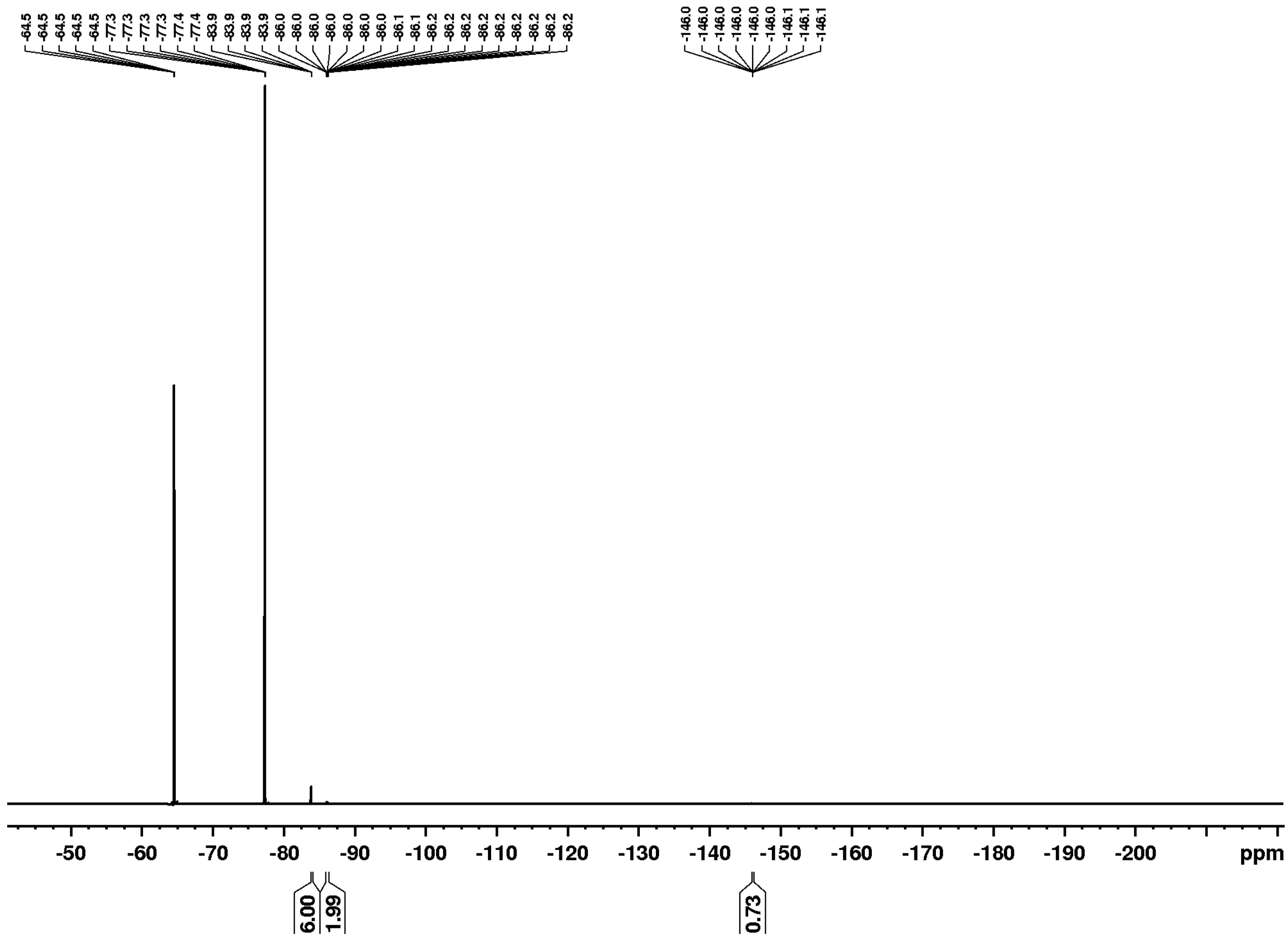

Figure S52. ^{19}F NMR of $(CF_3)_2CFOCHF_2$ (mixture with $(CF_3)_2CHOCF_3$) measured as neat substance with acetone-d_6 and CCl_3F as external standard.

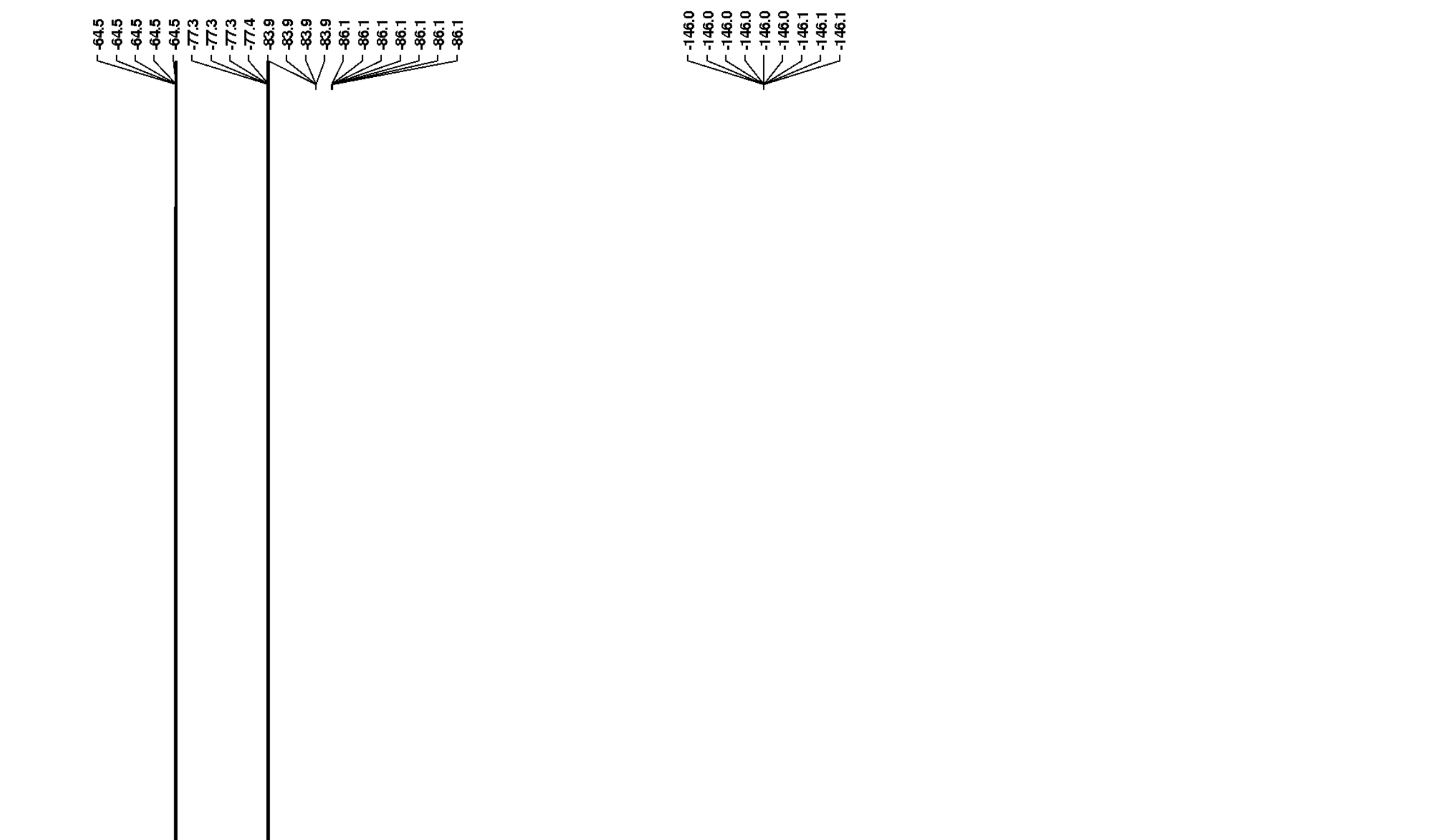

Figure S53. ^{19}F NMR of $(CF_3)_2CFOCHF_2$ (mixture with $(CF_3)_2CHOCF_3$) measured as neat substance with acetone-d_6 and CCl_3F as external standard.

3.2. Electrochemical fluorination of n-$C_3F_7OCH_3$ (Novec7000)

A 430 mL stainless steel cell with nickel electrodes (effective anode area S = 4.4 dm^2), the construction of which is described elsewhere [15], was filled with anhydrous hydrogen fluoride (335 mL). A constant voltage of 5.0 V was applied for 23 hours. $C_3F_7COCH_3$ (314.4 g, 1.572 mol) was added at regular intervals to the pre-electrolyzed anhydrous HF. The gaseous products from the cell were collected in a PTFE trap maintained at −78 °C. The cell was operated at 4.6 – 6.0 V and the current was held between 0.4 and 1.5 A. The cell temperature was maintained between 2 – 4 °C and the temperature of the condenser was kept between −21 and −15 °C. After consumption of 254 Ah the reaction was stopped. In total, 324.8 g of a crude organic phase was collected from the cold traps, consisting of $C_3F_7OCF_3$ (59%), $C_3F_7OCHF_2$ (32%), $C_3F_7OCH_2F$ (1%) and $C_3F_7OCH_3$ (8%). From the cell no crude organic was obtained. The aHF from the cell was stored at −78°C. After one day the aHF phase was carefully poured onto ice (300 g) but no organic layer formed. The crude organic was stirred with potassium fluoride to remove residual HF. Afterwards, the organic layer was separated from the formed bifluoride by condensation.

The separation of the individual species was carried out via distillation. $C_3F_7OCH_2F$ was not isolated.

***n*-$C_3F_7OCH_3$**

Boiling point (vapor pressure): T_{bp} = 34.3 °C at 1013 mbar (Antoine parameter: A = 3.991; B = 1012.1; C = −53.478 for equation: $p = 10^{(A-B/(T+C))}$).

Enthalpy of vaporization (T = 298.15 K): $\Delta_{vap}H$ = 28.8 kJ mol^{-1} (for equation: $\Delta_{vap}H = \ln(10)\cdot R\cdot T^2\cdot(B/(T+C)^2)/1000$).

^{1}H NMR (600.2 MHz, $CDCl_3$): δ = 3.73 (s, 3H, $C_3F_7OC\mathbf{H_3}$) ppm.

^{1}H{^{19}F} NMR (600.2 MHz, $CDCl_3$): δ = 3.79 (m, 3H, $C_3F_7OC\mathbf{H_3}$) ppm.

^{13}C NMR (100.7 MHz, $(CH_3)_2CO$): δ = 117.7 (qt, 1C, $\mathbf{C}F_3CF_2CF_2OCH_3$, $^1J(^{19}F, ^{13}C)$ = 284.9 Hz, $^2J(^{19}F, ^{13}C)$ = 33.4 Hz), 117.0 (ttq, 1C, $CF_3CF_2\mathbf{C}F_2OCH_3$, $^1J(^{19}F, ^{13}C)$ = 270.9 Hz, $^2J(^{19}F, ^{13}C)$ = 29.5 Hz, $^3J(^{113}C, ^1H)$ = 3.8 $CF_3\mathbf{C}F_2CF_2OCH_3$, $^1J(^{19}F, ^{13}C)$ = 264.3 Hz), 50.0 (qt, 1C, $CF_3CF_2CF_2O\mathbf{C}H_3$, $^1J(^{13}C, ^1H)$ = 148.4 Hz, $^3J(^{19}F, ^{13}C)$ = 6.9 Hz) ppm.**^{13}C{^{1}H} NMR** (150.9 MHz, $CDCl_3$): δ = 117.7 (qt, 1C, $\mathbf{C}F_3CF_2CF_2OCH_3$, $^1J(^{19}F, ^{13}C)$ = 286.4 Hz, $^2J(^{19}F, ^{13}C)$ = 33.4 Hz), 116.9 (tt, 1C, $CF_3CF_2\mathbf{C}F_2OCH_3$, $^1J(^{19}F, ^{13}C)$ = 272.2 Hz, $^2J(^{19}F, ^{13}C)$ = 29.7 Hz), $CF_3\mathbf{C}F_2CF_2OCH_3$, $^1J(^{19}F, ^{13}C)$ = 267.0 Hz), 51.7 (t, 1C, $CF_3CF_2CF_2O\mathbf{C}H_3$, $^3J(^{19}F, ^{13}C)$ = 6.5 Hz) ppm.

^{13}C{^{19}F} NMR (150.9 MHz, $CDCl_3$): δ = 117.7 (s, 1C, $\mathbf{C}F_3CF_2CF_2OCH_3$), 116.9 (q, 1C, $CF_3CF_2\mathbf{C}F_2OCH_3$, $^3J(^{13}C, ^1H)$ = 3.8 Hz), 107.3 (s, $CF_3\mathbf{C}F_2CF_2OCH_3$), 51.7 (q, 1C, $CF_3CF_2CF_2O\mathbf{C}H_3$, $^1J(^{13}C, ^1H)$ = 148.8 Hz) ppm.

^{17}O NMR (81.4 MHz, $CDCl_3$): δ = 40.5 (s, 1O, $CF_3CF_2CF_2\mathbf{O}CH_3$) ppm.

^{19}F NMR (564.7 MHz, $CDCl_3$): δ = −81.9 (t, 3F, $C\mathbf{F_3}CF_2CF_2OCH_3$, $^3J(^{19}F, ^{19}F)$ = 7.1 Hz), −89.8 (m, 2F, $CF_3C\mathbf{F_2}C\mathbf{F_2}OCH_3$), −129.8 (t, 2F, $CF_3CF_2C\mathbf{F_2}OCH_3$, $^3J(^{19}F, ^{19}F)$ = 3.8 Hz) ppm.

^{19}F{^{1}H} NMR (564.7 MHz, $CDCl_3$): δ = −81.9 (t, 3F, $C\mathbf{F_3}CF_2CF_2OCH_3$, $^3J(^{19}F, ^{19}F)$ = 7.1 Hz), −89.8 (m, 2F, $CF_3C\mathbf{F_2}CF_2\mathbf{O}CH_3$), −129.8 (t, 2F, $CF_3CF_2C\mathbf{F_2}OCH_3$, $^3J(^{19}F, ^{19}F)$ = 3.8 Hz) ppm.

GC-MS [t = 0.45 min] m/z^+: 200 $[M]^+$, 199 [M −H], 181 [M −F], 169 [M $-OCH_3$], 131 [M $-CF_3$], 119 [M $-CF_2OCH_3$], 100 [M $-CF_3OCH_3$], 81 [M $-C_2F_5$], 69 [M $-C_2F_4OCH_3$].

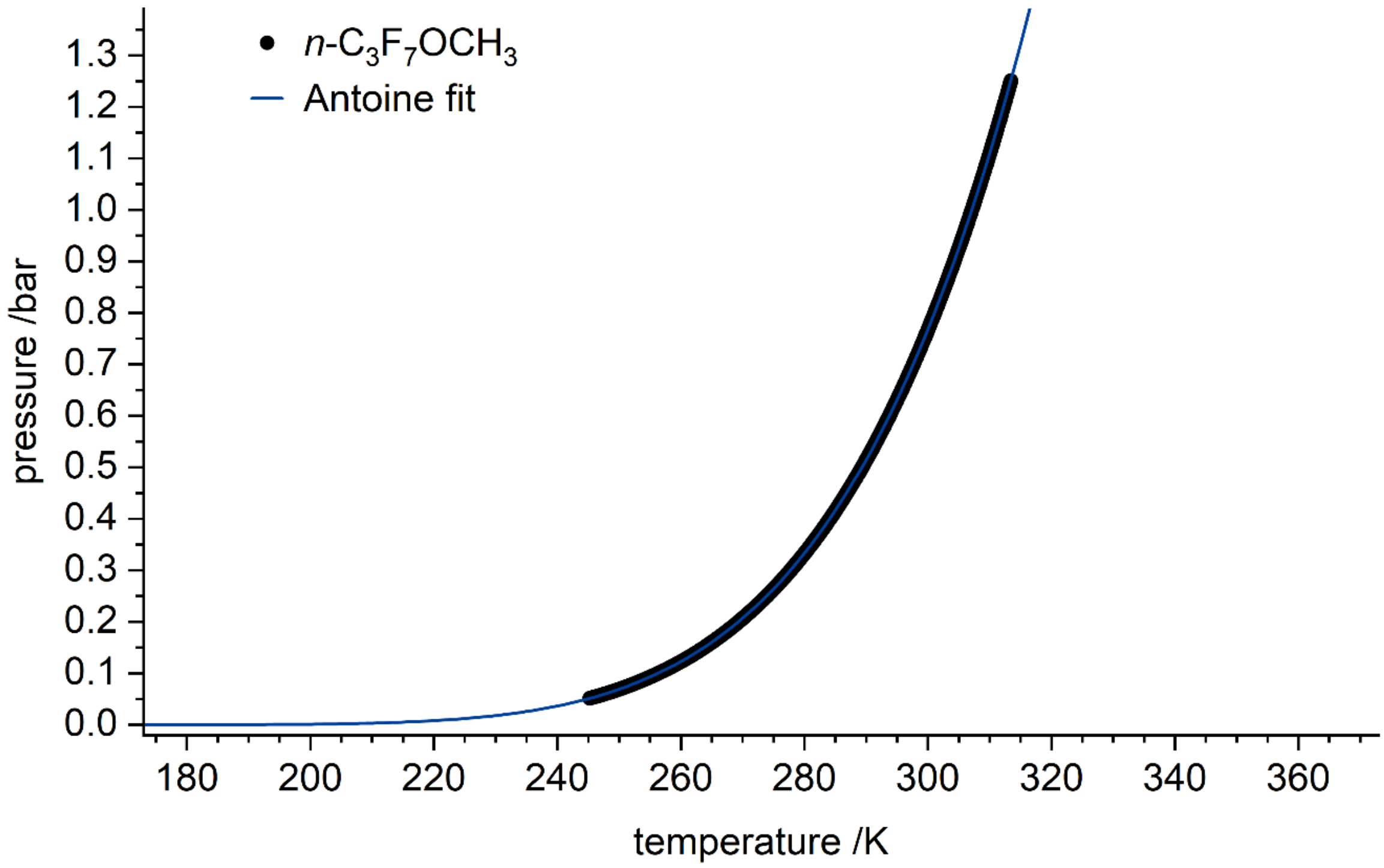

Figure S54. Vapor pressure measurement and Antoine fit of $CF_3CF_2CF_2OCH_3$ (Antoine parameter: A = 3.991; B = 1012.1; C = −53.478 for equation: $p = 10^{(A-B/(T+C))}$).

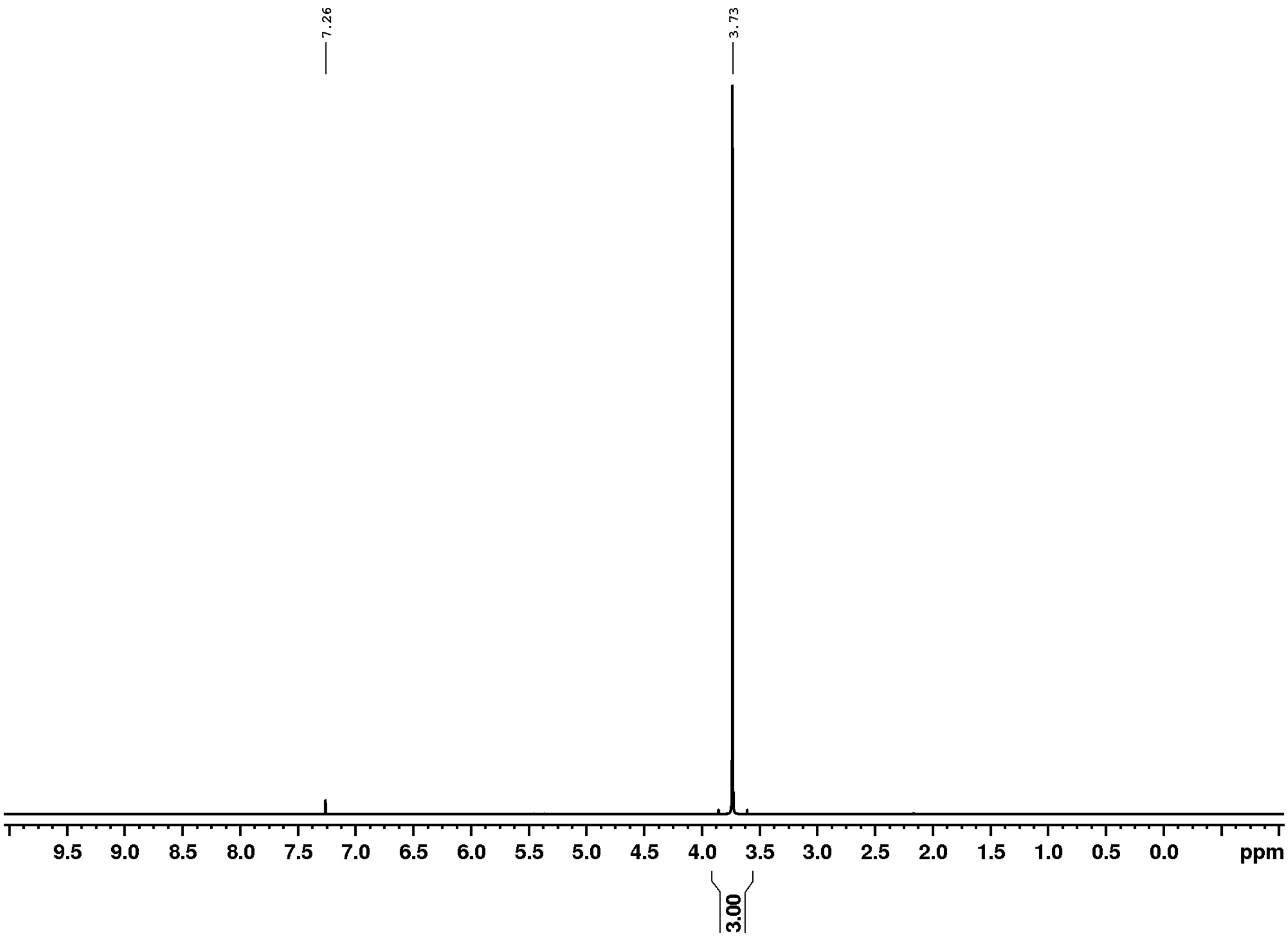

Figure S55. ^{1}H NMR of *n*-$C_3F_7OCH_3$ in $CDCl_3$.

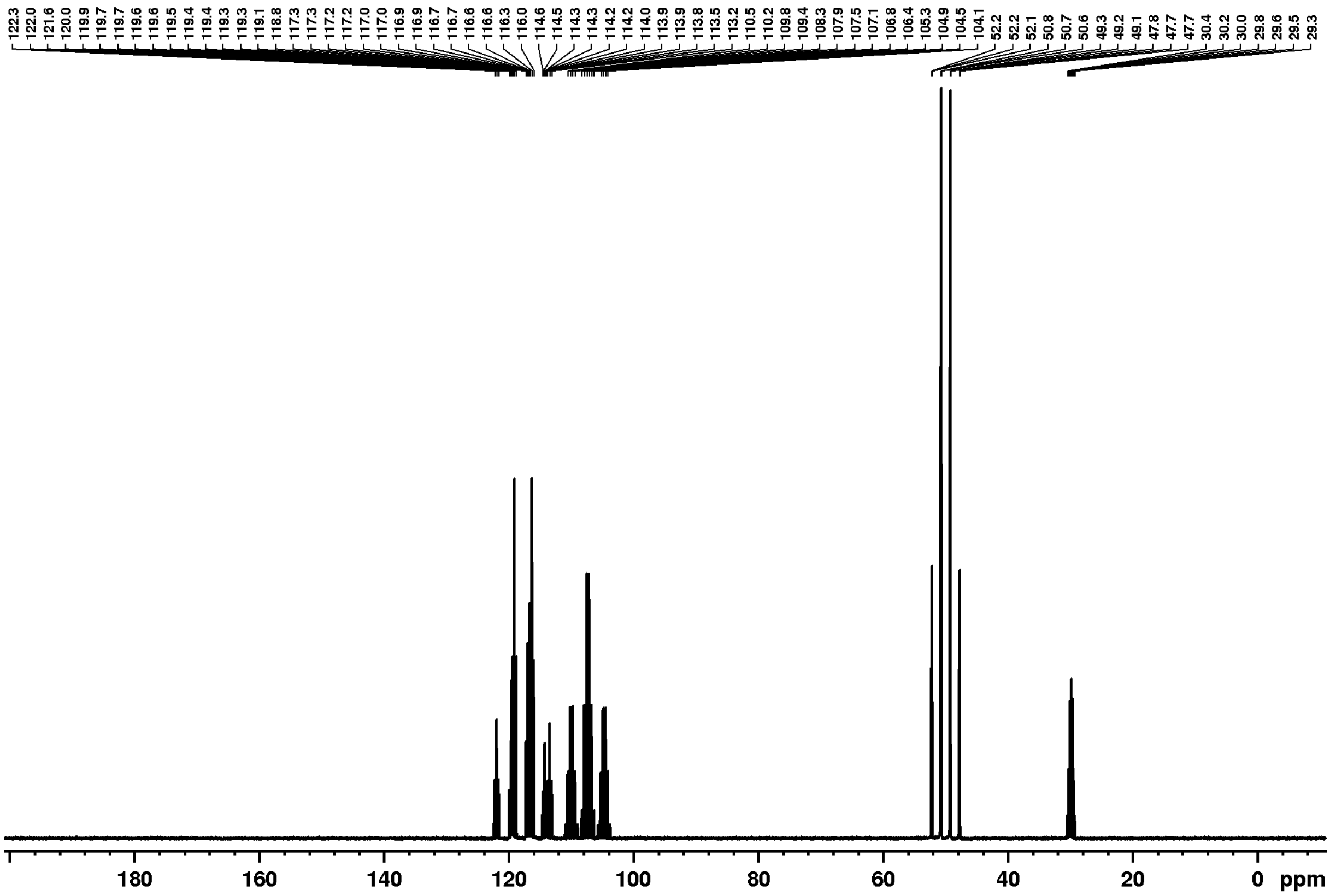

Figure S 56. ^{13}C NMR of *n*-$C_3F_7OCH_3$ measured as neat substance with acetone-d_6 and CCl_3F as external standard.

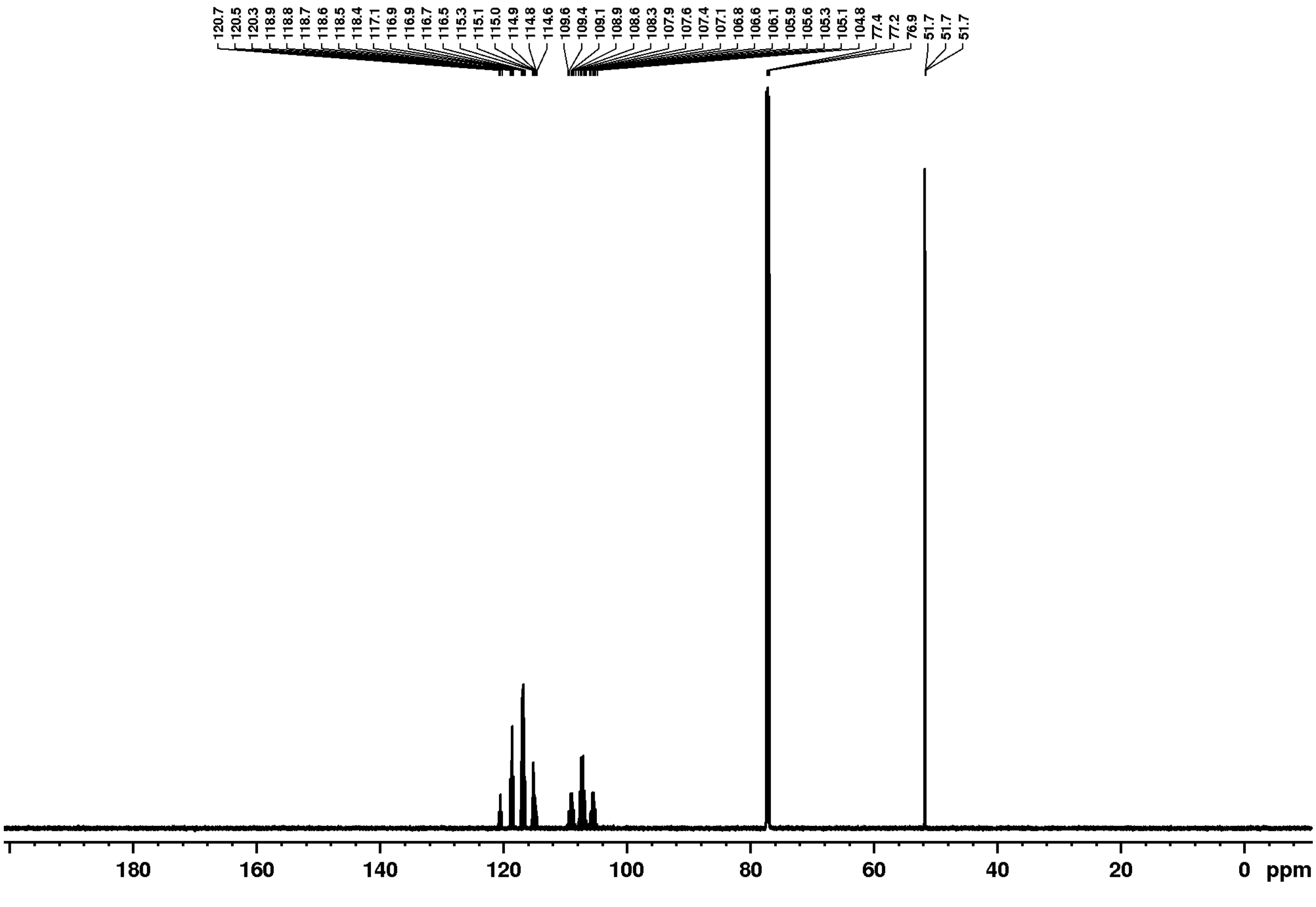

Figure S57. $^{13}C\{^1H\}$ NMR of *n*-$C_3F_7OCH_3$ in $CDCl_3$.

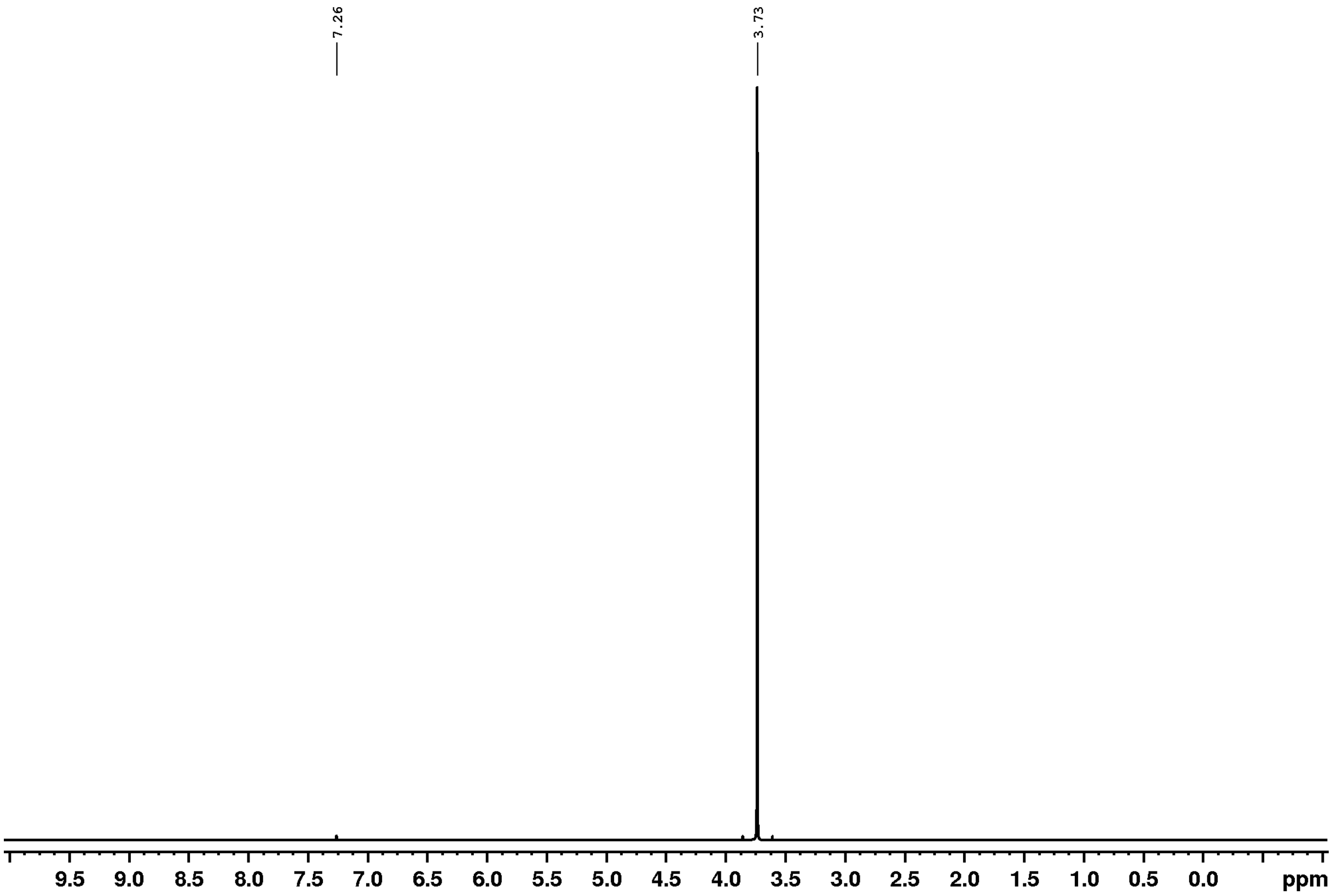

Figure S58. $^{1}H\{^{19}F\}$ NMR of *n*-$C_3F_7OCH_3$ in $CDCl_3$.

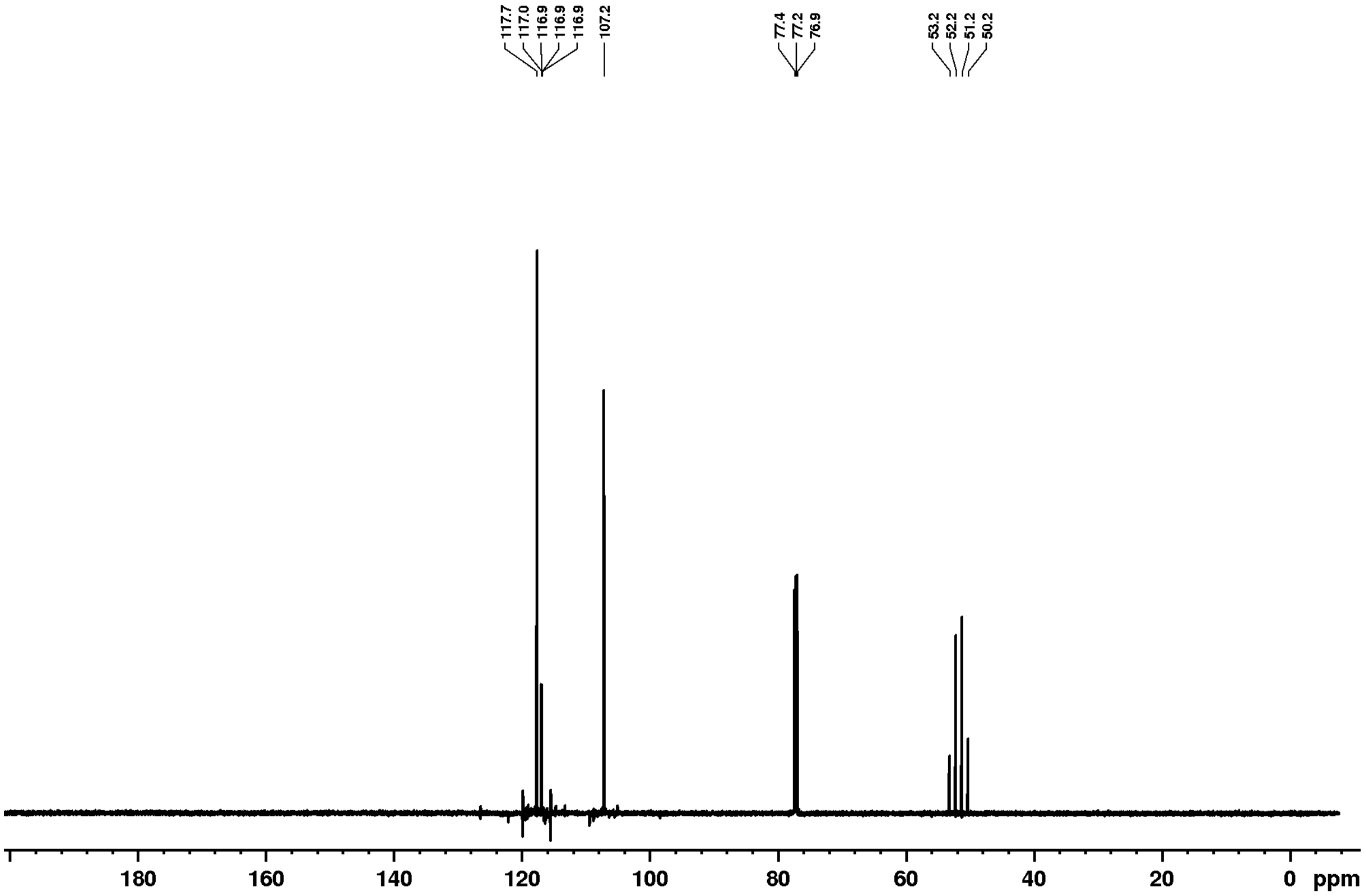

Figure S59. $^{13}C\{^{19}F\}$ NMR of *n*-$C_3F_7OCH_3$ in $CDCl_3$.

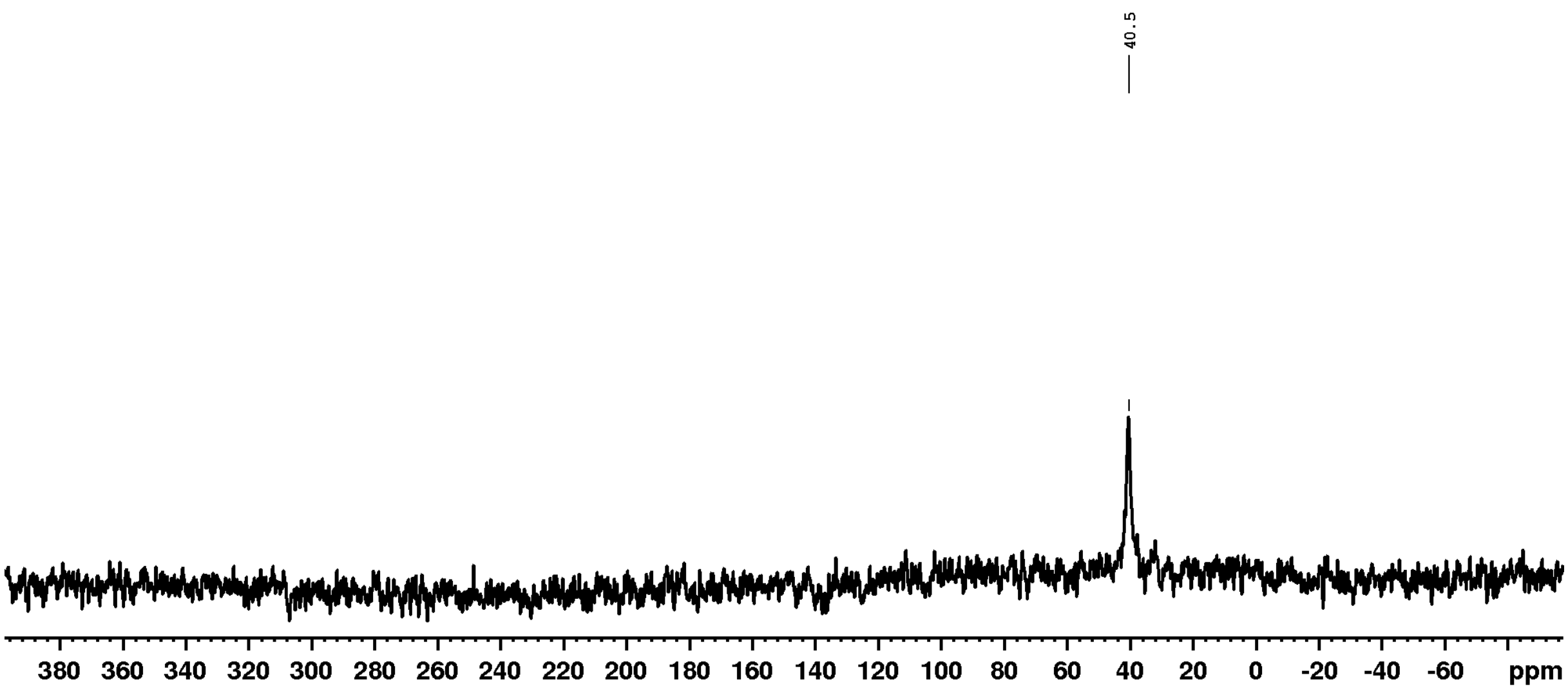

Figure S60. ^{17}O NMR of *n*-$C_3F_7OCH_3$ in $CDCl_3$.

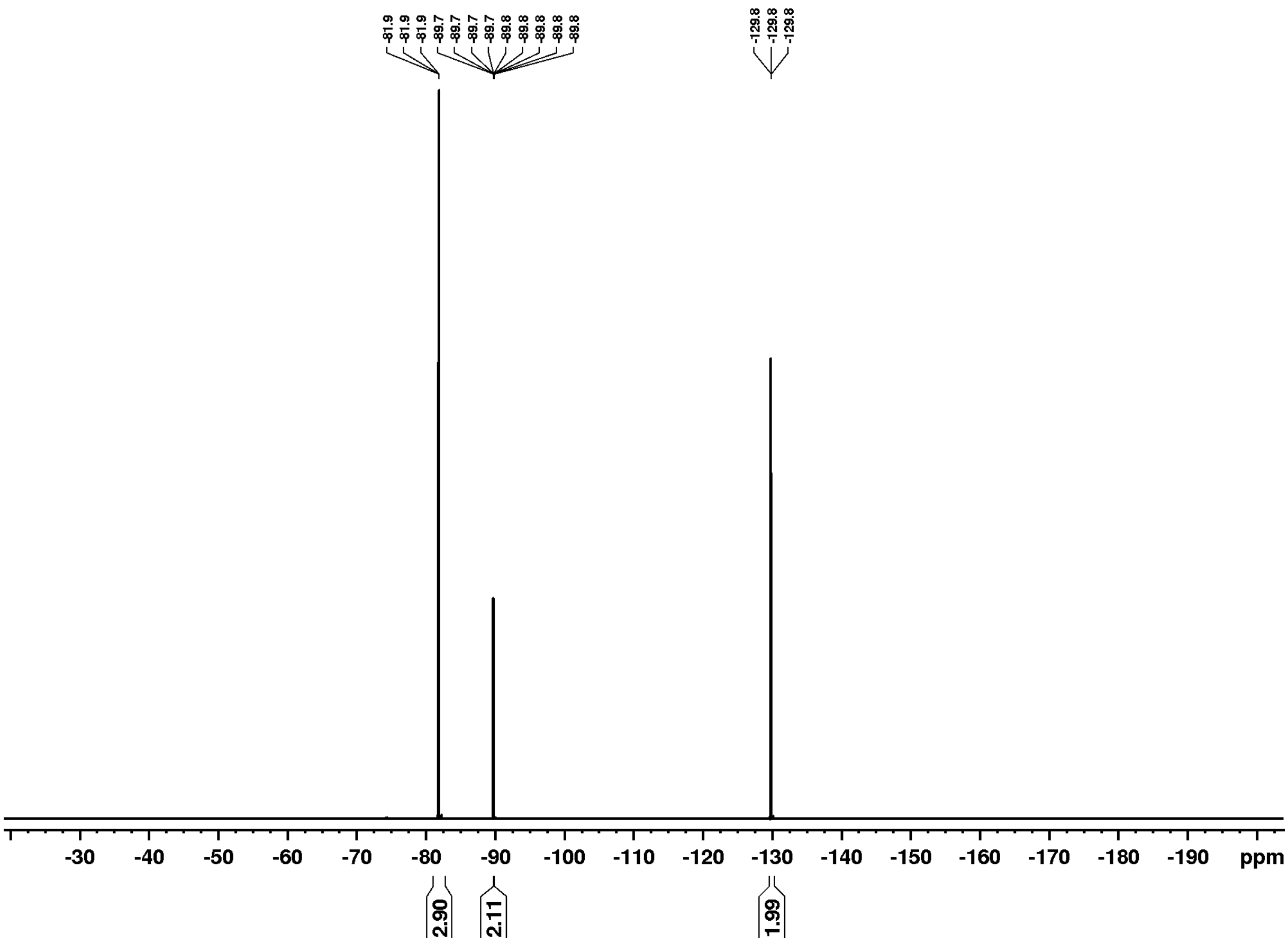

Figure S61. ^{19}F NMR of *n*-$C_3F_7OCH_3$ in $CDCl_3$.

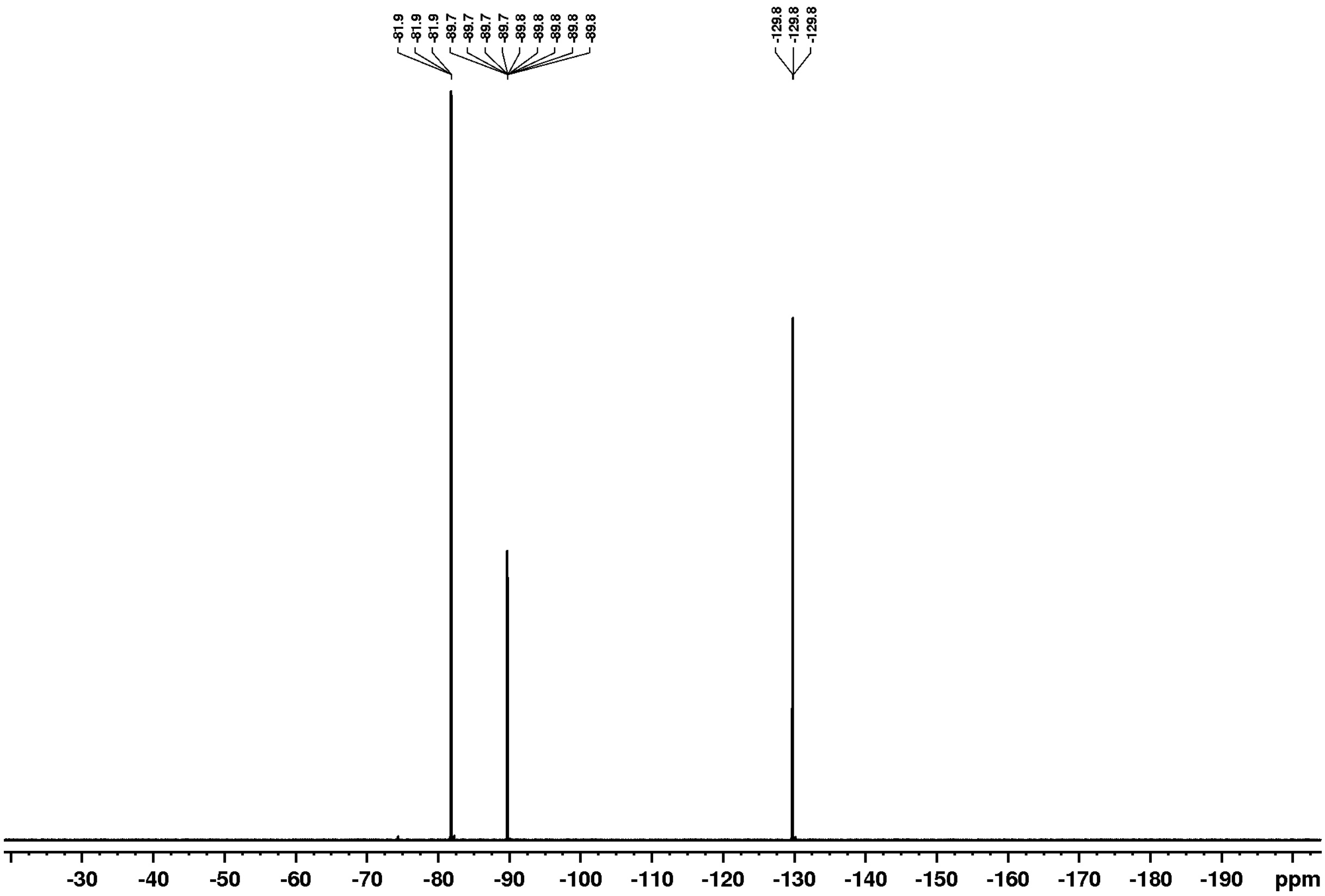

Figure S62. $^{19}F\{^{1}H\}$ NMR of *n*-$C_3F_7OCH_3$ in $CDCl_3$.

***n*-$C_3F_7OCH_2F$** (mixture with *n*-$C_3F_7OCH_3$, not isolated)

^{1}H NMR (600.2 MHz, $CDCl_3$): δ = 5.64 (d, 2H, $CF_3CF_2CF_2OC\mathbf{H}_2F$, $^2J(^{19}F, ^1H)$ = 50.8 Hz) ppm.

^{1}H{^{19}F} NMR (600.3 MHz, $CDCl_3$): δ = 5.64 (s, 2H, $CF_3CF_2CF_2OC\mathbf{H}_2F$) ppm.

^{13}C NMR (100.7 MHz, $(CH_3)_2CO$): δ = 117.5 (qt, 1C, $\mathbf{C}F_3CF_2CF_2OCH_2F$, $^1J(^{19}F, ^{13}C)$ = 285.1 Hz, $^2J(^{19}F, ^{13}C)$ = 33.1 Hz), 116.3 (ttd, 1C, $CF_3CF_2\mathbf{C}F_2OCH_2F$, $^1J(^{19}F, ^{13}C)$ = 277.2 Hz, $^2J(^{19}F, ^{13}C)$ = 30.4 Hz, $^3J(^{19}F, ^{13}C)$ = 1.7 Hz), 107.1 (tq, 1C, $CF_3\mathbf{C}F_2CF_2OCH_2F$, $^1J(^{19}F, ^{13}C)$ = 265.5 Hz, $^2J(^{19}F, ^{13}C)$ = 38.8 Hz), 95.2 (dtt, 1C, $CF_3CF_2CF_2O\mathbf{C}H_2F$, $^1J(^{19}F, ^{13}C)$ = 229.4 Hz, $^1J(^{13}C, ^1H)$ = 180.5 Hz, $^3J(^{19}F, ^{13}C)$ = 6.1 Hz) ppm.

^{13}C{^{1}H} NMR (150.9 MHz, $CDCl_3$): δ = 117.5 (qt, 1C, $\mathbf{C}F_3CF_2CF_2OCH_2F$, $^1J(^{19}F, ^{13}C)$ = 286.8 Hz, $^2J(^{19}F, ^{13}C)$ = 33.2 Hz), 116.3 (ttd, 1C, $CF_3CF_2\mathbf{C}F_2OCH_2F$, $^1J(^{19}F, ^{13}C)$ = 278.5 Hz, $^2J(^{19}F, ^{13}C)$ = 30.4 Hz, $^3J(^{19}F, ^{13}C)$ = 1.7 Hz), 107.0 (tq, 1C, $CF_3\mathbf{C}F_2CF_2OCH_2F$, $^1J(^{19}F, ^{13}C)$ = 265.9 Hz, $^2J(^{19}F, ^{13}C)$ = 38.8 Hz), 95.7 (dt, 1C, $CF_3CF_2CF_2O\mathbf{C}H_2F$, $^1J(^{19}F, ^{13}C)$ = 231.0 Hz, $^3J(^{19}F, ^{13}C)$ = 5.9 Hz) ppm.**^{13}C{^{19}F} NMR** (150.9 MHz, $CDCl_3$): δ = 117.5 (s, 1C, $\mathbf{C}F_3CF_2CF_2OCH_2F$), 116.2 (t, 1C, $CF_3CF_2\mathbf{C}F_2OCH_2F$, $^3J(^{13}C, ^1H)$ = 6.0 Hz), 106.9 (s, 1C, $CF_3\mathbf{C}F_2CF_2OCH_2F$), 95.7 (t, 1C, $CF_3CF_2CF_2O\mathbf{C}H_2F$, $^1J(^{13}C, ^1H)$ = 180.6 Hz) ppm.**^{17}O NMR** (81.4 MHz, $CDCl_3$): δ = 102.2 (s, 1O, $CF_3CF_2CF_2\mathbf{O}CH_2F$) ppm.

^{19}F NMR (564.7 MHz, $CDCl_3$): δ = −81.6 (t, 3F, $C\mathbf{F}_3CF_2CF_2OCH_2F$, $^3J(^{19}F, ^{19}F)$ = 7.3 Hz), −86.9 (m, 2F, $CF_3CF_2C\mathbf{F}_2OCH_2F$), −130.1 (m, 2F; $CF_3C\mathbf{F}_2CF_2OCH_2F$), −154.5 (tt, 1F, $CF_3CF_2CF_2OCH_2\mathbf{F}$, $^2J(^{19}F, ^1H)$ = 50.8 Hz, $^3J(^{19}F, ^{19}F)$ = 9.4 Hz) ppm.

^{19}F{^{1}H} NMR (564.7 MHz, $CDCl_3$): δ = −81.6 (t, 3F, $C\mathbf{F}_3CF_2CF_2OCH_2F$, $^3J(^{19}F, ^{19}F)$ = 7.5 Hz), −86.9 (m, 2F, $CF_3CF_2C\mathbf{F}_2OCH_2F$), −130.1 (m, 2F; $CF_3C\mathbf{F}_2CF_2OCH_2F$), −154.5 (t, 1F, $CF_3CF_2CF_2OCH_2\mathbf{F}$, $^3J(^{19}F, ^{19}F)$ = 9.3 Hz) ppm.

GC-MS [t = 0.45 min] m/z$^+$: 219 $[M]^+$, 199 [M −F], 181 [HM −F_2], 169 [M −OCH_2F], 131 [HM −CF_4], 119 [M −CF_2OCH_2F], 100 [M −CF_3OCH_2F], 81 [M −CF_4OCH_2F], 69 [M −$CF_2CF_2OCH_2F$].

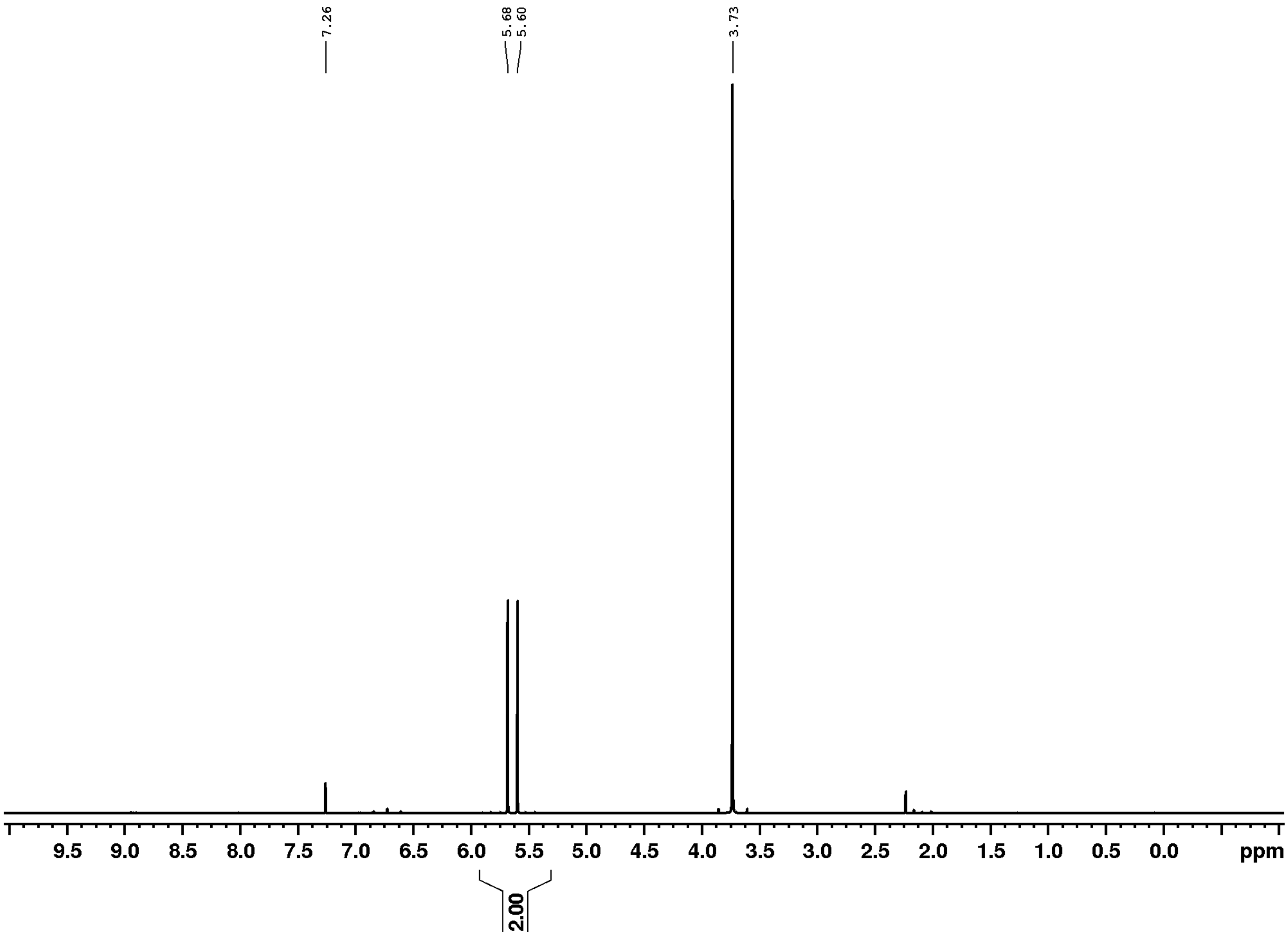

Figure S63. ^{1}H NMR of *n*-$C_3F_7OCH_2F$ (mixture with *n*-$C_3F_7OCH_3$) in $CDCl_3$.

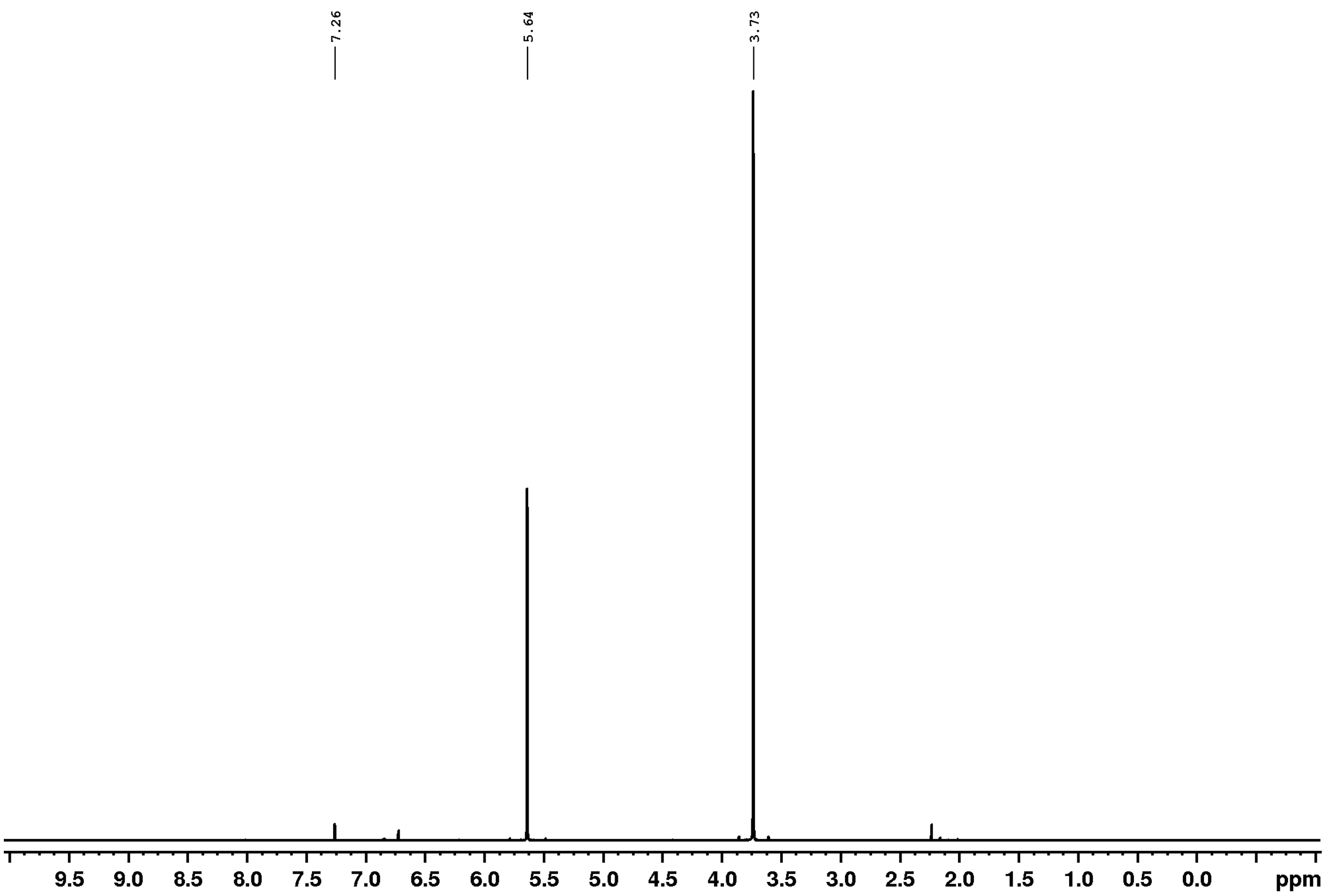

Figure S64. $^{1}H\{^{19}F\}$ NMR of *n*-$C_3F_7OCH_2F$ (mixture with *n*-$C_3F_7OCH_3$) in $CDCl_3$.

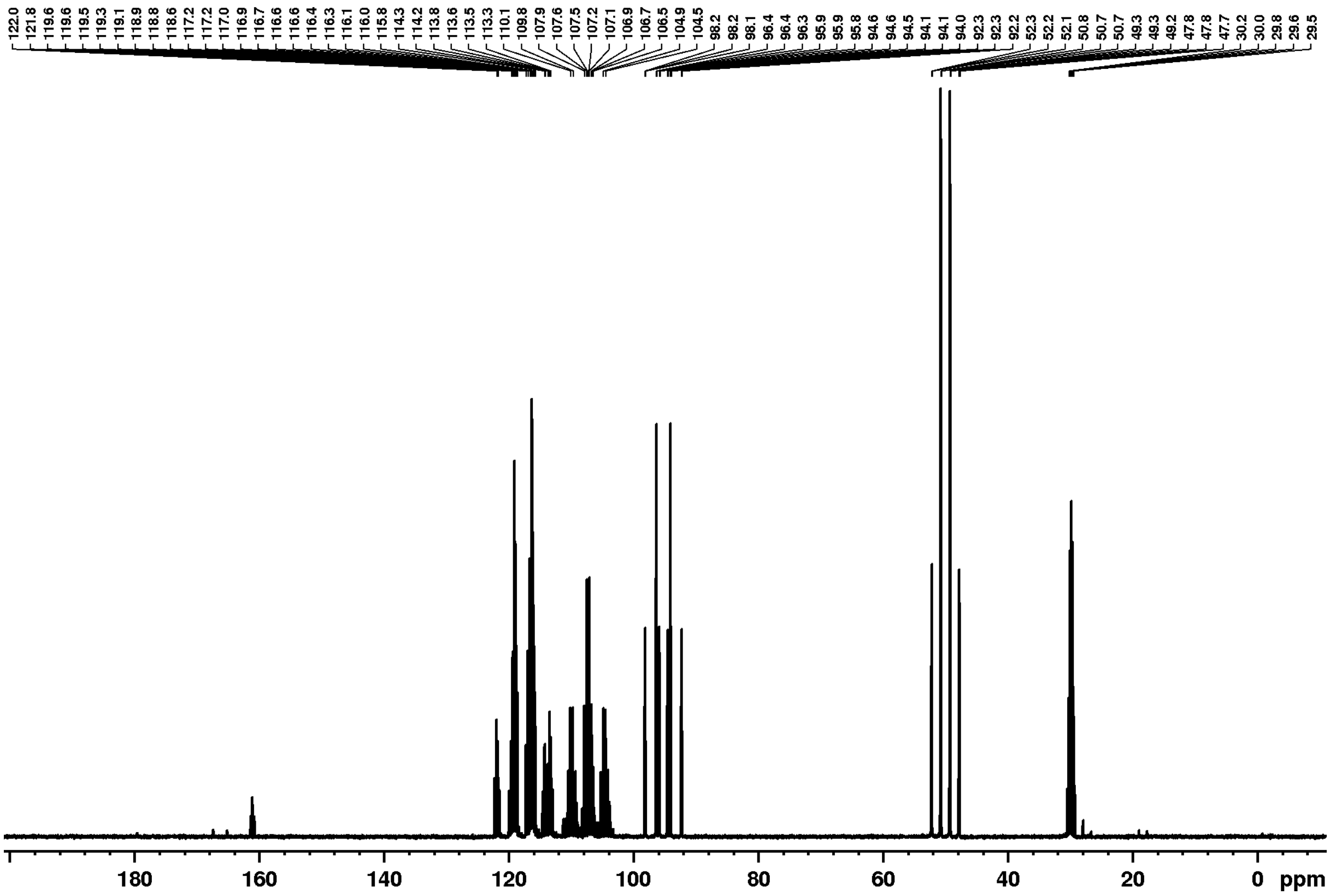

Figure S65. ^{13}C NMR of n-$C_3F_7OCH_2F$ (mixture with n-$C_3F_7OCH_3$) measured as neat substance with acetone-d_6 and CCl_3F as external standard.

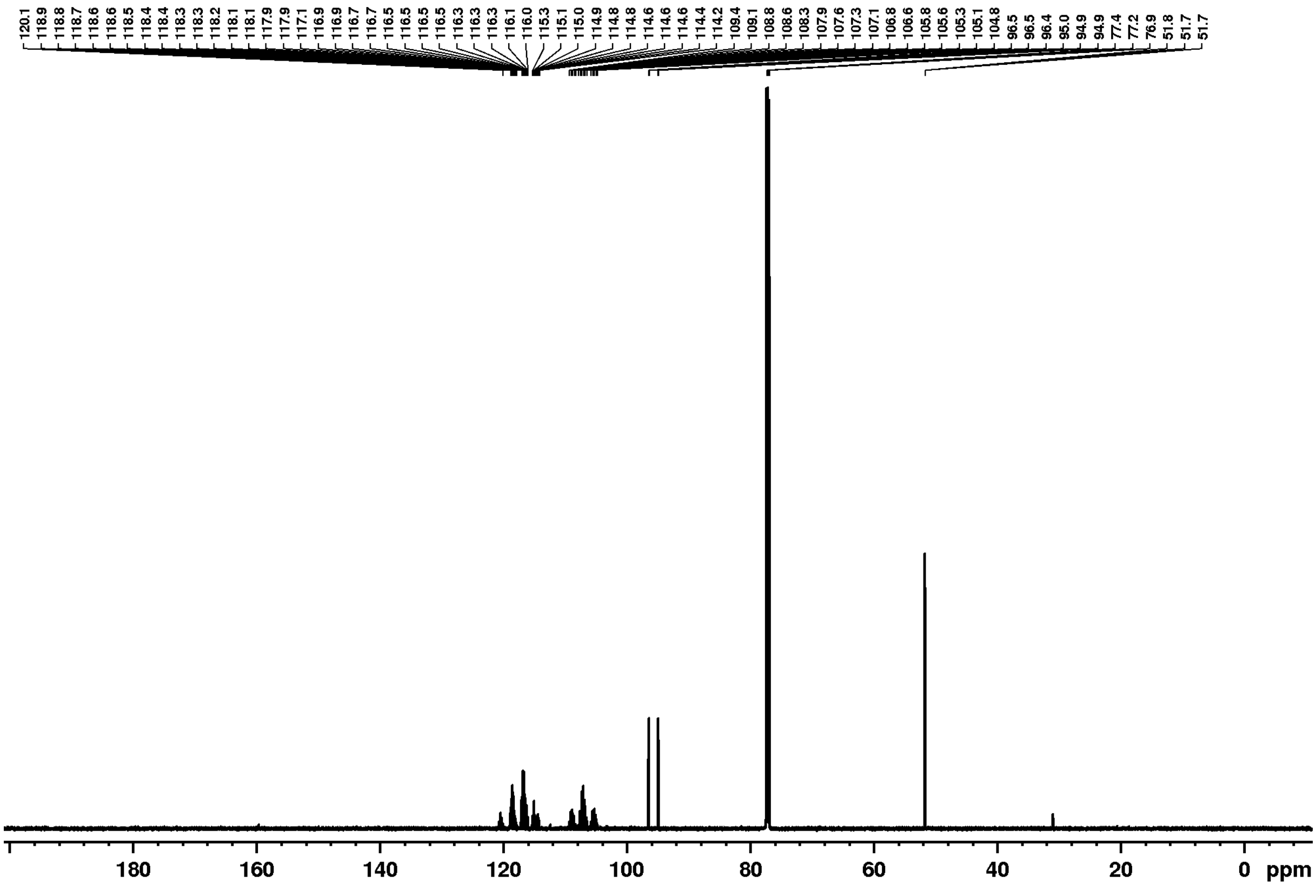

Figure S66. $^{13}C\{^1H\}$ NMR of n-$C_3F_7OCH_2F$ (mixture with n-$C_3F_7OCH_3$) in $CDCl_3$.

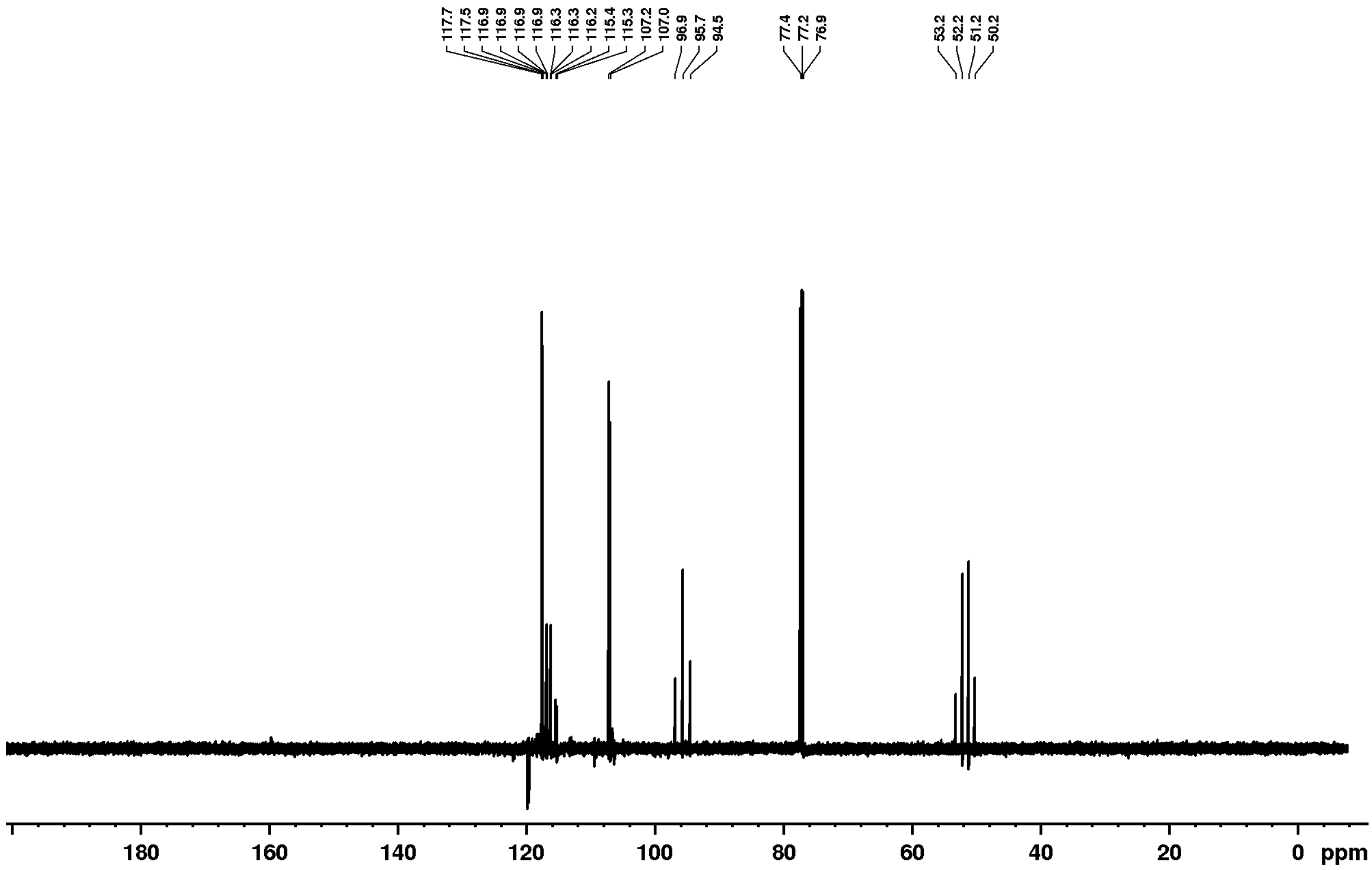

Figure S67. $^{13}C\{^{19}F\}$ NMR of *n*-$C_3F_7OCH_2F$ (mixture with *n*-$C_3F_7OCH_3$) in $CDCl_3$.

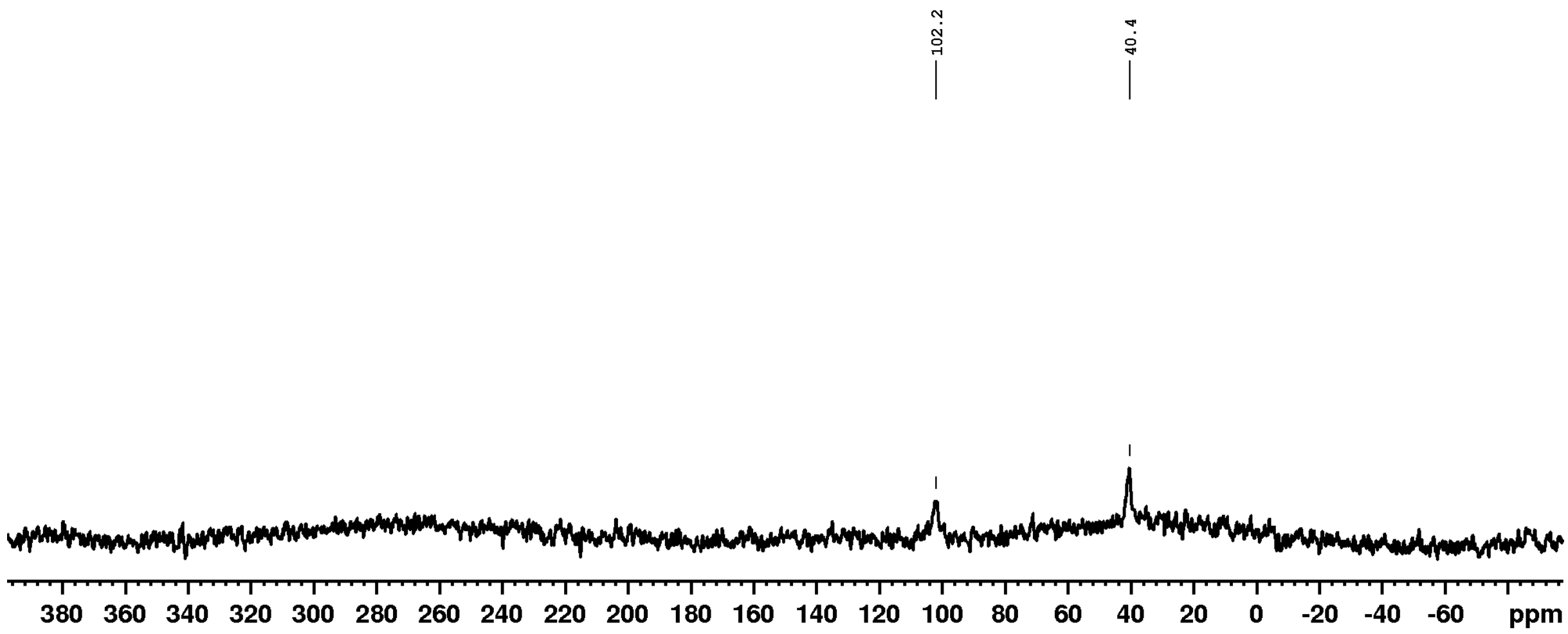

Figure S68. ^{17}O NMR of *n*-$C_3F_7OCH_2F$ (mixture with *n*-$C_3F_7OCH_3$) in $CDCl_3$.

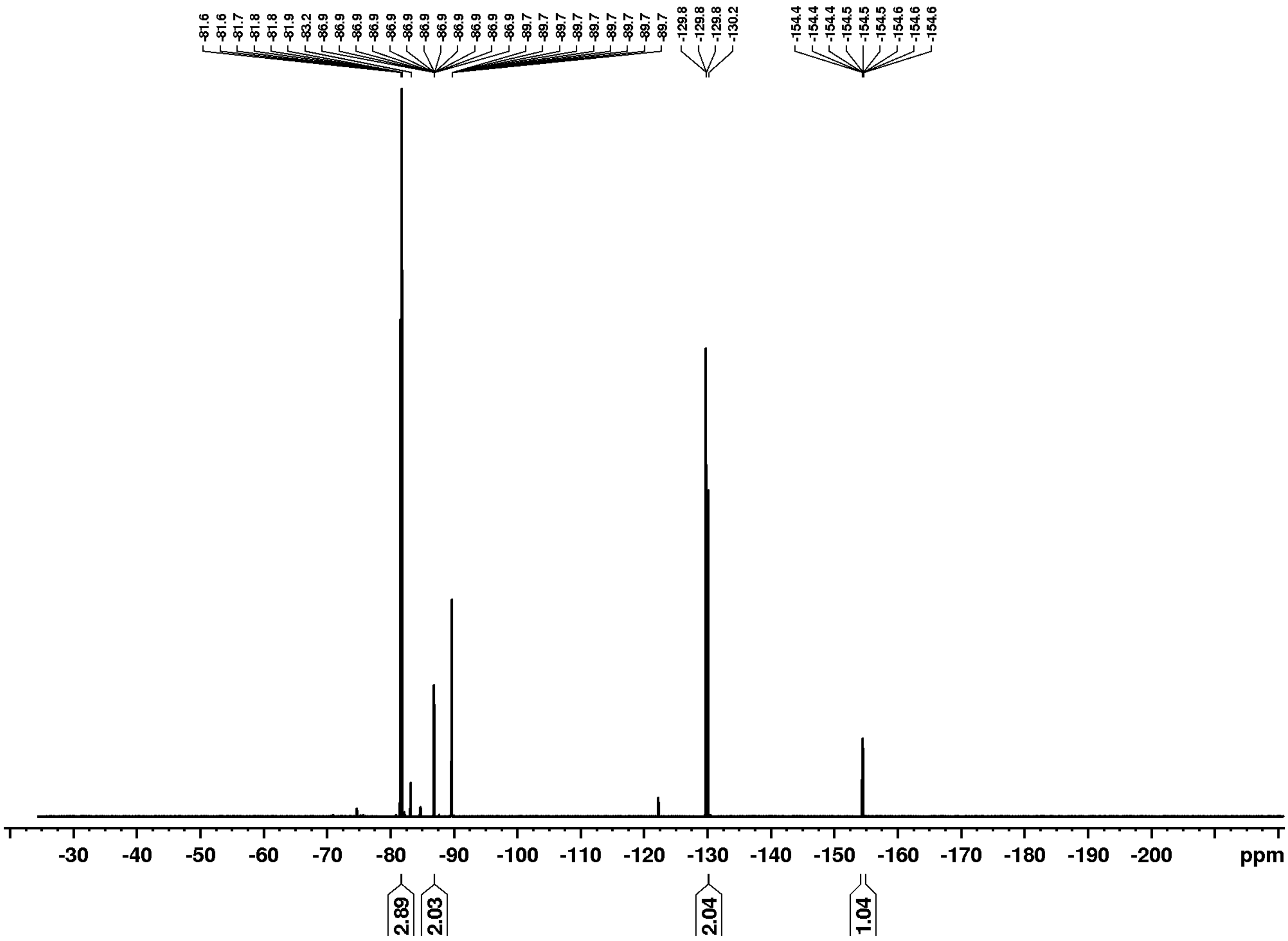

Figure S69. ^{19}F NMR of *n*-$C_3F_7OCH_2F$ (mixture with *n*-$C_3F_7OCH_3$) in $CDCl_3$.

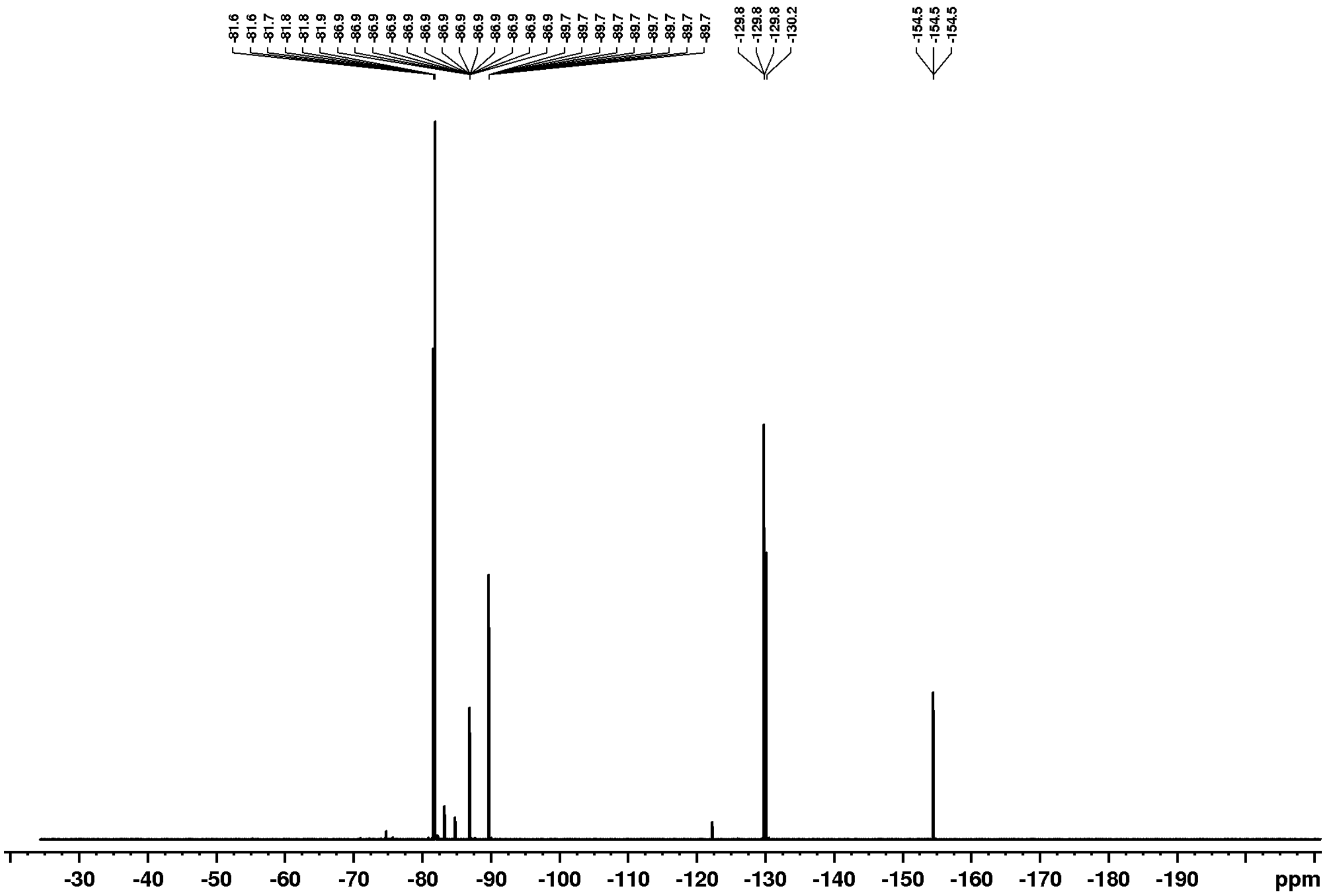

Figure S70. $^{19}F\{^1H\}$ NMR of *n*-$C_3F_7OCH_2F$ (mixture with *n*-$C_3F_7OCH_3$) in $CDCl_3$.

***n*-$C_3F_7OCHF_2$**

Boiling point (distillation): T_{bp} = 23 °C at 980 mbar.

Boiling point (vapor pressure): T_{bp} = 23.5 °C at 1013 mbar (Antoine parameter: A = 4.042; B = 993.94; C = −50.418 for equation: $p = 10^{(A-B/(T+C))}$).

Enthalpy of vaporization (T = 298.15 K): $\Delta_{vap}H$ = 27.6 kJ mol^{-1} (for equation: $\Delta_{vap}H = \ln(10)\cdot R\cdot T^2\cdot(B/(T+C)^2)/1000$).

^{1}H NMR (600.2 MHz, $CDCl_3$): δ = 6.73 (tm, 1H, $CF_3CF_2CF_2OC\mathbf{H}F_2$, $^2J(^{19}F, ^1H)$ = 69.7 Hz) ppm.

^{1}H{^{19}F} NMR (600.2 MHz, $CDCl_3$): δ = 6.73 (s, 1H, $CF_3CF_2CF_2OC\mathbf{H}F_2$) ppm.

^{13}C NMR (150.9 MHz, $CDCl_3$): δ = 117.2 (qt, 1C, $\mathbf{C}F_3CF_2CF_2OCHF_2$, $^1J(^{19}F, ^{13}C)$ = 286.9 Hz, $^2J(^{19}F, ^{13}C)$ = 32.9 Hz), 115.1 (ttm, 1C, $CF_3CF_2\mathbf{C}F_2OCHF_2$, $^1J(^{19}F, ^{13}C)$ = 281.3 Hz, $^2J(^{19}F, ^{13}C)$ = 29.1 Hz), 112.4 (tdt, 1C, $CF_3CF_2CF_2O\mathbf{C}HF_2$, $^1J(^{19}F, ^{13}C)$ = 266.5 $^1J(^{13}C, ^1H)$ = 226.0 Hz, $^3J(^{19}F, ^{13}C)$ = 5.8 Hz), 106.6 (tm, 1C, $CF_3\mathbf{C}F_2CF_2OCHF_2$, $^1J(^{19}F, ^{13}C)$ = 267.3 Hz) ppm.

^{13}C{^{1}H} NMR (150.9 MHz, $CDCl_3$): δ = 117.2 (qt, 1C, $\mathbf{C}F_3CF_2CF_2OCHF_2$, $^1J(^{19}F, ^{13}C)$ = 286.9 Hz, $^2J(^{19}F, ^{13}C)$ = 32.9 Hz), 115.1 (ttm, 1C, $CF_3CF_2\mathbf{C}F_2OCHF_2$, $^1J(^{19}F, ^{13}C)$ = 281.3 Hz, $^2J(^{19}F, ^{13}C)$ = 29.1 Hz), 112.4 (tt, 1C, $CF_3CF_2CF_2O\mathbf{C}HF_2$, $^1J(^{19}F, ^{13}C)$ = 266.5 $^3J(^{19}F, ^{13}C)$ = 5.8 Hz), 106.6 (tm, 1C, $CF_3\mathbf{C}F_2CF_2OCHF_2$, $^1J(^{19}F, ^{13}C)$ = 267.3 Hz) ppm.**^{13}C{^{19}F} NMR** (150.9 MHz, $CDCl_3$): δ = 117.2 (s, 1C, $\mathbf{C}F_3CF_2CF_2OCHF_2$), 115.1 (s, 1C, $CF_3CF_2\mathbf{C}F_2OCHF_2$), 112.4 (d, 1C, $CF_3CF_2CF_2O\mathbf{C}HF_2$, $^1J(^{13}C, ^1H)$ = 226.0 Hz), 106.6 (s, 1C, $CF_3\mathbf{C}F_2CF_2OCHF_2$) ppm.**^{17}O NMR** (81.4 MHz, $CDCl_3$): δ = 126.2 (s, 1O, $CF_3CF_2CF_2\mathbf{O}CHF_2$) ppm.

^{19}F NMR (564.7 MHz, $CDCl_3$): δ = −81.5 (t, 3F, $C\mathbf{F}_3CF_2CF_2OCHF_2$, $^3J(^{19}F, ^{19}F)$ = 7.3 Hz), −84.7 (dt, 2F, $CF_3CF_2CF_2OCH\mathbf{F}_2$, $^2J(^{19}F, ^1H)$ = 69.8 Hz, $^4J(^{19}F, ^{19}F)$ = 4.7 Hz), −84.8 (m, 2F, $CF_3C\mathbf{F}_2CF_2OCHF_2$), −130.1 (m, 2F, $CF_3CF_2C\mathbf{F}_2OCHF_2$) ppm.

^{19}F{^{1}H} NMR (564.7 MHz, $CDCl_3$): δ = −81.5 (t, 3F, $C\mathbf{F}_3CF_2CF_2OCHF_2$, $^3J(^{19}F, ^{19}F)$ = 7.3 Hz), −84.7 (t, 2F, $CF_3CF_2CF_2OCH\mathbf{F}_2$, $^4J(^{19}F, ^{19}F)$ = 4.7 Hz), −84.8 (m, 2F, $CF_3C\mathbf{F}_2CF_2OCHF_2$), −130.1 (m, 2F, $CF_3CF_2C\mathbf{F}_2OCHF_2$) ppm.

GC-MS [t = 0.45 min] m/z$^+$: 235 $[M]^+$, 169 [M −$OCHF_2$], 119 [M −CF_2OCHF_2], 117 [M −CF_3CF_2], 100 [M −CF_3OCF_3], 69 [M −$CF_2CF_2OCHF_2$], 51 [M −$CF_3CF_2CF_2O$].

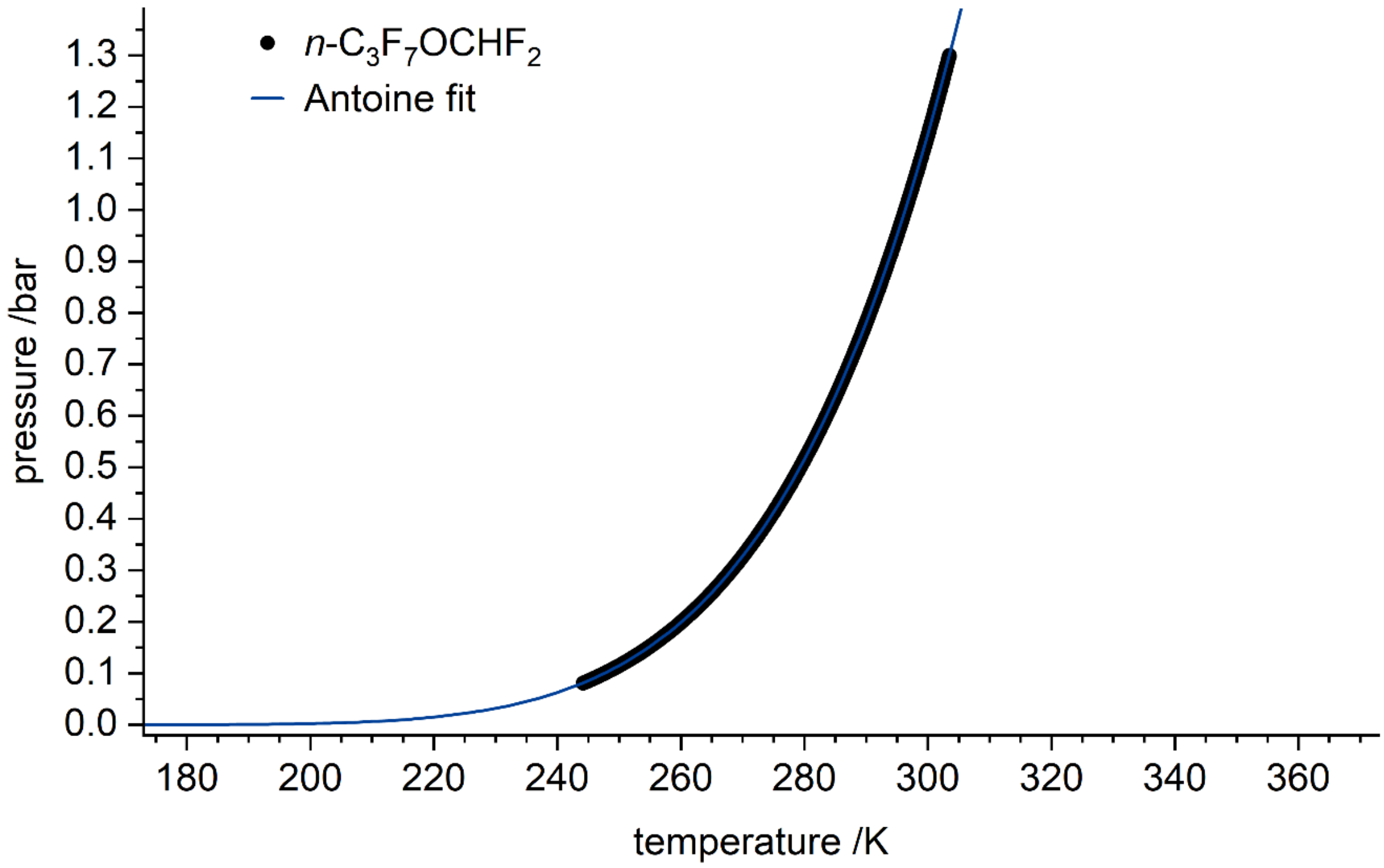

Figure S71. Vapor pressure measurement and Antoine fit of $CF_3CF_2CF_2OCHF_2$ (Antoine parameter: A = 4.042; B = 993.94; C = −50.418 for equation: p = 10(A−B/(T+C))).

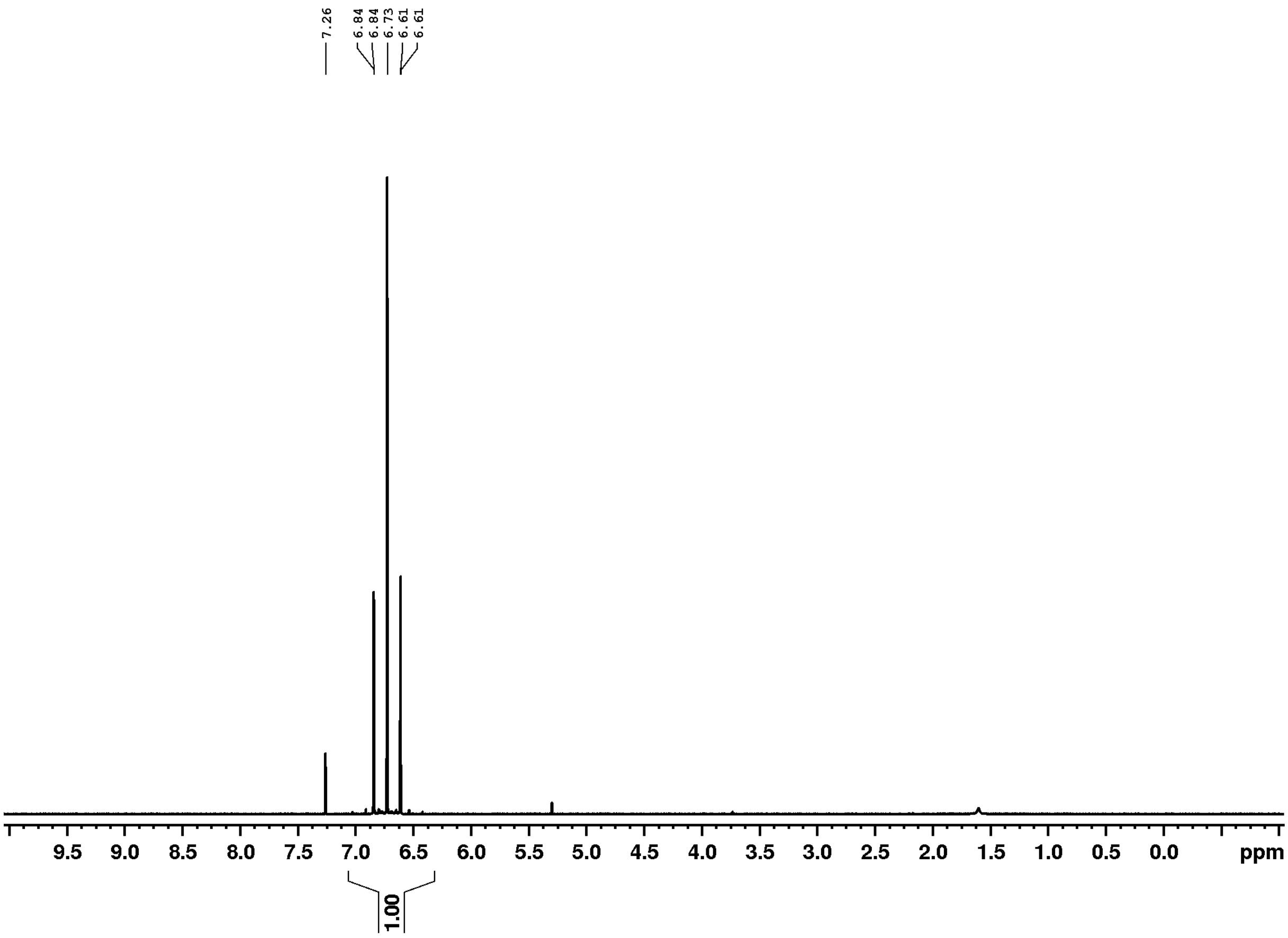

Figure S72. ^{1}H NMR of *n*-$C_3F_7OCHF_2$ in $CDCl_3$.

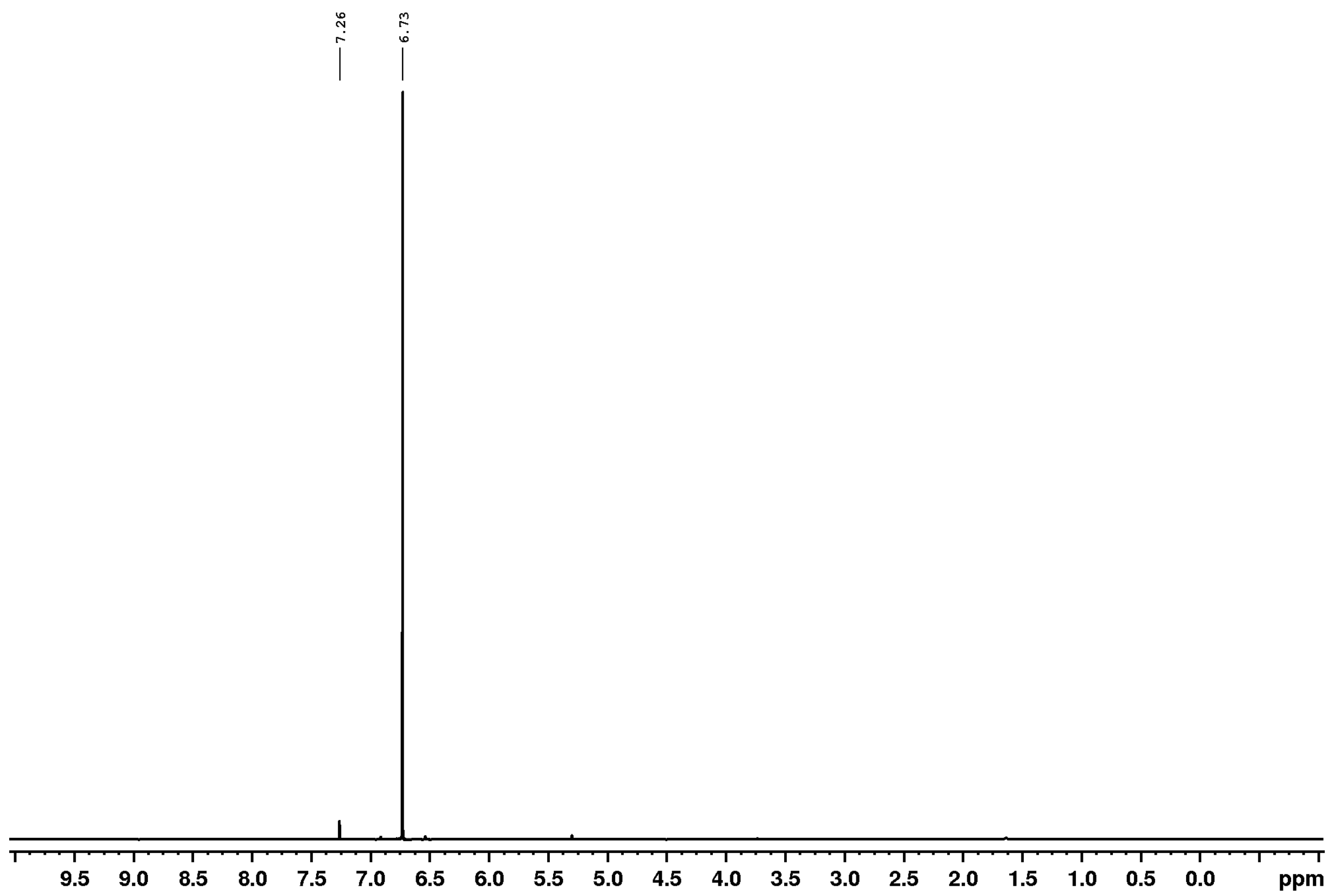

Figure S73. $^{1}H\{^{19}F\}$ NMR of *n*-$C_3F_7OCHF_2$ in $CDCl_3$.

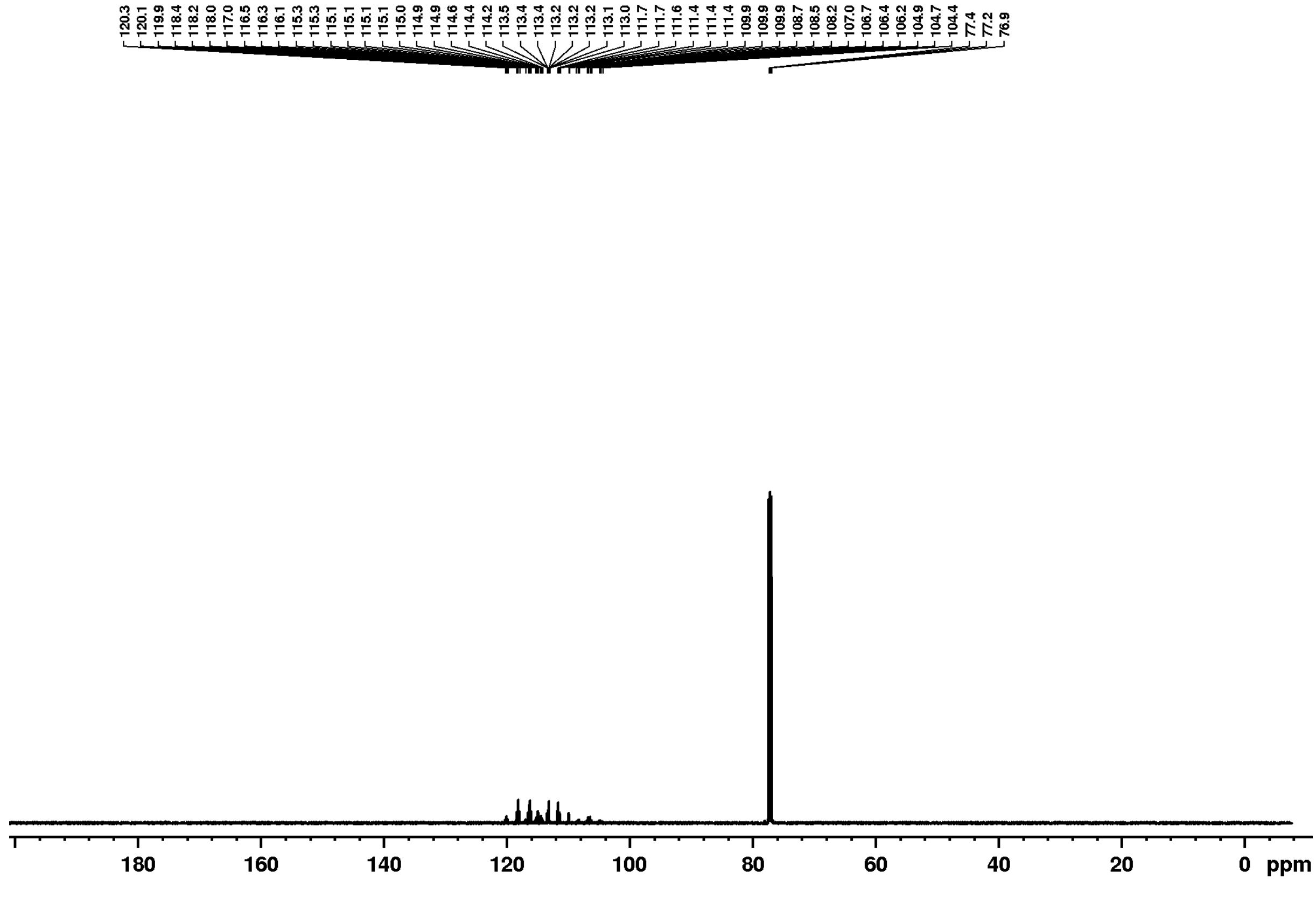

Figure S74. ^{13}C NMR of *n*-$C_3F_7OCHF_2$ in $CDCl_3$.

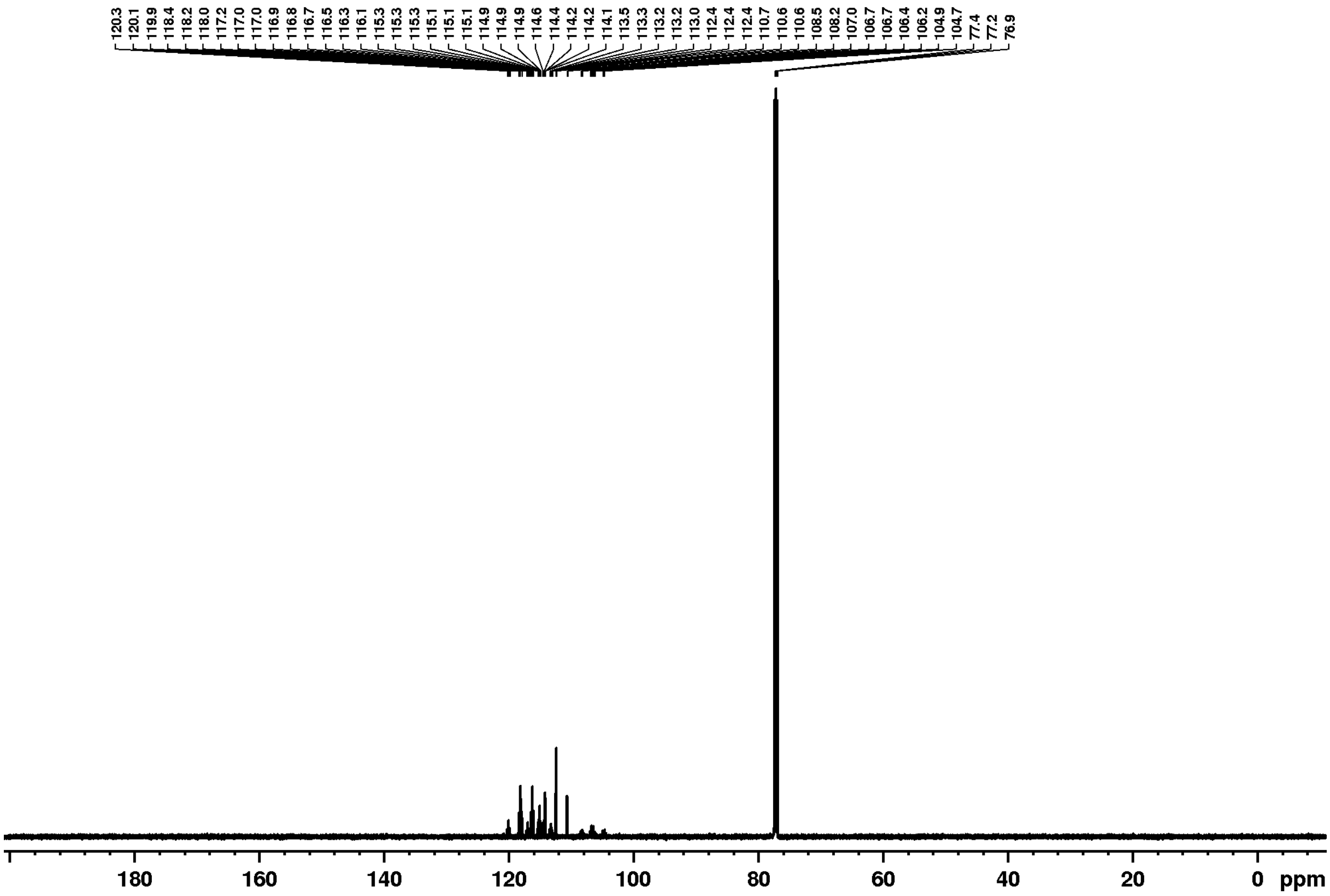

Figure S75. $^{13}C\{^{1}H\}$ NMR of n-$C_3F_7OCHF_2$ in $CDCl_3$.

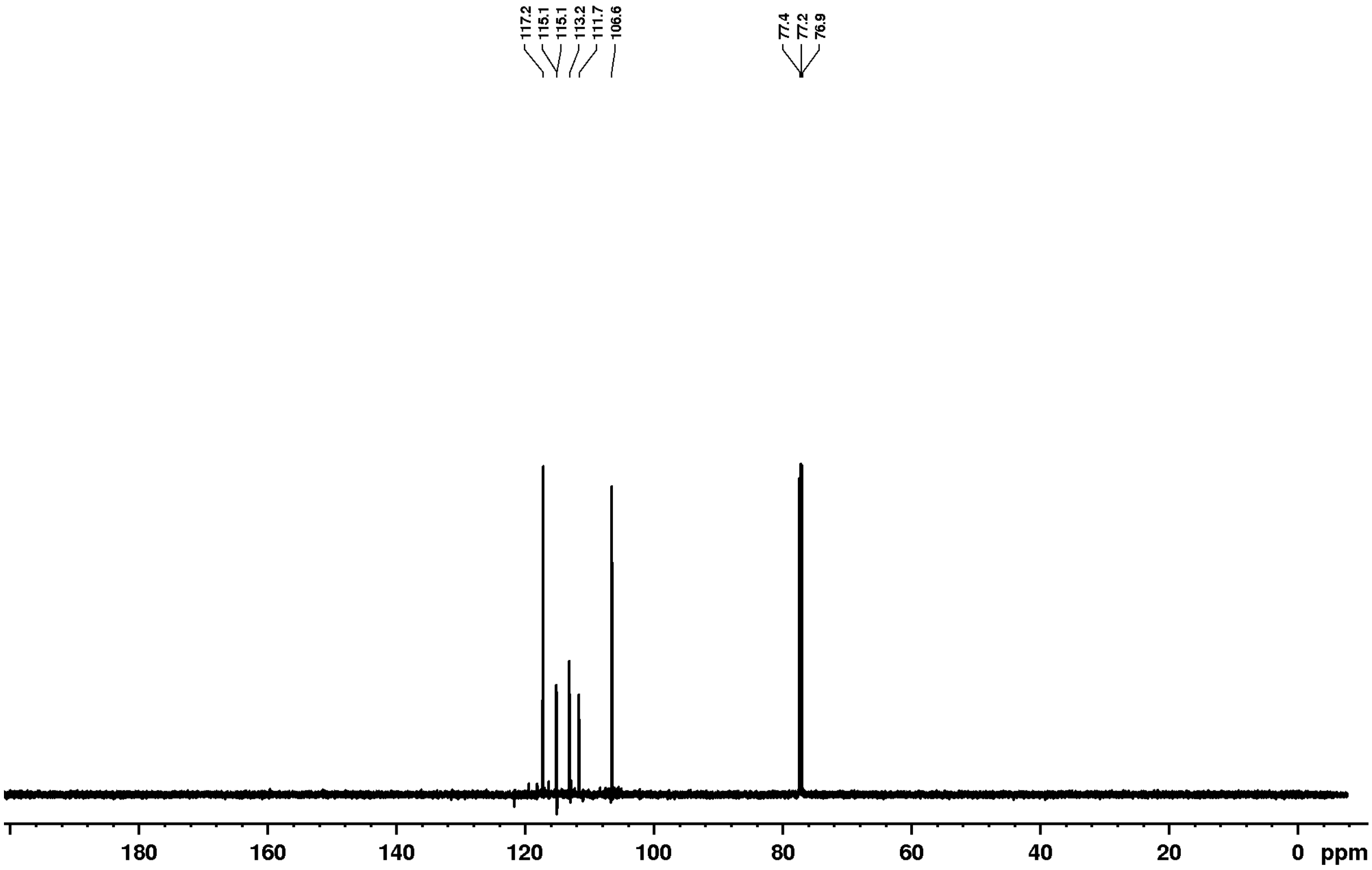

Figure S76. $^{13}C\{^{19}F\}$ NMR of n-$C_3F_7OCHF_2$ in $CDCl_3$.

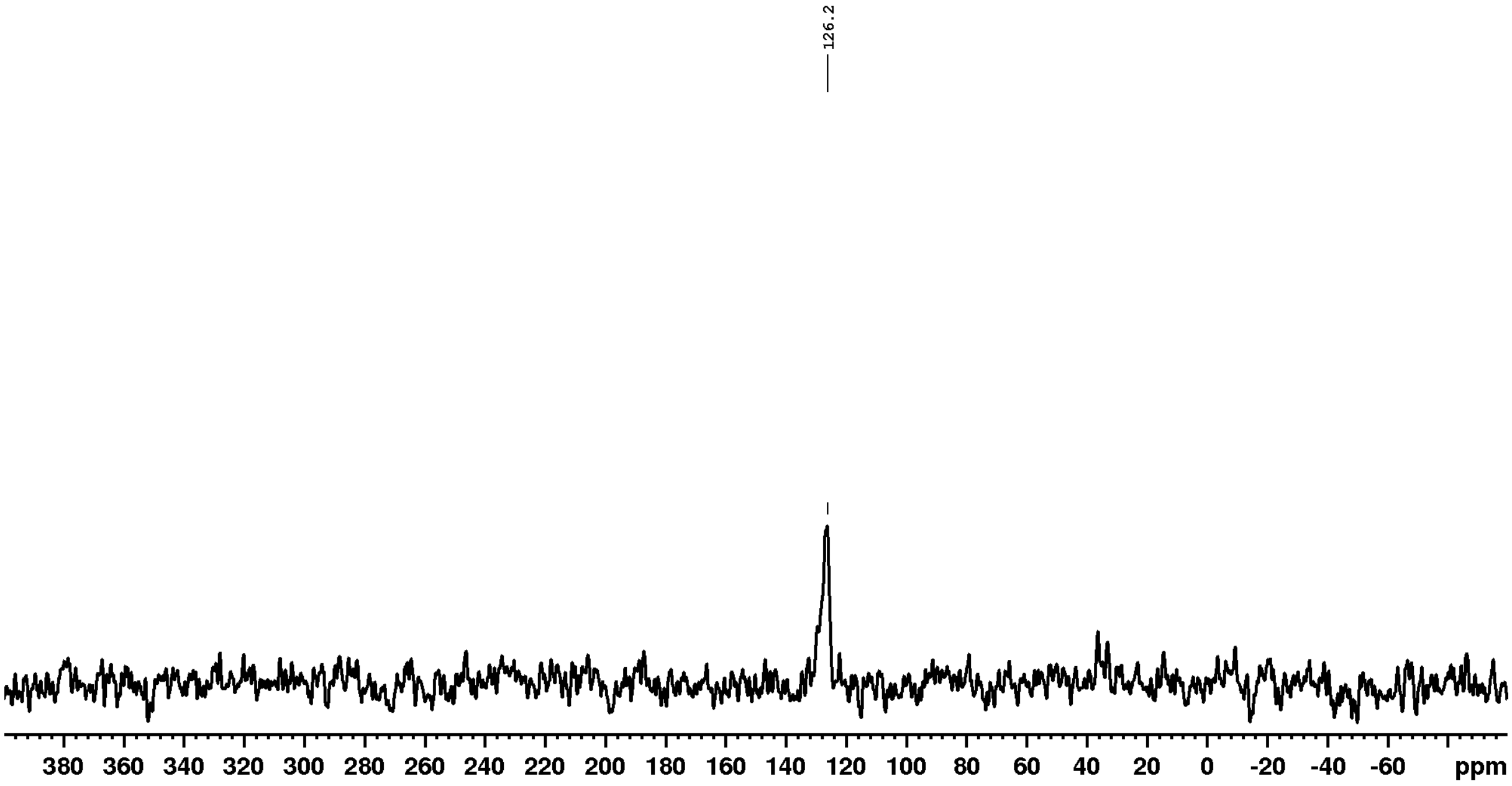

Figure S77. ^{17}O NMR of *n*-$C_3F_7OCHF_2$ in $CDCl_3$.

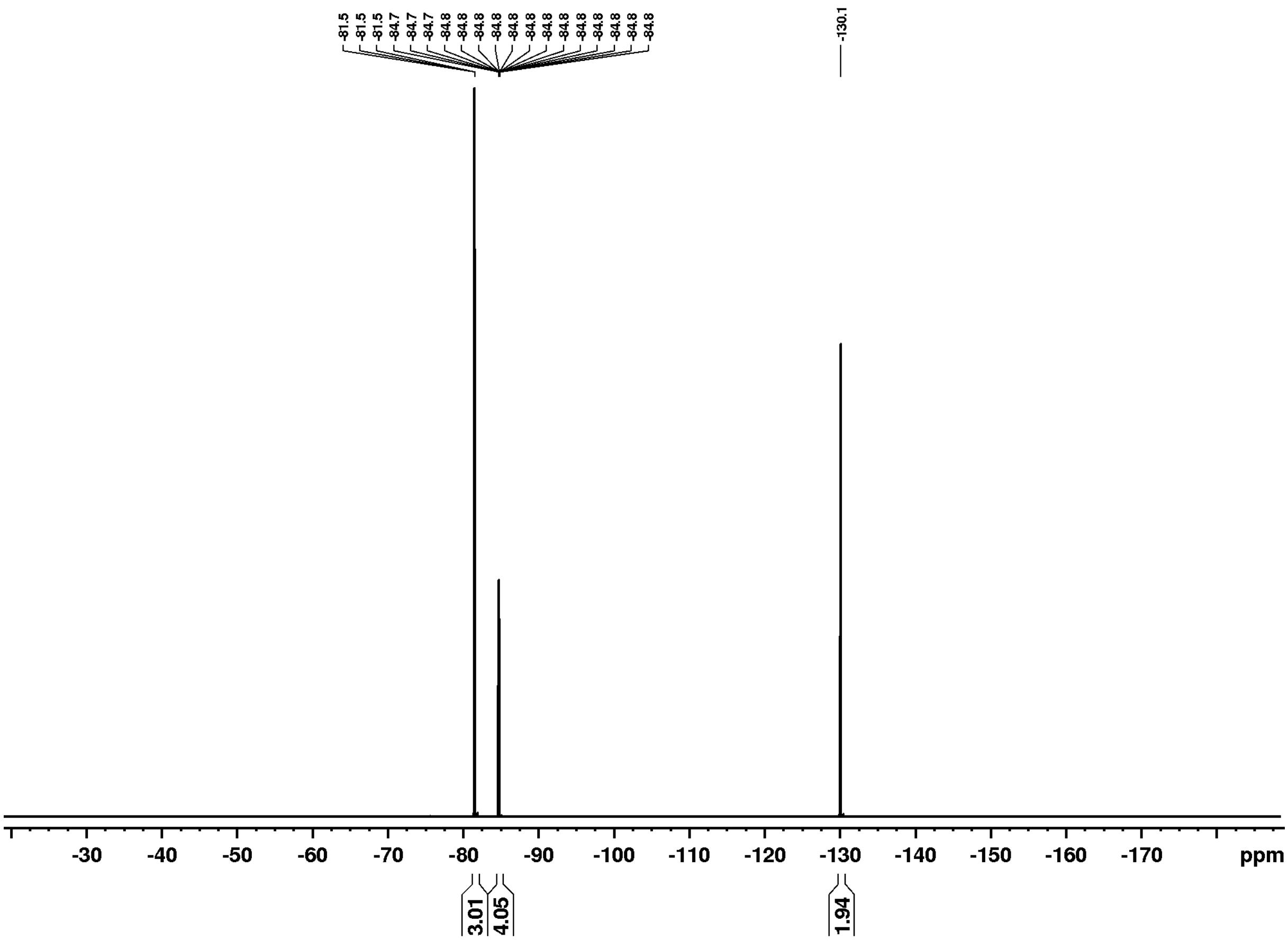

Figure S78. ^{19}F NMR of *n*-$C_3F_7OCHF_2$ in $CDCl_3$.

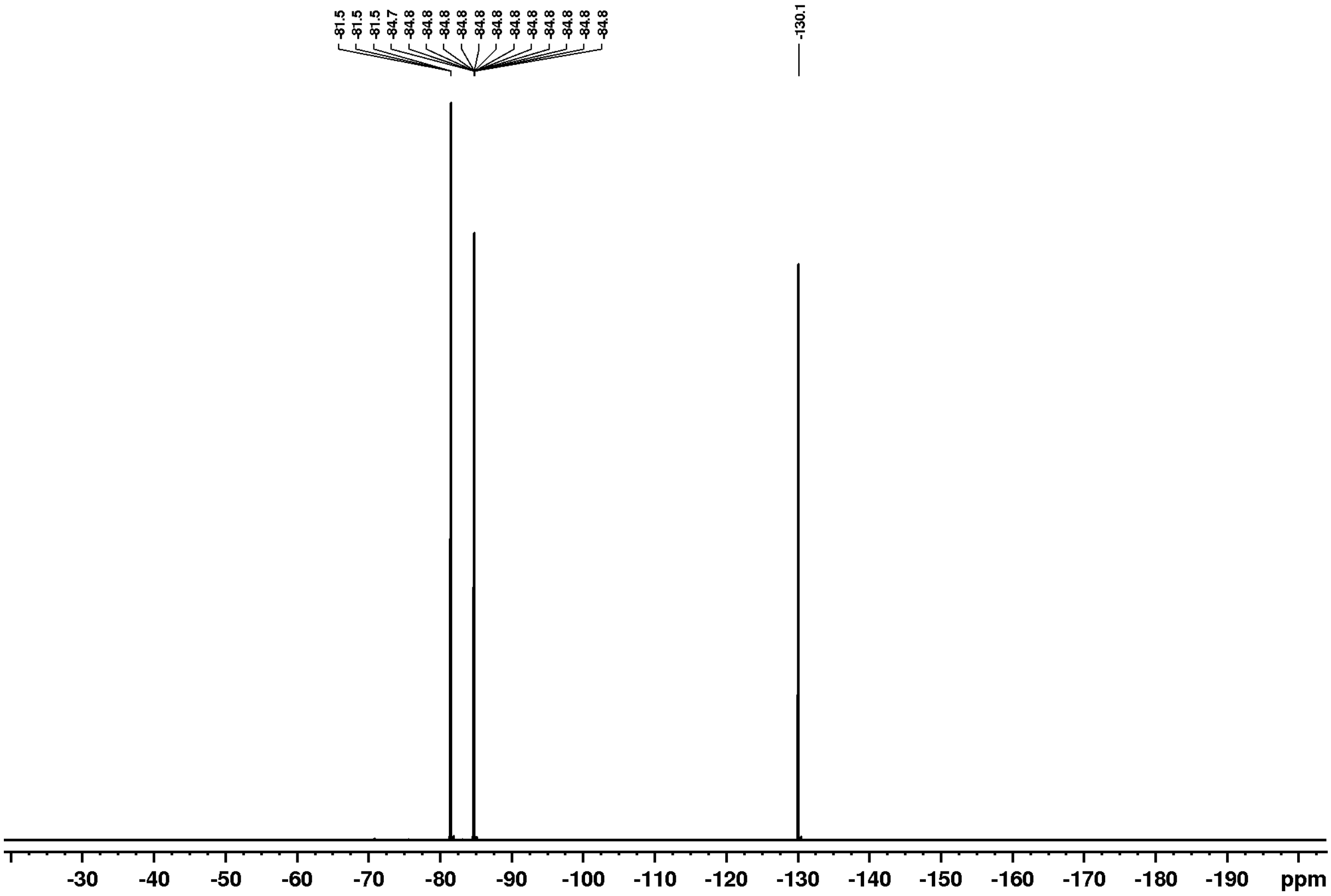

Figure S 79. $^{19}F\{^1H\}$ NMR of *n*-$C_3F_7OCHF_2$ in $CDCl_3$.

***n*-$C_3F_7OCF_3$**

Boiling point (distillation): T_{bp} = 13 °C at 1325 mbar.

Boiling point (vapor pressure): T_{bp} = 6.8 °C at 1013 mbar (Antoine parameter: A = 3.942; B = 900.81; C =−51.128 for equation: $p = 10^{(A-B/(T+C))}$).

Enthalpy of vaporization (T = 298.15 K): $\Delta_{vap}H$ = 25.1 kJ mol^{-1} (for equation: $\Delta_{vap}H = \ln(10)\cdot R\cdot T^2\cdot(B/(T+C)^2)/1000$).

$^{13}C\{^1H\}$ NMR (125.8.7 MHz, $CDCl_3$): δ = 119.3 (q, 1C, $CF_3CF_2CF_2O\mathbf{C}F_3$, $^1J(^{19}F, ^{13}C)$ = 269.4 Hz), 117.1 (qt, 1C, $\mathbf{C}F_3CF_2CF_2OCF_3$, $^1J(^{19}F, ^{13}C)$ = 287.0 Hz, $^2J(^{19}F, ^{13}C)$ = 32.8 Hz), 114.5 (ttq, 1C, $CF_3CF_2\mathbf{C}F_2OCF_3$, $^1J(^{19}F, ^{13}C)$ = 287.2 Hz, $^2J(^{19}F, ^{13}C)$ = 31.5 Hz, $^4J(^{19}F, ^{13}C)$ = 2.0 Hz), 106.5 (tm, 1C, $CF_3\mathbf{C}F_2CF_2OCF_3$, $^1J(^{19}F, ^{13}C)$ = 269.6 Hz) ppm.

$^{13}C\{^{19}F\}$ NMR (125.8 MHz, $CDCl_3$): δ = 119.3 (s, 1C, $CF_3CF_2CF_2O\mathbf{C}F_3$), 117.1 (s, 1C, $\mathbf{C}F_3CF_2CF_2OCF_3$), 114.5 (s, 1C, $CF_3CF_2\mathbf{C}F_2OCF_3$), 106.5 (s, 1C, $CF_3\mathbf{C}F_2CF_2OCF_3$) ppm.

^{17}O NMR (81.4 MHz, $CDCl_3$): δ = 125.8 (s, 1O, $CF_3CF_2CF_2\mathbf{O}CF_3$) ppm.

^{19}F NMR (470.5 MHz, $CDCl_3$): δ = −55.3 (t, 3F, $CF_3CF_2CF_2OC\mathbf{F_3}$, $^4J(^{19}F, ^{19}F)$ = 8.9 Hz), −81.5 (t, 3F, $C\mathbf{F_3}CF_2CF_2OCF_3$, $^3J(^{19}F, ^{19}F)$ = 7.4 Hz), −86.6 (m, 2F, $CF_3CF_2C\mathbf{F_2}OCF_3$), −130.0 (m, 2F, $CF_3C\mathbf{F_2}CF_2OCF_3$) ppm.

GC-MS [t = 0.44 min] m/z$^+$: 169 [M −OCF_3], 135 [M −CF_2CF_3], 119 [M −CF_2OCF_3], 100 [M −C_2F_6O], 69 [M −C_3F_7O].

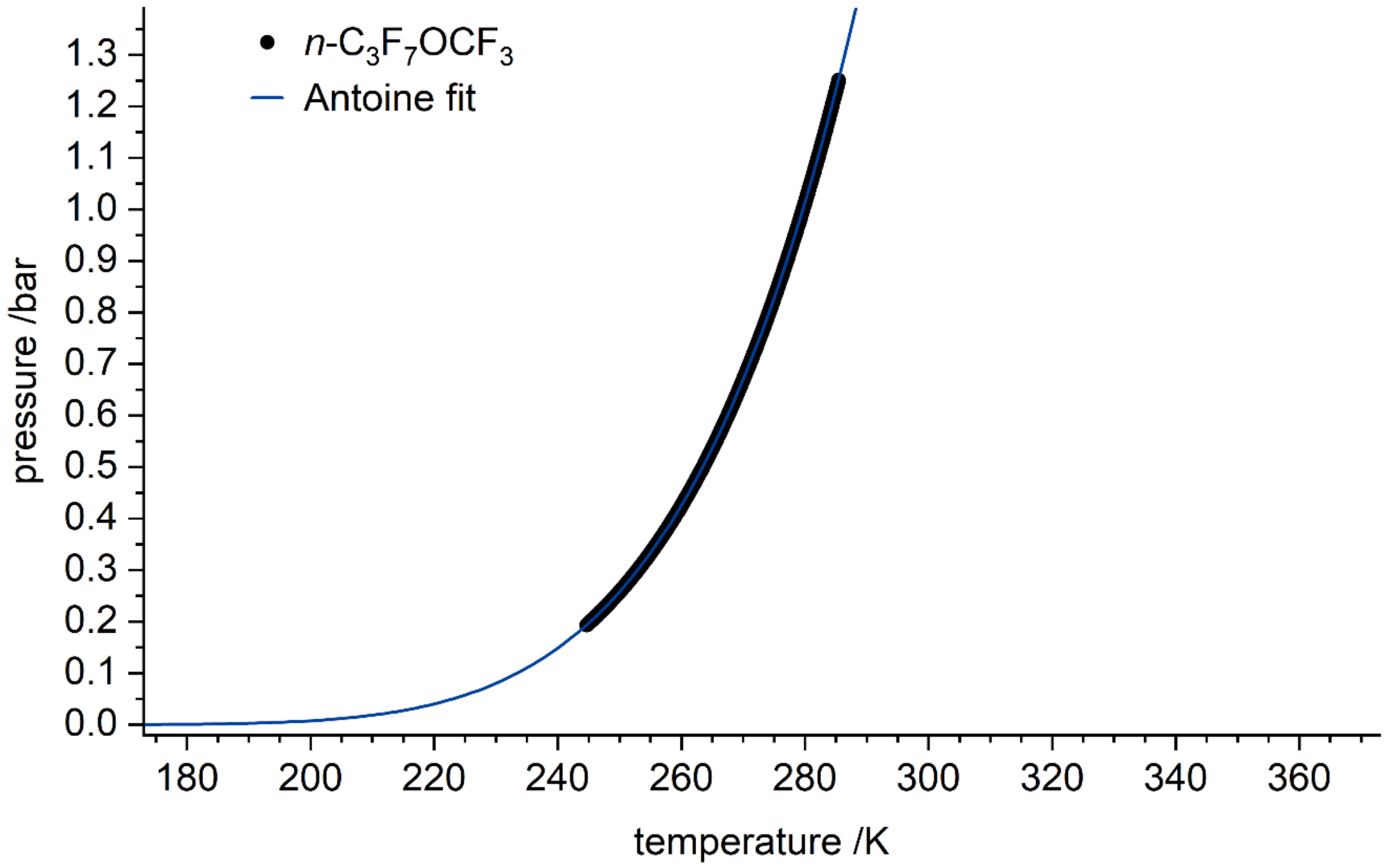

Figure S80. Vapor pressure measurement and Antoine fit of $CF_3CF_2CF_2OCF_3$ (Antoine parameter: A = 3.942; B = 900.81; C = −51.128 for equation: $p = 10^{(A-B/(T+C))}$).

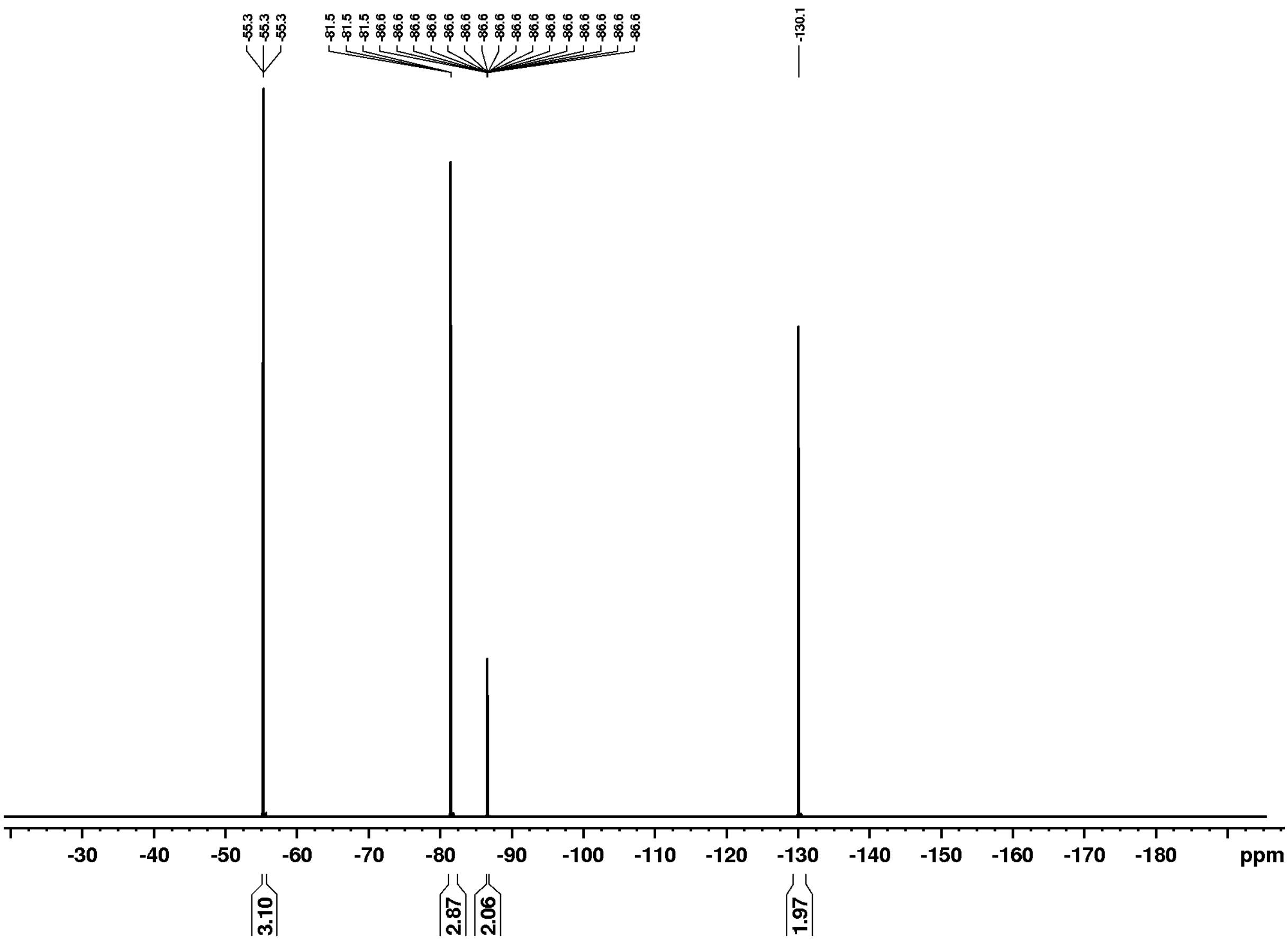

Figure S81. ^{19}F NMR of *n*-$C_3F_7OCF_3$ in $CDCl_3$.

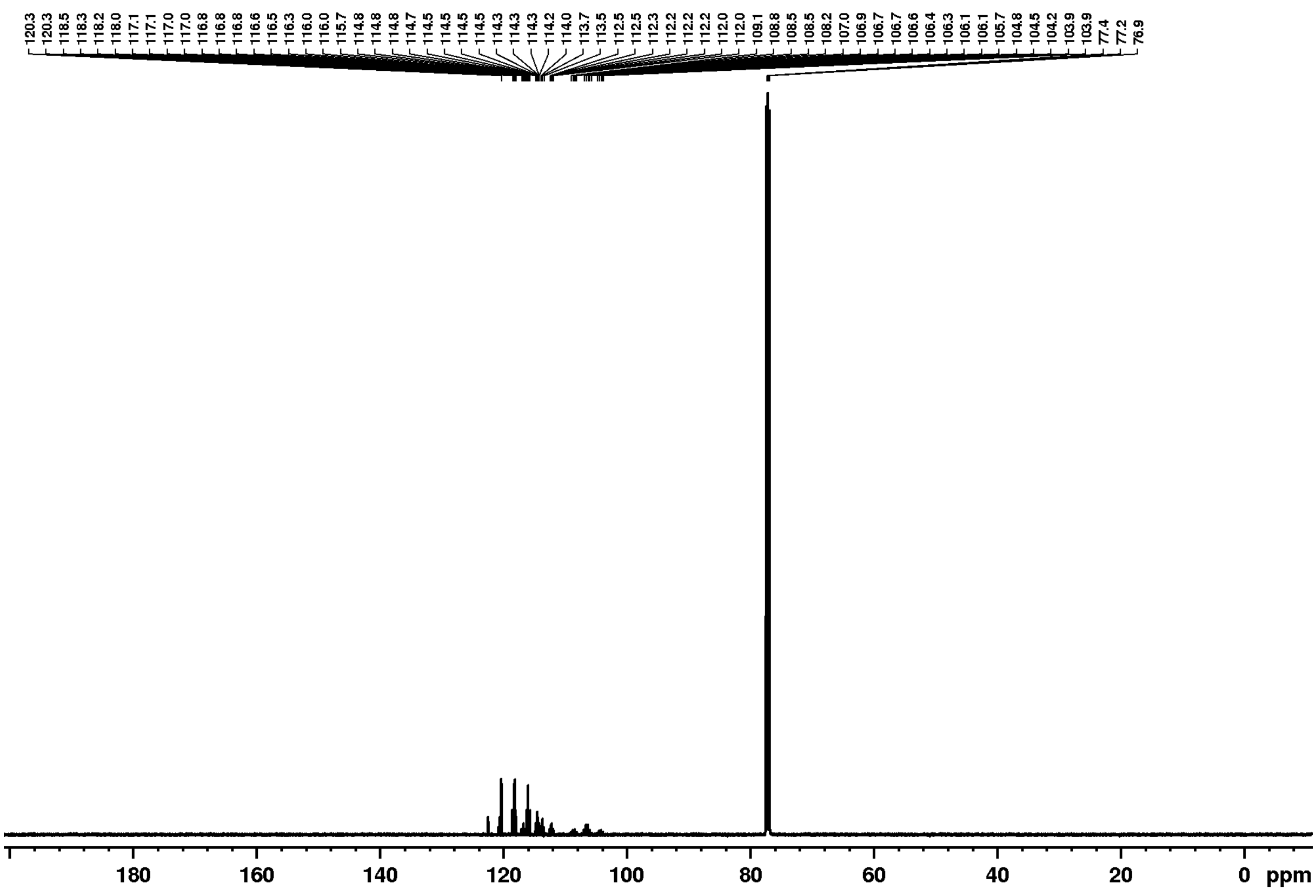

Figure S82. $^{13}C\{^1H\}$ NMR of *n*-$C_3F_7OCHF_2$ in $CDCl_3$.

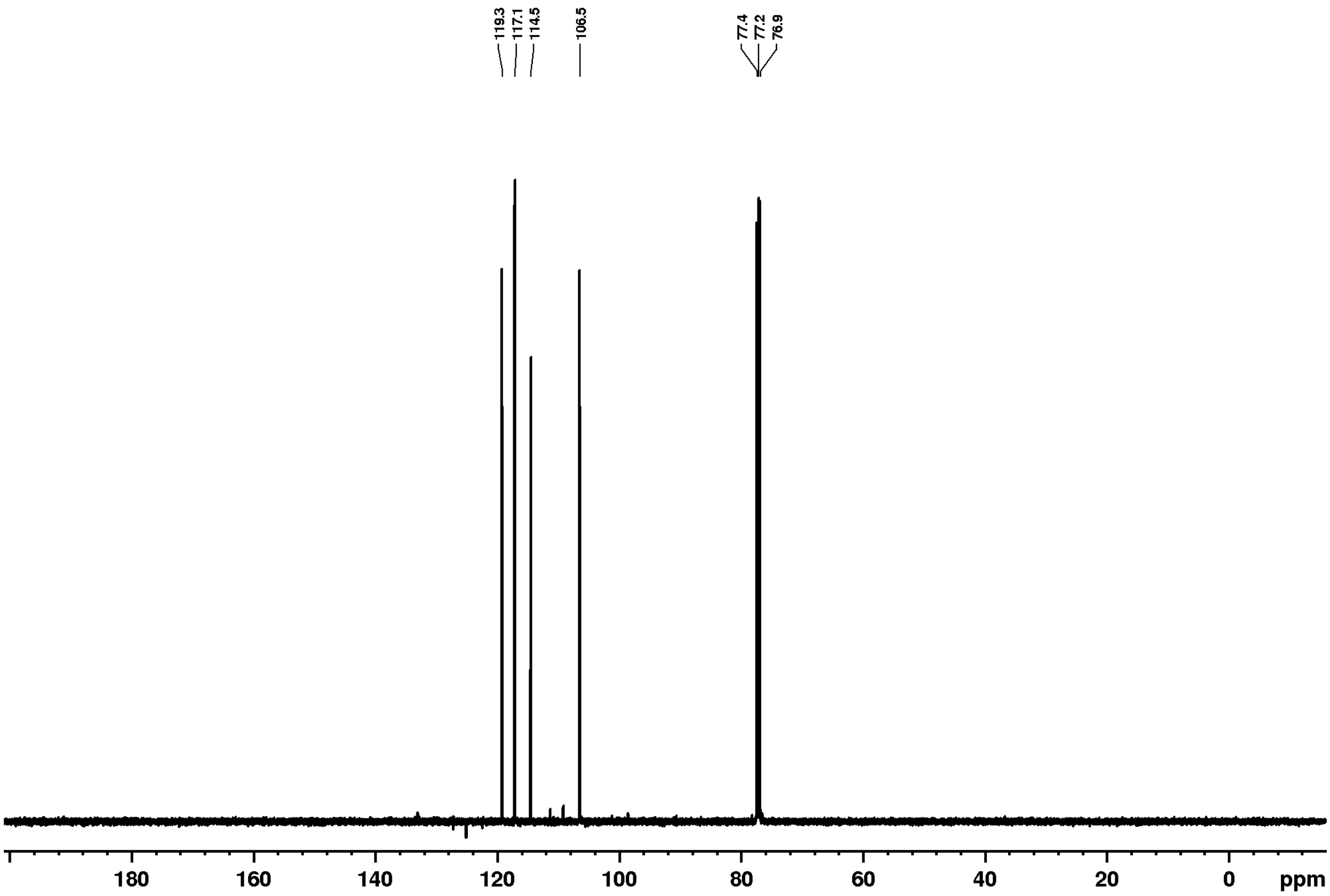

Figure S83. $^{13}C\{^{19}F\}$ NMR of n-$C_3F_7OCHF_2$ in $CDCl_3$.

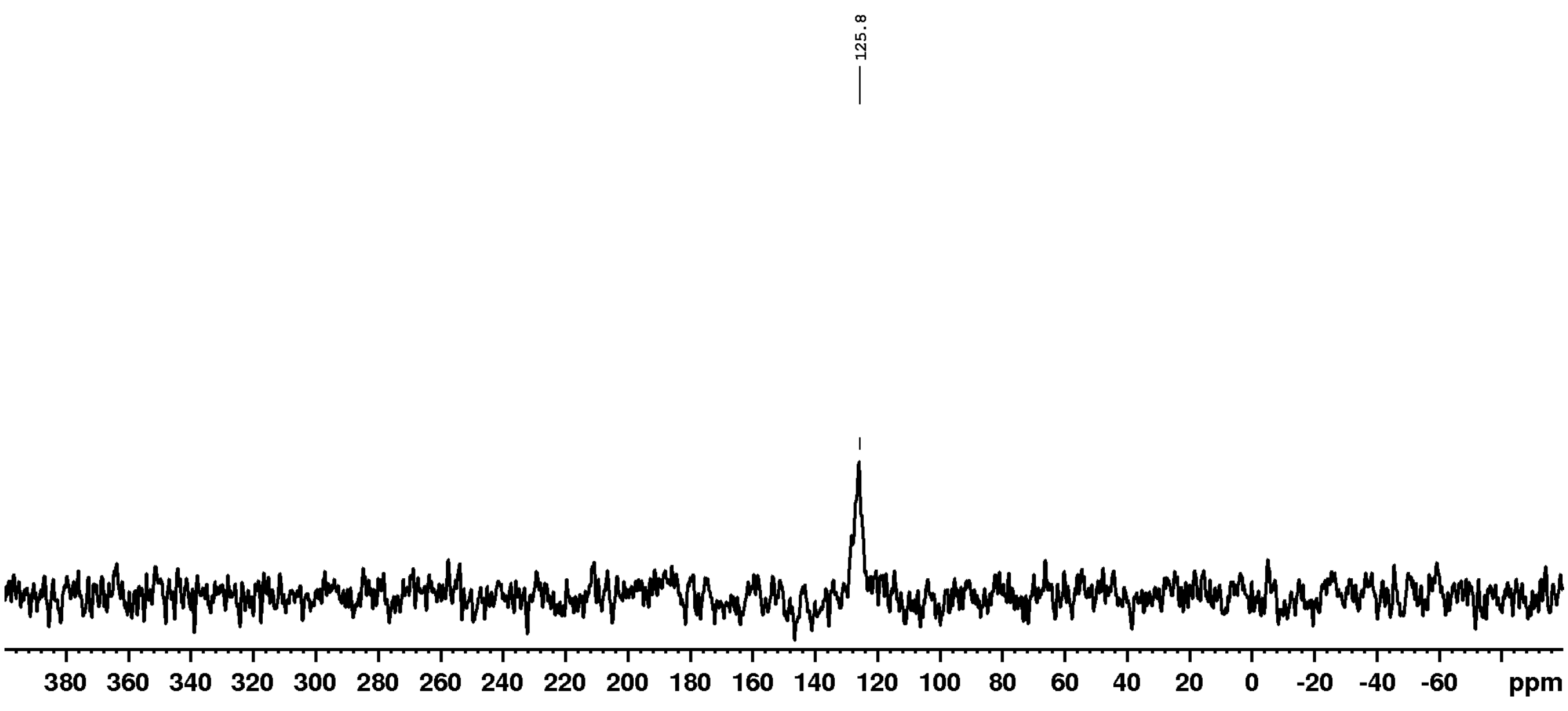

Figure S84. ^{17}O NMR of n-$C_3F_7OCF_3$ in $CDCl_3$.

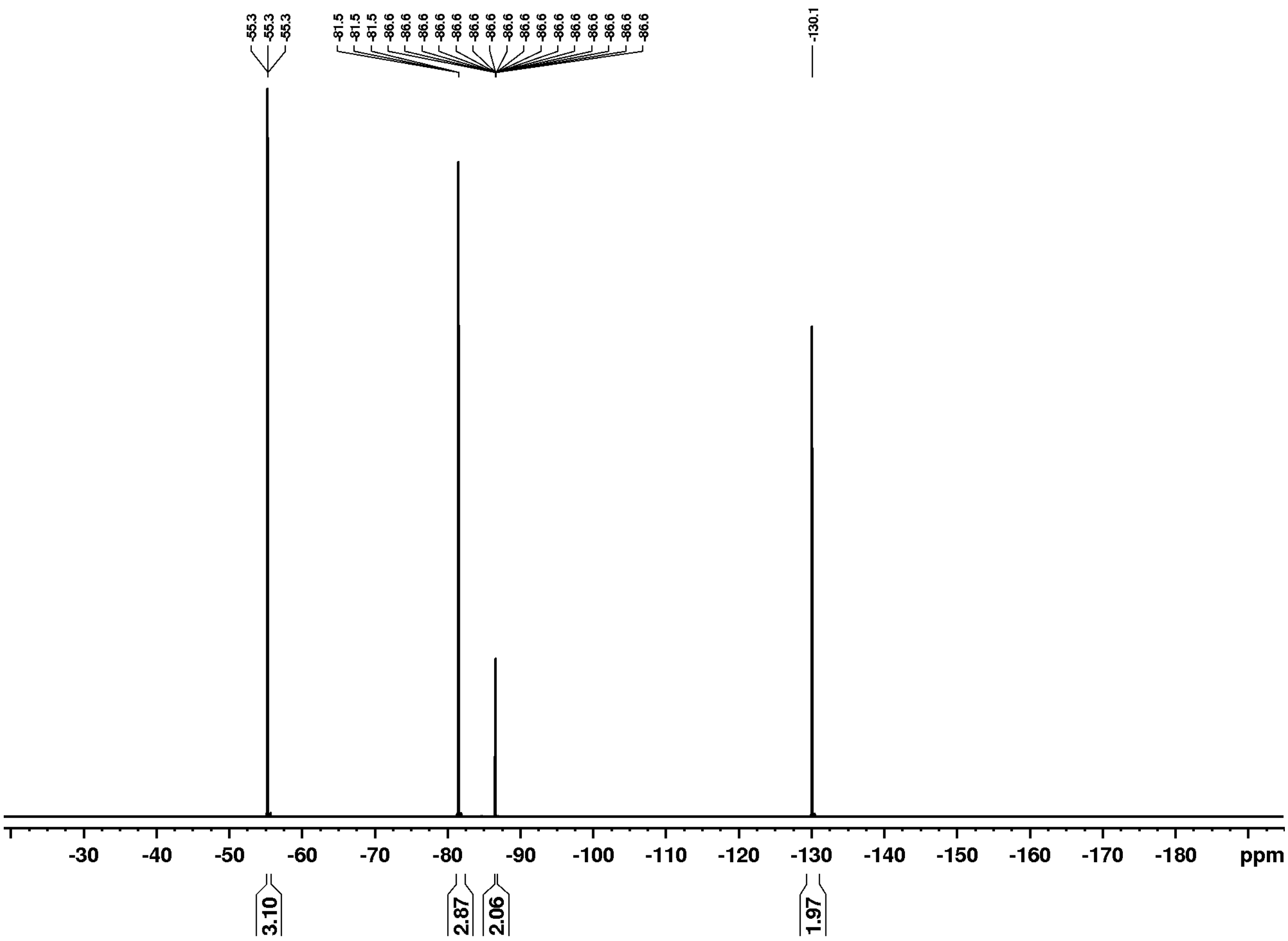

Figure S85. ^{19}F NMR of *n*-$C_3F_7OCF_3$ in $CDCl_3$.

3.3. Characterization of $(CF_3)_2CHOCH_3$

Boiling point (vapor pressure): T_{bp} = 51.1 °C at 1013 mbar (Antoine parameter: A = 4.155; B = 1102.6; C = −58.518 for equation: $p = 10^{(A-B/(T+C))}$).

Enthalpy of vaporization (T = 298.15 K): $\Delta_{vap}H$ = 32.7 kJ mol^{-1} (for equation: $\Delta_{vap}H = \ln(10)\cdot R\cdot T^2\cdot(B/(T+C)^2)/1000$).

^{1}H NMR (400.2 MHz, $CDCl_3$): δ = 3.92 (sept, 1H, $(CF_3)_2C\mathbf{H}OCH_3$, $^3J(^{19}F, ^1H)$ = 6.0 Hz), 3.72 (s, 3H, $(CF_3)_2CHOC\mathbf{H}_3$) ppm.

^{1}H{^{19}F} NMR (400.2 MHz, $CDCl_3$): δ = 3.92 (s, 1H, $(CF_3)_2C\mathbf{H}OCH_3$), 3.72 (s, 3H, $(CF_3)_2CHOC\mathbf{H}_3$) ppm.

^{13}C NMR (100.7 MHz, $CDCl_3$): δ = 121.8 (qm, 2C, $(\mathbf{C}F_3)_2CHOCH_3$, $^1J(^{19}F, ^{13}C)$ = 283.6 Hz), 77.8 (dseptq, 1C, $(CF_3)_2\mathbf{C}HOCH_3$; $^1J(^{13}C, ^1H)$ = 144.6 Hz, $^2J(^{19}F, ^{13}C)$ = 32.4 Hz, $^3J(^{13}C, ^1H)$ = 5.0 Hz), 62.6 (qd, 1C, $(CF_3)_2CHO\mathbf{C}H_3$, $^1J(^{13}C, ^1H)$ = 145.0 Hz, $^3J(^{13}C, ^1H)$ = 6.2 Hz) ppm.

^{13}C{^{1}H} NMR (100.7 MHz, $CDCl_3$): δ = 121.8 (qm, 2C, $(\mathbf{C}F_3)_2CHOCH_3$, $^1J(^{19}F, ^{13}C)$ = 283.6 Hz), 77.9 (sept, 1C, $(CF_3)_2\mathbf{C}HOCH_3$; $^2J(^{19}F, ^{13}C)$ = 32.4 Hz), 62.6 (s, 1C, $(CF_3)_2CHO\mathbf{C}H_3$) ppm.

^{17}O NMR (54.3 MHz, $CDCl_3$): δ = −24.0 (s, 1O, $(CF_3)_2CH\mathbf{O}CH_3$) ppm.

^{19}F NMR (376.6 MHz, $CDCl_3$): δ = −74.4 (d, 6F, $(C\mathbf{F}_3)_2CHOCH_3$, $^4J(^{19}F, ^{19}F)$ = 6.0 Hz) ppm.

^{19}F{^{1}H} NMR (376.6 MHz, $CDCl_3$): δ = −74.4 (s, 6F, $(C\mathbf{F}_3)_2CHOCH_3$) ppm.

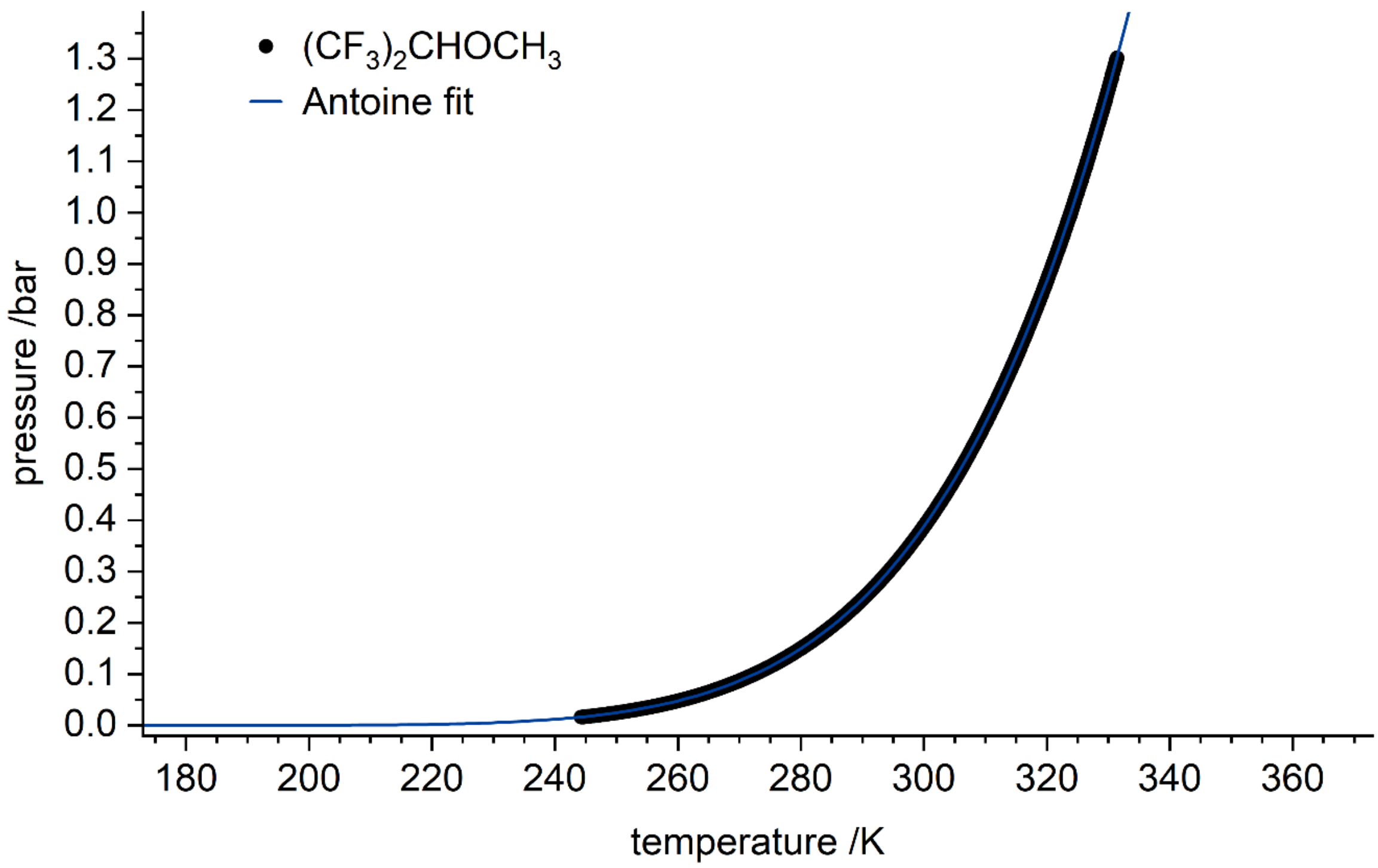

Figure S86. Vapor pressure measurement and Antoine fit of $(CF_3)_2CHOCH_3$ (Antoine parameter: A = 4.155; B = 1102.6; C = −58.518 for equation: $p = 10^{(A-B/(T+C))}$).

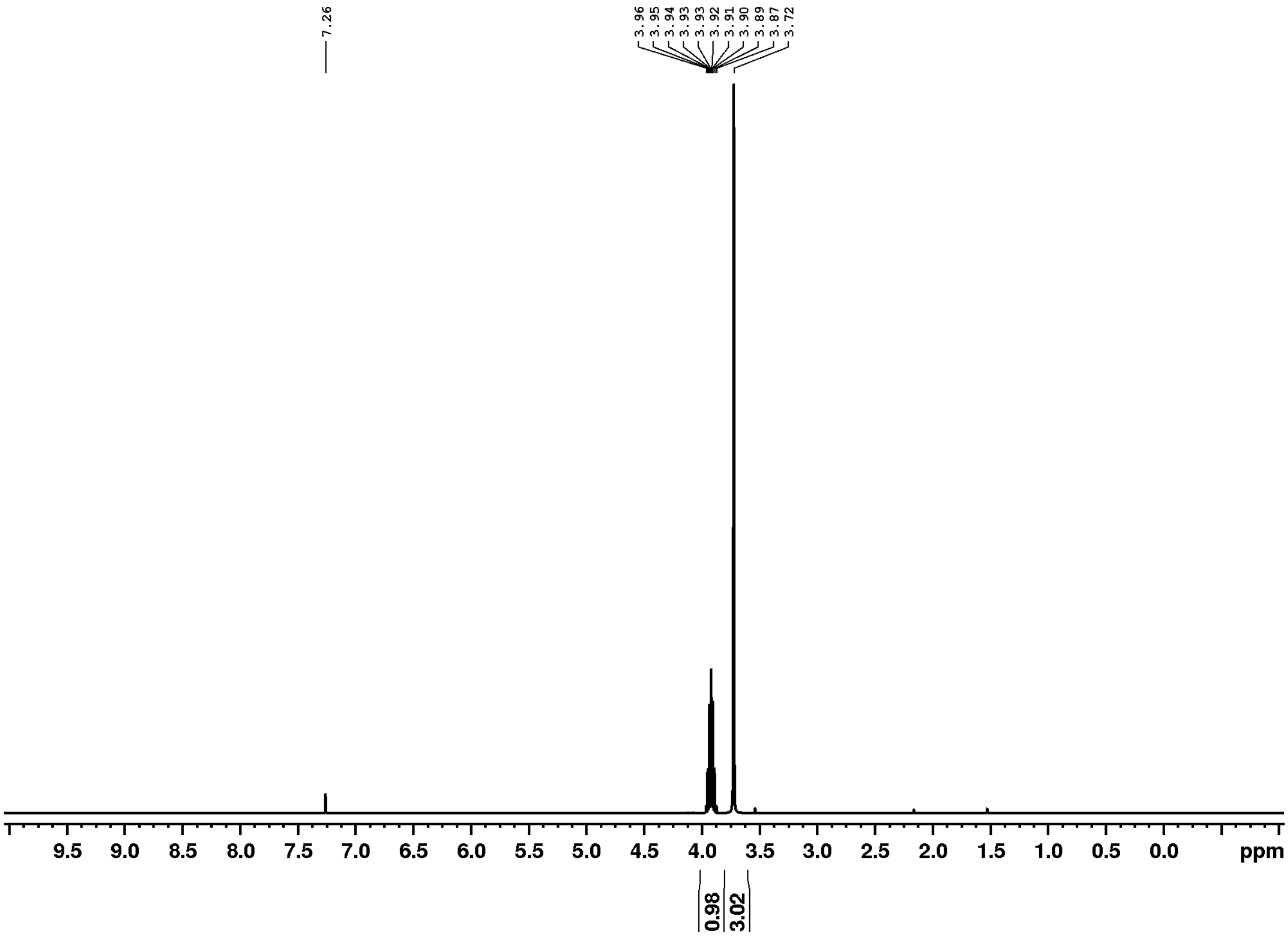

Figure S87. ^{1}H NMR of $(CF_3)_2CHOCH_3$ in $CDCl_3$.

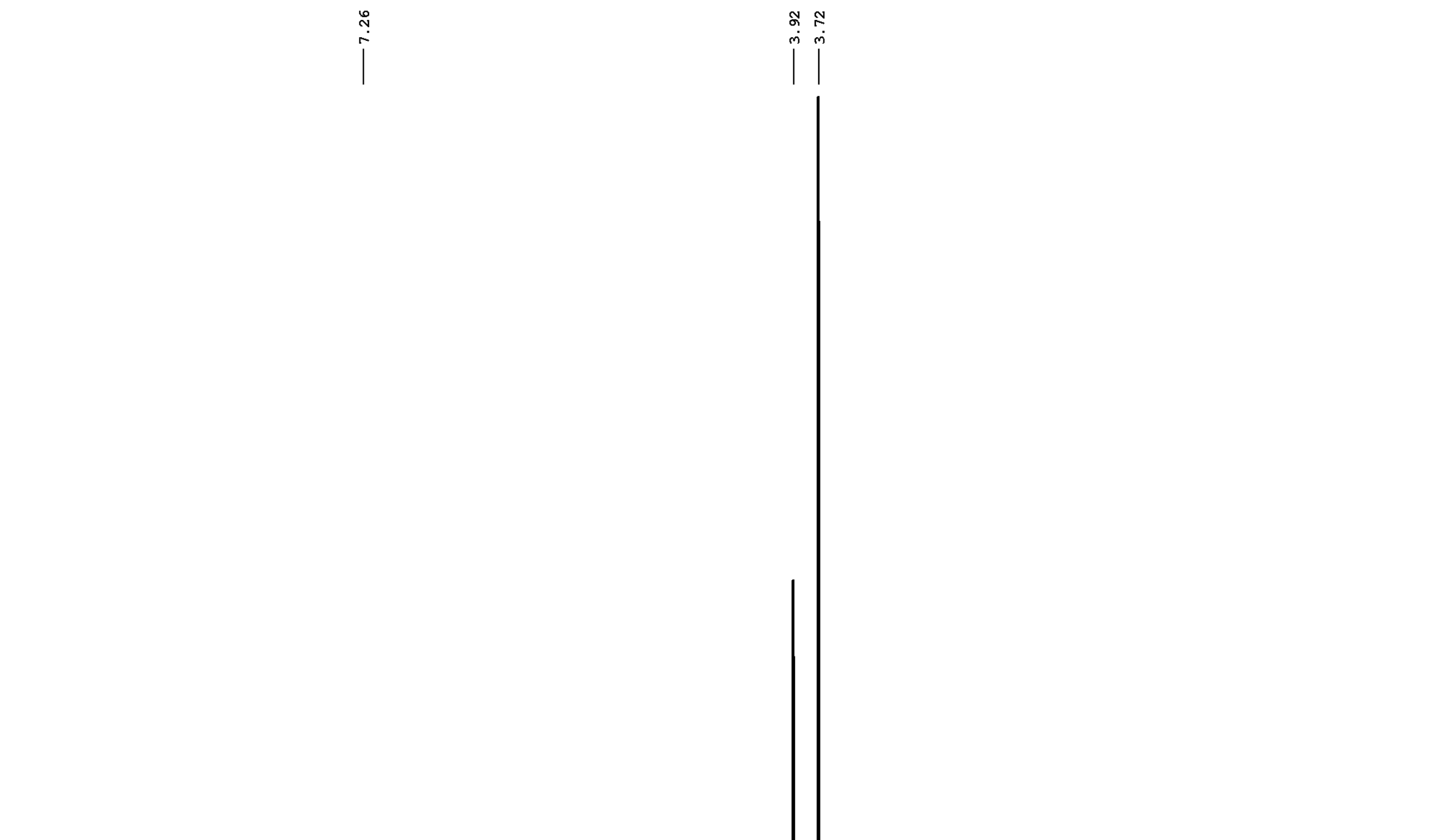

Figure S88. ^{1}H{^{19}F} NMR of $(CF_3)_2CHOCH_3$ in $CDCl_3$.

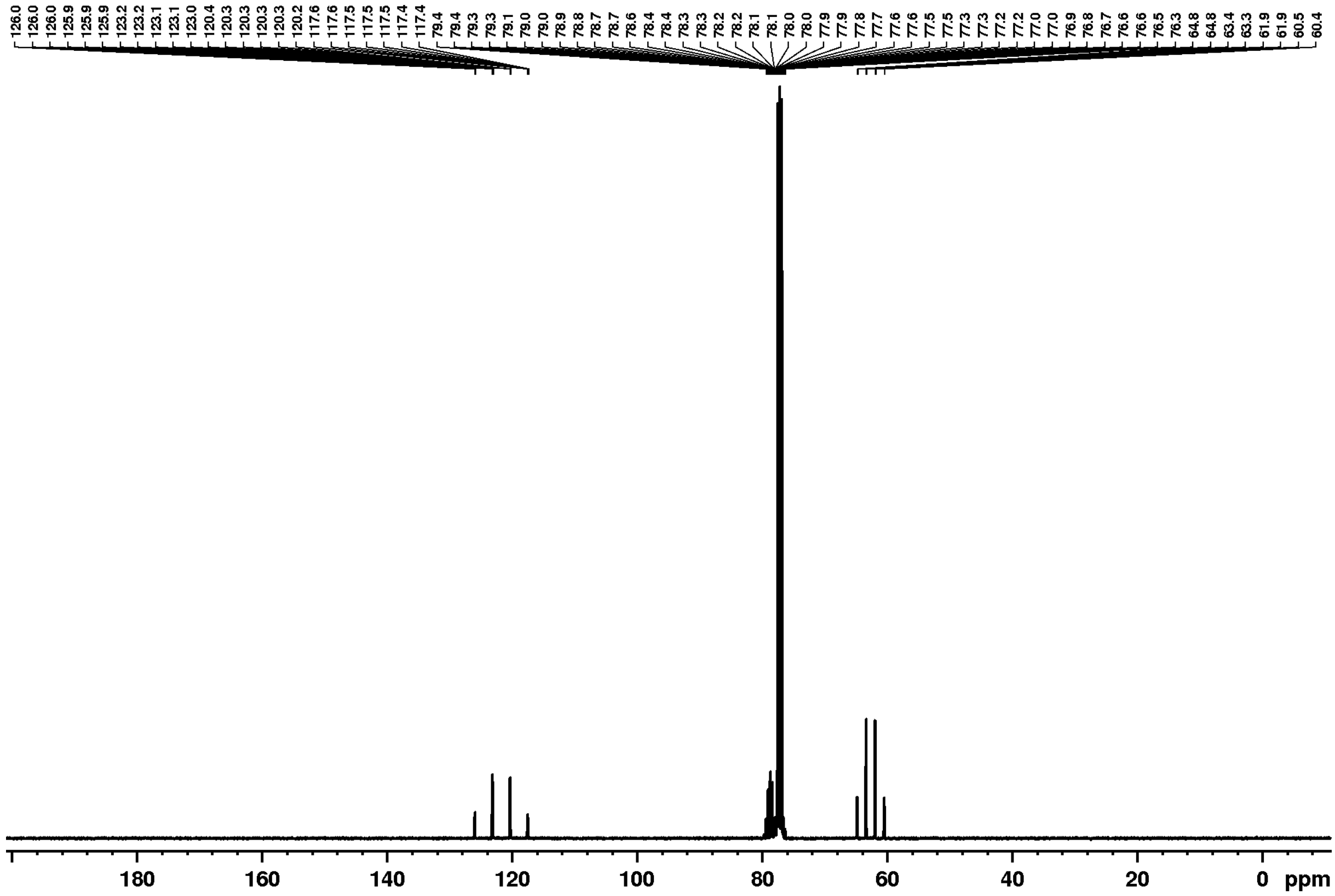

Figure S89. ^{13}C NMR of $(CF_3)_2CHOCH_3$ in $CDCl_3$.

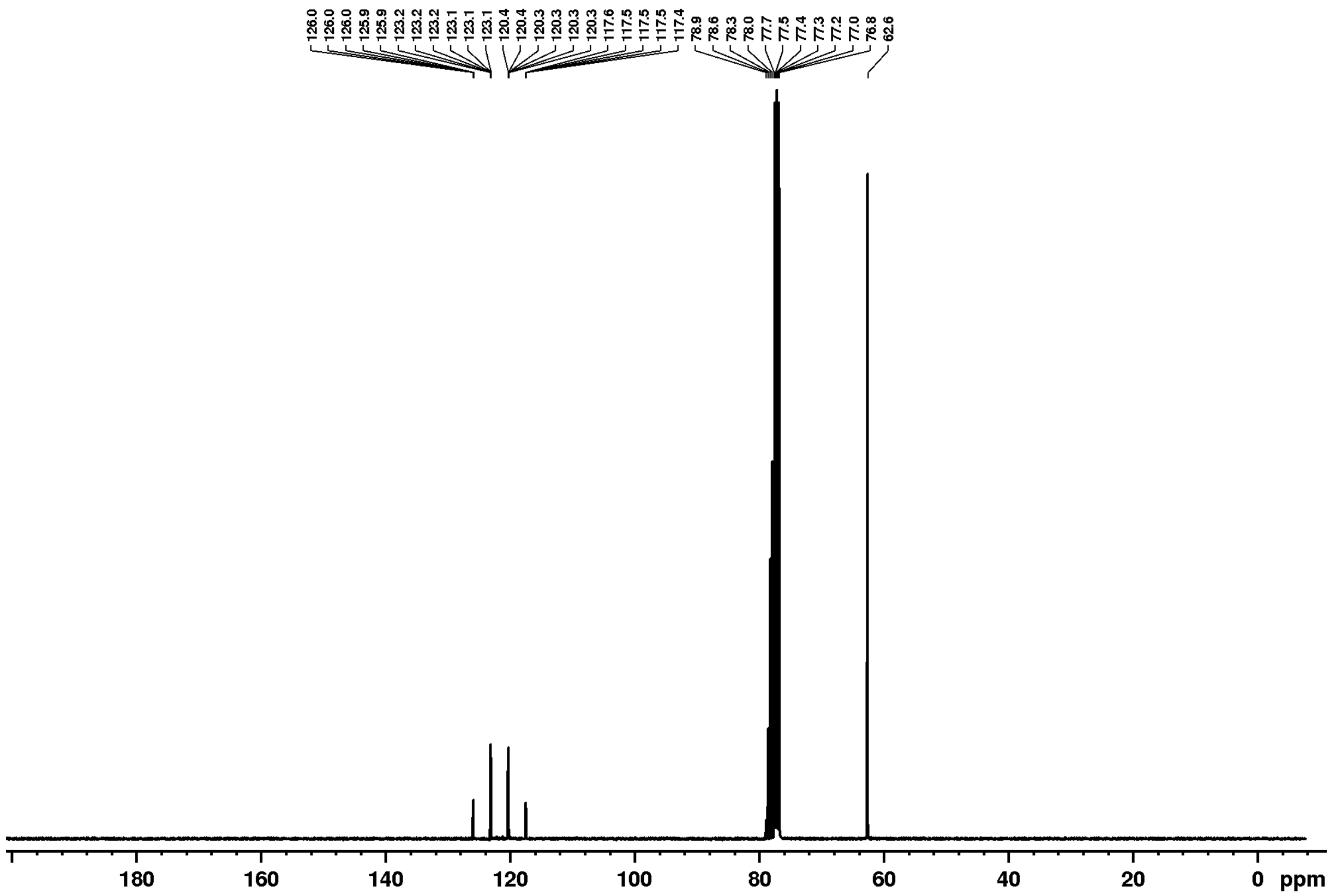

Figure S90. $^{13}C\{^1H\}$ NMR of $(CF_3)_2CHOCH_3$ in $CDCl_3$.

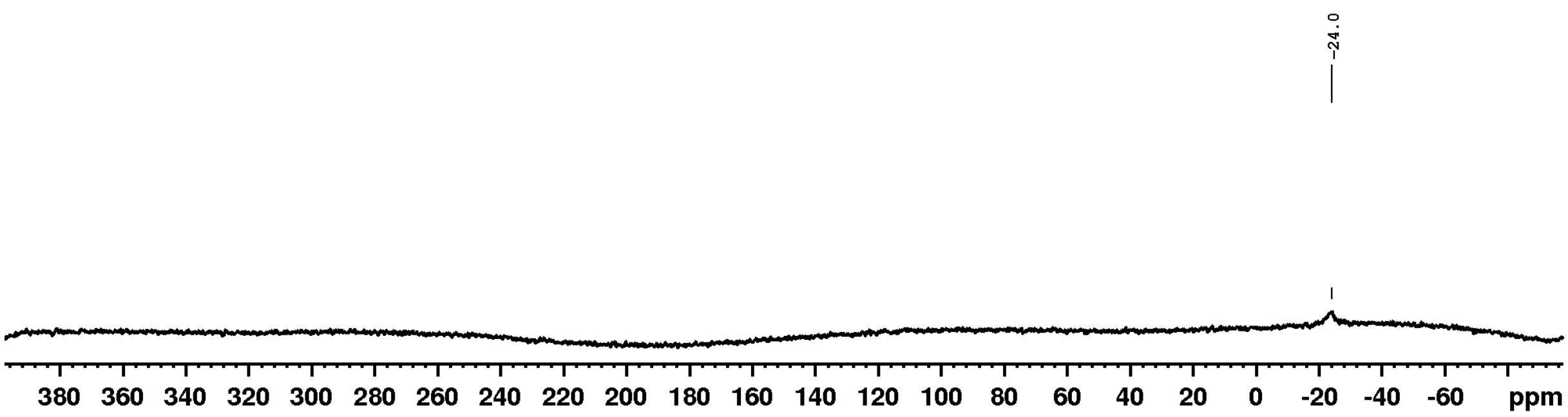

Figure S91. ^{17}O NMR of $(CF_3)_2CHOCH_3$ in $CDCl_3$.

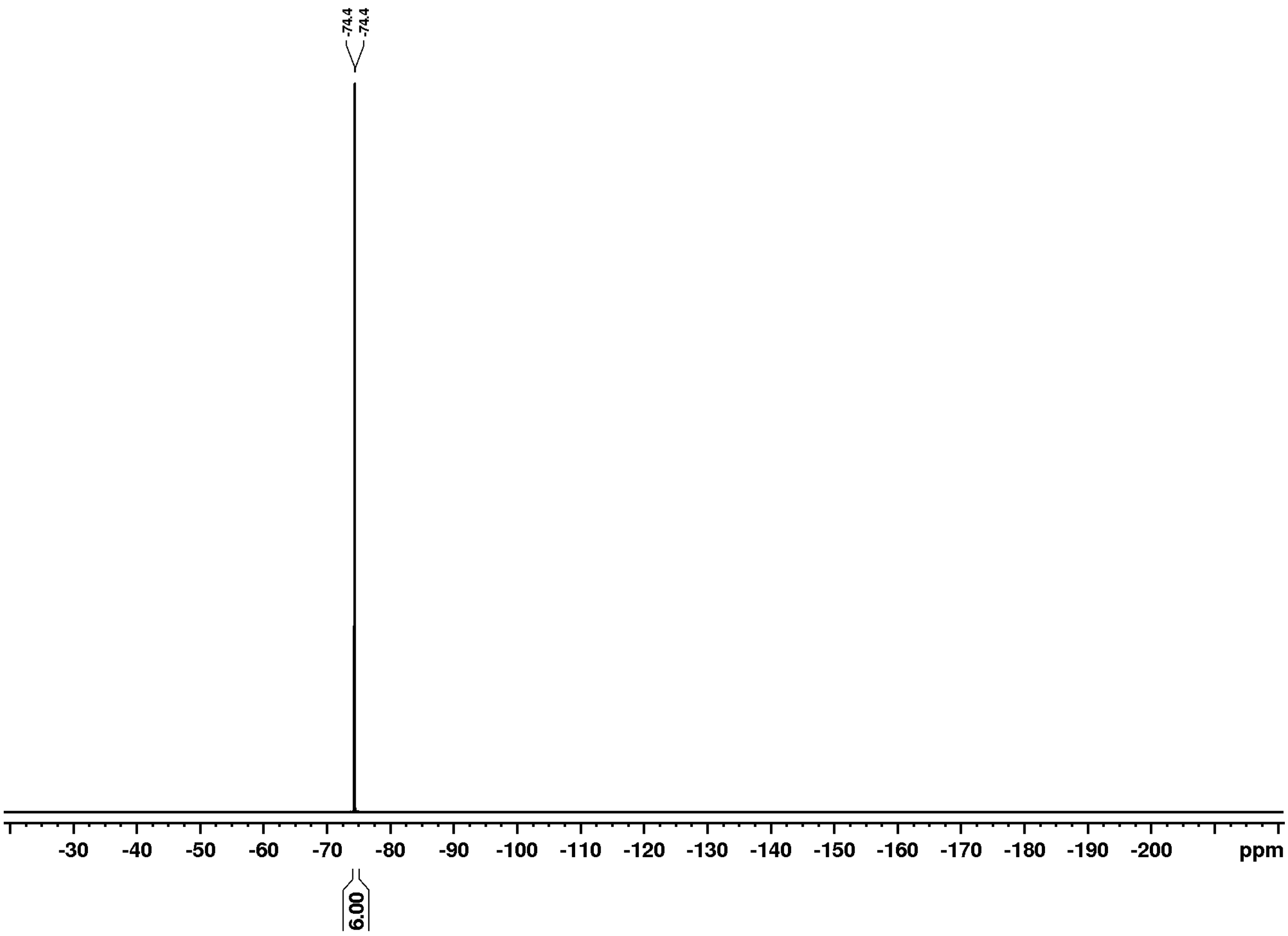

Figure S92. ^{19}F NMR of $(CF_3)_2CHOCH_3$ in $CDCl_3$.

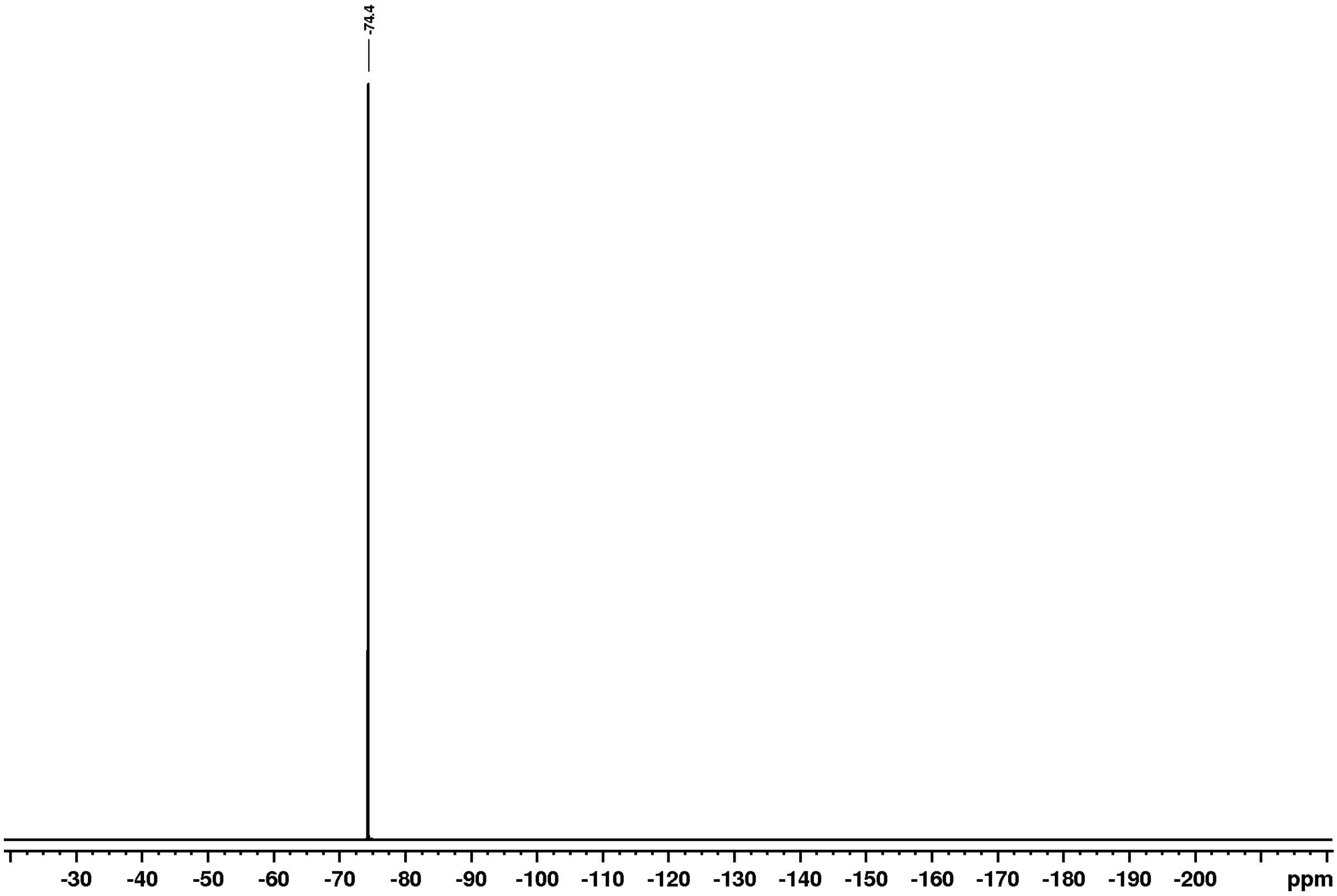

Figure S93. $^{19}F\{^{1}H\}$ NMR of $(CF_3)_2CHOCH_3$ in $CDCl_3$.

3.4. Electrochemical fluorination of $(CF_3)_3COCH_3$

A 350 mL stainless steel cell with nickel electrodes (effective anode area S = 2.6 dm^2), the construction of which is described elsewhere [15], was filled with anhydrous hydrogen fluoride (290 mL). A constant voltage of 5.0 V was applied for 20 hours. $(CF_3)_3COCH_3$ (177.6 g, 710.3 mmol) was added at regular intervals to the pre-electrolyzed anhydrous HF. The gaseous products from the cell were collected in a PTFE trap maintained at −78 °C. The cell was operated at 5.3 – 6.0 V and the current was held between 0.9 and 1.1 A. The cell temperature was maintained at 0 °C and the temperature of the condenser was kept at −31 °C. After consumption of 124 Ah the reaction was stopped. In total, 111 g of crude organic material was collected from the cell and the cold traps. The aHF from the cell was carefully poured onto ice (130 g). An organic layer (52 g) separated from the aqueous phase. Both organic phases were combined, and the residual HF was removed by the addition of potassium fluoride. Afterwards, the organic layer was separated from the formed bifluoride by condensation.

Separation of the mixture of $(CF_3)_3COCF_3$ (56%), $(CF_3)_3COCHF_2$ (22%) and $(CF_3)_3COCH_3$ (19%) was carried out via distillation. $(CF_3)_3COCH_2F$ (3%) was not isolated.

***t*-$C_4F_9OCH_3$**

Boiling point (distillation): T_{bp} = 52 °C at 990 mbar.

Boiling point (vapor pressure): T_{bp} = 53.6 °C at 1013 mbar (Antoine parameter: A = 4.023; B = 1089.3 ; C = −55.616 for equation: $p = 10^{(A-B/(T+C))}$).

Enthalpy of vaporization (T = 298.15 K): $\Delta_{vap}H$ = 31.5 kJ mol^{-1} (for equation: $\Delta_{vap}H = \ln(10)\cdot R\cdot T^2\cdot(B/(T+C)^2)/1000$).

^{1}H NMR (400.2 MHz, $CDCl_3$): δ = 3.82 (dec, 3H, $(CF_3)_3COC\mathbf{H}_3$ $^5J(^{19}F, ^1H)$ = 1.1 Hz) ppm.

$^1H\{^{19}F\}$ NMR (400.2 MHz, $CDCl_3$): δ = 3.82 (s, 3H, $(CF_3)_3COC\mathbf{H}_3$) ppm.

^{13}C NMR (100.7 MHz, $CDCl_3$): δ = 120.6 (qm, 3C, $(\mathbf{C}F_3)_3COCH_3$, $^1J(^{19}F, ^{13}C)$ = 294.3 Hz), 80.0 (decq, 1C, $(CF_3)_3\mathbf{C}OCH_3$, $^2J(^{19}F, ^{13}C)$ = 29.6 Hz, $^3J(^{13}C, ^1H)$ = 3.6 Hz), 57.2 (qdec, 1C, $(CF_3)_3CO\mathbf{C}H_3$, $^3J(^{13}C, ^1H)$ = 147.6 Hz, $^4J(^{19}F, ^{13}C)$ = 1.8 Hz) ppm.

$^{13}C\{^1H\}$ NMR (100.7 MHz, $CDCl_3$): δ = 120.6 (qm, 3C, $(\mathbf{C}F_3)_3COCH_3$, $^1J(^{19}F, ^{13}C)$ = 294.3 Hz), 80.0 (dec, 1C, $(CF_3)_3\mathbf{C}OCH_3$, $^2J(^{19}F, ^{13}C)$ = 29.5 Hz), 57.2 (dec, 1C, $(CF_3)_3CO\mathbf{C}H_3$, $^4J(^{19}F, ^{13}C)$ = 1.8 Hz) ppm.

^{17}O NMR (54.3 MHz, $(CH_3)_2CO$): δ = −14.9 (s, 1O, $(CF_3)_3C\mathbf{O}CH_3$) ppm.

^{19}F NMR (376.6 MHz, $CDCl_3$): δ = −70.7 (q, 9F, $(C\mathbf{F}_3)_3OCH_3$, $^5J(^{19}F, ^1H)$ = 1.2 Hz) ppm.

$^{19}F\{^1H\}$ NMR (376.6 MHz, $CDCl_3$): δ = −70.7 (s, 9F, $(C\mathbf{F}_3)_3OCH_3$) ppm.**GC-MS** [t = 0.48 min] m/z^+: 249 [M −H], 231 [M −F], 181 [M $-CF_3$], 97 [M $-C_2F_6CH_3$], 69 [M – $(CF_3)_2COCH_3$].

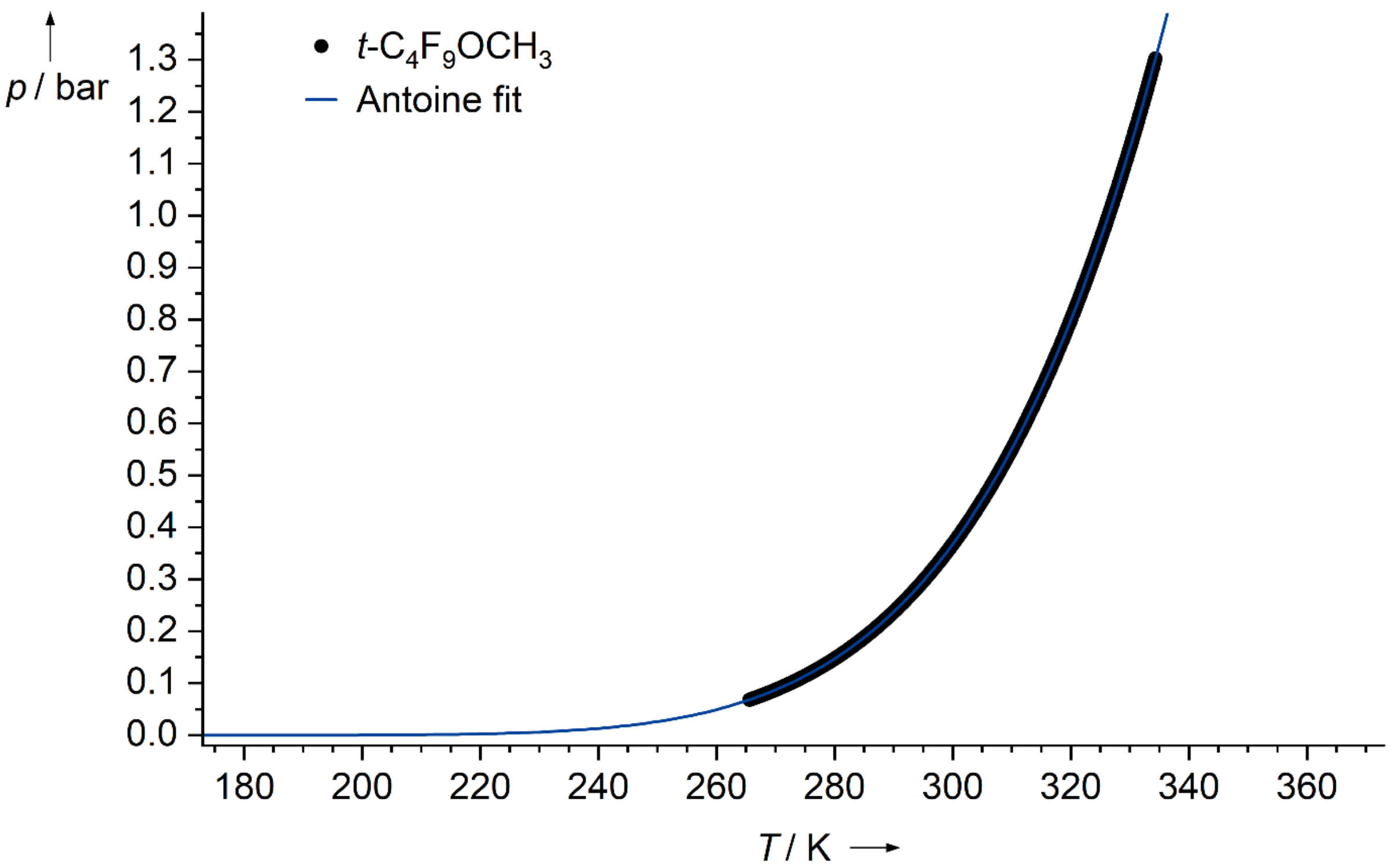

Figure S94. Vapor pressure measurement and Antoine fit of *t*-$C_4F_9OCH_3$ (Antoine parameter: A = 4.023; B = 1089.3; C = −55.616 for equation: $p = 10^{(A-B/(T+C))}$).

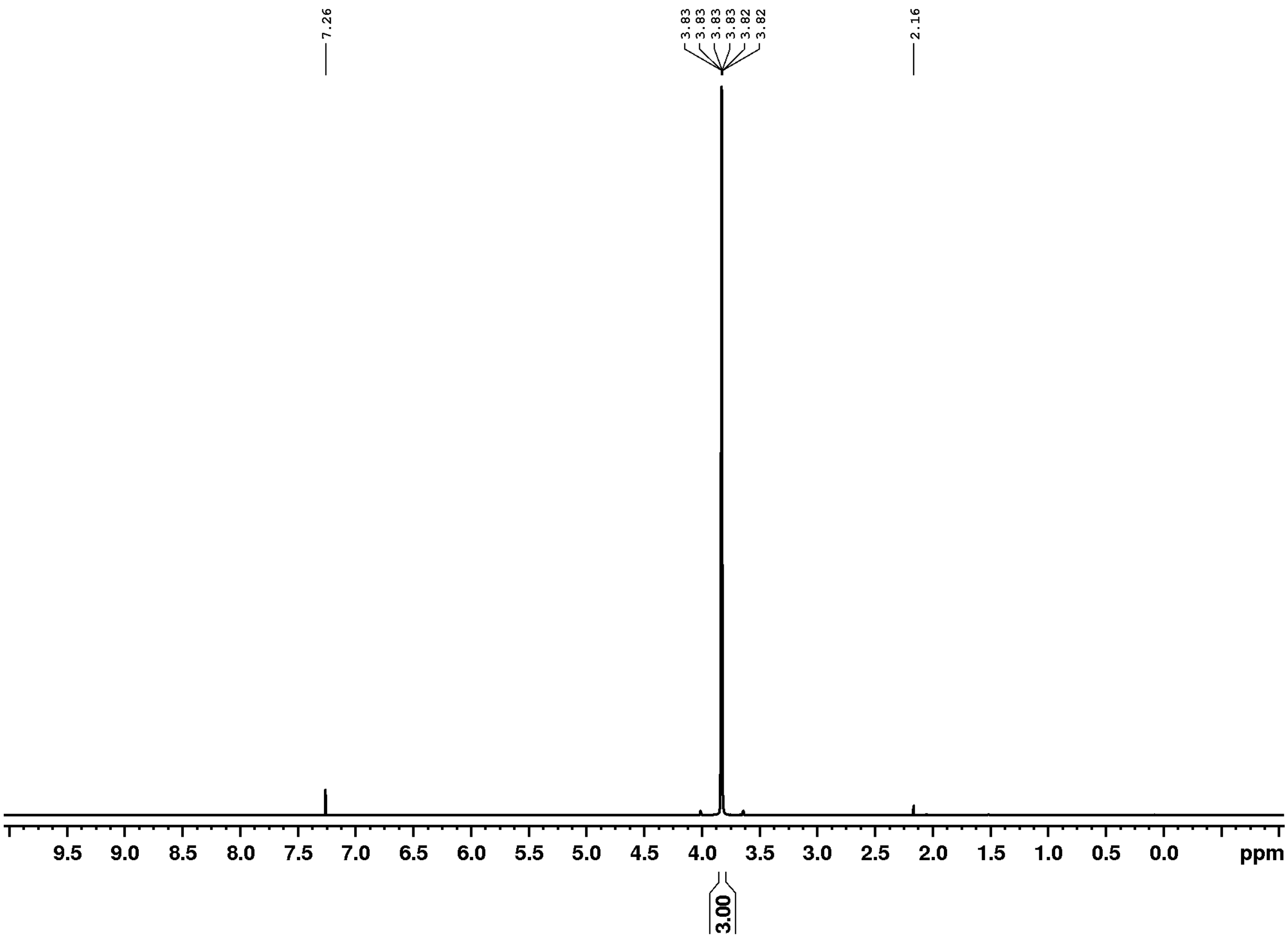

Figure S95. ^{1}H NMR of $(CF_3)_3COCH_3$ in $CDCl_3$.

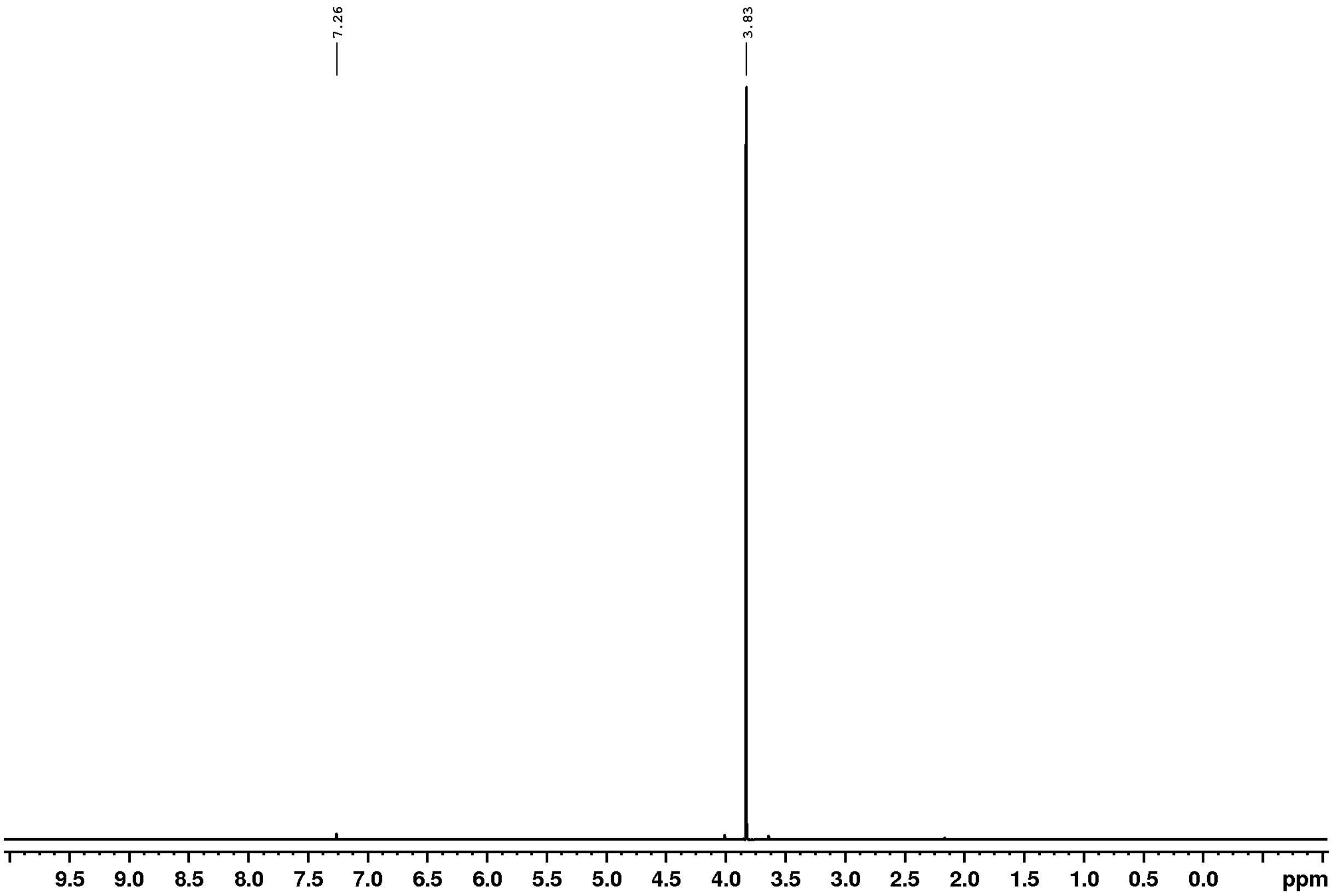

Figure S96. $^{1}H\{^{19}F\}$ NMR of $(CF_3)_3COCH_3$ in $CDCl_3$.

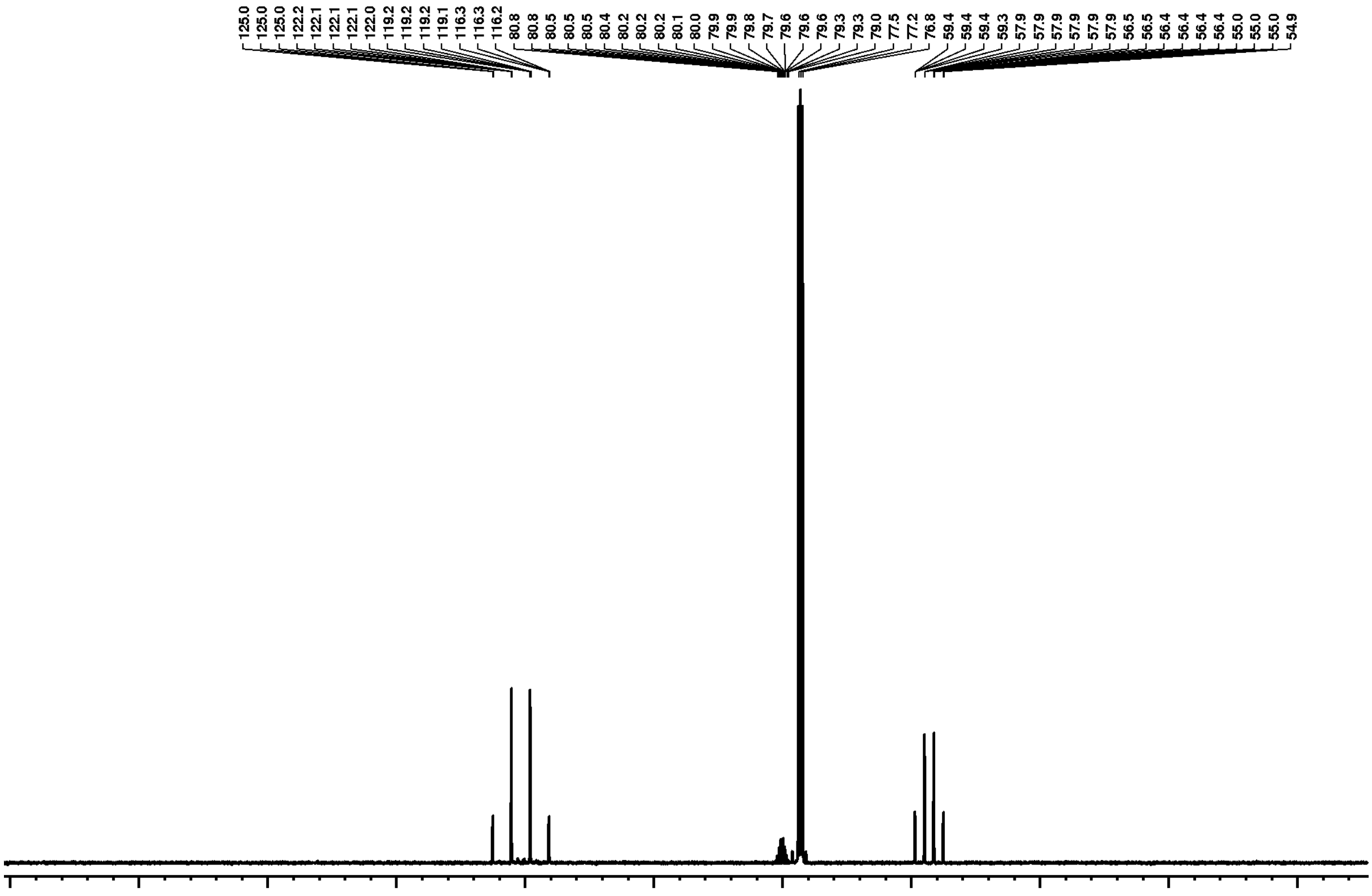

Figure S97. ^{13}C NMR of $(CF_3)_3COCH_3$ in $CDCl_3$.

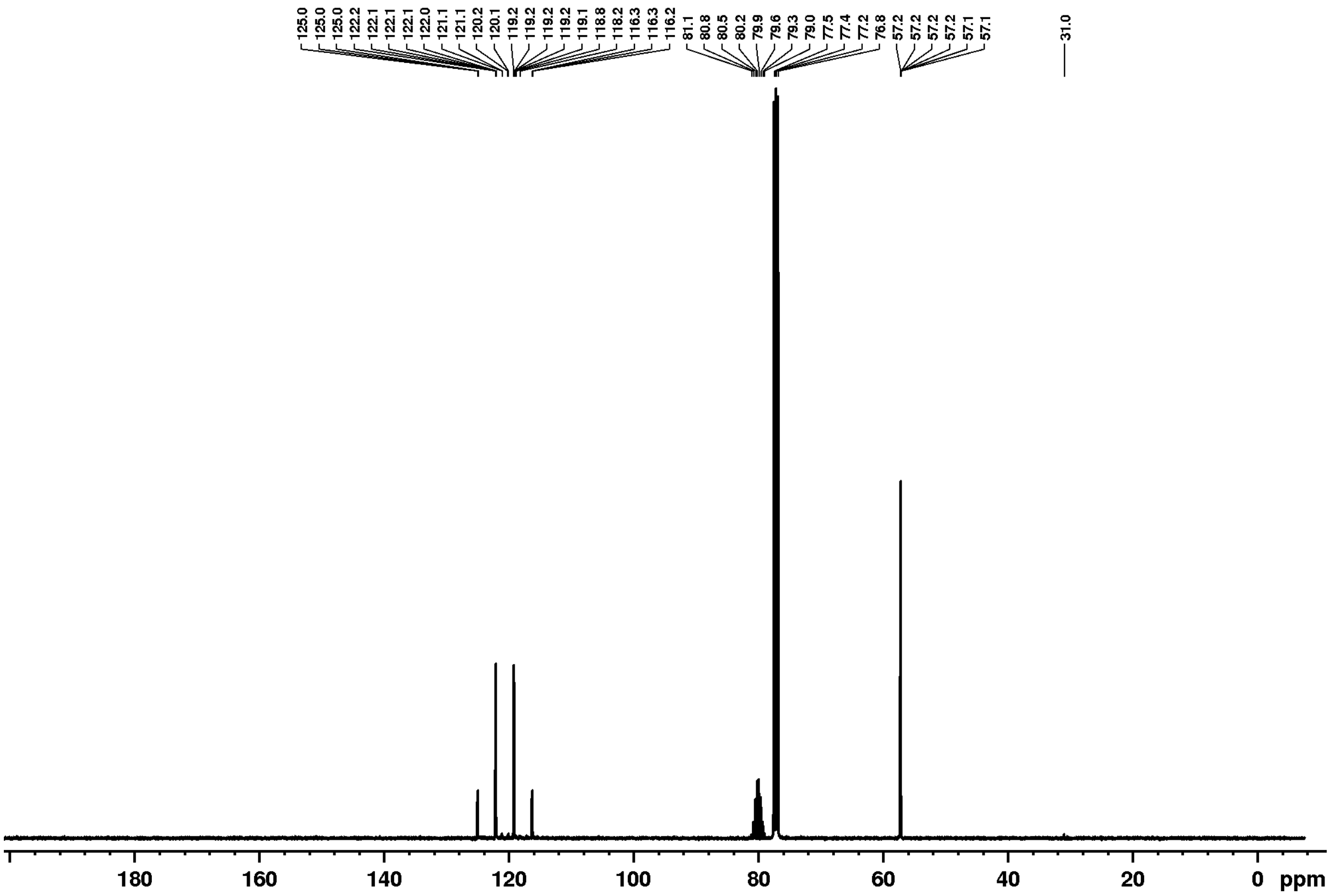

Figure S98. $^{13}C\{^{1}H\}$ NMR of $(CF_3)_3COCH_3$ in $CDCl_3$.

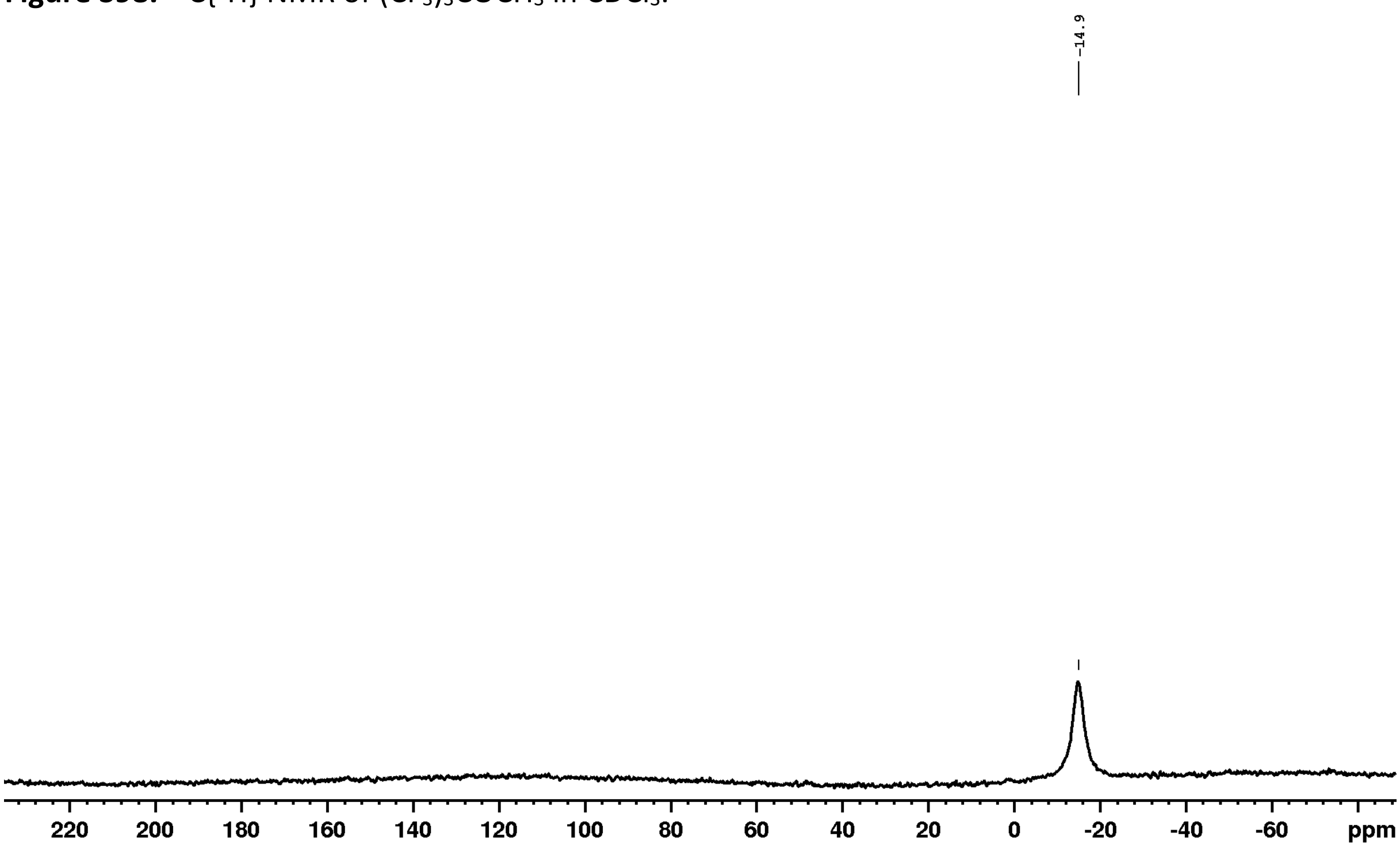

Figure S99. ^{17}O NMR of $(CF_3)_3COCH_3$ measured as neat substance with acetone-d_6 and CCl_3F as external standard.

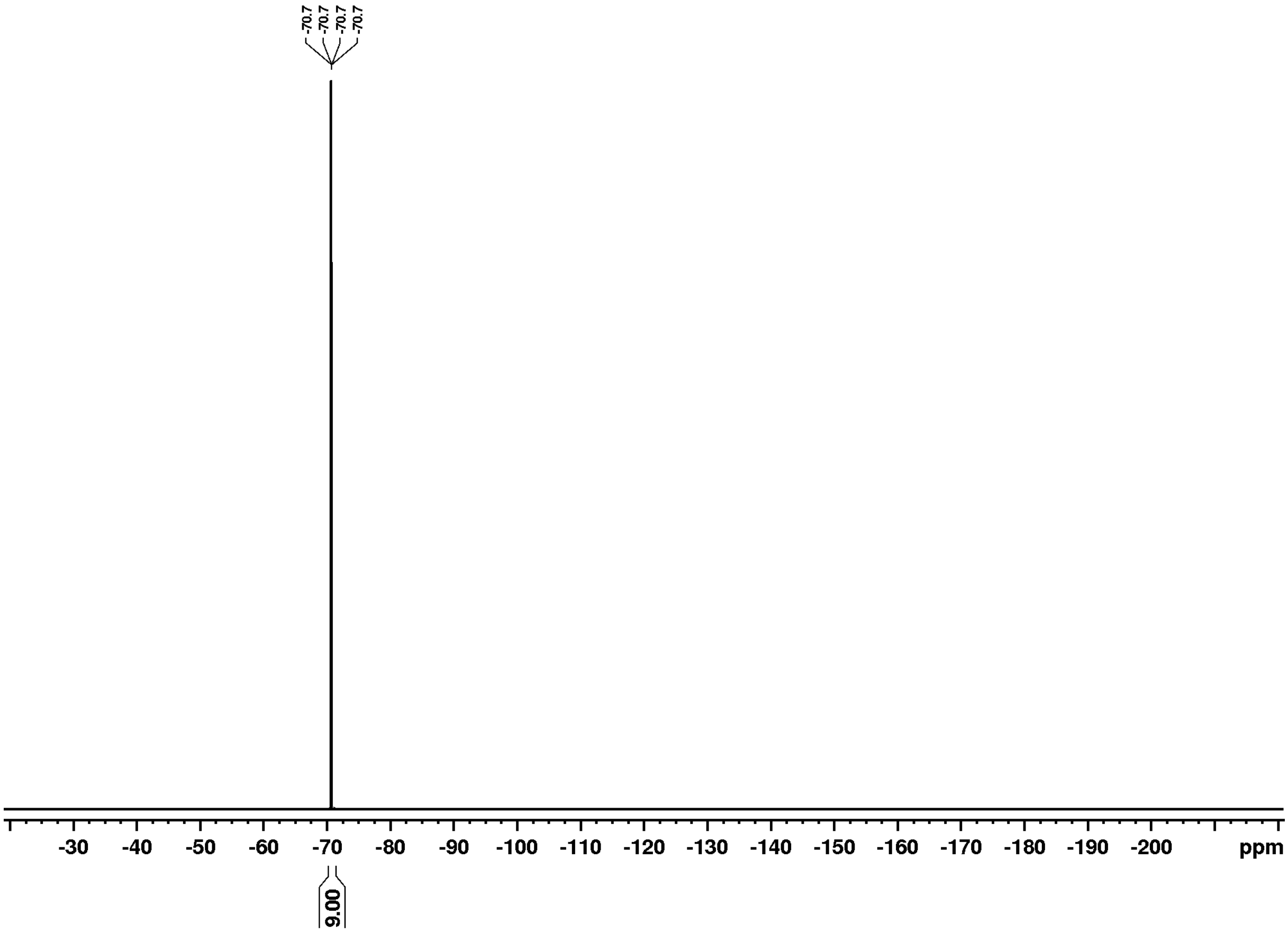

Figure S100. ^{19}F NMR of $(CF_3)_3COCH_3$ in $CDCl_3$.

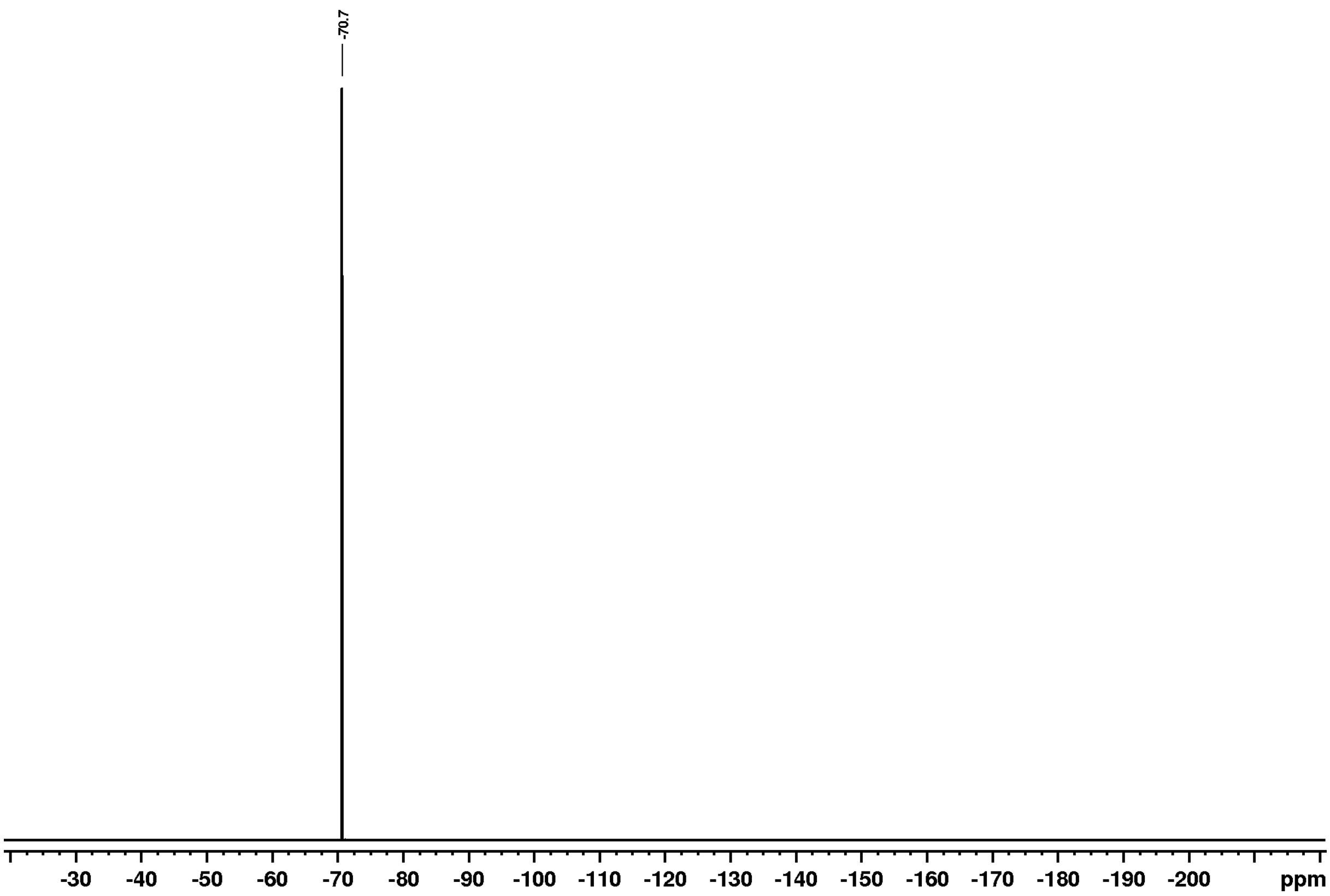

Figure S101. $^{19}F\{^1H\}$ NMR of $(CF_3)_3COCH_3$ in $CDCl_3$.

***t*-$C_4F_9OCH_2F$** (not isolated, mixture with $(CF_3)_3COCH_3$ and other, unidentified species)

^{1}H NMR (400.3 MHz, $(CH_3)_2CO$): δ = 4.79 (t, 2H, $(CF_3)_3COC\mathbf{H}_2F$, $^5J(^{19}F, ^{19}F)$ = 51.3 Hz) ppm.**^{1}H{^{19}F} NMR** (400.3 MHz, $(CH_3)_2CO$): δ = 4.79 (s, 2H, $(CF_3)_3COC\mathbf{H}_2F$) ppm.

^{19}F NMR (470.6 MHz, $(CH_3)_2CO$): δ = −72.4 (d, 9F, $(C\mathbf{F}_3)_3COCH_2F$, $^5J(^{19}F, ^{19}F)$ = 5.5 Hz), −151.1 (tdec, 1F, $(CF_3)_3COCH_2\mathbf{F}$, $^2J(^{19}F, ^1H)$ = 51.3.6 Hz, $^5J(^{19}F, ^{19}F)$ = 5.4 Hz) ppm.

^{19}F{^{1}H} NMR (470.6 MHz, $(CH_3)_2CO$): δ = −72.4 (d, 9F, $(C\mathbf{F}_3)_3COCH_2F$, $^5J(^{19}F, ^{19}F)$ = 5.5 Hz), −151.1 (dec, 1F, $(CF_3)_3COCH_2\mathbf{F}$, $^5J(^{19}F, ^{19}F)$ = 5.4 Hz) ppm.

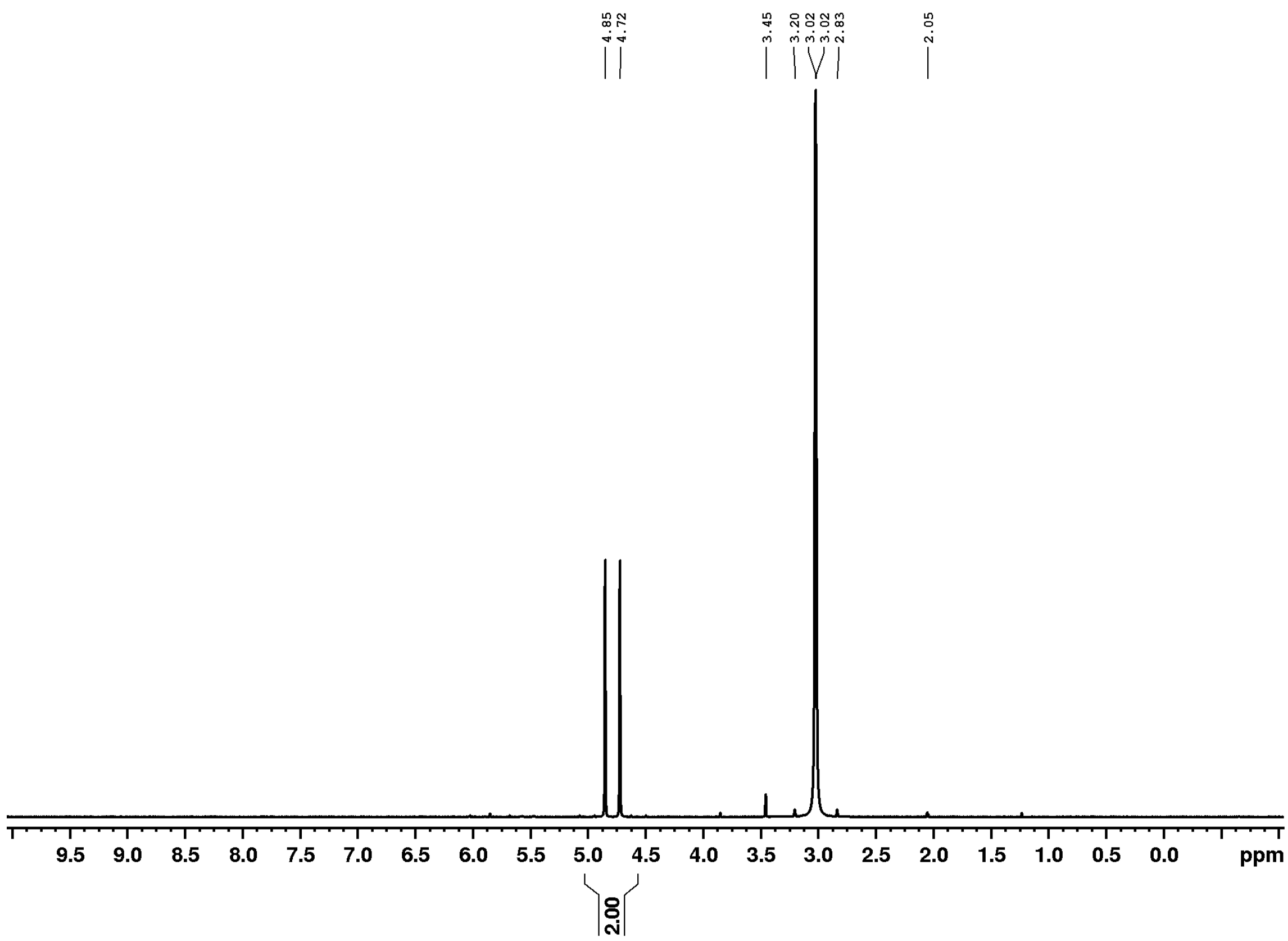

Figure S102. ^1H NMR of $(CF_3)_3COCH_2F$ (mixture with $(CF_3)_3COCH_3$ and other, unidentified species) in $CDCl_3$.

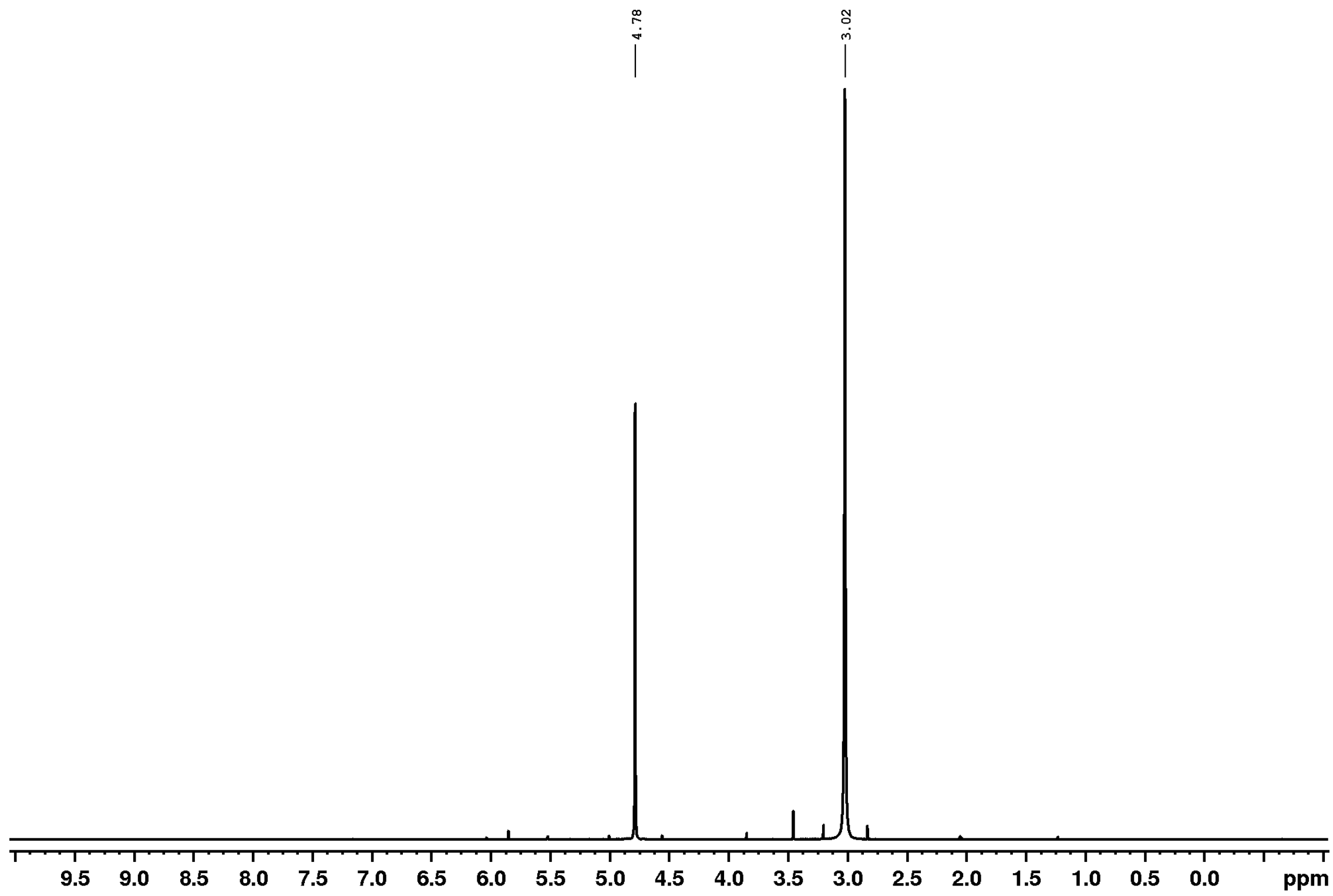

Figure S103. $^1H\{^{19}F\}$ NMR of $(CF_3)_3COCH_2F$ (mixture with $(CF_3)_3COCH_3$ and other, unidentified species) measured as neat substance with acetone-d_6 and CCl_3F as external standard.

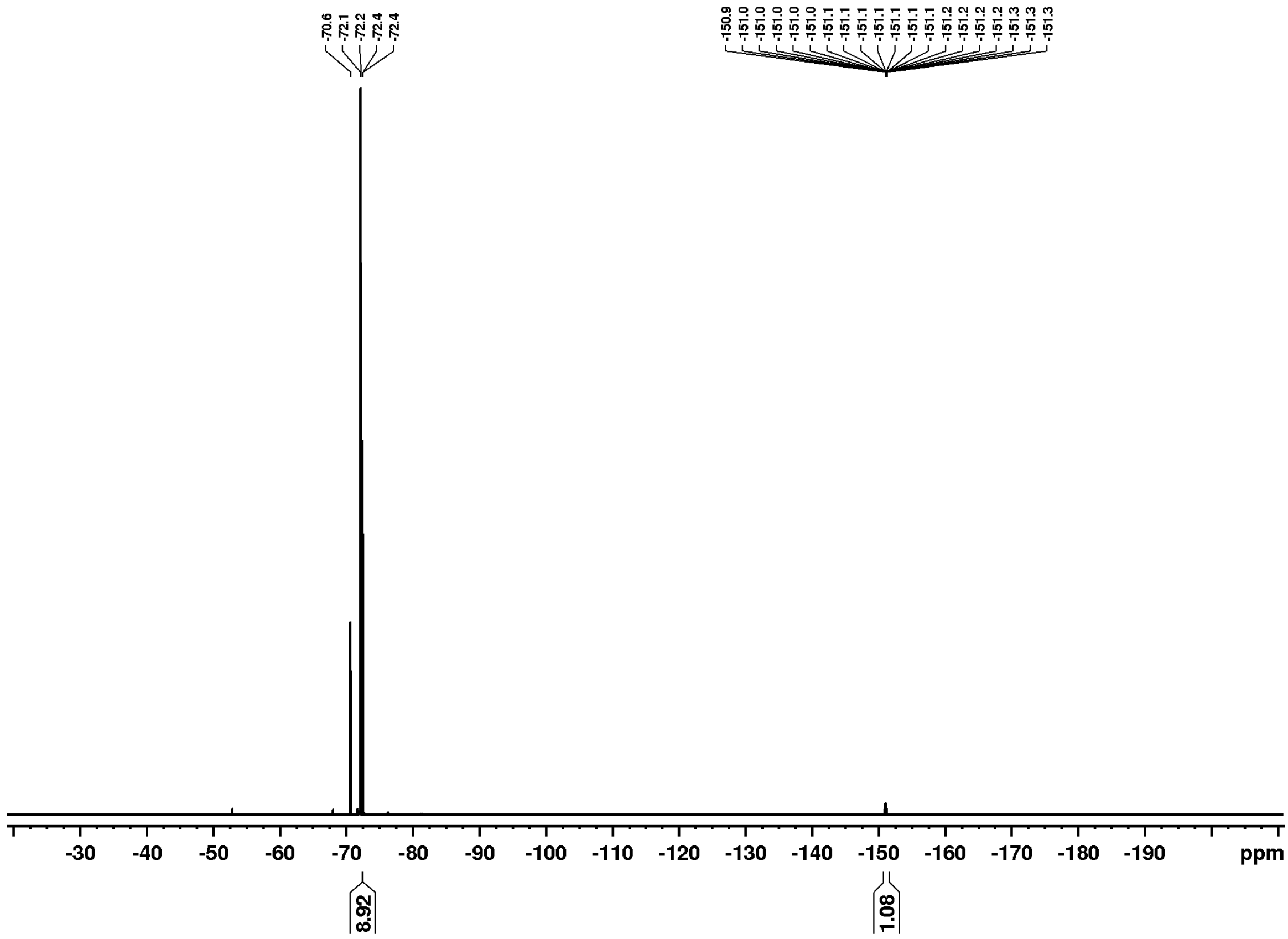

Figure S104. ^{19}F NMR of $(CF_3)_3COCH_2F$ (mixture with $(CF_3)_3COCH_3$ and other, unidentified species) measured as neat substance with acetone-d_6 and CCl_3F as external standard.

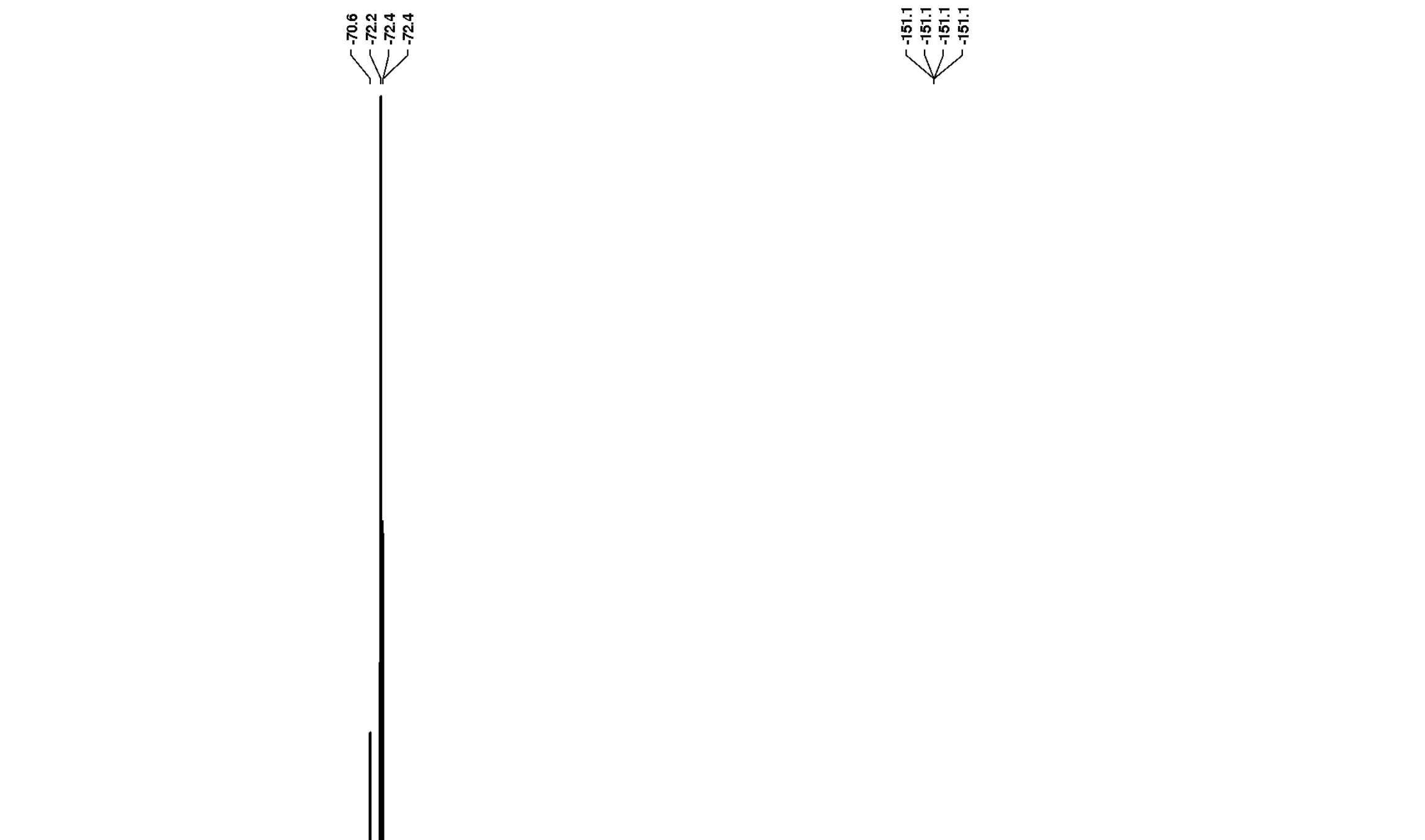

Figure S105. $^{19}F\{^1H\}$ NMR of $(CF_3)_3COCH_2F$ (mixture with $(CF_3)_3COCH_3$ and other, unidentified species) measured as neat substance with acetone-d_6 and CCl_3F as external standard.

***t*-$C_4F_9OCHF_2$**

Boiling point (distillation): T_{bp} = 47 °C at 989 mbar.

Boiling point (vapor pressure): T_{bp} = 47.2 °C at 1013 mbar (Antoine parameter: A = 4.027; B = 1061.5; C = −56.347 for equation: $p = 10^{(A-B/(T+C))}$).

Enthalpy of vaporization (T = 298.15 K): $\Delta_{vap}H$ = 30.9 kJ mol^{-1} (for equation: $\Delta_{vap}H = \ln(10)\cdot R\cdot T^2\cdot(B/(T+C)^2)/1000$).

^{1}H NMR (600.2 MHz, $CDCl_3$): δ = 6.64 (t, 1H, $(C\mathbf{F}_3)_3COCHF_2$, $^2J(^{19}F, ^1H)$ = 69.0 Hz) ppm.

^{1}H{^{19}F} NMR (600.2 MHz, $(CH_3)_2CO$): δ = 6.64 (s, 1H, $(C\mathbf{F}_3)_3COCHF_2$) ppm.

^{13}C NMR (100.7 MHz, $CDCl_3$): δ = 119.6 (qm, 3C, $(\mathbf{C}F_3)_3COCHF_2$, $^1J(^{19}F, ^{13}C)$ = 291.4 Hz), 114.4 (tddec, 1C, $(CF_3)_3CO\mathbf{C}HF_2$, $^1J(^{19}F, ^{13}C)$ = 261.4 Hz, $^1J(^{13}C, ^1H)$ = 223.6 Hz, $^4J(^{19}F, ^{13}C)$ = 2.1 Hz), 79.6 (dectd, 1C, $(CF_3)_3\mathbf{C}OCHF_2$, $^2J(^{19}F, ^{13}C)$ = 31.6 Hz, $^3J(^{19}F, ^{13}C)$ = 2.7 Hz, $^3J(^{13}C, ^1H)$ = 1.5 Hz) ppm.

^{13}C{^{1}H} NMR (150.9 MHz, $CDCl_3$): δ = 119.6 (qm, 3C, $(\mathbf{C}F_3)_3COCHF_2$, $^1J(^{19}F, ^{13}C)$ = 292.0), 114.4 (tdec, 1C, $(CF_3)_3CO\mathbf{C}HF_2$, $^1J(^{19}F, ^{13}C)$ = 261.5 Hz, $^4J(^{19}F, ^{13}C)$ = 2.2 Hz), 79.6 (dect, 1C, $(CF_3)_3\mathbf{C}OCHF_2$, $^2J(^{19}F, ^{13}C)$ = 31.7 Hz, $^3J(^{19}F, ^{13}C)$ = 2.8 Hz) ppm.

^{13}C{^{19}F} NMR (150.9 MHz, $CDCl_3$): δ = 119.6 (m, 3C, $(\mathbf{C}F_3)_3COCHF_2$), 114.2 (d, 1C, $(CF_3)_3CO\mathbf{C}HF_2$, $^1J(^{13}C, ^1H)$ = 223.7 Hz), 79.6 (d, 1C, $(CF_3)_3\mathbf{C}OCHF_2$, $^3J(^{13}C, ^1H)$ = 1.4 Hz) ppm.

^{17}O NMR (81.4 MHz, $(CH_3)_2CO$): δ = 75.3 (s, 1O, $(CF_3)_3C\mathbf{O}CHF_2$) ppm.

^{19}F NMR (564.7 MHz, $CDCl_3$): δ = −71.3 (t, 9F, $(C\mathbf{F}_3)_3COCHF_2$, $^5J(^{19}F, ^{19}F)$ = 3.0 Hz), −82.8 (ddec, 2F, $(C\mathbf{F}_3)_3COCHF_2$, $^2J(^{19}F, ^1H)$ = 68.8 Hz, $^5J(^{19}F, ^{19}F)$ = 3.0 Hz) ppm.

^{19}F{^{1}H} NMR (564.7 MHz, $CDCl_3$): δ = −71.3 (t, 9F, $(C\mathbf{F}_3)_3COCHF_2$, $^5J(^{19}F, ^{19}F)$ = 3.0 Hz), −80.0 (ddec, 2F, $(C\mathbf{F}_3)_3COCHF_2$, $^5J(^{19}F, ^{19}F)$ = 3.0 Hz) ppm.

GC-MS [t = 0.44 min] m/z$^+$: 285 $[M]^+$, 267 [M −F], 217 [M −HCF_3], 131 [M −CF_4OCHF_2], 69 [M −$(CF_3)_2COCHF_2$], 51 [M –$(CF_3)_3CO$].

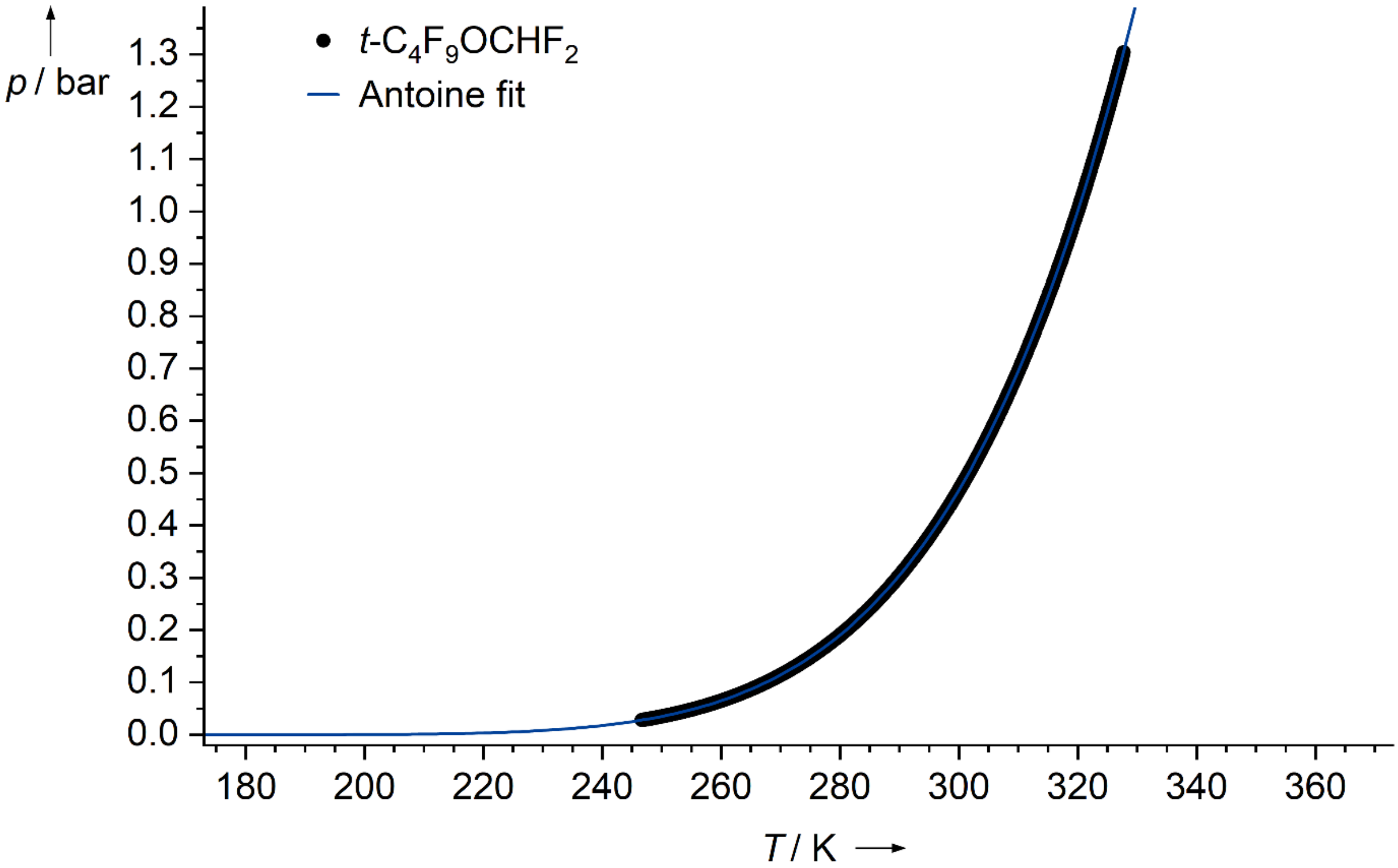

Figure S106. Vapor pressure measurement and Antoine fit of *t*-$C_4F_9OCHF_2$ (Antoine parameter: A = 4.027; B = 1061.5; C = −56.347 for equation: $p = 10^{(A-B/(T+C))}$).

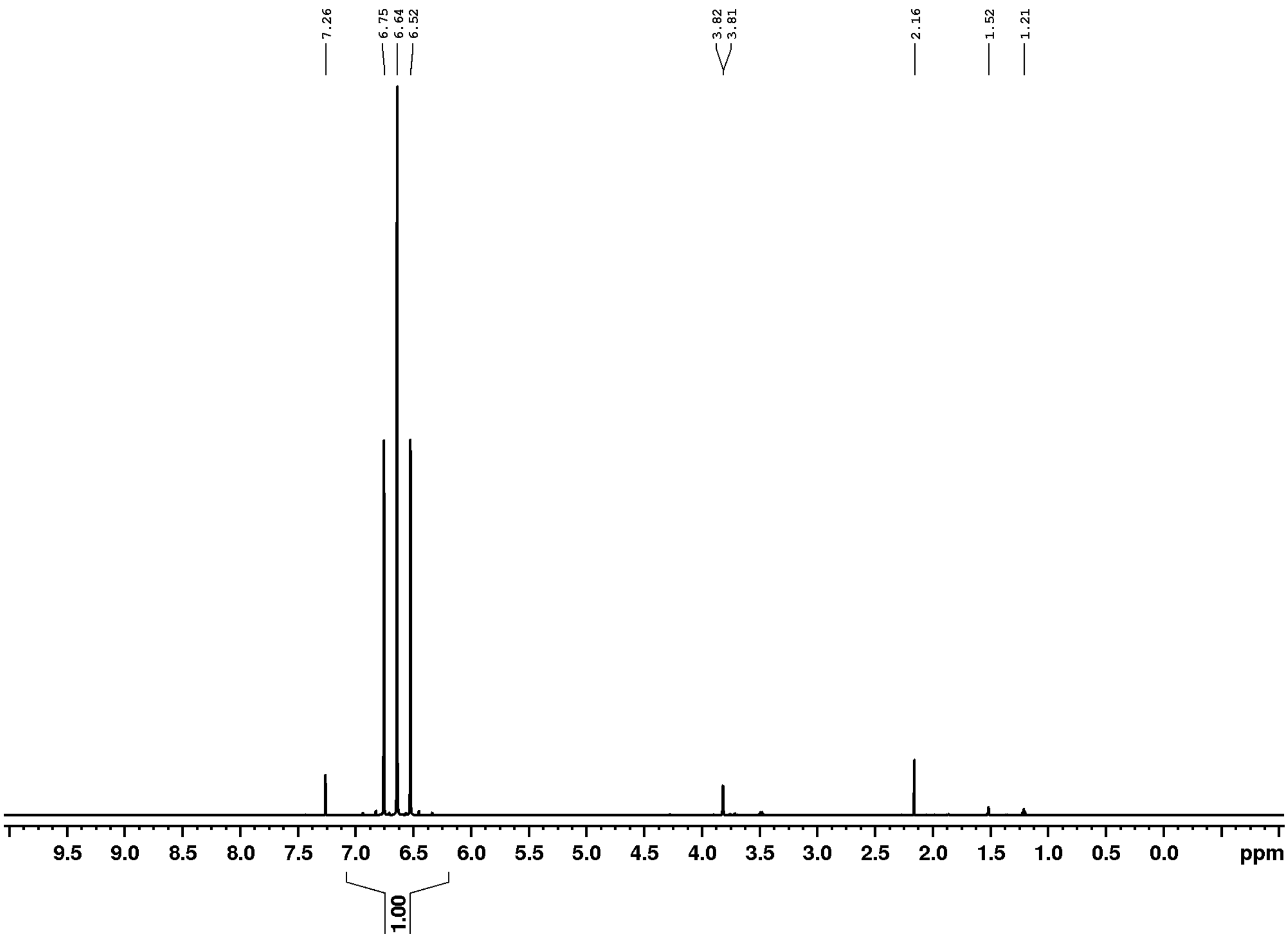

Figure S107. ^1H NMR of $(CF_3)_3COCHF_2$ in $CDCl_3$.

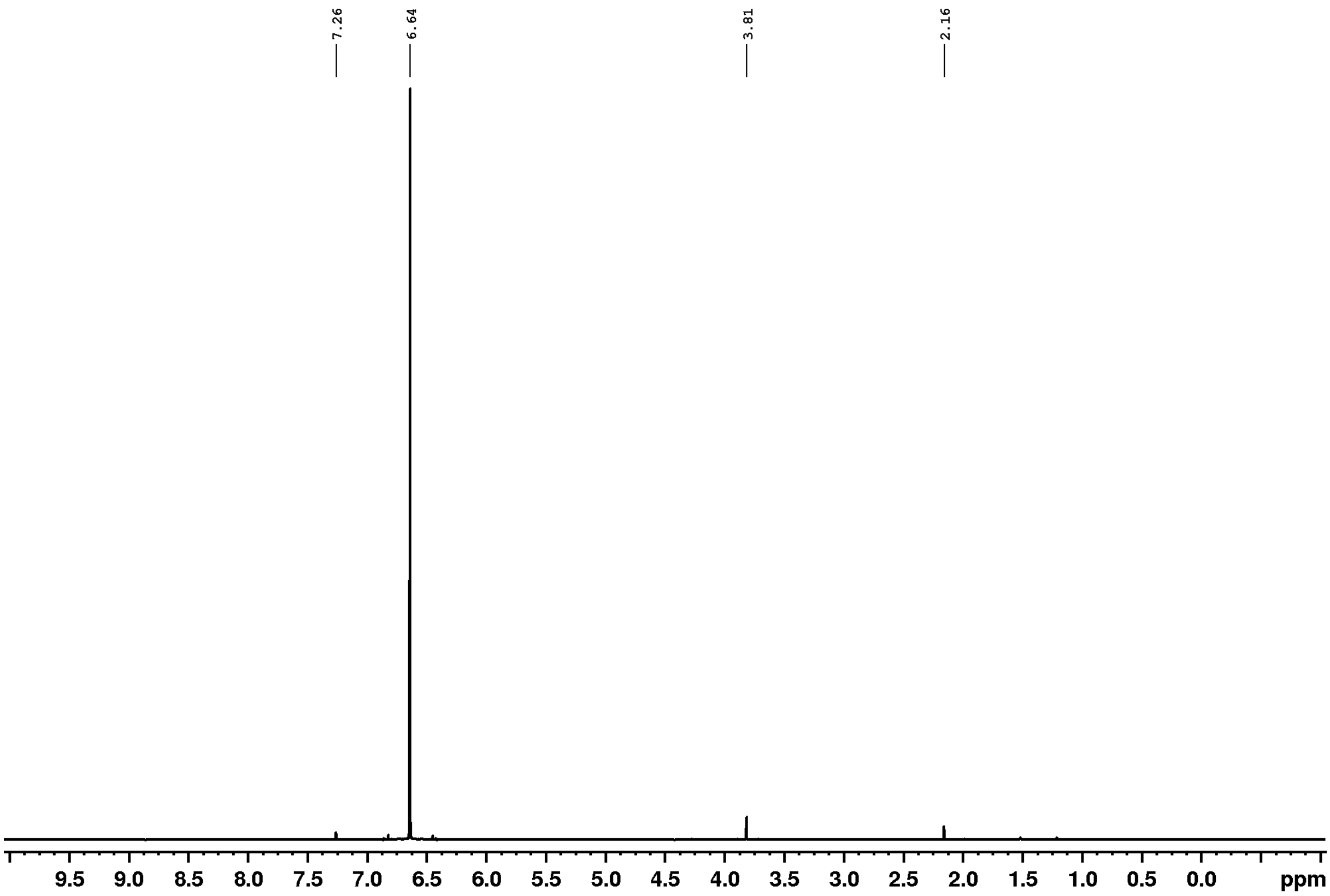

Figure S108. $^{1}H\{^{19}F\}$ NMR of $(CF_3)_3COCHF_2$ in $CDCl_3$.

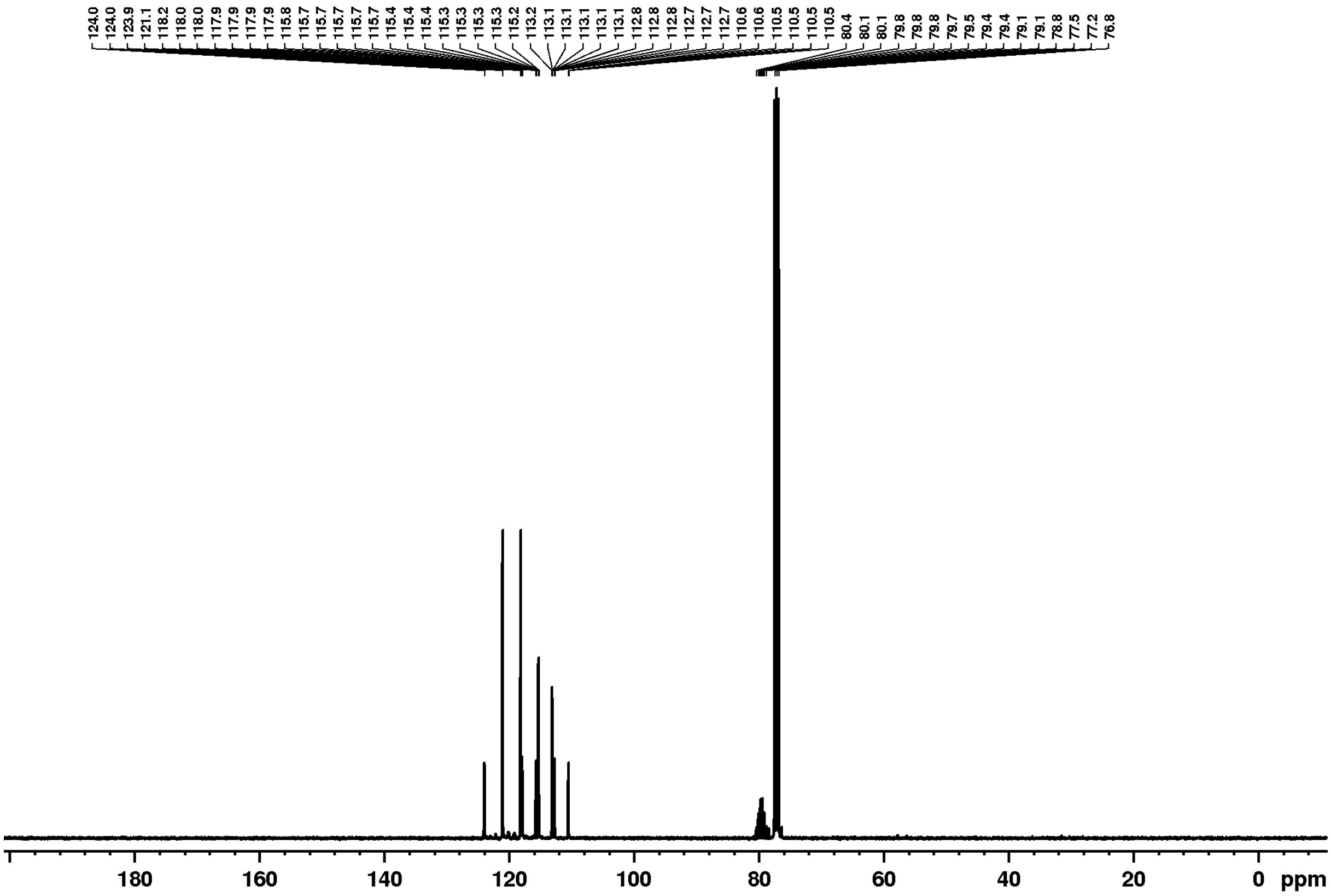

Figure S109. ^{13}C NMR of $(CF_3)_3COCHF_2$ in $CDCl_3$.

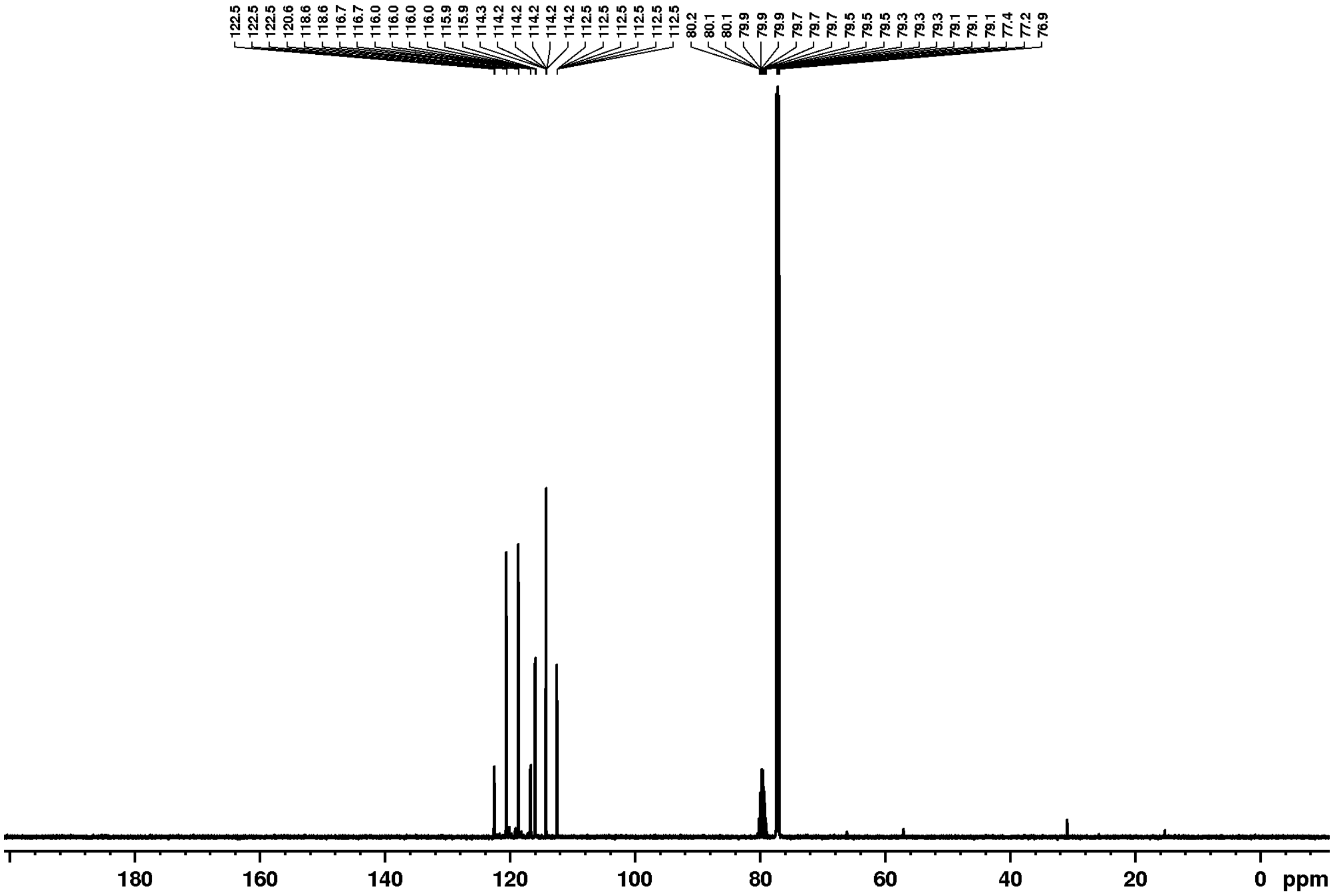

Figure S110. $^{13}C\{^{1}H\}$ NMR of $(CF_3)_3COCHF_2$ in $CDCl_3$.

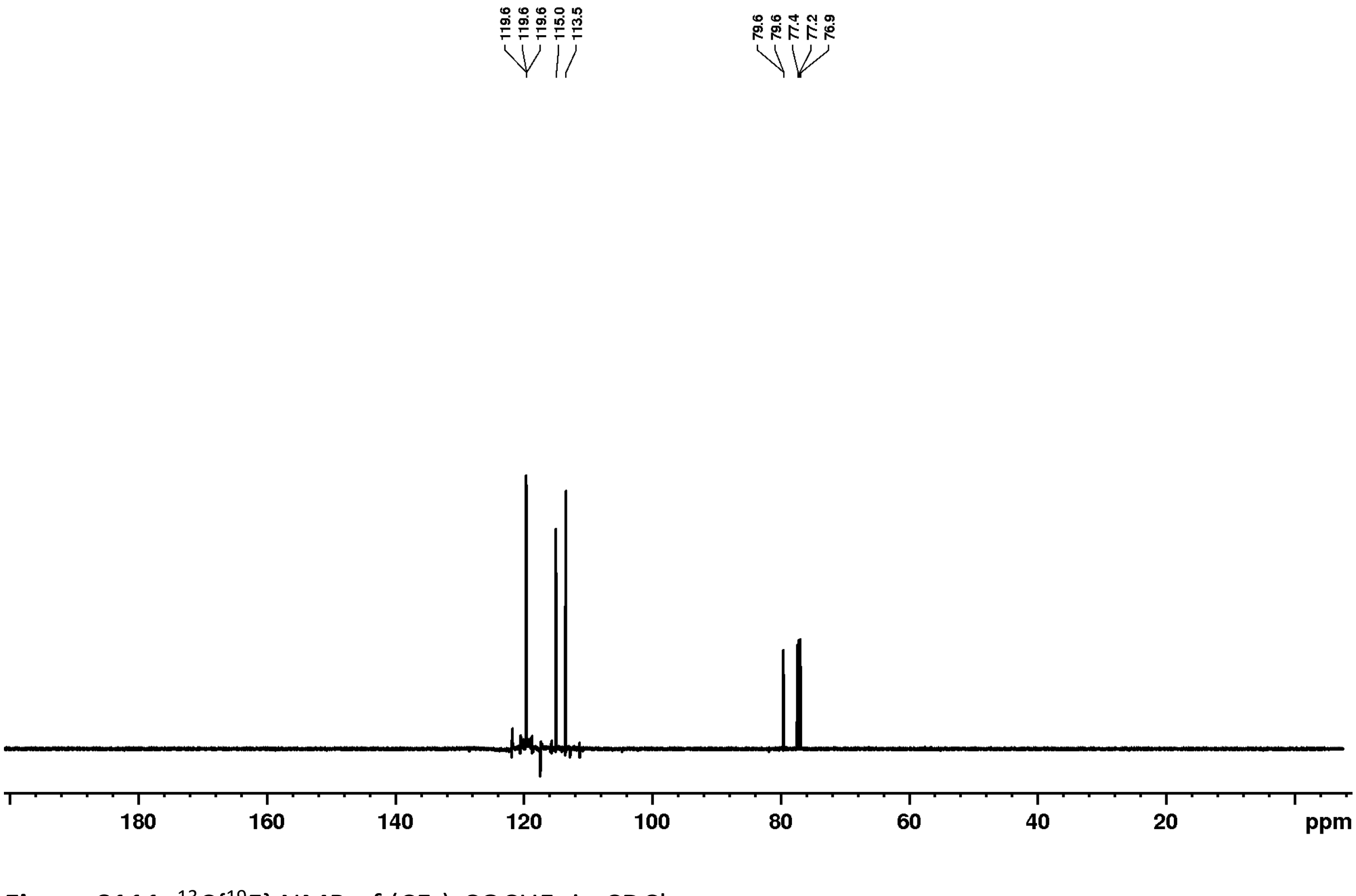

Figure S111. $^{13}C\{^{19}F\}$ NMR of $(CF_3)_3COCHF_2$ in $CDCl_3$.

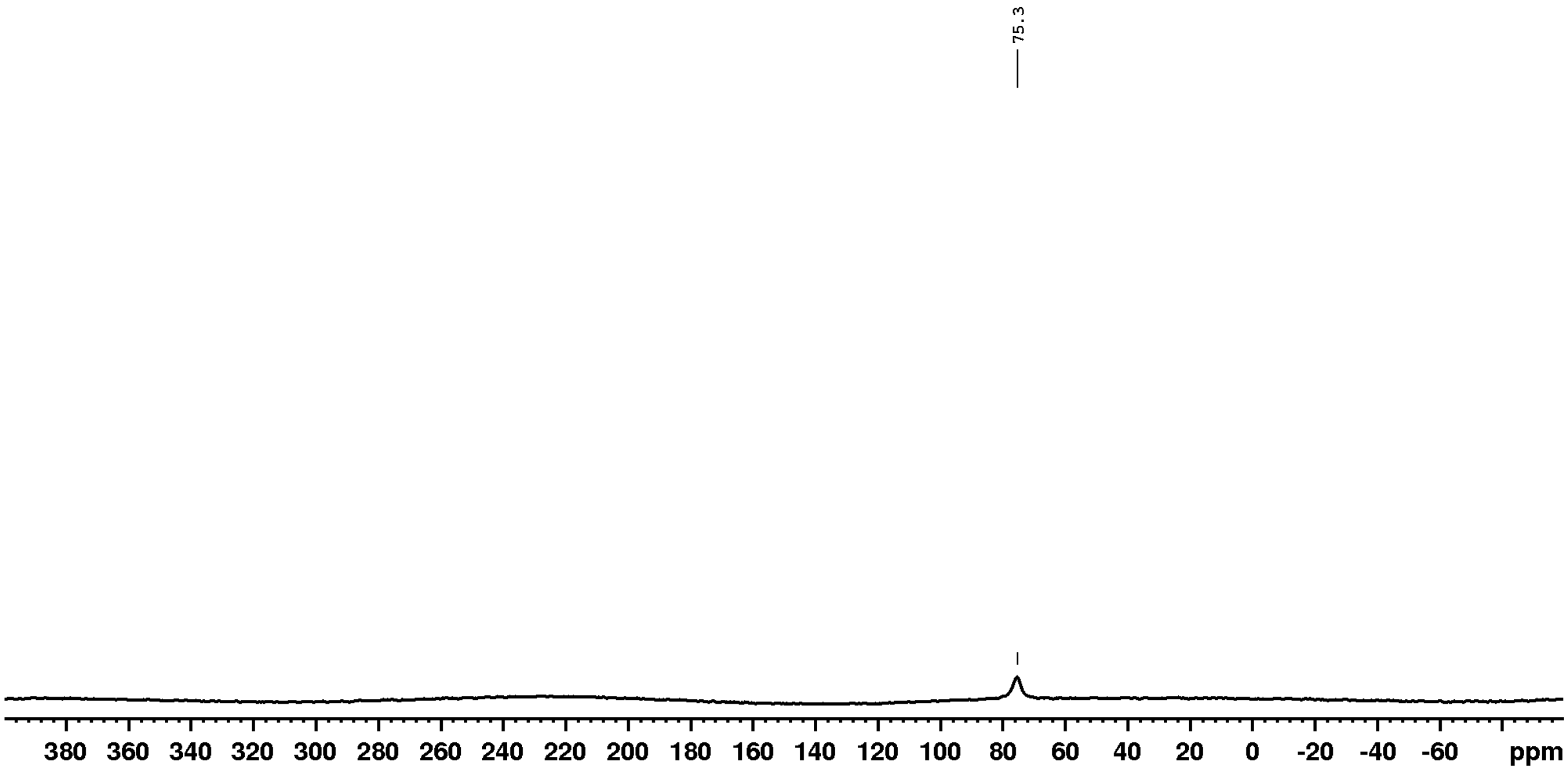

Figure S112. ^{17}O NMR of $(CF_3)_3COCHF_2$ in $CDCl_3$.

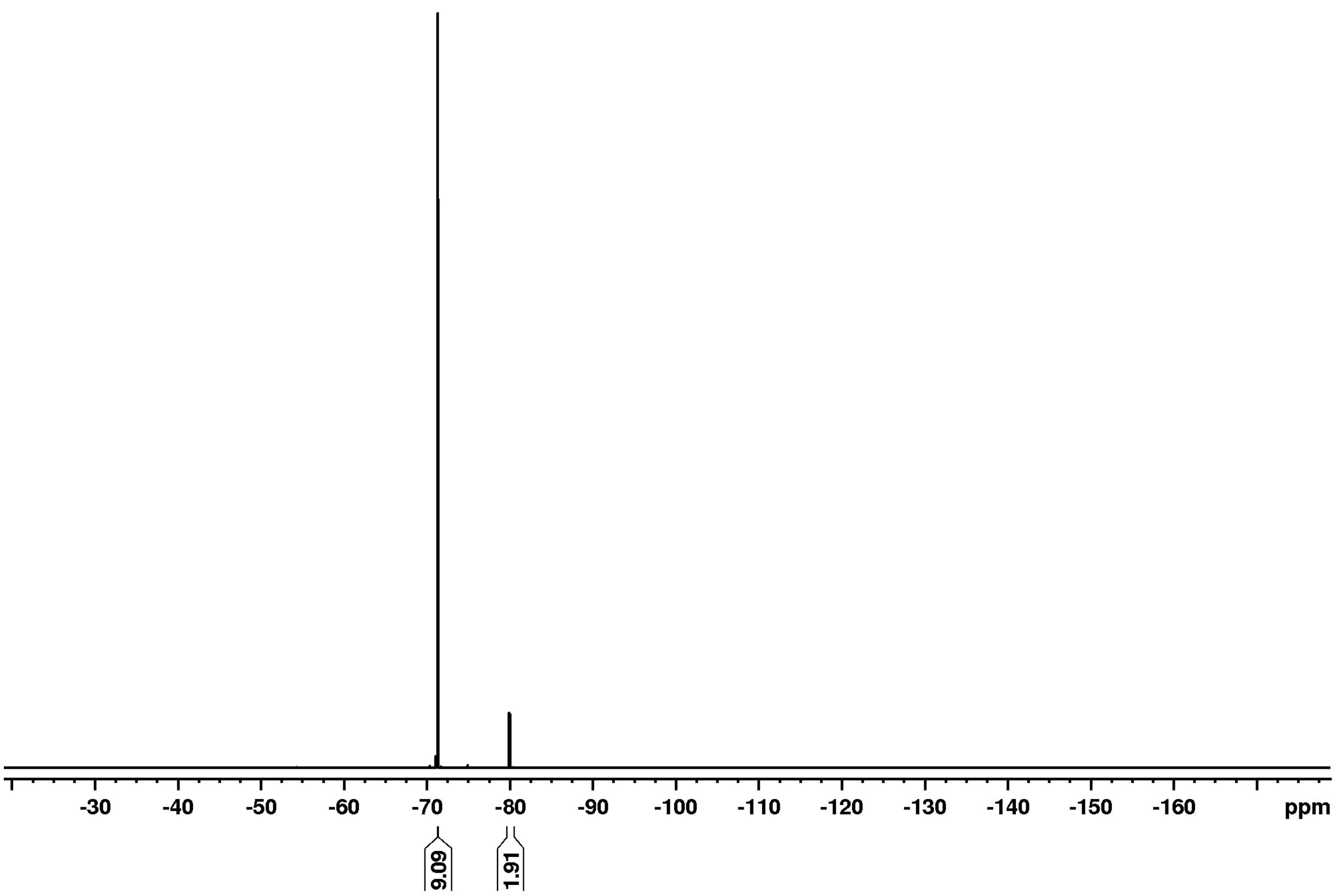

Figure S113. ^{19}F NMR of $(CF_3)_3COCHF_2$ in $CDCl_3$.

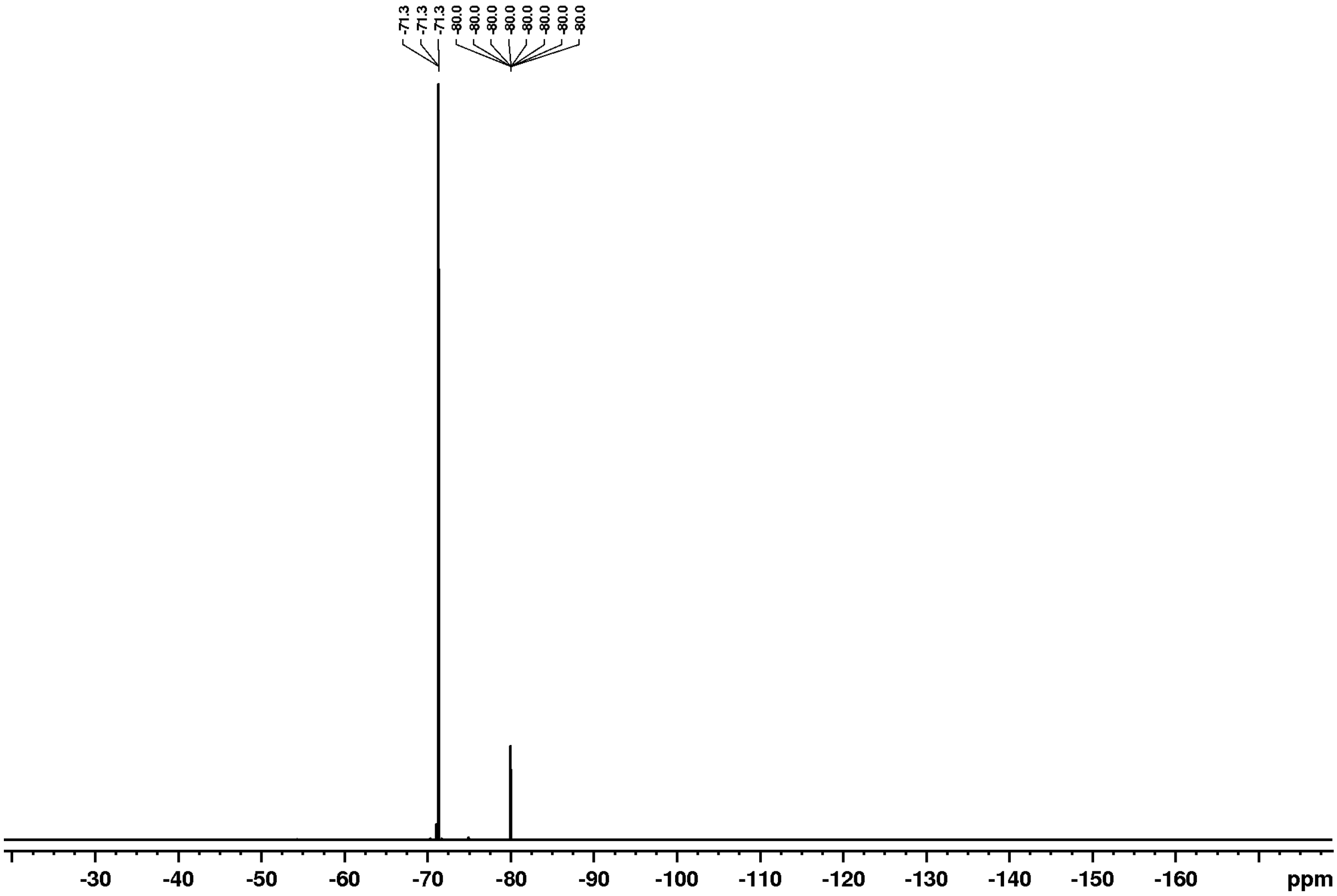

Figure S114. $^{19}F\{^{1}H\}$ NMR of $(CF_3)_3COCHF_2$ in $CDCl_3$.

***t*-$C_4F_9OCF_3$**

Boiling point (distillation): T_{bp} =35 °C at 978 mbar.

Boiling point (vapor pressure): T_{bp} = 36.3 °C at 1013 mbar (Antoine parameter: A = 3.949; B = 994.93; C = −57.118 for equation: $p = 10^{(A-B/(T+C))}$).

Enthalpy of vaporization (T = 298.15 K): $\Delta_{vap}H$ = 29.1 kJ mol^{-1} (for equation: $\Delta_{vap}H = \ln(10)\cdot R\cdot T^2\cdot(B/(T+C)^2)/1000$).

^{13}C NMR (100.7 MHz, $(CH_3)_2CO$): δ = 119.8 (q, 1 C, $(CF_3)_3CO\mathbf{C}F_3$, $^1J(^{19}F, ^{13}C)$ =265.0 Hz), 118.9 (qm, 3C, $(\mathbf{C}F_3)_3COCF_3$, $^1J(^{19}F, ^{13}C)$ = 289.2 Hz), 80.6 (dec, 1C, $(CF_3)_3\mathbf{C}OCF_3$, $^2J(^{19}F, ^{13}C)$ = 32.6 Hz) ppm.

^{13}C{^{1}H} NMR (125.8 MHz, $CDCl_3$): δ = 119.8 (q, 1 C, $(CF_3)_3CO\mathbf{C}F_3$, $^1J(^{19}F, ^{13}C)$ = 266.8 Hz), 199.0 (qm, 3C, $(\mathbf{C}F_3)_3COCF_3$, $^1J(^{19}F, ^{13}C)$ = 290.7 Hz), 80.7 (decq, 1C, $(CF_3)_3\mathbf{C}OCF_3$, $^2J(^{19}F, ^{13}C)$ = 32.6 Hz, $^3J(^{19}F, ^{13}C)$ = 1.1 Hz) ppm.

^{13}C{^{19}F} NMR (125.8 MHz, $CDCl_3$): δ = 119.8 (s, 1 C, $(CF_3)_3CO\mathbf{C}F_3$), 199.0 (s, 3C, $(\mathbf{C}F_3)_3COCF_3$), 80.7 (s, 1C, $(CF_3)_3\mathbf{C}OCF_3$) ppm.

^{17}O NMR (81.4 MHz, $CDCl_3$): δ = 46.4 (s, 1O, $(CF_3)_3C\mathbf{O}CF_3$) ppm.

^{19}F NMR (376.6 MHz, $CDCl_3$): δ = −53.7 (dec, 3F, $(CF_3)_3COC\mathbf{F}_3$, $^5J(^{19}F, ^{19}F)$ = 6.2 Hz), −70.6 (q, 9F, $(C\mathbf{F}_3)_3COC\mathbf{F}_3$, $^5J(^{19}F, ^{19}F)$ = 6.2 Hz) ppm.

GC-MS [t = 0.46 min] m/z$^+$: 285 [M −F], 219 [M −OCF_3], 197 [M −CF_5], 131 [M −CF_3FOCF_3], 69 [M −OC_2F_9].

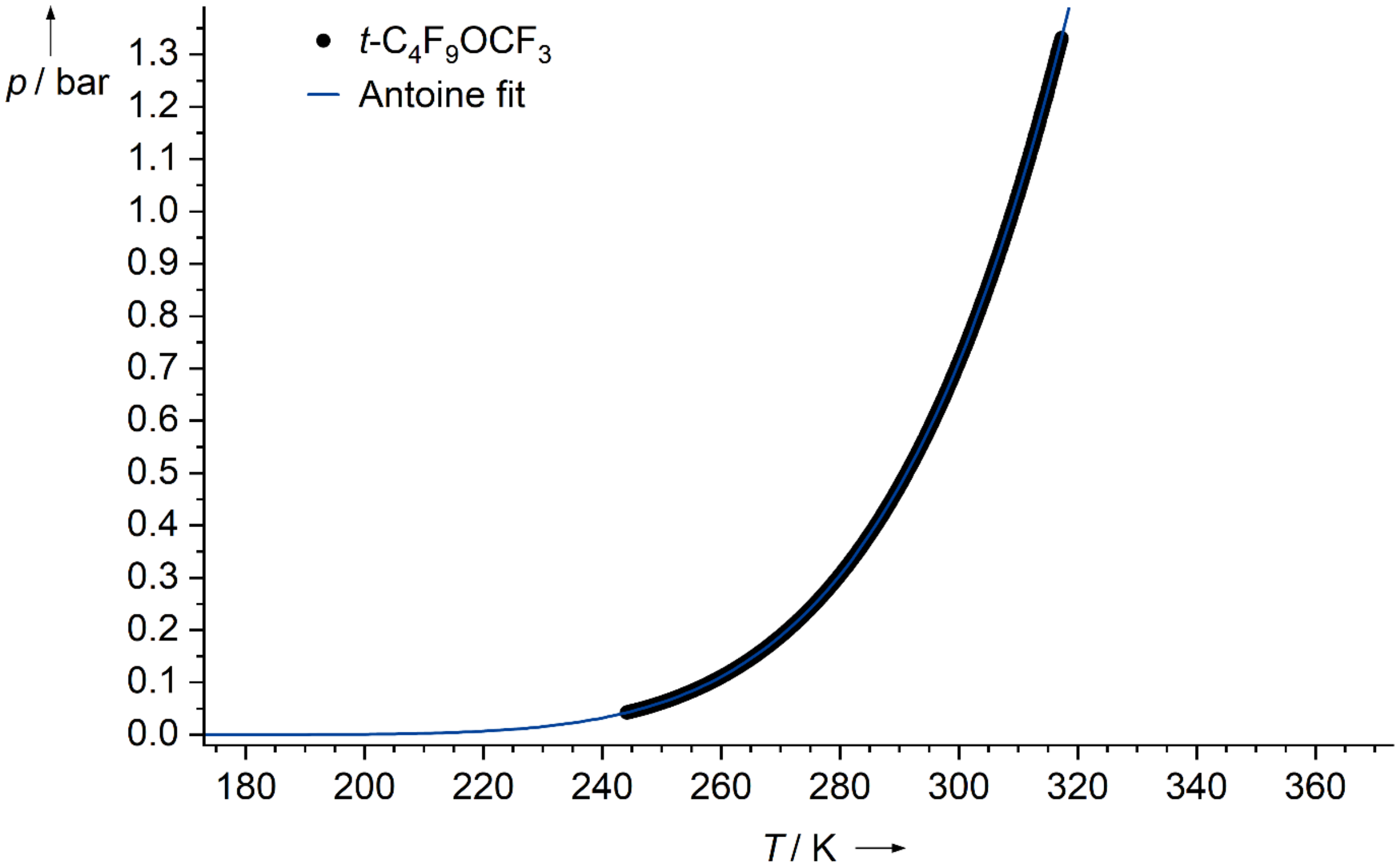

Figure S115. Vapor pressure measurement and Antoine fit of t-$C_4F_9OCF_3$ (Antoine parameter: A = 3.949; B = 994.93; C = −57.118 for equation: $p = 10^{(A-B/(T+C))}$).

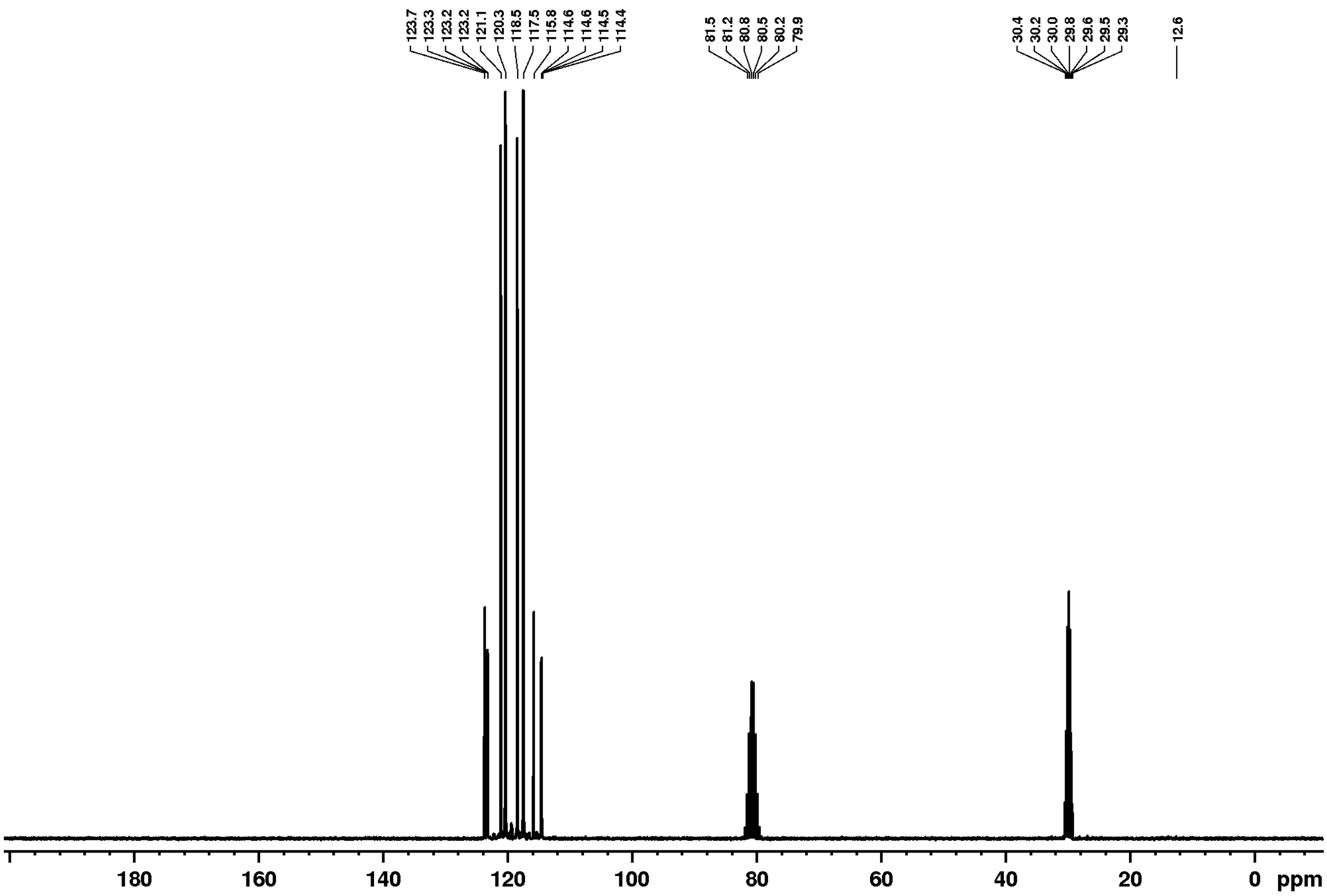

Figure S116. ^{13}C NMR of $(CF_3)_3COCF_3$ measured as neat substance with acetone-d_6 and CCl_3F as external standard.

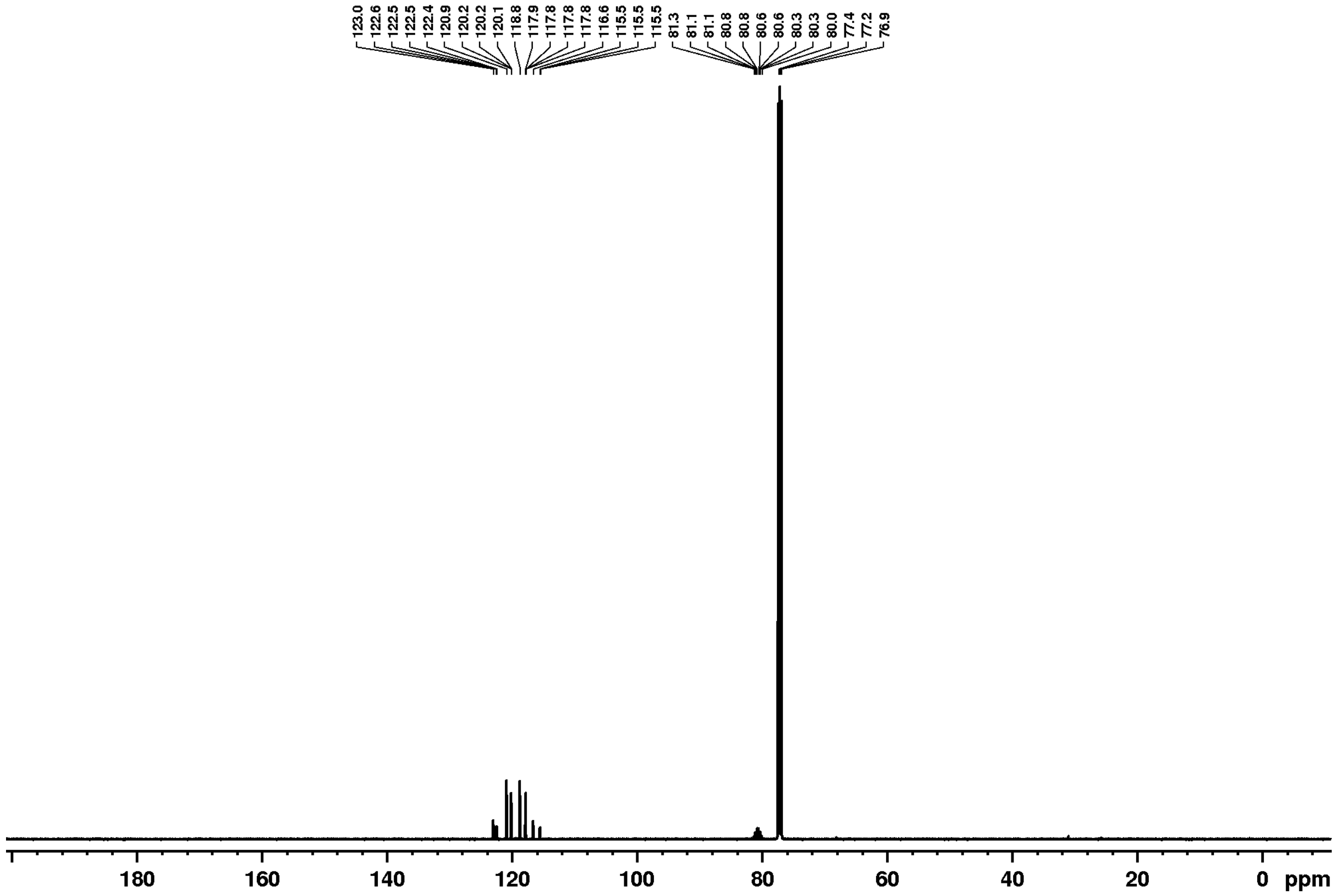

Figure S117. $^{13}C\{^1H\}$ NMR of $(CF_3)_3COCF_3$ in $CDCl_3$.

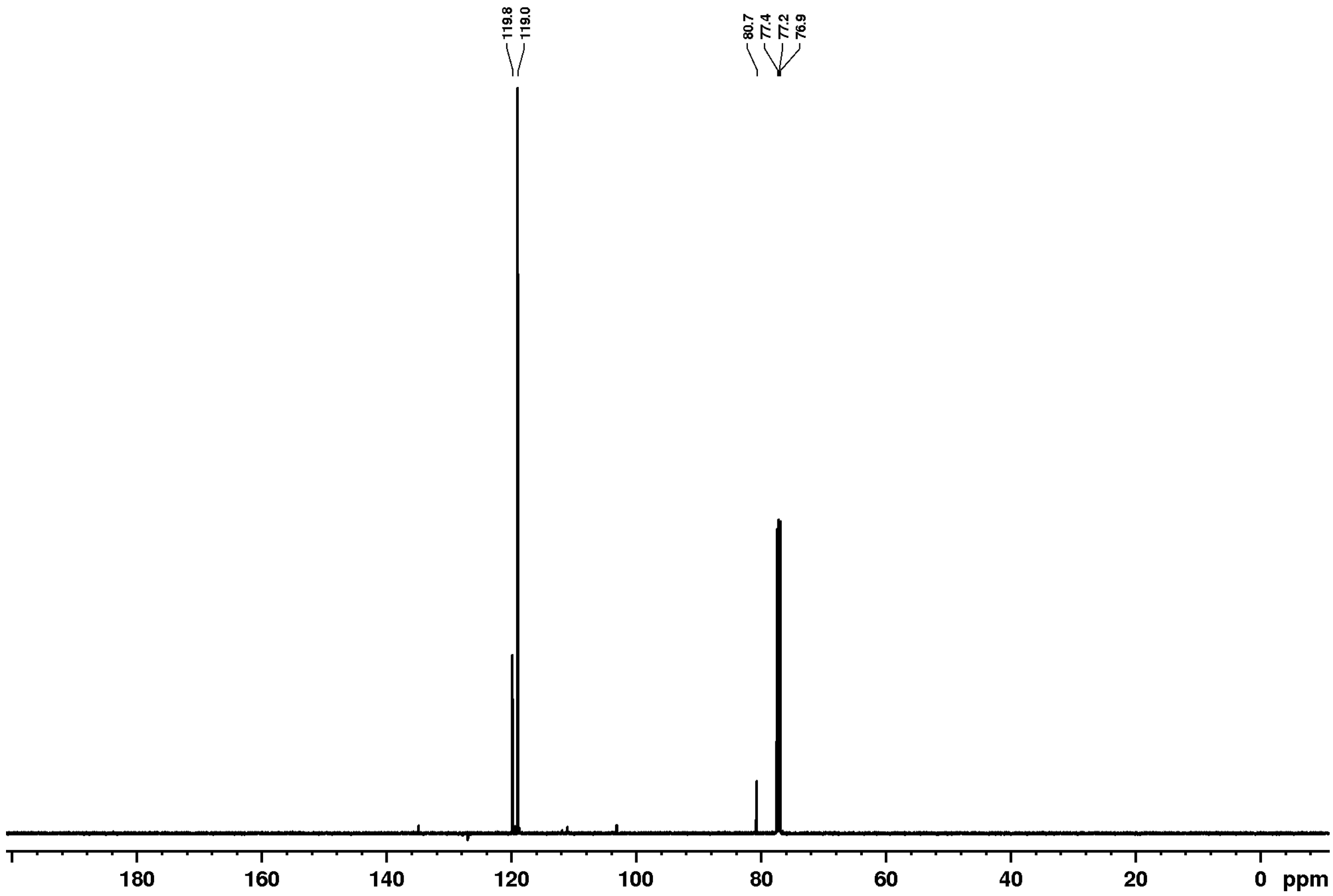

Figure S118. $^{13}C\{^{19}F\}$ NMR of $(CF_3)_3COCF_3$ in $CDCl_3$.

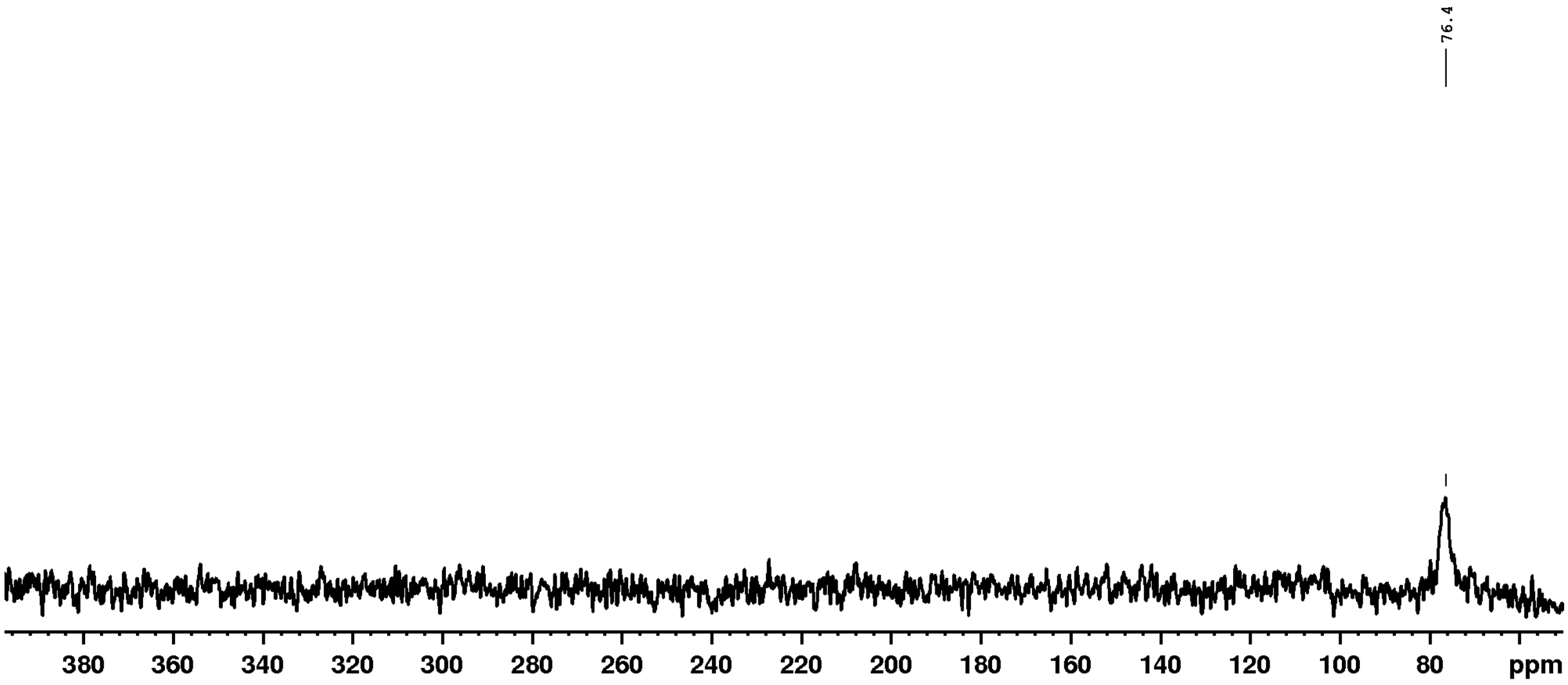

Figure S119. ^{17}O NMR of $(CF_3)_3COCF_3$in $CDCl_3$.

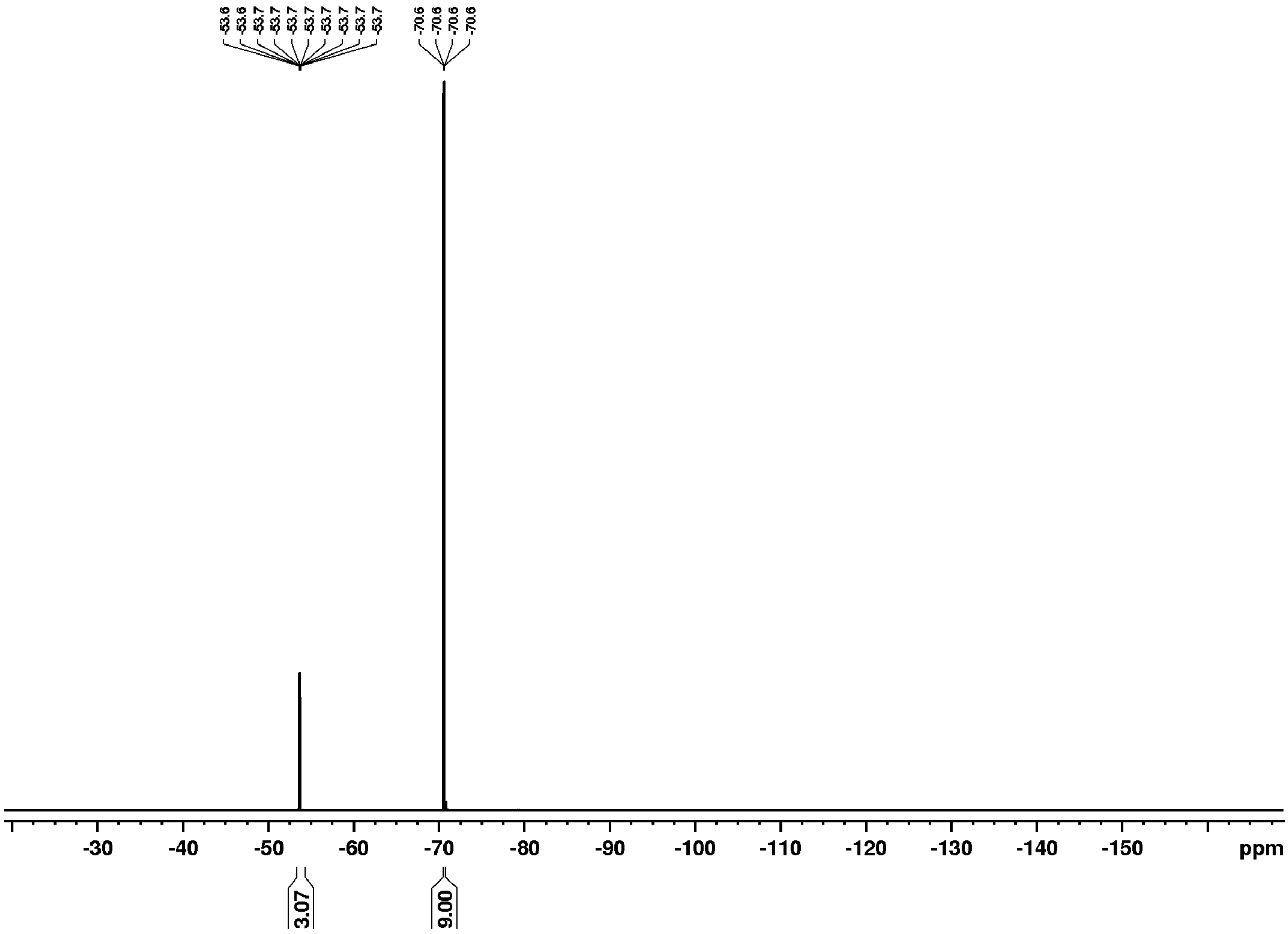

Figure S120. ^{19}F NMR of $(CF_3)_3COCF_3$in $CDCl_3$.

3.5. Electrochemical fluorination of $(CF_3)_2CFCF_2COCH_3$

A 340 mL stainless steel cell with nickel electrodes (effective anode area S = 3.2 dm^2), the construction of which is described elsewhere [15] was filled with anhydrous hydrogen fluoride (280 mL). A constant voltage of 5.0 V was applied for 20 hours. $(CF_3)_2CFCF_2OCH_3$ (990.5 g, 3.961 mol) was added gradually to the pre-electrolyzed anhydrous HF. The gaseous products from the cell were collected in a PTFE trap maintained at −78 °C. The cell was operated at 4.4 – 6.2 V and the current was held between 0.5 and 1.5 A. The cell temperature was maintained at −5 – 7 °C and the temperature of the condenser was kept at −32 °C. After consumption of 585 Ah the reaction was stopped. In total, 787.0 g of crude organic was collected from the cell, consisting of $(CF_3)_2CFCF_2OCF_3$ (56%), $(CF_3)_2CFCF_2OCHF_2$ (29%), $(CF_3)_2CFCF_2OCH_2F$ (5%) and $(CF_3)_2CFCF_2OCH_3$ (10%). From the cold traps, 117.1 g of crude organic was obtained, consisting of $(CF_3)_2CFCF_2OCF_3$ (89%), $(CF_3)_2CFCF_2OCHF_2$ (7%), $(CF_3)_2CFCF_2OCH_2F$ (1%) and $(CF_3)_2CFCF_2OCH_3$ (3%). The aHF from the cell was stored at −78°C. After one day the aHF phase was carefully poured onto ice (300 g). An organic layer (26.1 g) separated from the aqueous phase and was combined with the organic phase obtained from the cell. Both batches of crude organic were stirred with potassium fluoride to remove residual HF. After filtration of the suspension the individual species were isolated by distillation.

***i*-$C_4F_9OCH_3$**

Boiling point (distillation): T_{bp} = 61 °C at 990 mbar.

Boiling point (vapor pressure): T_{bp} = 59.9 °C at 1013 mbar (Antoine parameter: A = 4.006; B = 1096.7; C = −58.935 for equation: $p = 10^{(A-B/(T+C))}$).

Enthalpy of vaporization (T = 298.15 K): $\Delta_{vap}H$ = 32.6 kJ mol^{-1} (for equation: $\Delta_{vap}H = \ln(10)\cdot R\cdot T^2\cdot(B/(T+C)^2)/1000$).

^{1}H NMR (600.2 MHz, $CDCl_3$): δ = 3.74 (s, 3H, $(CF_3)_2CFCF_2OC\mathbf{H}_3$) ppm.

^{1}H{^{19}F} NMR (600.2 MHz, $CDCl_3$): δ = 3.74 (s, 3H, $(CF_3)_2CFCF_2OC\mathbf{H}_3$) ppm.

^{13}C{^{1}H} NMR (150.9 MHz, $CDCl_3$): δ = 119.1 (qdm, 2C, $(\mathbf{C}F_3)_2CFCF_2OCH_3$, $^1J(^{19}F, ^{13}C)$ = 288.8 Hz, $^3J(^{19}F, ^{13}C)$ = 26.6 Hz), 118.1 (td, 1C, $(CF_3)_2CF\mathbf{CF_2}OCH_3$, $^1J(^{19}F, ^{13}C)$ = 273.7 Hz, $^2J(^{19}F, ^{13}C)$ = 24.1 Hz), 88.7 (dsept, 1C, $(CF_3)_2\mathbf{C}FC\mathbf{F_2}OCH_3$, $^1J(^{19}F, ^{13}C)$ = 220.2 Hz, $^2J(^{19}F, ^{13}C)$ = 33.2 Hz), 51.8 (t, 1C, $(CF_3)_2CFC\mathbf{F_2}O\mathbf{C}H_3$, $^3J(^{13}C, ^1H)$ = 6.8 Hz) ppm.

^{13}C{^{19}F} NMR (150.9 MHz, $CDCl_3$): δ = 119.1 (s, 2C, $(\mathbf{C}F_3)_2CFC\mathbf{F_2}OCH_3$), 118.1 (q, 1C, $(CF_3)_2CF\mathbf{CF_2}OCH_3$, $^3J(^{13}C, ^1H)$ = 3.9 Hz), 88.7 (s, 1C, $(CF_3)_2\mathbf{C}FCF_2OCH_3$), 51.8 (q, 1C, $(CF_3)_2CFC\mathbf{F_2}O\mathbf{C}H_3$, $^1J(^{13}C, ^1H)$ = 148.9 Hz) ppm.

^{17}O NMR (81.4 MHz, $CDCl_3$): δ = 44.3 (s, 1O, $(CF_3)_2CFC\mathbf{F_2}\mathbf{O}CH_3$) ppm.

^{19}F NMR (564.7 MHz, $CDCl_3$): δ = −73.9 (dt, 6F, $(C\mathbf{F}_3)_2CFC\mathbf{F_2}OCH_3$, $^3J(^{19}F, ^{19}F)$ = 6.1 Hz, $^4J(^{19}F, ^{19}F)$ = 9.7 Hz), −82.3 (m, 2F, $(CF_3)_2CFC\mathbf{F_2}OCH_3$), −187.7 (m, 1F, $(CF_3)_2C\mathbf{F}C\mathbf{F_2}OCH_3$) ppm.

^{19}F{^{1}H} NMR (564.7 MHz, $CDCl_3$): δ = −73.9 (dt, 6F, $(C\mathbf{F}_3)_2CFC\mathbf{F_2}OCH_3$, $^3J(^{19}F, ^{19}F)$ = 6.1 Hz, $^4J(^{19}F, ^{19}F)$ = 9.7 Hz), −82.3 (m, 2F, $(CF_3)_2CFC\mathbf{F_2}OCH_3$), −187.7 (m, 1F, $(CF_3)_2C\mathbf{F}C\mathbf{F_2}OCH_3$) ppm.**GC-MS** [t = 0.49 min] m/z^+: 249 $[M]^+$, 231 [M −F], 219 [M $-OCH_3$], 197 [M $-F_2CH_3$], 169 [M $-CF_2OCH_3$], 150 [M $-FCF_2OCH_3$], 131 [M $-CF_3FOCH_3$], 100 [M $-CF_3CF_2OCH_3$], 81 [M $-(CF_3)_2CF_2$], 69 [M $-CF_3CFCF_2OCH_3$].

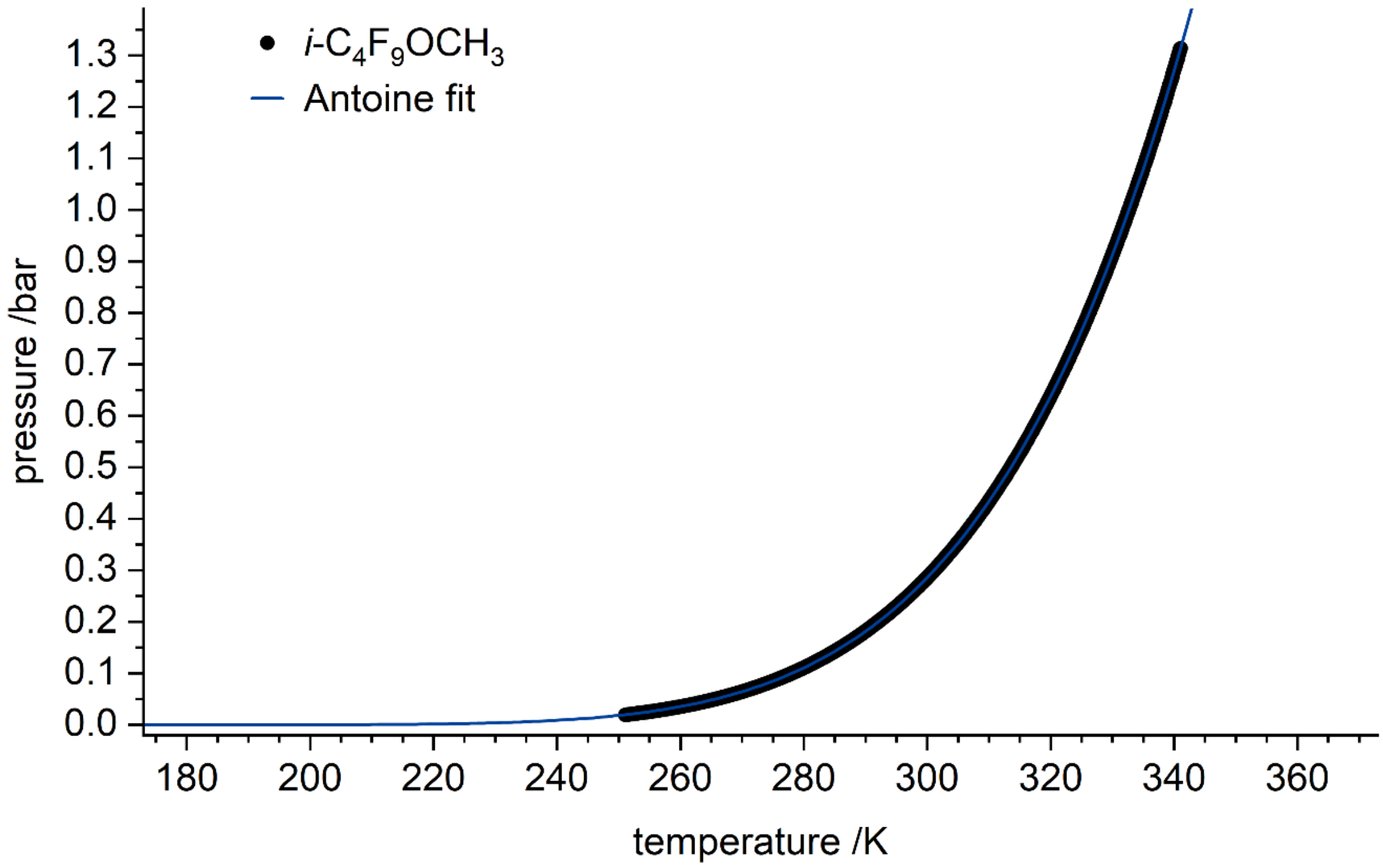

Figure S121. Vapor pressure measurement and Antoine fit of i-$C_4F_9OCH_3$ (Antoine parameter: A = 4.006; B = 1096.7; C = −58.935for equation: $p = 10^{(A-B/(T+C))}$).

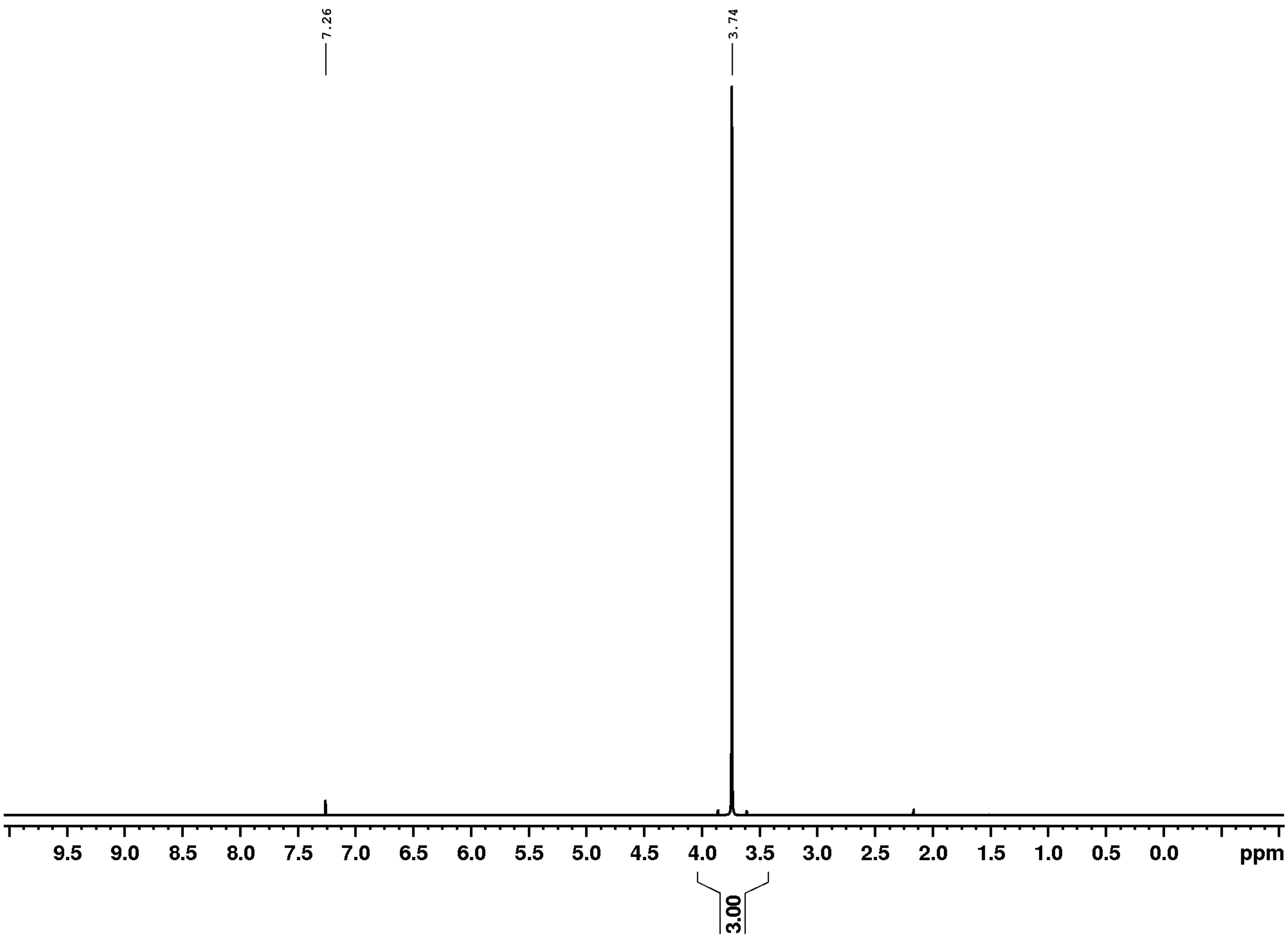

Figure S122. ^{1}H NMR of $(CF_3)_2CFCF_2OCH_3$ in $CDCl_3$.

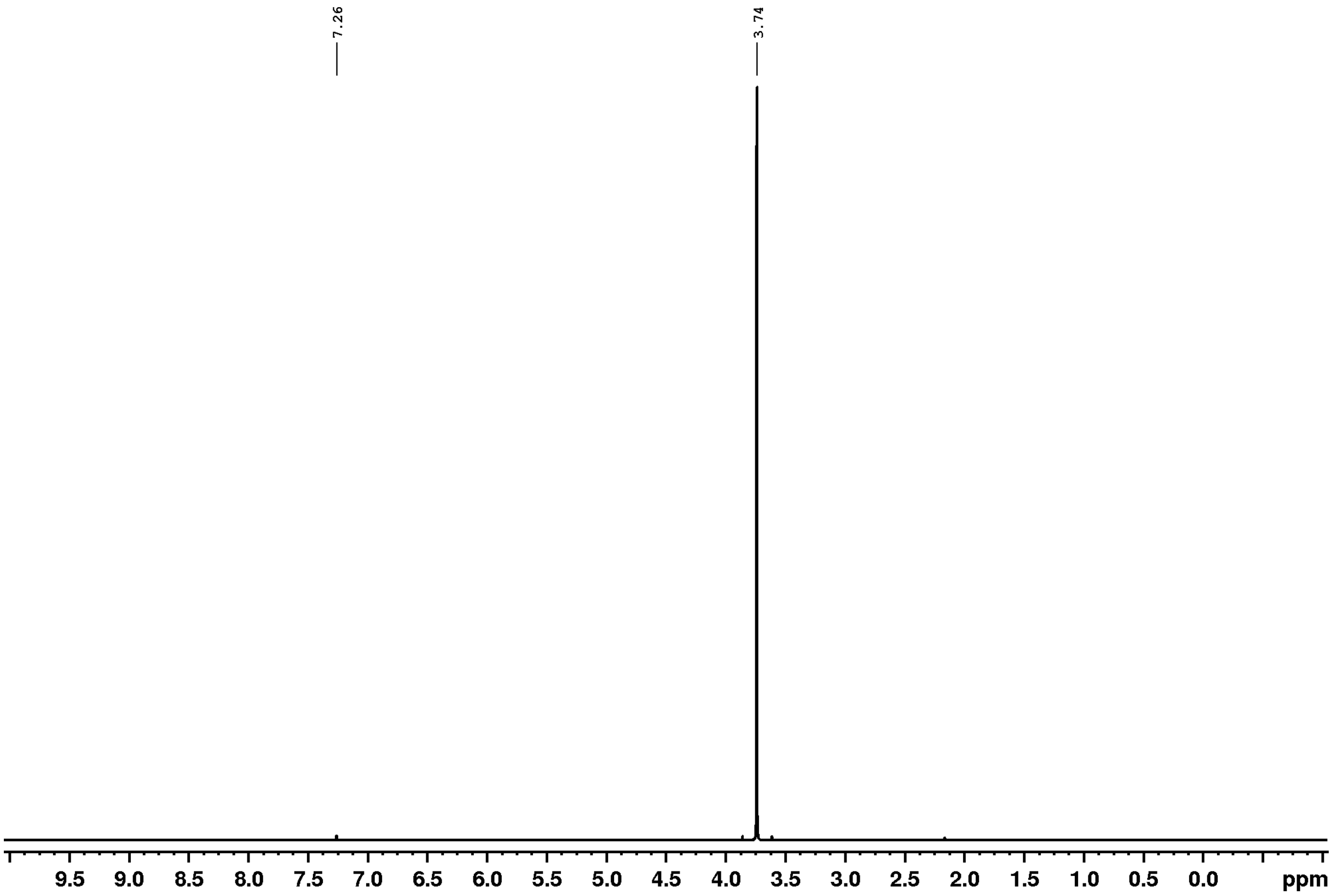

Figure S123. $^{1}H\{^{19}F\}$ NMR of $(CF_3)_2CFCF_2OCH_3$ in $CDCl_3$.

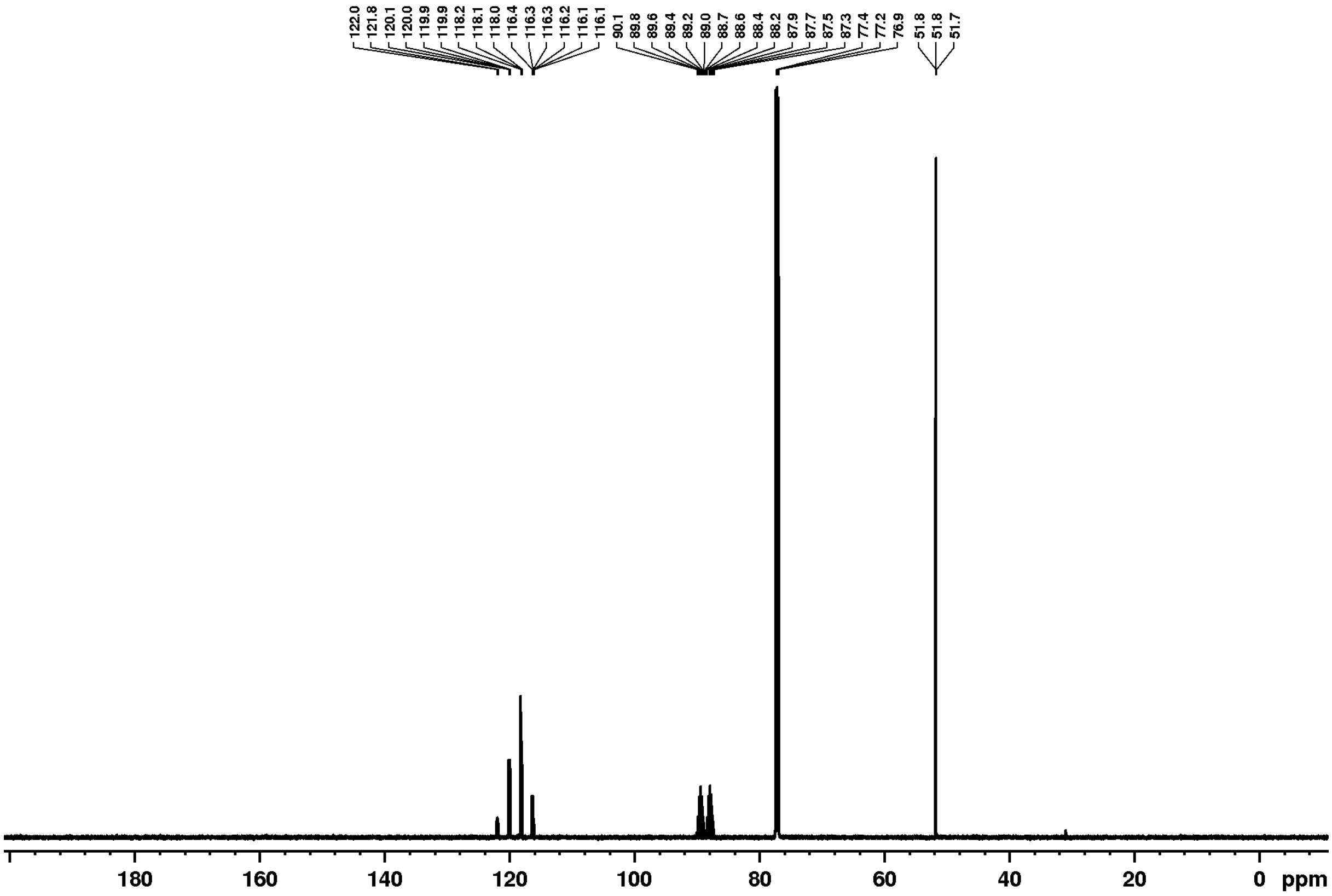

Figure S124. $^{13}C\{^{1}H\}$ NMR of $(CF_3)_2CFCF_2OCH_3$ in $CDCl_3$.

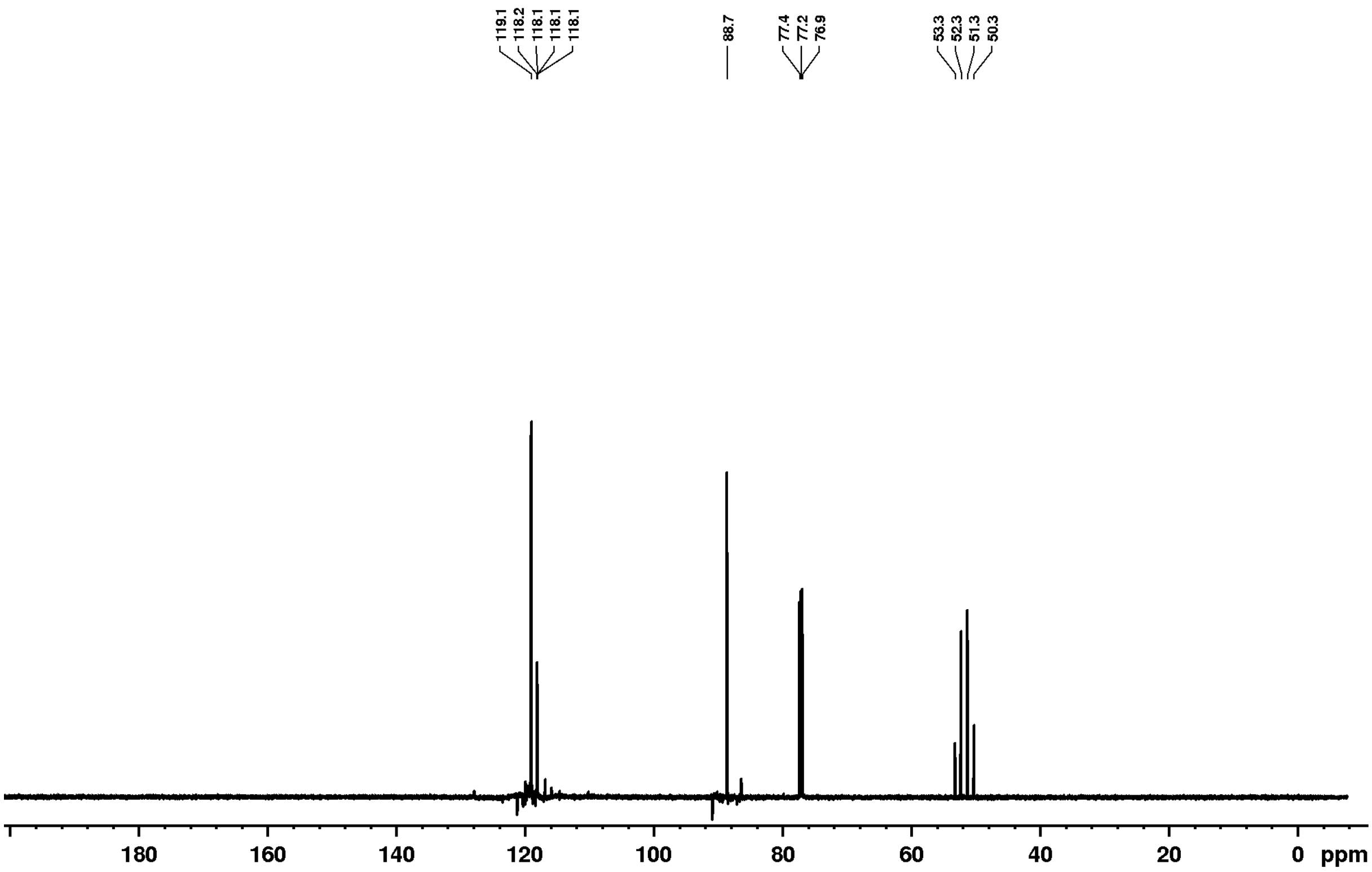

Figure S125. $^{13}C\{^{19}F\}$ NMR of $(CF_3)_2CFCF_2OCH_3$ in $CDCl_3$.

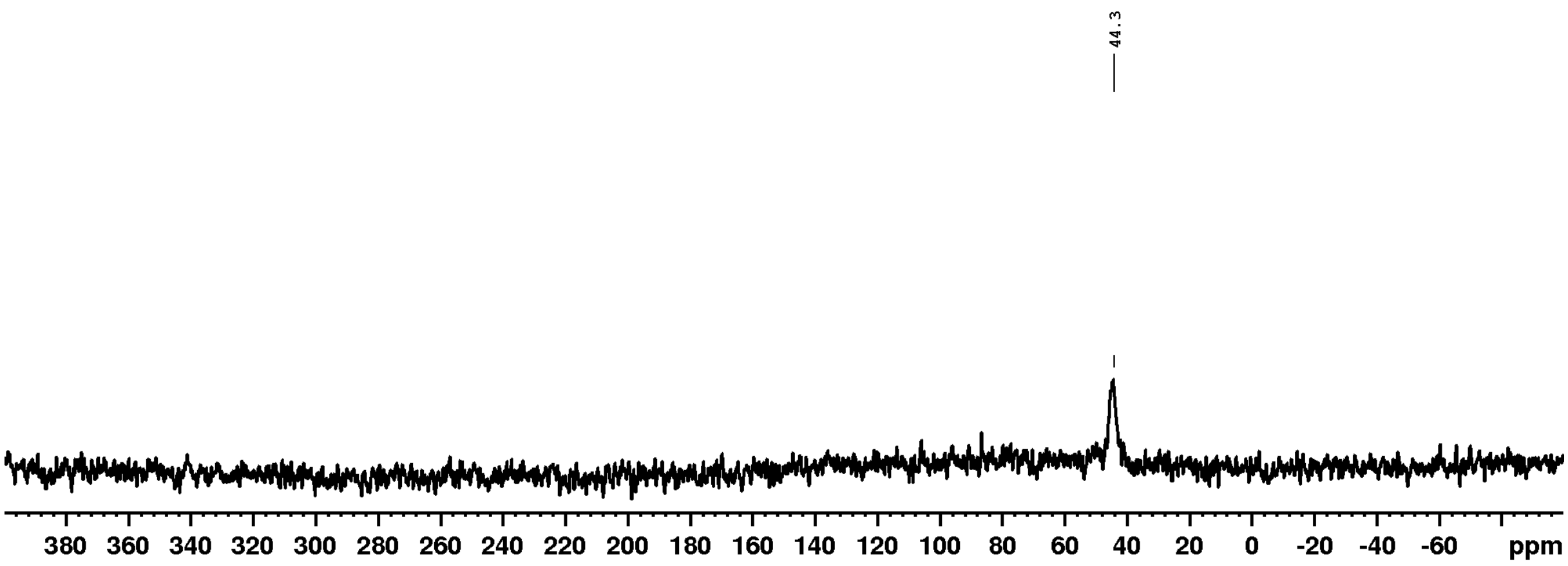

Figure S126. ^{17}O NMR of $(CF_3)_2CFCF_2OCH_3$ in $CDCl_3$.

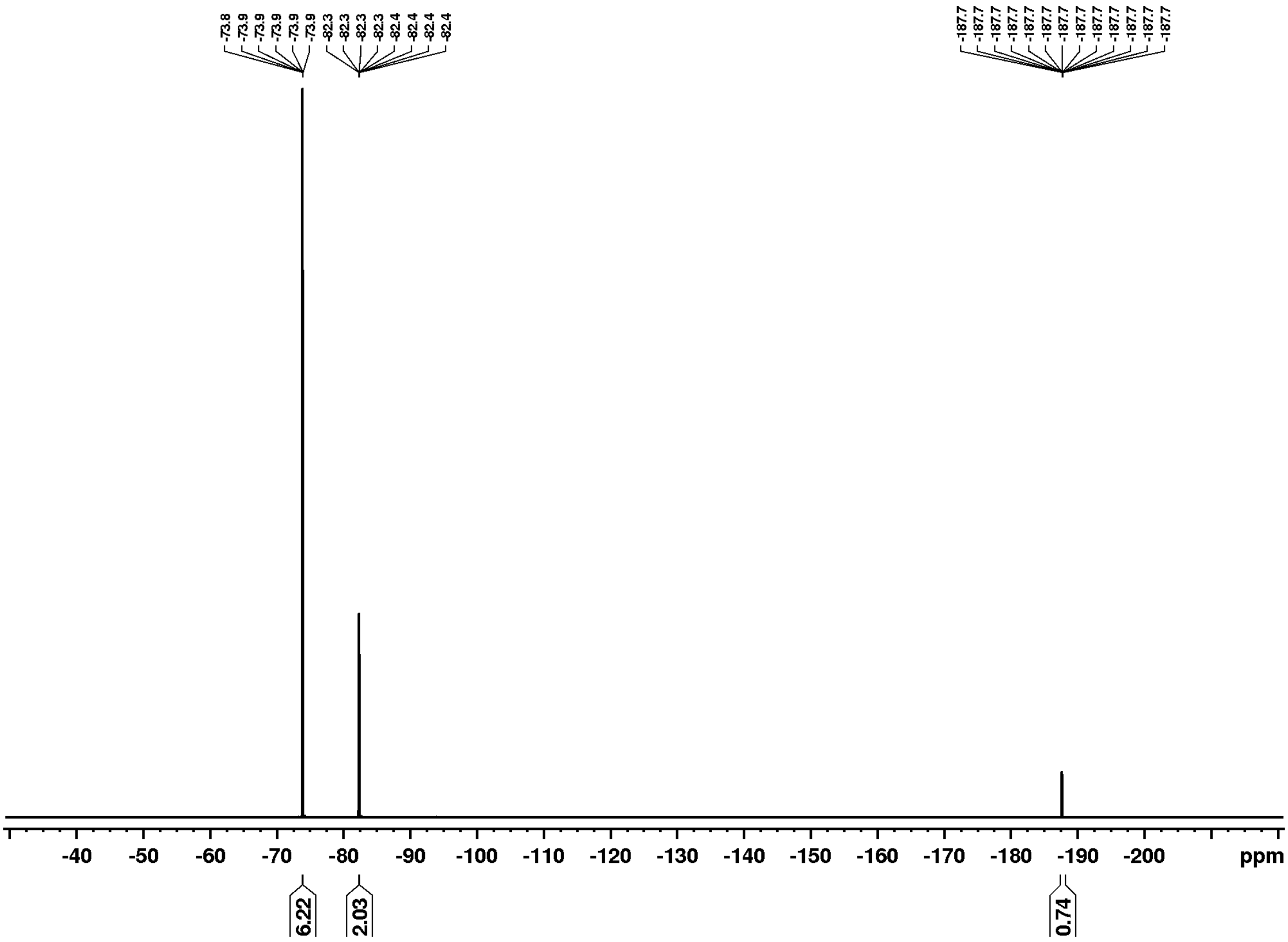

Figure S127. ^{19}F NMR of $(CF_3)_2CFCF_2OCH_3$ in $CDCl_3$.

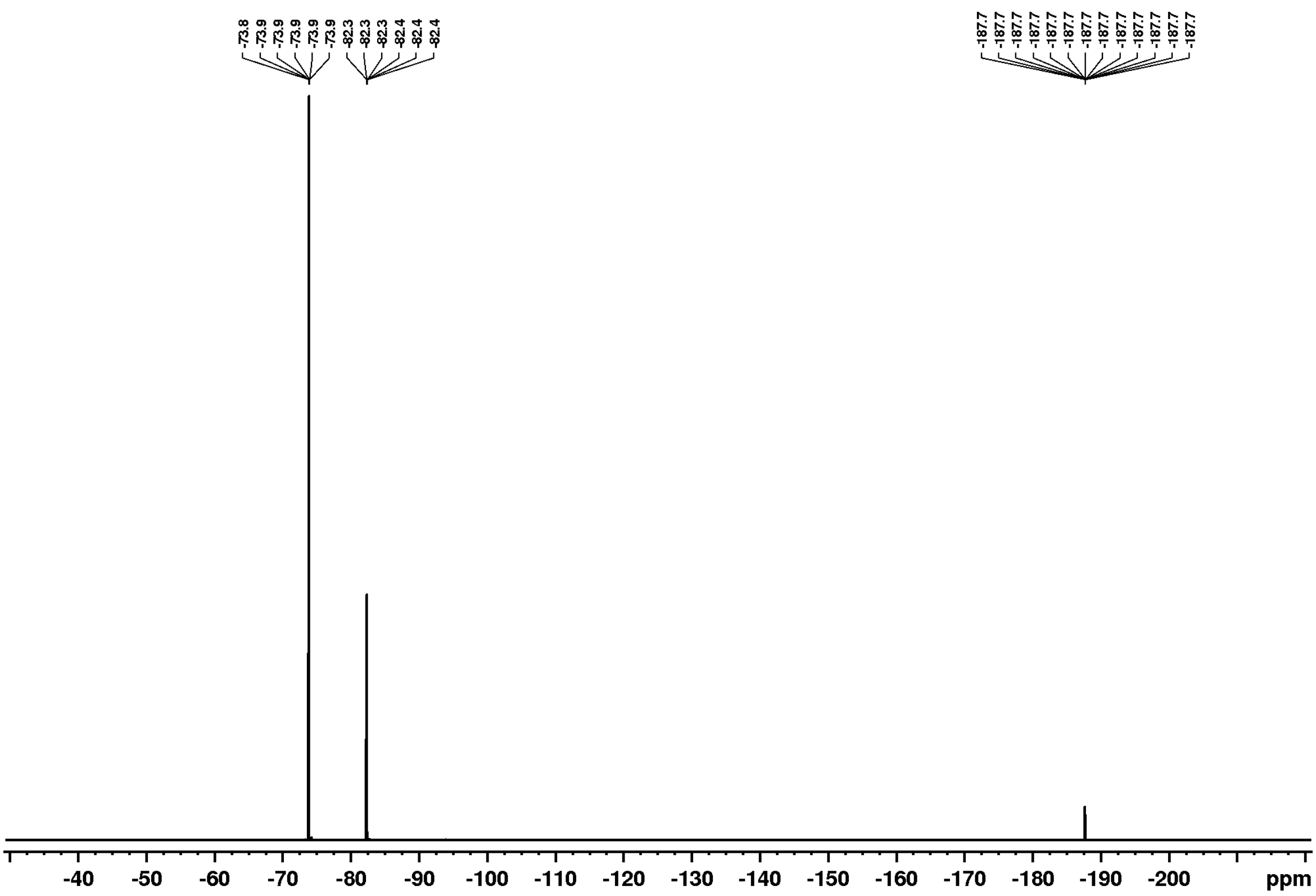

Figure S128. ^{19}F{^{1}H} NMR of $(CF_3)_2CFCF_2OCH_3$ in $CDCl_3$.

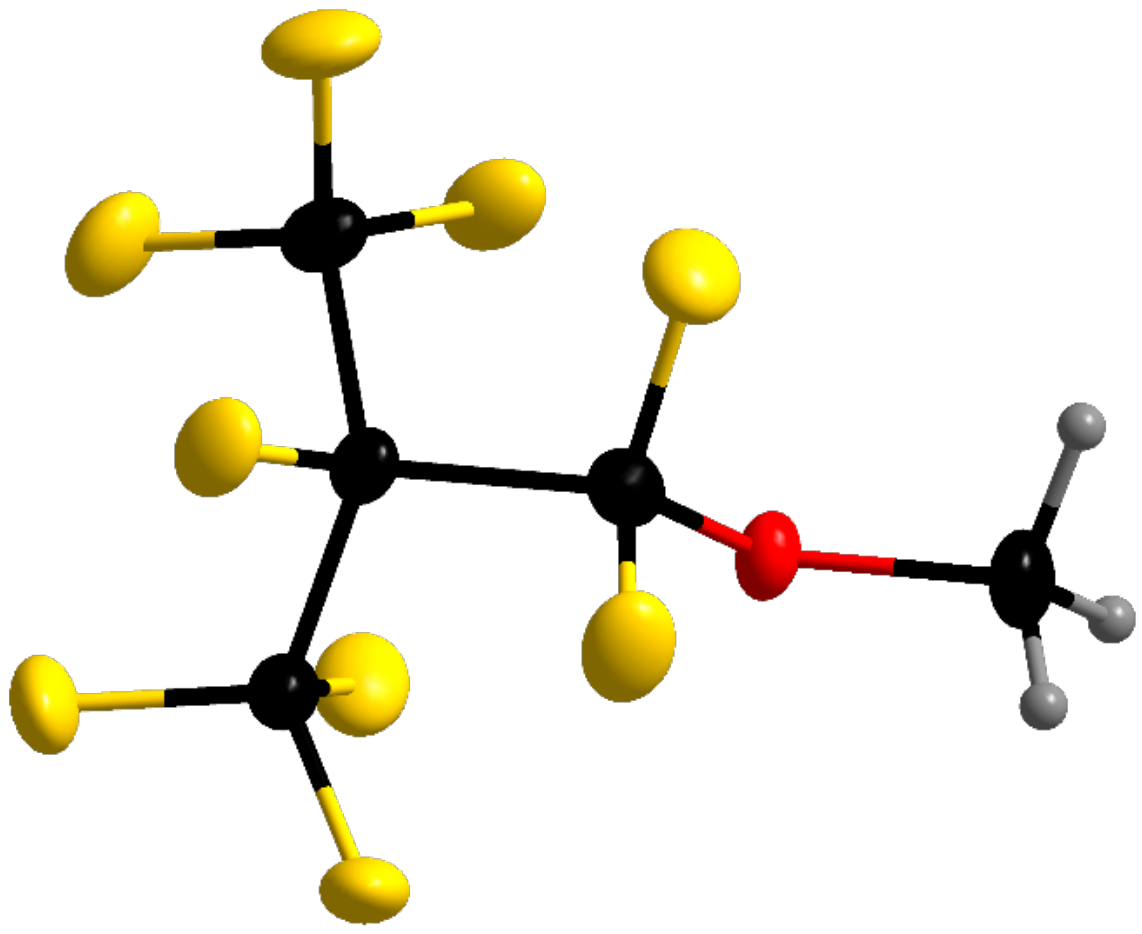

Figure S 129. Solid state structure of $(CF_3)_2CFCF_2OCH_3$.

***i*-$C_4F_9OCH_2F$**

Boiling point (distillation): T_{bp} = 67 °C at 984 mbar.

Boiling point (vapor pressure): T_{bp} = 67.8 °C at 1013 mbar (Antoine parameter: A = 4.144; B = 1167.7; C = −58.727for equation: $p = 10^{(A-B/(T+C))}$).

Enthalpy of vaporization (T = 298.15 K): $\Delta_{vap}H$ = 34.7 kJ mol^{-1} (for equation: $\Delta_{vap}H = \ln(10)\cdot R\cdot T^2\cdot(B/(T+C)^2)/1000$).

^{1}H NMR (600.2 MHz, $CDCl_3$): δ = 5.64 (d, 2H, $(CF_3)CFCF_2OC\mathbf{H}_2F$, $^2J(^{19}F, ^1H)$ = 50.7 Hz) ppm.**^{1}H{^{19}F} NMR** (600.2 MHz, $CDCl_3$): δ = 5.64 (s, 2H, $(CF_3)CFCF_2OC\mathbf{H}_2F$) ppm.

^{13}C{^{1}H} NMR (150.9 MHz, $CDCl_3$): δ = 118.9 (qdm, 2C, $(\mathbf{C}F_3)_2CFCF_2OCH_2F$, $^1J(^{19}F, ^{13}C)$ = 289.5 Hz, $^2J(^{19}F, ^{13}C)$ = 26.4 Hz), 117.7 (td, 1C, $(CF_3)_2CF\mathbf{C}F_2OCH_2F$, $^1J(^{19}F, ^{13}C)$ = 280.4 Hz, $^2J(^{19}F, ^{13}C)$ = 25.2 Hz), 95.7 (dt, 1C, $(CF_3)_2CFCF_2O\mathbf{C}H_2F$, $^1J(^{19}F, ^{13}C)$ = 231.3 Hz, $^3J(^{19}F, ^{13}C)$ = 6.1 Hz), 88.5 (dsept, 1C, $(CF_3)_2\mathbf{C}FCF_2OCH_2F$, $^1J(^{19}F, ^{13}C)$ = 221.6 Hz, $^2J(^{19}F, ^{13}C)$ = 33.1 Hz) ppm.

^{13}C{^{19}F} NMR (150.9 MHz, $CDCl_3$): δ = 118.9 (s, 2C, $(\mathbf{C}F_3)_2CFCF_2OCH_2F$), 117.7 (t, 1C, $(CF_3)_2CF\mathbf{C}F_2OCH_2F$, $^3J(^{13}C, ^1H)$ = 6.1 Hz), 95.7 (t, 1C, $(CF_3)_2CFCF_2O\mathbf{C}H_2F$, $^1J(^{13}C, ^1H)$ = 180.7 Hz), 88.5 (s, 1C, $(CF_3)_2\mathbf{C}FCF_2OCH_2F$) ppm.

^{17}O NMR (81.4 MHz, $CDCl_3$): δ = 105.5 (s, 1O, $(CF_3)_2CFCF_2\mathbf{O}CH_2F$) ppm.

^{19}F NMR (564.7 MHz, $CDCl_3$): δ = −73.7 (dt, 6F, $(C\mathbf{F}_3)_2CFCF_2OCH_2F$, $^3J(^{19}F, ^{19}F)$ = 6.2 Hz, $^4J(^{19}F, ^{19}F)$ = 9.6 Hz), −79.4 (dsept, 2F, $(CF_3)_2CFC\mathbf{F}_2OCH_2F$, $^3J(^{19}F, ^{19}F)$ = 19.1 Hz, $^4J(^{19}F, ^{19}F)$ = 9.4 Hz), −154.9 (tt, 1F, $(CF_3)_2CFCF_2OCH_2\mathbf{F}$, $^2J(^{19}F, ^1H)$ = 50.7 Hz, $^4J(^{19}F, ^{19}F)$ = 9.7 Hz), −187.9 (m, $(CF_3)_2C\mathbf{F}CF_2OCH_2F$) ppm.

^{19}F{^{1}H} NMR (564.7 MHz, $CDCl_3$): δ = −73.7 (dt, 6F, $(C\mathbf{F}_3)_2CFCF_2OCH_2F$, $^3J(^{19}F, ^{19}F)$ = 6.2 Hz, $^4J(^{19}F, ^{19}F)$ = 9.6 Hz), −79.4 (dsept, 2F, $(CF_3)_2CFC\mathbf{F}_2OCH_2F$, $^3J(^{19}F, ^{19}F)$ = 19.1 Hz, $^4J(^{19}F, ^{19}F)$ = 9.4 Hz), −154.9 (t, 1F, $(CF_3)_2CFCF_2OCH_2\mathbf{F}$, $^4J(^{19}F, ^{19}F)$ = 9.7 Hz), −187.9 (m, $(CF_3)_2C\mathbf{F}CF_2OCH_2F$) ppm.

GC-MS [t = 0.49 min] m/z$^+$: 219 [M −OCH_2F], 197 [M −FCF_2H_2], 169 [M −CF_2OCH_2F], 150 [M −CF_3OCH_2F], 131 [M −FCF_3OCHF_2], 100, 99 [M –$(CF_3)_2CF$], 69 [M –$(CF_3)CFCF_2OCH_2F$].

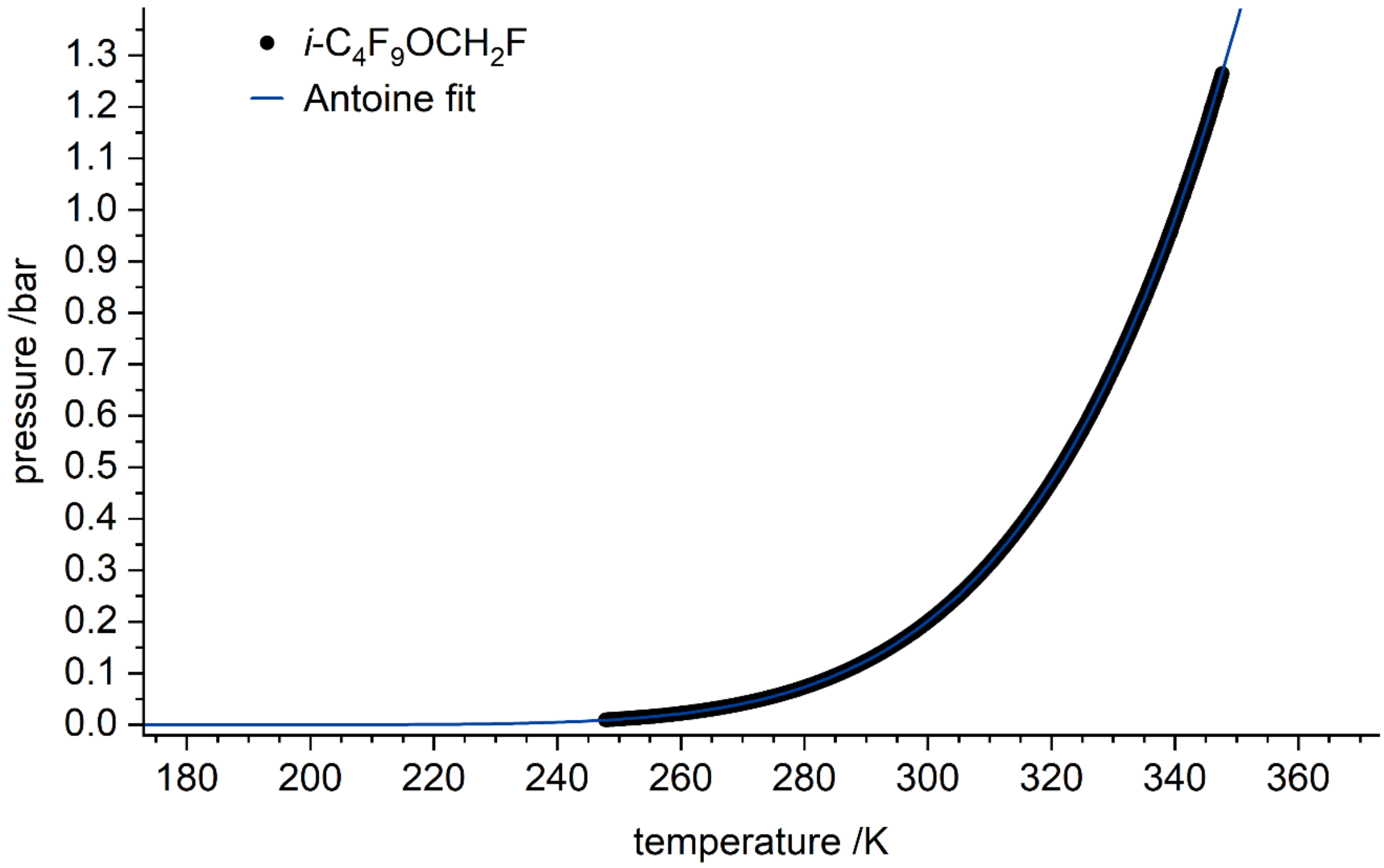

Figure S130. Vapor pressure measurement and Antoine fit of i-$C_4F_9OCH_2F$ (Antoine parameter: A = 4.144; B = 1167.7; C = −58.727for equation: $p = 10^{(A-B/(T+C))}$).

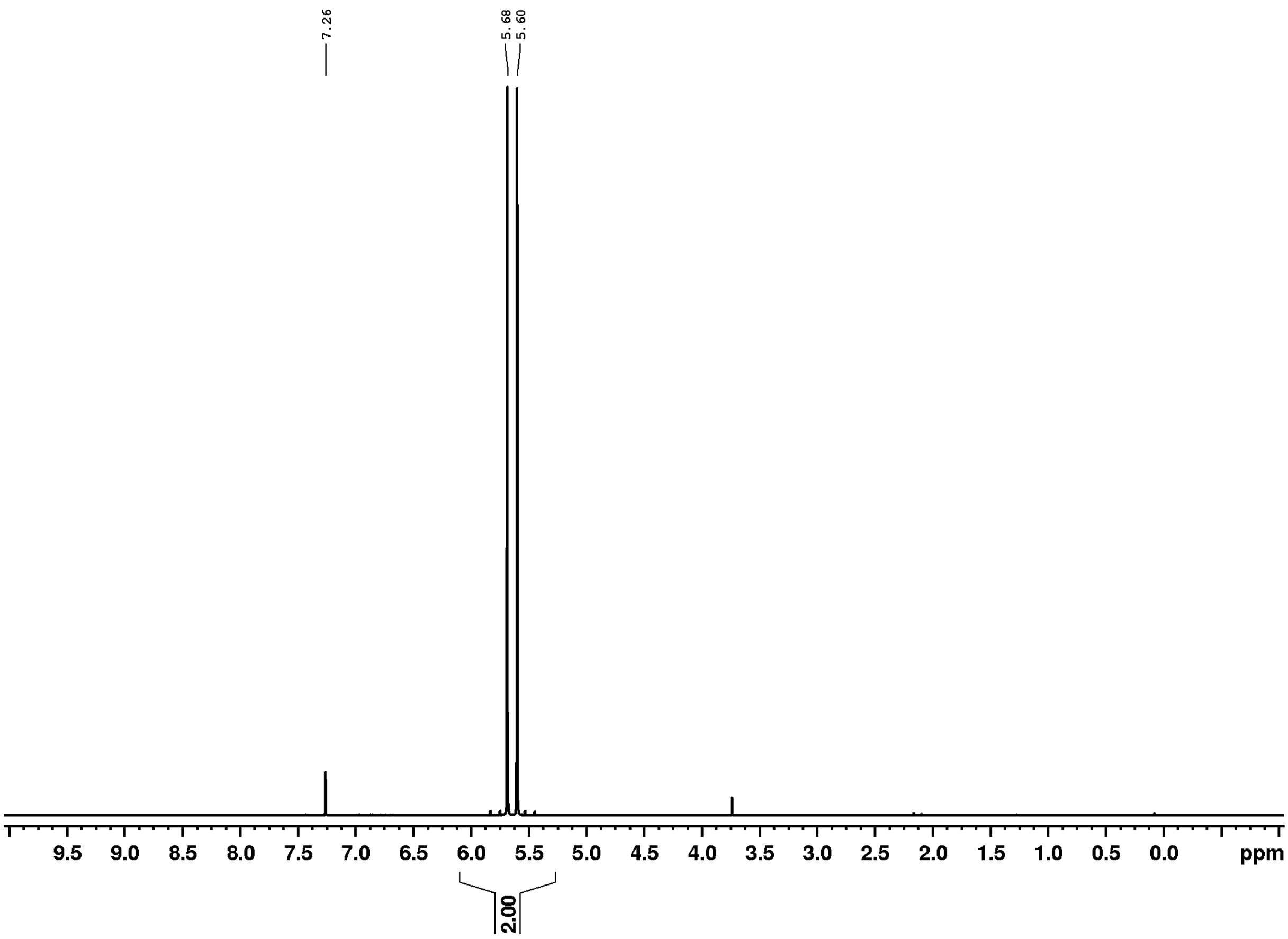

Figure S131. ^{1}H NMR of $(CF_3)_2CFCF_2OCH_2F$ in $CDCl_3$.

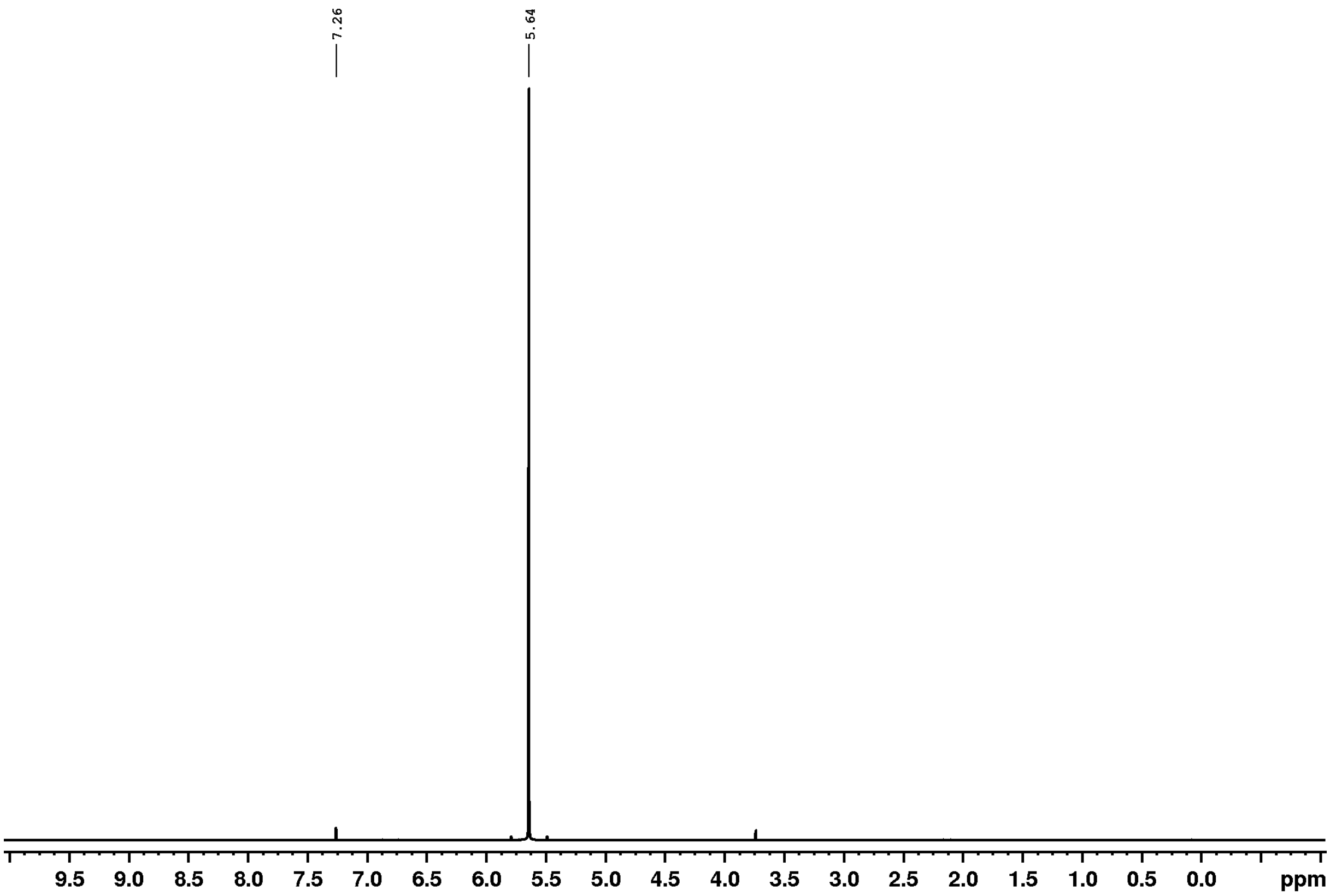

Figure S132. $^{1}H\{^{19}F\}$ NMR of $(CF_3)_2CFCF_2OCH_2F$ in $CDCl_3$.

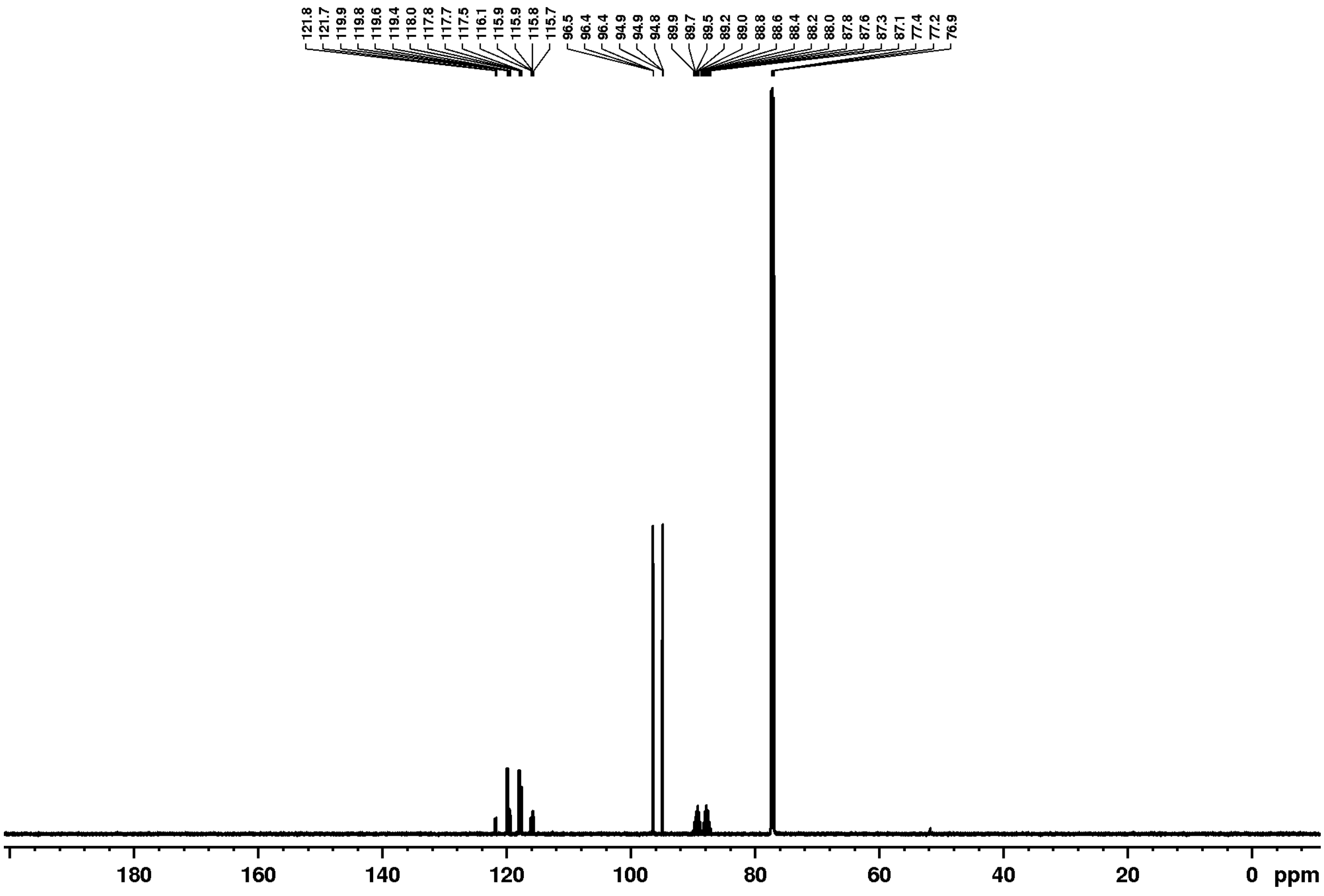

Figure S133. $^{13}C\{^{1}H\}$ NMR of $(CF_3)_2CFCF_2OCH_2F$ in $CDCl_3$.

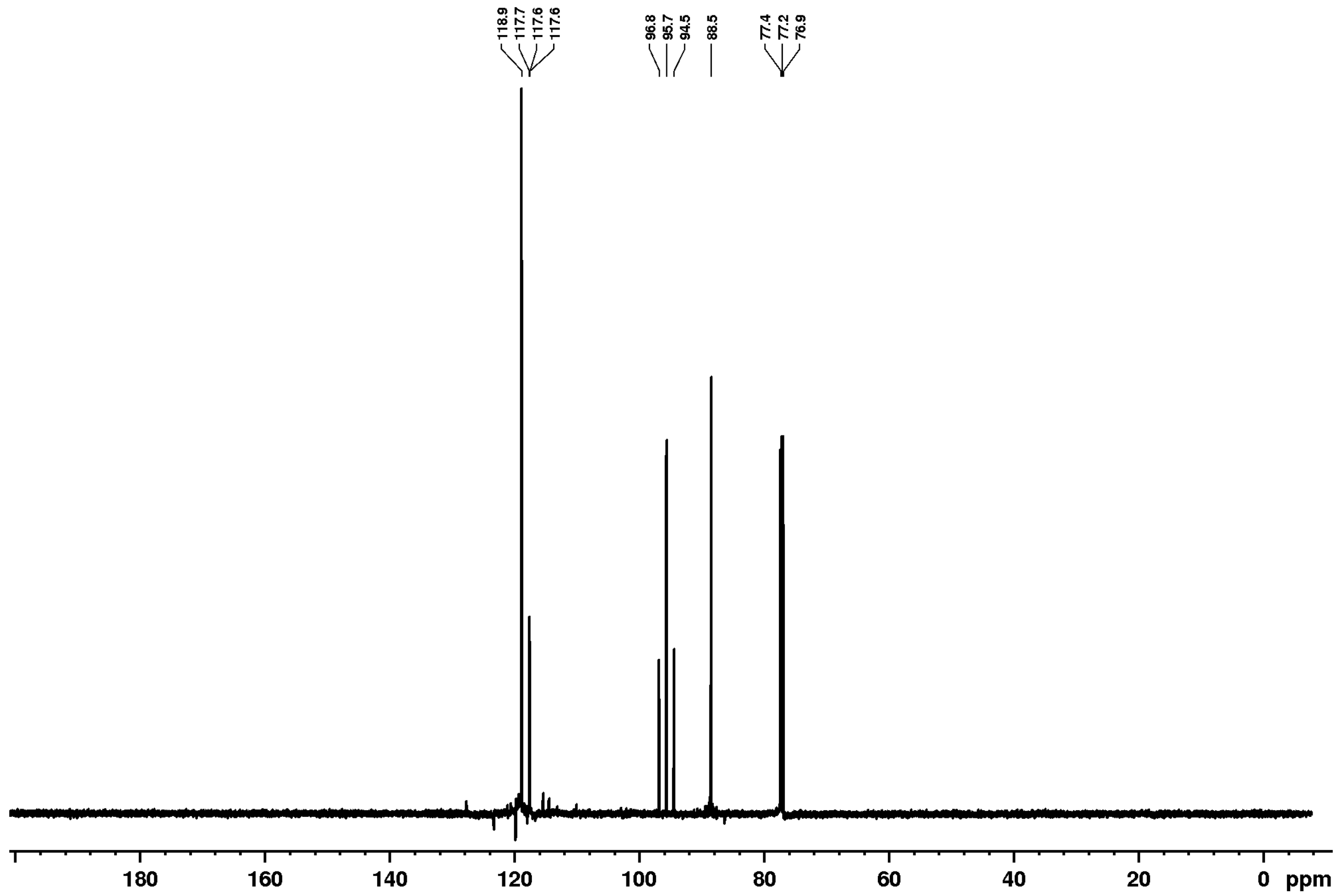

Figure S134. $^{13}C\{^{19}F\}$ NMR of $(CF_3)_2CFCF_2OCH_2F$ in $CDCl_3$.

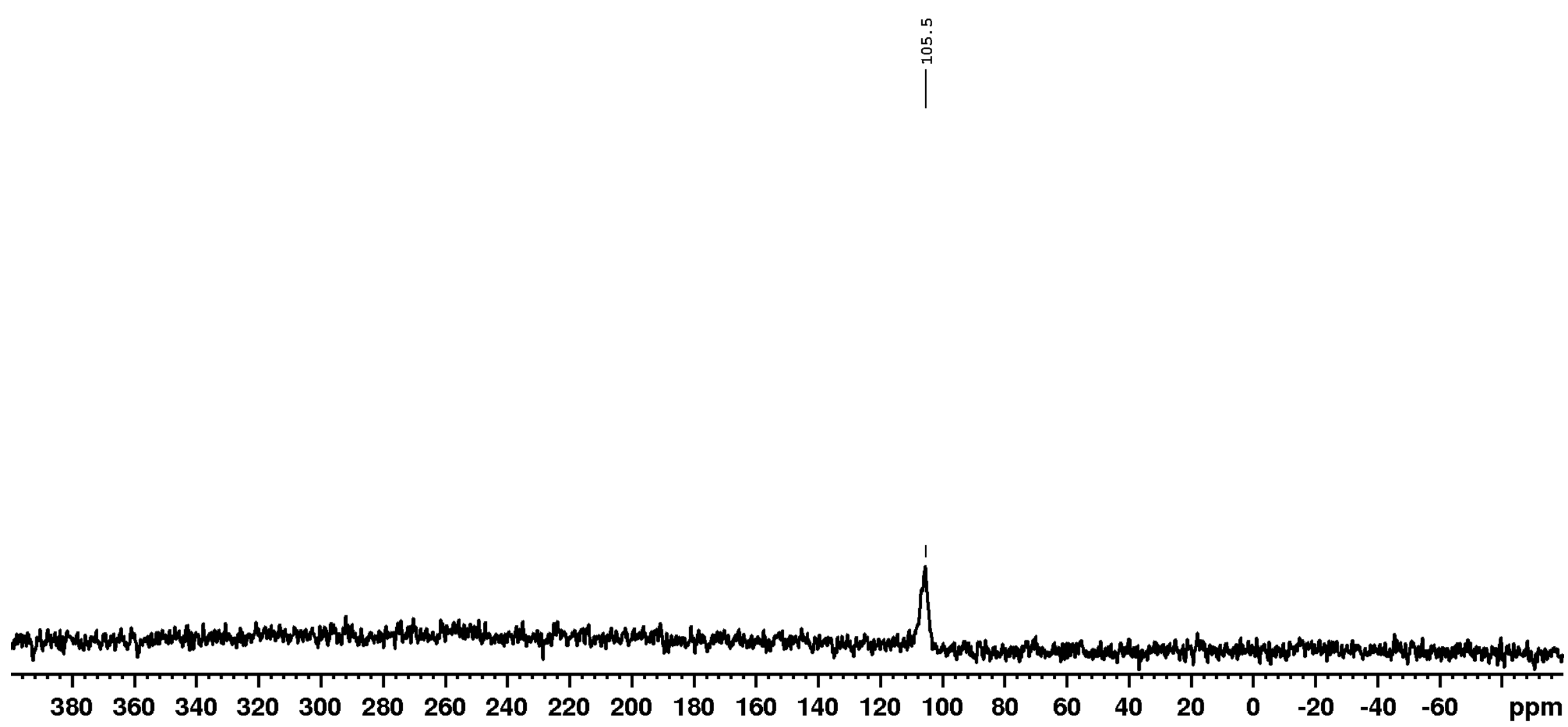

Figure S135. ^{17}O NMR of $(CF_3)_2CFCF_2OCH_2F$ in $CDCl_3$.

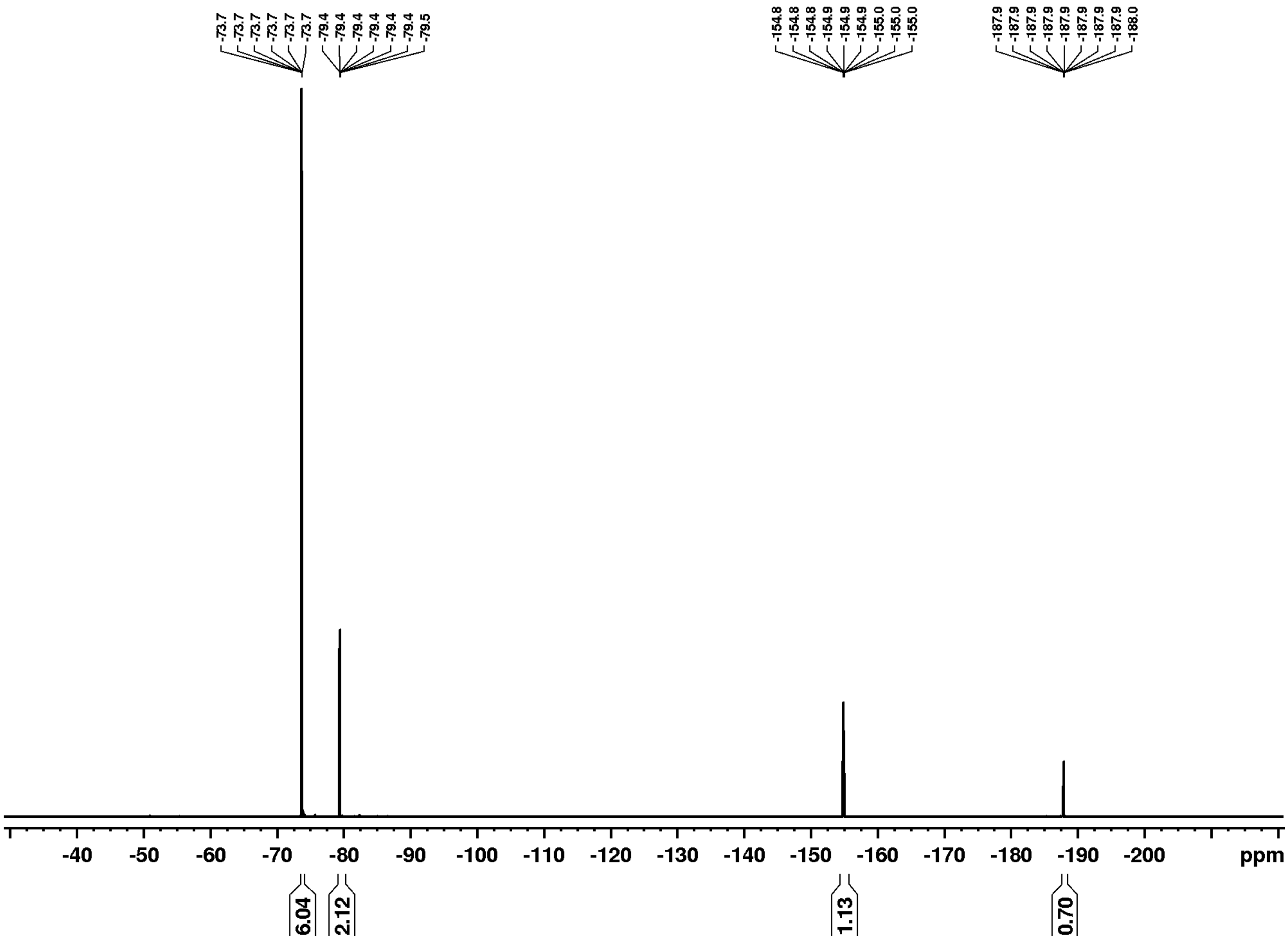

Figure S136. ^{19}F NMR of $(CF_3)_2CFCF_2OCH_2F$ in $CDCl_3$.

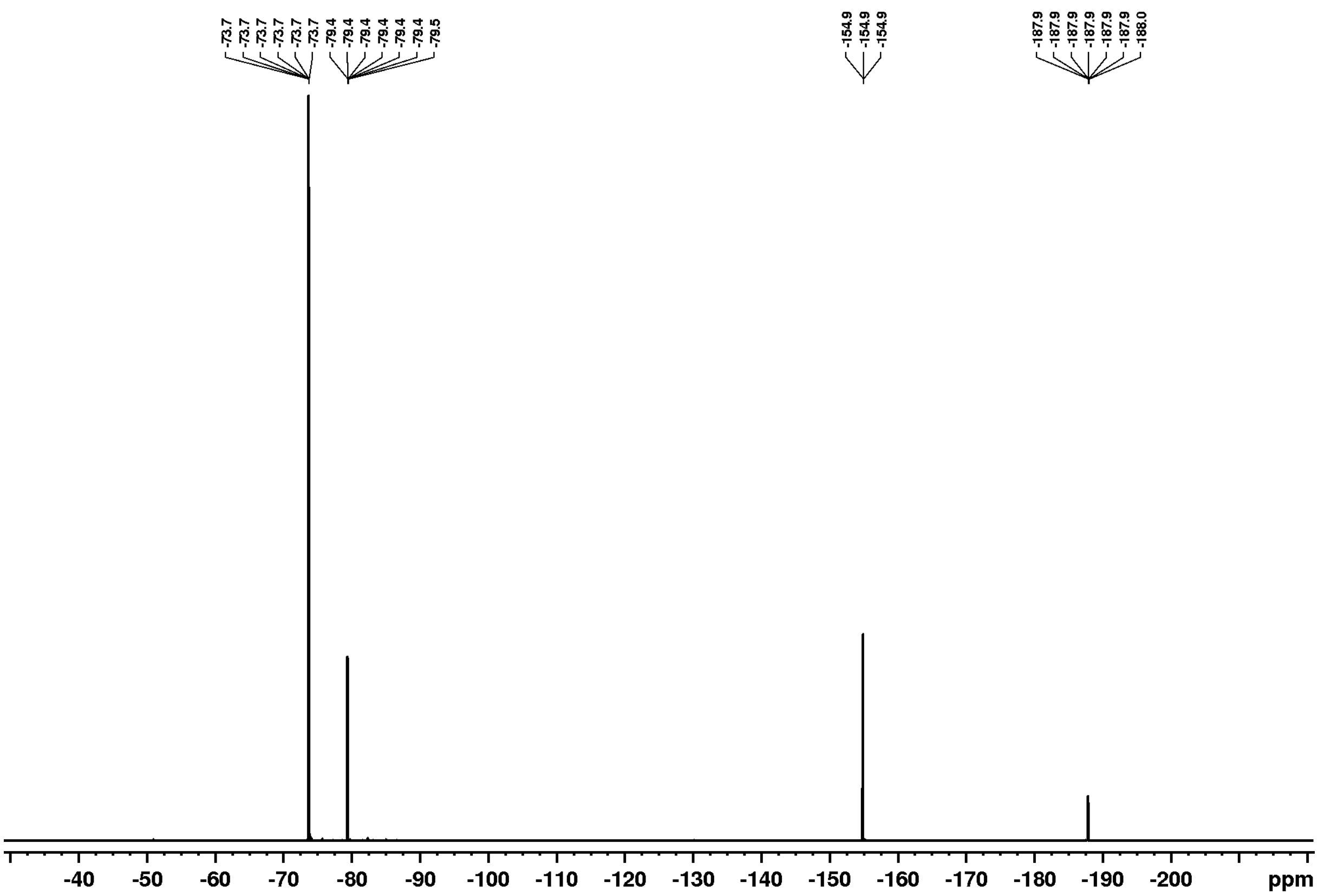

Figure S137. $^{19}F\{^1H\}$ NMR of $(CF_3)_2CFCF_2OCH_2F$ in $CDCl_3$.

***i*-$C_4F_9OCHF_2$**

Boiling point (distillation): T_{bp} =49 °C at 1000 mbar

Boiling point (vapor pressure): T_{bp} = 49.8 °C at 1013 mbar (Antoine parameter: A = 4.053; B = 1080.0; C = −56.093 for equation: $p = 10^{(A-B/(T+C))}$).

Enthalpy of vaporization (T = 298.15 K): $\Delta_{vap}H$ = 31.4 kJ mol^{-1} (for equation: $\Delta_{vap}H = \ln(10)\cdot R\cdot T^2\cdot(B/(T+C)^2)/1000$).

^{1}H NMR (500.1 MHz, $CDCl_3$): δ = 6.74 (t, 1H, (CF_3)CFCF_2OC**H**F_2, $^2J(^{19}F,^1H)$ = 68.8 Hz) ppm.

^{1}H{^{19}F} NMR (400.3 MHz, $CDCl_3$): δ = 6.74 (s, 1H, (CF_3)CFCF_2OC**H**F_2) ppm.

^{13}C NMR (100.7 MHz, $CDCl_3$): δ = 118.6 (qdm, 2C, (**C**F_3)$_2$CFCF_2OCHF_2, $^1J(^{19}F,\ ^{13}C)$ = 287.7 $^3J(^{19}F,\ ^{13}C)$ = 26.2 Hz), 116.5 (tdt, 1C, (CF_3)CF**C**F_2OCHF_2, $^1J(^{19}F,\ ^{13}C)$ = 283.1$^2J(^{19}F,\ ^{13}C)$ = 25.4 Hz, $^3J(^{19}F,\ ^{13}C)$ = 4.3 Hz), 112.3 (tdtd, 1C, (CF_3)CFCF_2O**C**HF_2, $^1J(^{19}F,\ ^{13}C)$ = 266.7$^1J(^{13}C,\ ^1H)$ = 226.4 Hz,$^3J(^{19}F,\ ^{13}C)$ = 5.9 Hz, $^4J(^{19}F,\ ^{13}C)$ = 1.0 Hz), 88.3 (dsept, 1C, (CF_3)**C**FCF_2OCHF_2, $^1J(^{19}F,\ ^{13}C)$ = 222.9$^2J(^{19}F,\ ^{13}C)$ = 32.9 Hz) ppm.

^{13}C{^{1}H} NMR (125.8 MHz, $CDCl_3$): δ = 118.6 (qd, 2C, (**C**F_3)$_2$CFCF_2OCHF_2, $^1J(^{19}F,\ ^{13}C)$ = 289.1 $^3J(^{19}F,\ ^{13}C)$ = 26.1 Hz), 116.5 (tdt, 1C, (CF_3)CF**C**F_2OCHF_2, $^1J(^{19}F,\ ^{13}C)$ = 283.1$^2J(^{19}F,\ ^{13}C)$ = 25.4 Hz, $^3J(^{19}F,\ ^{13}C)$ = 4.3 Hz), 112.3 (ttd, 1C, (CF_3)CFCF_2O**C**HF_2, $^1J(^{19}F,\ ^{13}C)$ = 266.4$^3J(^{19}F,\ ^{13}C)$ = 5.9 Hz, $^4J(^{19}F,\ ^{13}C)$ = 1.0 Hz), 88.3 (dsept, 1C, (CF_3)**C**FCF_2OCHF_2, $^1J(^{19}F,^{13}C)$ = 222.9$^2J(^{19}F,^{13}C)$ = 32.9 Hz) ppm.

^{13}C{^{19}F} NMR (125.8 MHz, $CDCl_3$): δ = 118.6 (s, 2C, (**C**F_3)CFCF_2OCHF_2), 116.5 (d, 1C, (CF_3)CF**C**F_2OCHF_2, $^3J(^{13}C,^1H)$ = 2.1112.3 (d, 1C, (CF_3)CFCF_2O**C**HF_2, $^1J(^{13}C,^1H)$ = 226.288.3 (s, 1C, (CF_3)**C**FCF_2OCHF_2) ppm.

^{17}O NMR (54.3 MHz, $(CH_3)_2CO$): δ = 129.5 (s, 1O, (CF_3)CFCF_2**O**CHF_2) ppm.

^{19}F NMR (470.6 MHz, $CDCl_3$): δ = −73.7 (dt, 6F, (C**F$_3$**)CFCF_2OCHF_2, $^3J(^{19}F,^{19}F)$ = 6.2 Hz, $^4J(^{19}F,^{19}F)$ = 9.6 Hz), −77.3 (m, 2F, (CF_3)CFC**F$_2$**OCHF_2), −85.1 (dt, 2F, (CF_3)CFCF_2OCH**F$_2$**, $^2J(^{19}F,\ ^1H)$ = 68.8 Hz, $^4J(^{19}F,\ ^{19}F)$ = 4.8 Hz), −187.9 (m, 1F, (CF_3)C**F**CF_2OCHF_2) ppm.

^{19}F{^{1}H} NMR (376.6 MHz, $CDCl_3$): δ = −73.7 (dt, 6F, (C**F$_3$**)CFCF_2OCHF_2, $^3J(^{19}F,\ ^{19}F)$ = 6.2 Hz, $^4J(^{19}F,\ ^{19}F)$ = 9.6 Hz), −77.3 (m, 2F, (CF_3)CFC**F$_2$**OCHF_2), −85.1 (t, 2F, (CF_3)CFCF_2OCH**F$_2$**, $^4J(^{19}F,\ ^{19}F)$ = 4.8 Hz), −187.9 (m, 1F, (CF_3)C**F**CF_2OCHF_2) ppm.

GC-MS [t = 0.46 min] m/z$^+$: 219 [M −OCHF_2], [M −CF_3CCF_2OCHF_2], 169 [M −CF_2OCHF_2], 150 [M −CF_3OCHF_2], 131 [M −CF_3FOCHF_2], 100 [M −(CF_3)$_2$CFOCF_2H], 69 [M −CF_3CFCF_2OCHF_2], 51 [M −(CF_3)$_2$CFCF_2O].

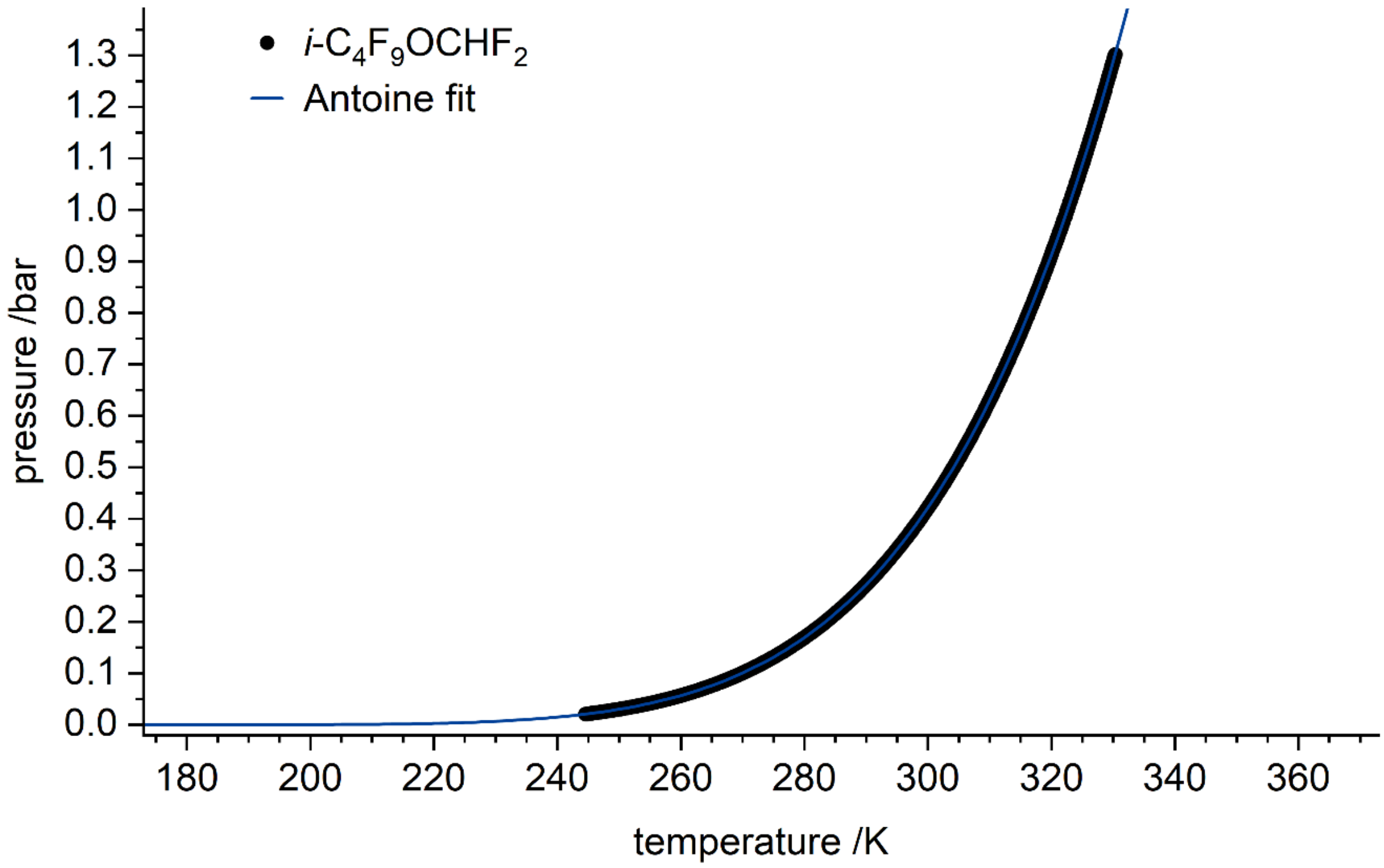

Figure S138. Vapor pressure measurement and Antoine fit of i-$C_4F_9OCHF_2$ (Antoine parameter: A = 4.053; B = 1080.0; C = −56.093 for equation: $p = 10^{(A-B/(T+C))}$).

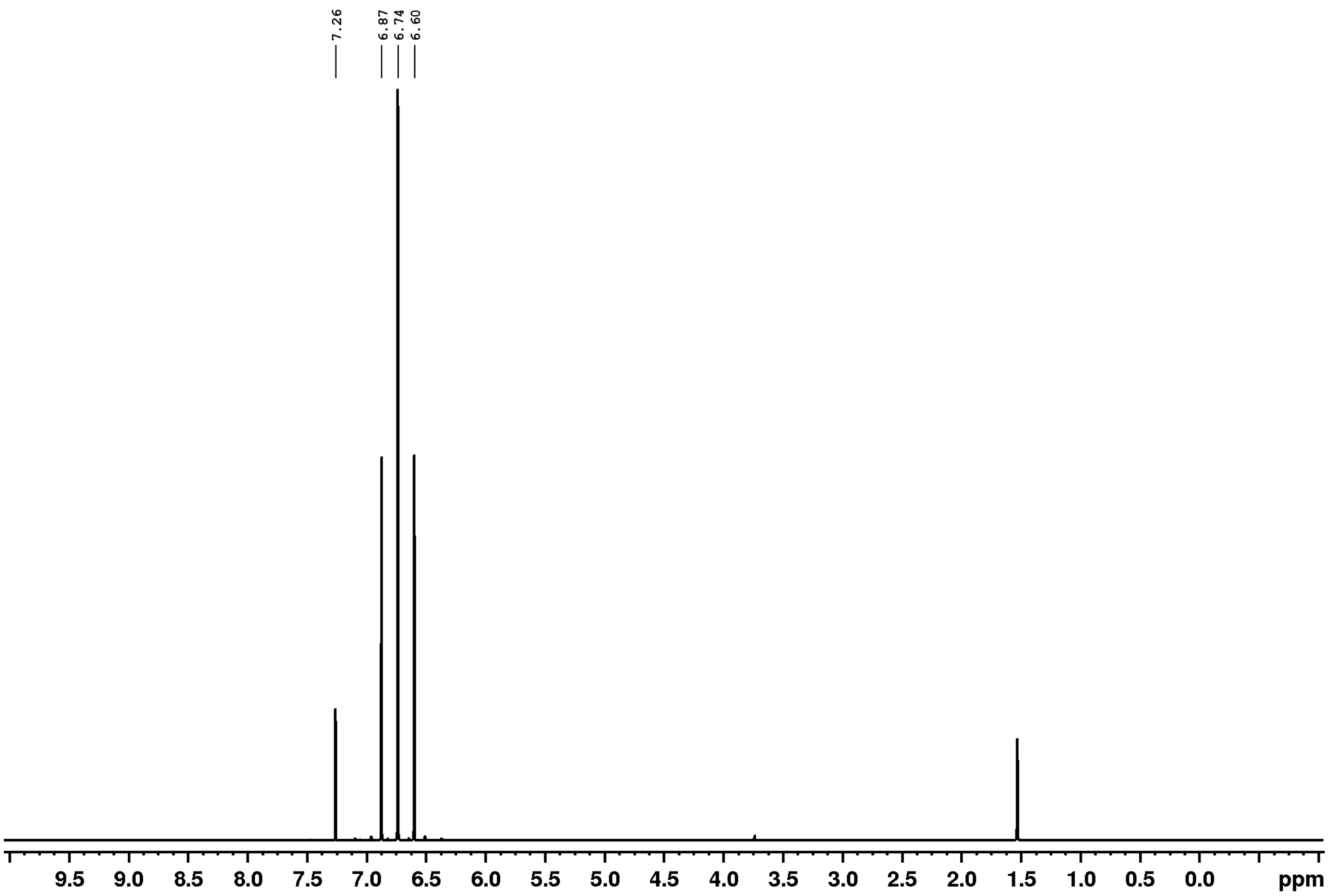

Figure S139. ^{1}H NMR of $(CF_3)_2CFCF_2OCHF_2$ in $CDCl_3$.

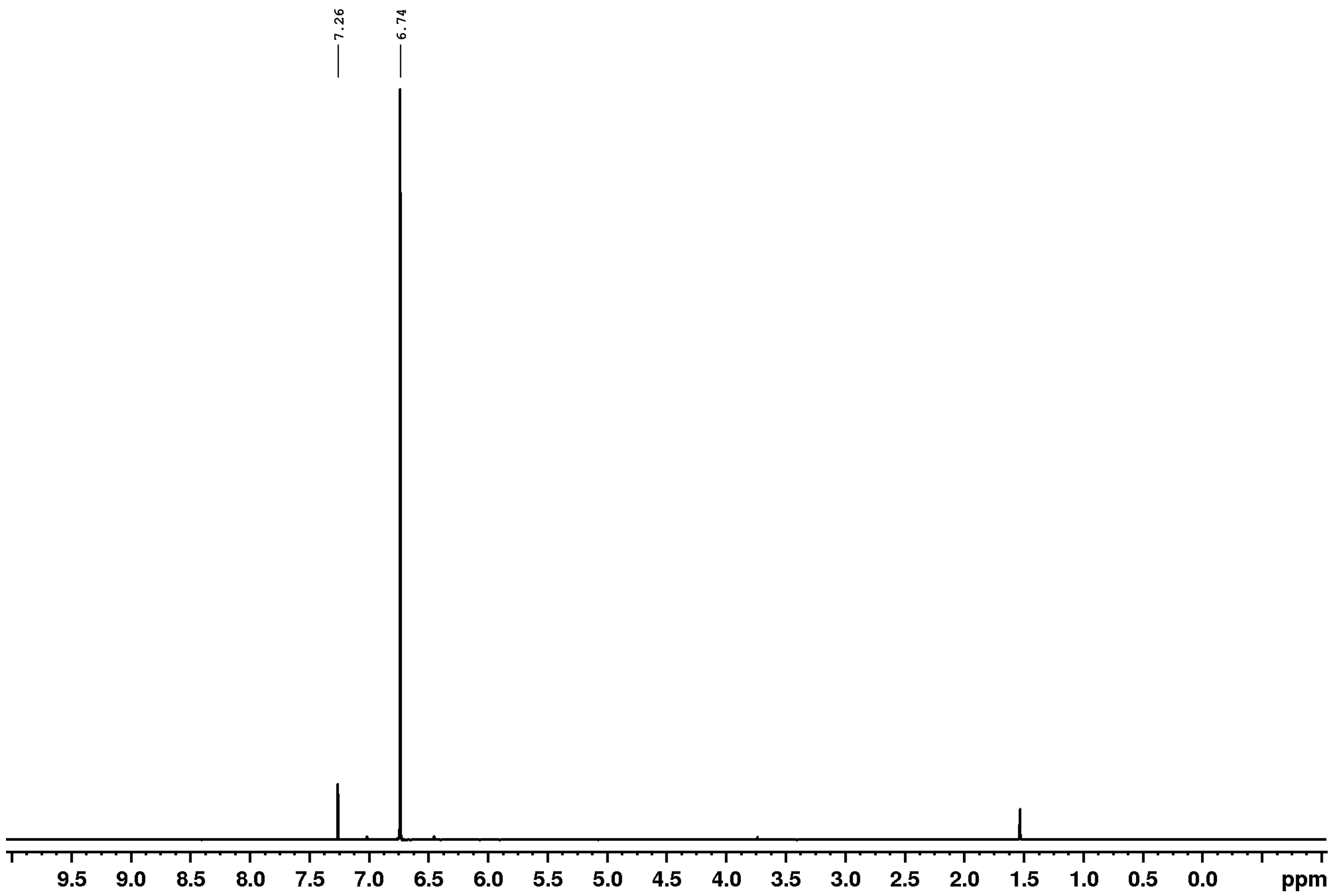

Figure S140. $^{1}H\{^{19}F\}$ NMR of $(CF_3)_2CFCF_2OCHF_2$ in $CDCl_3$.

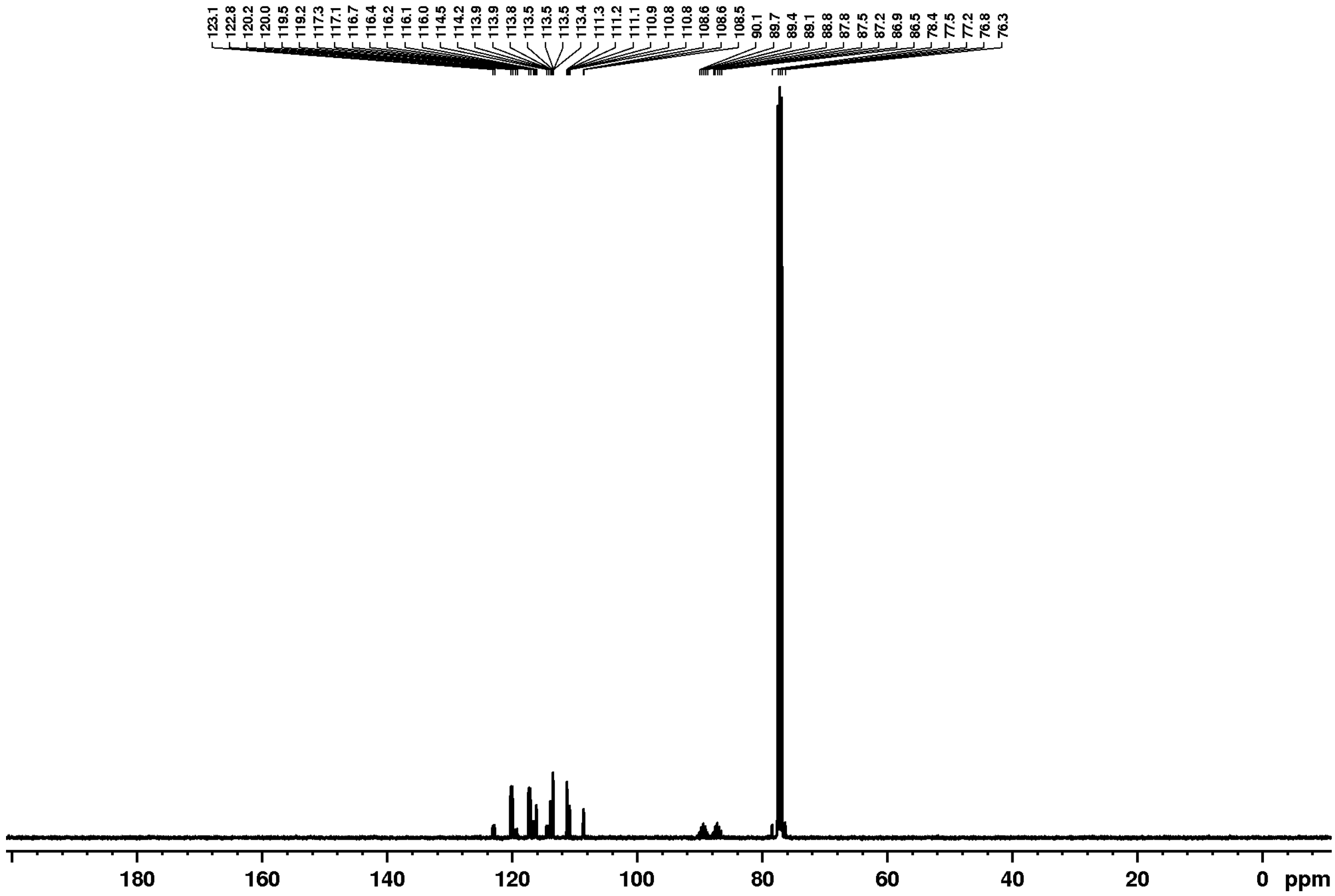

Figure S141. ^{13}C NMR of $(CF_3)_2CFCF_2OCHF_2$ in $CDCl_3$.

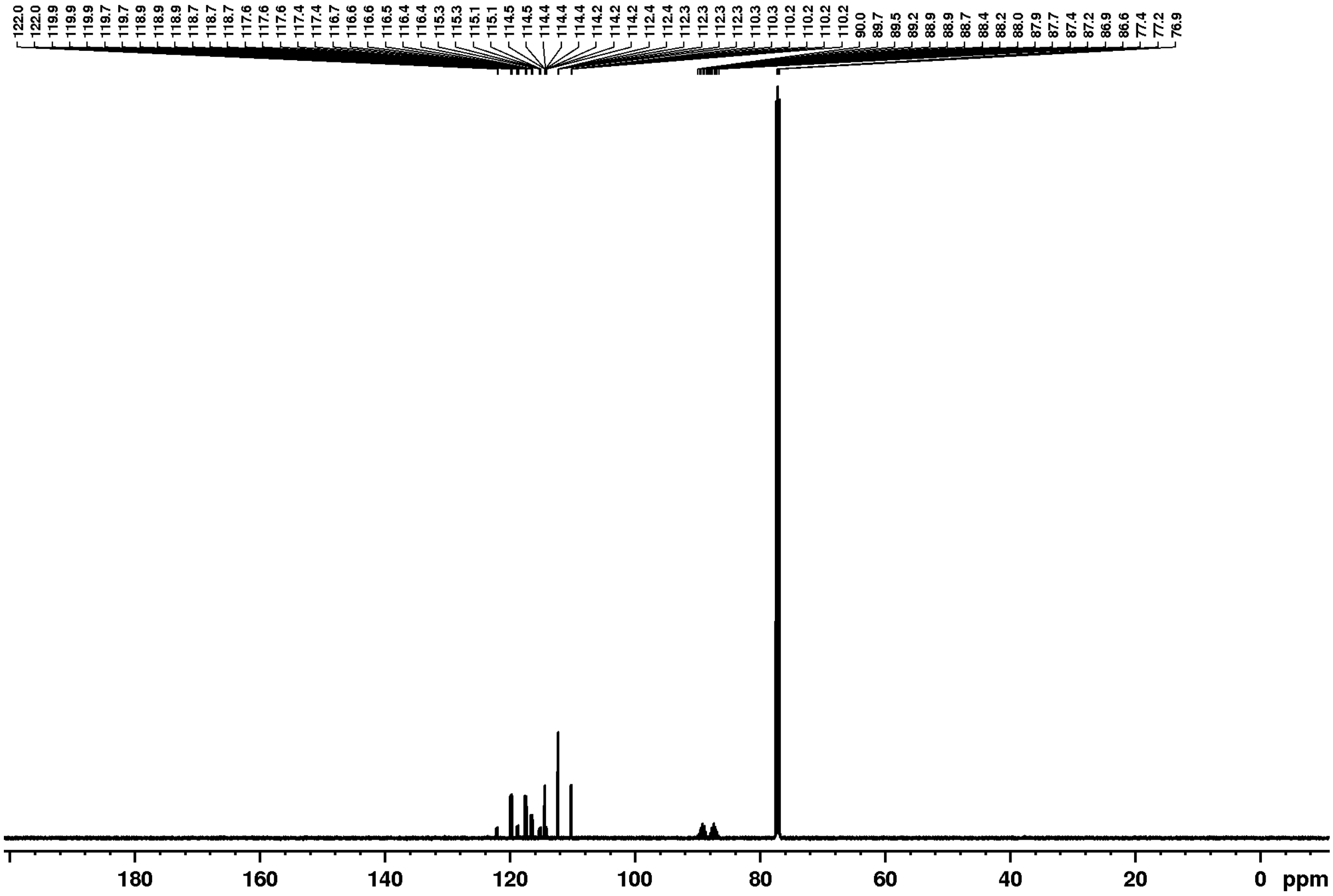

Figure S142. $^{13}C\{^{1}H\}$ NMR of $(CF_3)_2CFCF_2OCHF_2$ in $CDCl_3$.

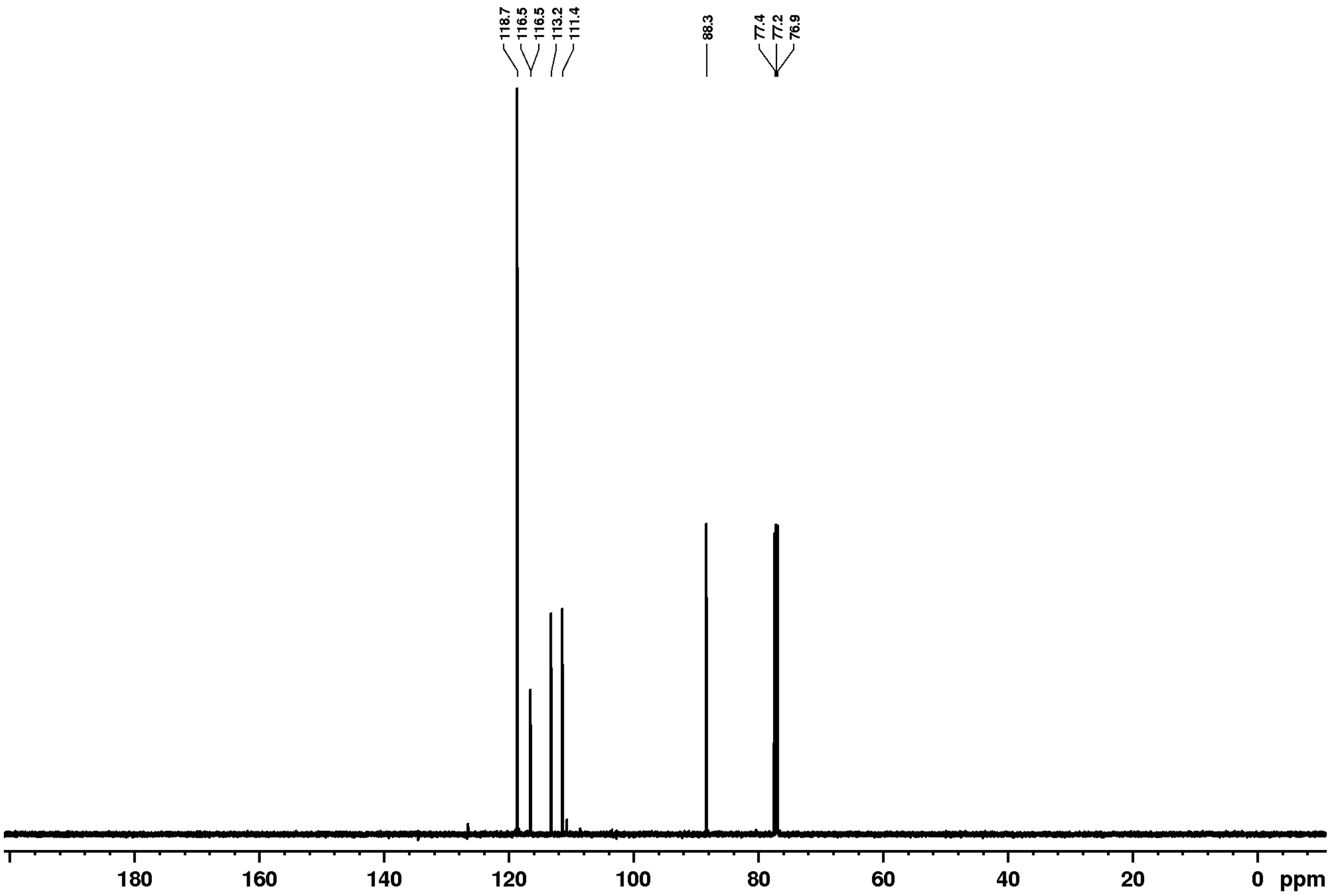

Figure S143. $^{13}C\{^{19}F\}$ NMR of $(CF_3)_2CFCF_2OCHF_2$ in $CDCl_3$.

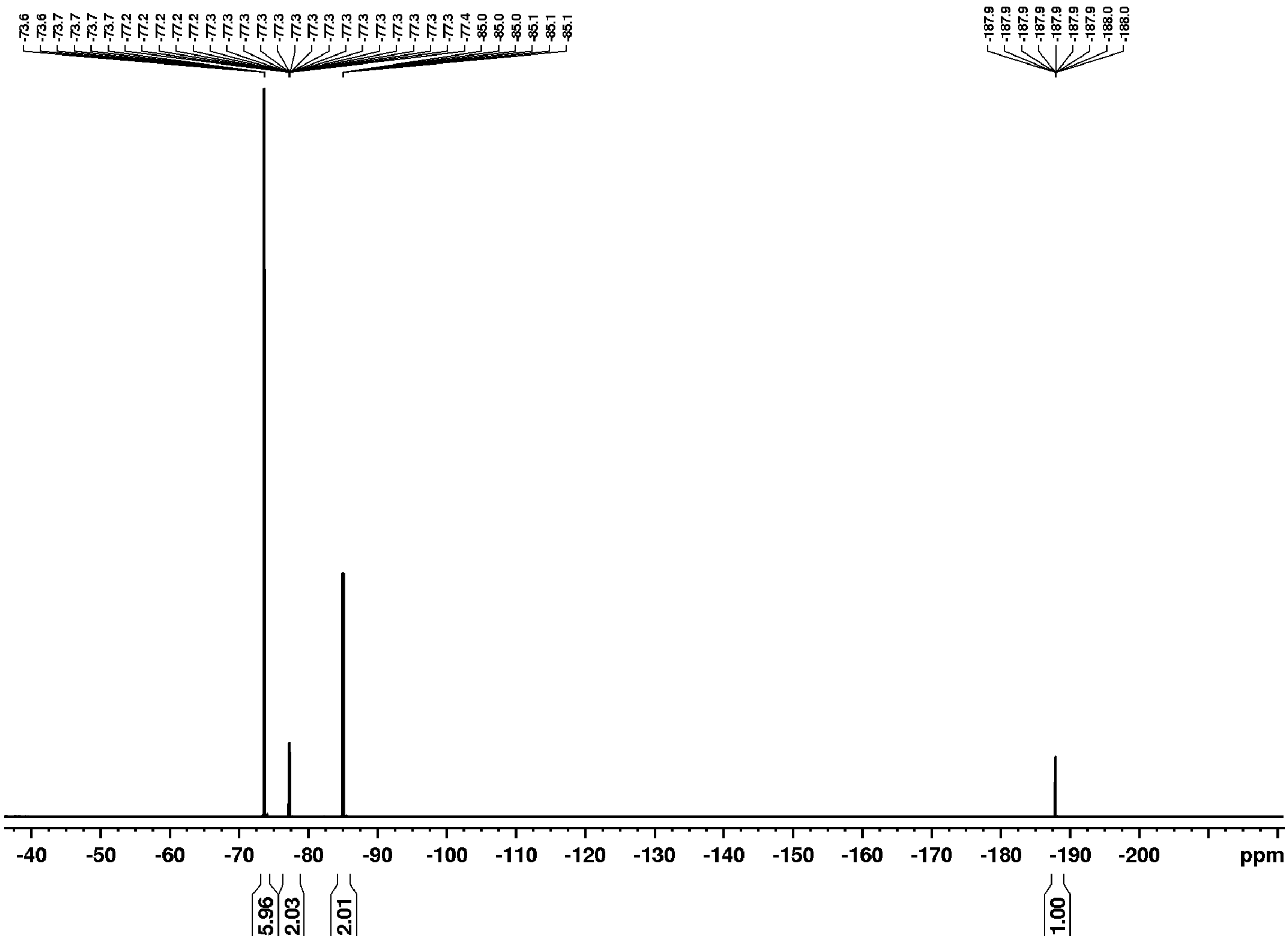

Figure S144. ^{19}F NMR of $(CF_3)_2CFCF_2OCHF_2$ in $CDCl_3$.

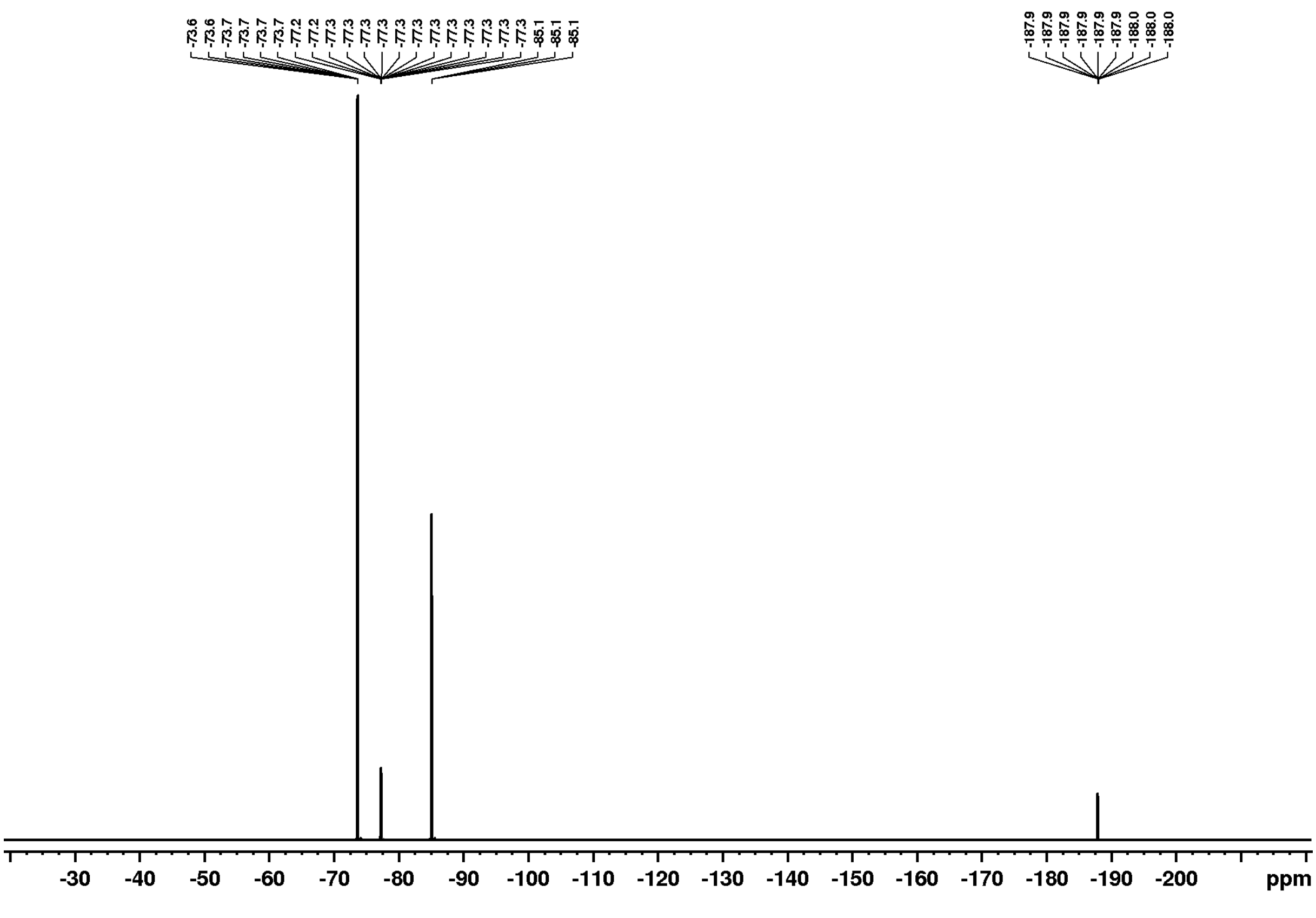

Figure S145. $^{19}F\{^1H\}$ NMR of $(CF_3)_2CFCF_2OCHF_2$ in $CDCl_3$.

***i*-$C_4F_9OCF_3$**

Boiling point (distillation): T_{bp} = 34 °C at 990 mbar.

Boiling point (vapor pressure): T_{bp} = 34.7 °C at 1013 mbar (Antoine parameter: A = 4.195; B = 1114.4; C = −41.861 for equation: $p = 10^{(A-B/(T+C))}$).

Enthalpy of vaporization (T = 298.15 K): $\Delta_{vap}H$ = 28.8 kJ mol^{-1} (for equation: $\Delta_{vap}H = \ln(10)\cdot R\cdot T^2\cdot(B/(T+C)^2)/1000$).

^{13}C NMR (100.7 MHz, $CDCl_3$): δ = 119.2 (q, 1C, $(CF_3)_2CFCF_2O$**C**F_3, $^1J(^{19}F, ^{13}C)$ = 267.8 (**C**$F_3)_2CFCF_2OCF_3$, $^1J(^{19}F, ^{13}C)$ = 287.4 $^2J(^{19}F, ^{13}C)$ = 26.2 Hz), 115.9 (td, 1C, $(CF_3)_2CF$**C**F_2OCF_3, $^1J(^{19}F, ^{13}C)$ = 287.1 Hz, $^2J(^{19}F, ^{13}C)$ = 25.1 Hz), 88.4 (dsept, 1C, $(CF_3)_2$**C**FCF_2OCF_3, $^1J(^{19}F, ^{13}C)$ = 223.9 Hz, $^2J(^{19}F, ^{13}C)$ = 33.1 Hz) ppm.

^{13}C{^{1}H} NMR (150.9 MHz, $CDCl_3$): δ = 119.1 (q, 1C, $(CF_3)_2CFCF_2O$**C**F_3, $^1J(^{19}F, ^{13}C)$ = 269.9 (**C**$F_3)_2CFCF_2OCF_3$, $^1J(^{19}F, ^{13}C)$ = 289.4 $^2J(^{19}F, ^{13}C)$ = 26.2 Hz), 115.9 (td, 1C, $(CF_3)_2CF$**C**F_2OCF_3, $^1J(^{19}F, ^{13}C)$ = 289.2 Hz, $^2J(^{19}F, ^{13}C)$ = 25.8 Hz), 88.4 (dsept, 1C, $(CF_3)_2$**C**FCF_2OCF_3, $^1J(^{19}F, ^{13}C)$ = 224.5 Hz, $^2J(^{19}F, ^{13}C)$ = 32.8 Hz) ppm.

^{13}C{^{19}F} NMR (150.9 MHz, $CDCl_3$): δ = 119.1 (s, 1C, $(CF_3)_2CFCF_2O$**C**F_3(**C**$F_3)_2CFCF_2OCF_3$), 115.9 (s, 1C, $(CF_3)_2CF$**C**F_2OCF_3), 88.4 (s, 1C, $(CF_3)_2$**C**FCF_2OCF_3) ppm.

^{17}O NMR (81.4 MHz, $CDCl_3$): δ = 129.2 (s, 1O, $(CF_3)_2CFCF_2$**O**CF_3) ppm.

^{19}F NMR (564.7 MHz, $CDCl_3$): δ = −55.2 (t, 3F, $(CF_3)_2CFCF_2OC$**F$_3$**, $^4J(^{19}F, ^{19}F)$ = 9.1 Hz), −73.5 (dt, 6F, (C**F$_3$**)$CFCF_2OCF_3$, $^3J(^{19}F, ^{19}F)$ = 6.2 Hz, $^4J(^{19}F, ^{19}F)$ = 9.6 Hz), −81.7 (m, 2F, $(CF_3)_2CFC$**F$_2$**OCF_3), −190.3 (m, 1F, $(CF_3)_2C$**F**CF_2OCF_3) ppm.

GC-MS [t = 0.45 min] m/z$^+$: 219 [M −OCF_3], 197 [M −CF_5], 169 [M −CF_2OCF_3], 150 [M −FCF_2OCF_3], 135 [M −$(CF_3)_2CF$], 131 [M −CF_3FOCF_3], 100 [M −$CF_3CF_2OCF_3$], 69 [M −$(CF_3)_2CF_2O$], 50 [M −$(CF_3)_2CFOCF_3$].

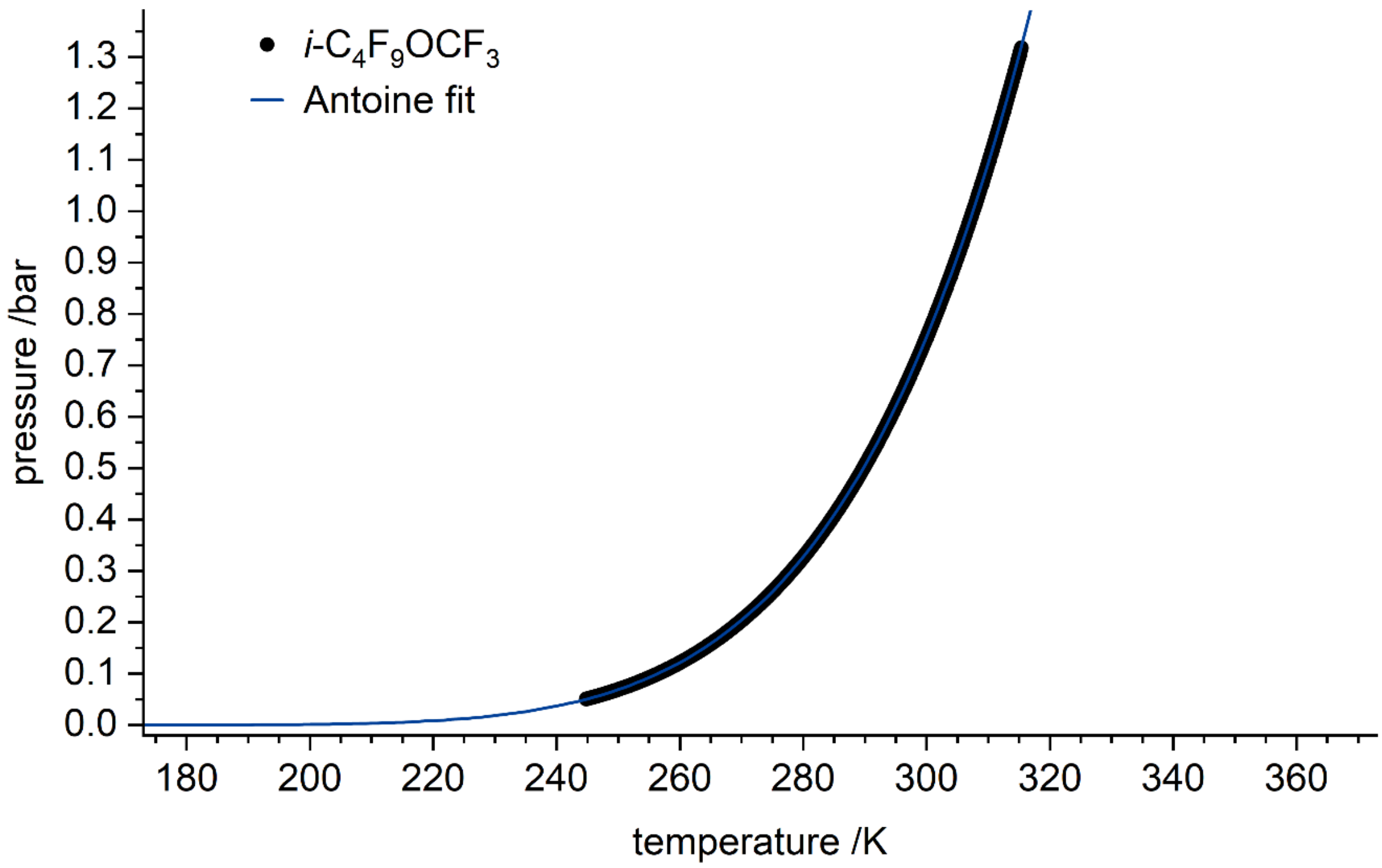

Figure S146. Vapor pressure measurement and Antoine fit of *i*-$C_4F_9OCF_3$ (Antoine parameter: A = 4.195; B = 1114.4; C = −41.861 for equation: $p = 10^{(A-B/(T+C))}$).

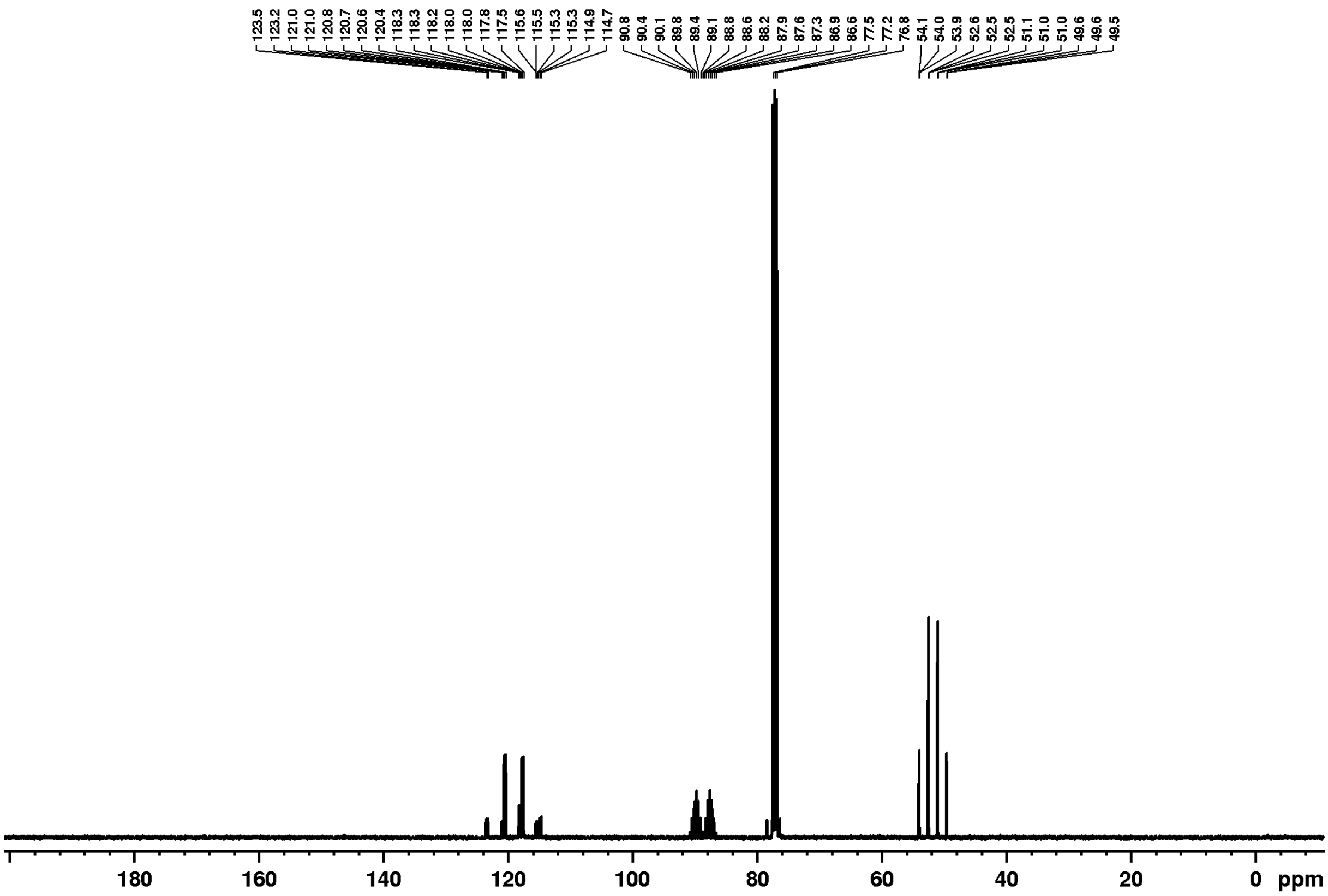

Figure S147. ^{13}C NMR of $(CF_3)_2CFCF_2OCF_3$ in $CDCl_3$.

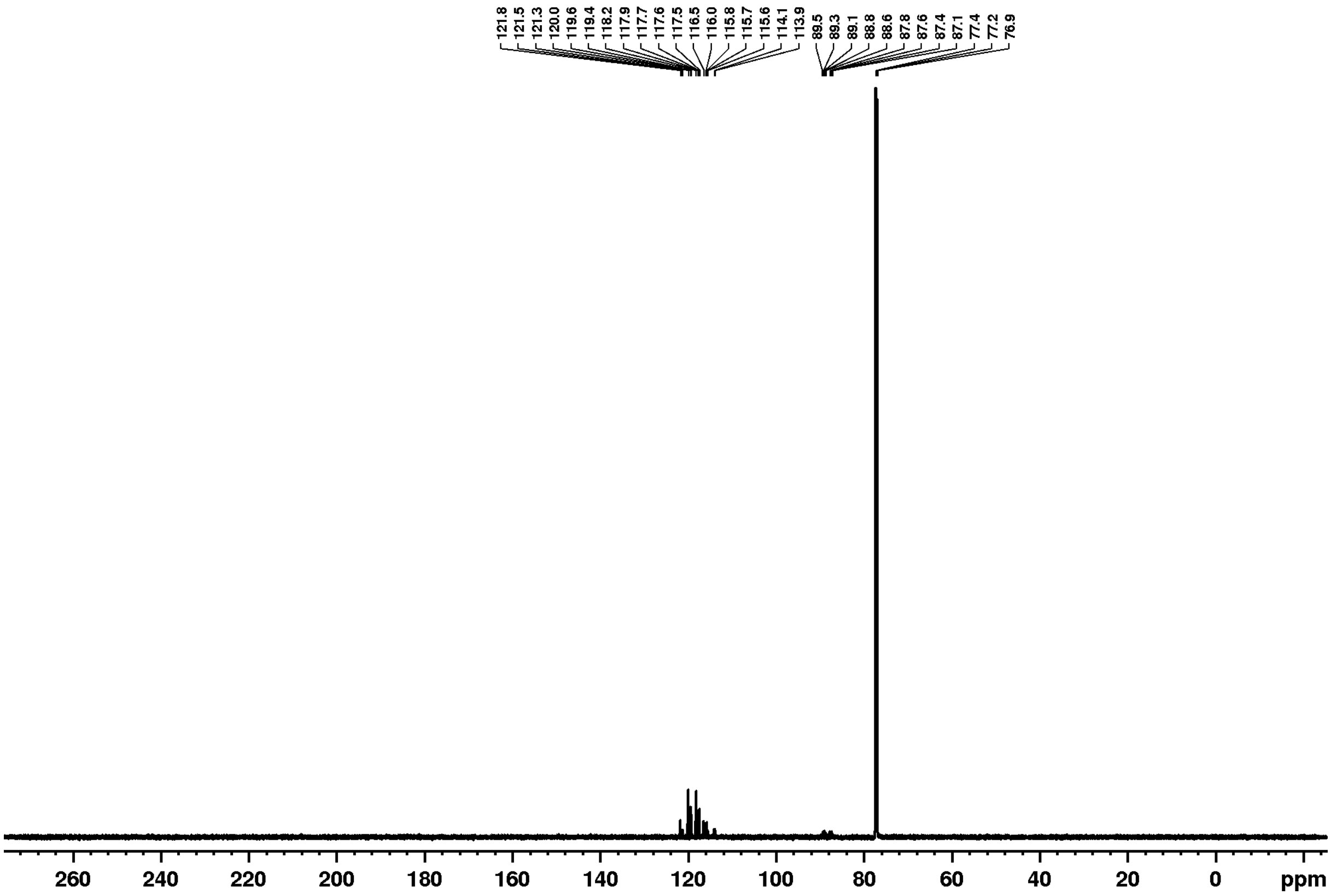

Figure S148. $^{13}C\{^1H\}$ NMR of $(CF_3)_2CFCF_2OCF_3$ in $CDCl_3$.

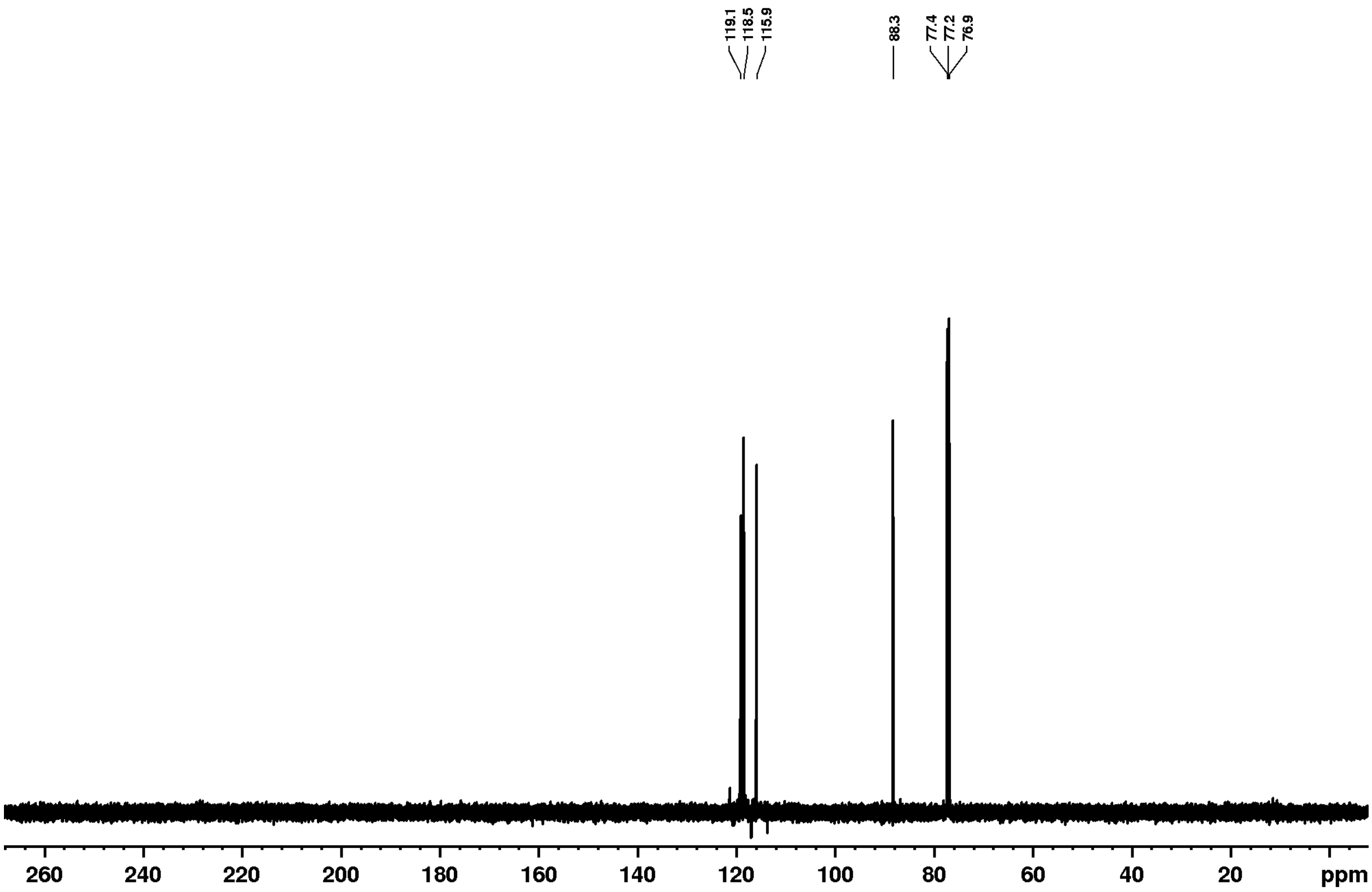

Figure S149. $^{13}C\{^{19}F\}$ NMR of $(CF_3)_2CFCF_2OCF_3$ in $CDCl_3$.

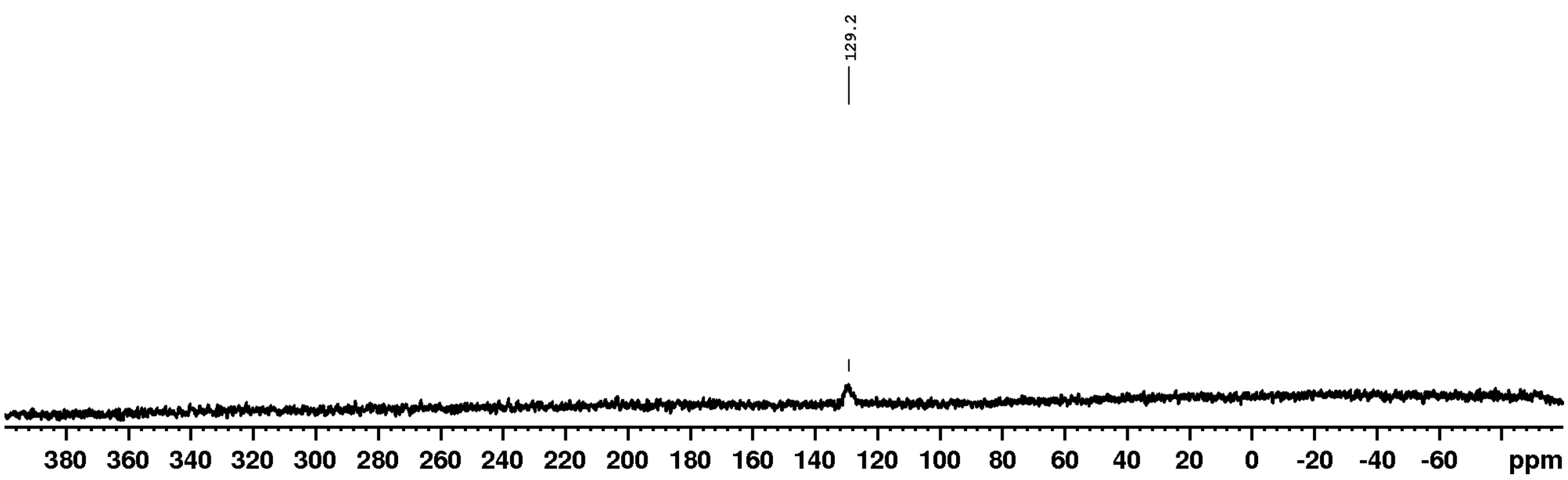

Figure S150. ^{17}O NMR of $(CF_3)_2CFCF_2OCF_3$ in $CDCl_3$.

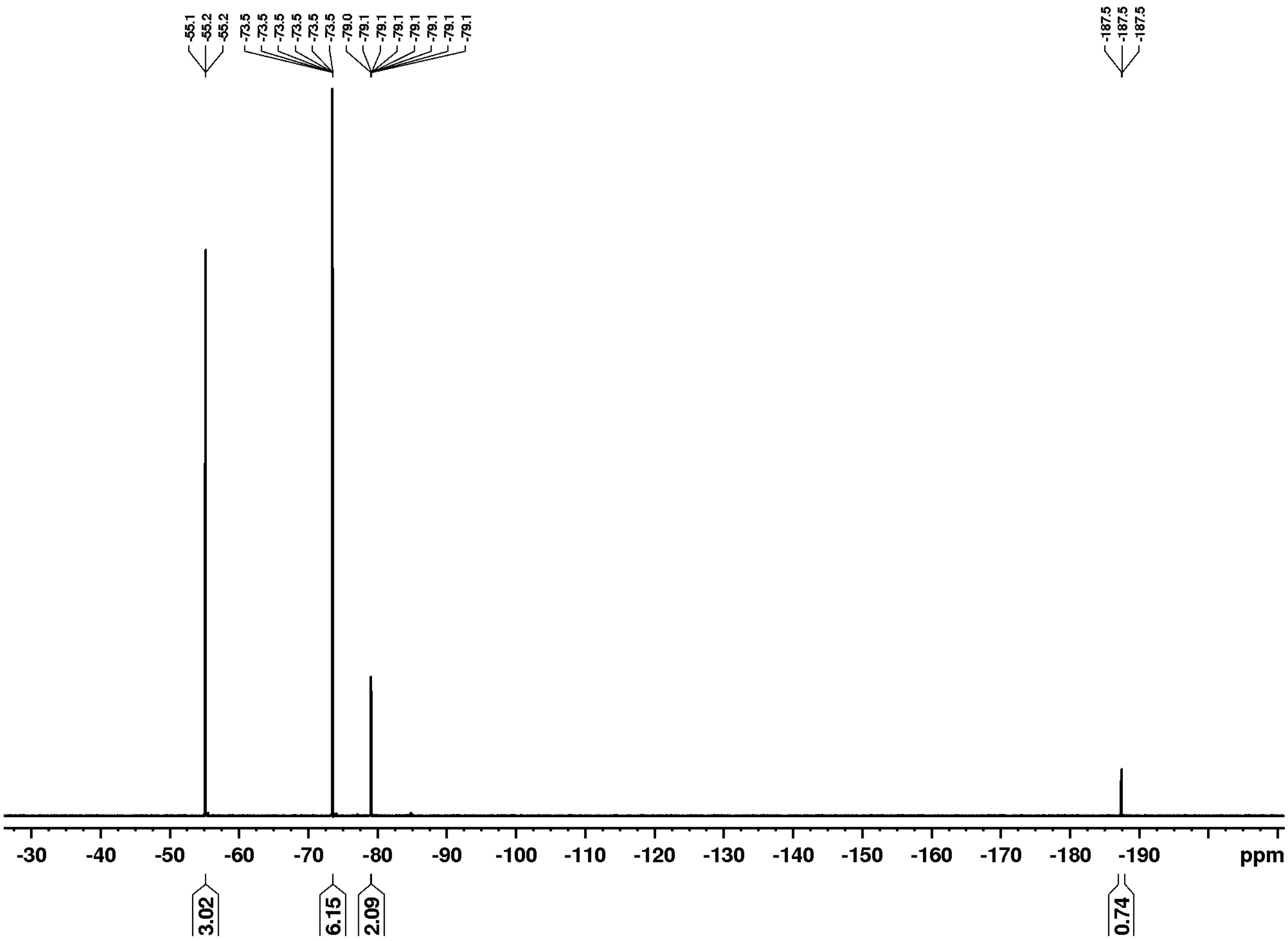

Figure S151. ^{19}F NMR of $(CF_3)_2CFCF_2OCF_3$ in $CDCl_3$.

3.6. Electrochemical fluorination of $(CF_3)_2CFCF_2COCH_3$/$CF_3CF_2CF_2CF_2COCH_3$ (Novec7100)

340 mL stainless steel cell with nickel electrodes (effective anode area S = 3.4 dm^2), the construction of which is described elsewhere [15], was filled with anhydrous hydrogen fluoride (260 mL). A constant voltage of 5.0 V was applied for 20 hours. Novec7100 (*i*+*n*-$C_4F_9OCH_3$) (332.7 g, 1:331 mol) was added at regular intervals to the pre-electrolyzed anhydrous HF. The gaseous products from the cell were collected in a PTFE trap maintained at −78 °C. The cell was operated at 5.7 – 6.5 V and the current was held between 0.3 and 1.7 A. The cell temperature was maintained at 3 °C and the temperature of the condenser was kept between −30 and −21 °C. After consumption of 221 Ah the reaction was stopped. In total, 290.6 g of crude organic was collected from the cell. From the cold traps additional 21.1 g of crude organic was received. The aHF from the cell was stored at −78°C. After one day the aHF phase was carefully poured onto ice (300 g) and a small organic layer (25 g) formed. All three batches of crude organic were combined and washed with an aqueous solution of $NaHCO_3$ (2%) until the pH was neutral and then dried over $MgSO_4$.

Separation of the mixture of *i*+*n*-$C_4F_9OCF_3$ (78%), *i*+*n*-$C_4F_9OCHF_2$ (20%) and *i*+*n*-$C_4F_9OCH_3$ (2%) was carried out via distillation $(CF_3)_3COCH_2F$ was not isolated.

***i*+*n*-$C_4F_9OCH_3$** (Novec 7100, 80% $(CF_3)_2CFCF_2OCH_3$, 20% $CF_3CF_2CF_2CF_2OCH_3$ according to ^{19}F NMR)

Boiling point (vapor pressure): T_{bp} = 59.8 °C at 1013 mbar (Antoine parameter: A = 4.049; B = 1119.6; C = −56.071 for equation: $p = 10^{(A-B/(T+C))}$).

Enthalpy of vaporization (T = 298.15 K): $\Delta_{vap}H$ = 32.5 kJ mol^{-1} (for equation: $\Delta_{vap}H = \ln(10)\cdot R\cdot T^2\cdot(B/(T+C)^2)/1000$).

^1H NMR (600.2 MHz, $CDCl_3$): δ = 3.74 (m, 3H, $(CF_3)_2CFCF_2OC\mathbf{H}_3$, $CF_3CF_2CF_2CF_2OC\mathbf{H}_3$) ppm.

$^1H\{^{19}F\}$ NMR (600.2 MHz, $CDCl_3$): δ = 3.74 (s, 3H, $(CF_3)_2CFCF_2OC\mathbf{H}_3$), 3.73 (s, 3H, $CF_3CF_2CF_2CF_2OC\mathbf{H}_3$) ppm.

$^{13}C\{^1H\}$ NMR (125.8 MHz, $CDCl_3$): δ = 118.8 (qdm, 2C, $(\mathbf{C}F_3)_2CFCF_2OCH_3$, $^1J(^{19}F, ^{13}C)$ = 288.1 Hz, $^2J(^{19}F, ^{13}C)$ = 26.4 Hz), 117.9 (td, 1C, $(CF_3)_2CF\mathbf{C}F_2OCH_3$, $^1J(^{19}F, ^{13}C)$ = 273.9 Hz, $^2J(^{19}F, ^{13}C)$ = 24.2 Hz), 117.4 (qtm, $\mathbf{C}F_3CF_2CF_2CF_2OCH_3$, $^1J(^{19}F, ^{13}C)$ = 287.9 Hz, $^2J(^{19}F, ^{13}C)$ = 33.4 Hz), 116.9 (ttm, 1C, $CF_3CF_2CF_2\mathbf{C}F_2OCH_3$, $^1J(^{19}F, ^{13}C)$ = 273.0 $^2J(^{19}F, ^{13}C)$ = 29.8 Hz), 108.3 (tm, 1C, $CF_3CF_2\mathbf{C}F_2CF_2OCH_3$, $^1J(^{19}F, ^{13}C)$ = 268.3 Hz), 108.2 (tm, 1C, $CF_3\mathbf{C}F_2CF_2CF_2OCH_3$, $^1J(^{19}F, ^{13}C)$ = 268.5 Hz), 88.4 (dsept, 1C, $(CF_3)_2\mathbf{C}FCF_2OCH_3$, $^1J(^{19}F, ^{13}C)$ = 220.0 Hz, $^2J(^{19}F, ^{13}C)$ = 33.2 Hz), 51.5 (td, 1C, $(CF_3)_2CFCF_2O\mathbf{C}H_3$, $^3J(^{19}F, ^{13}C)$ = 6.8 Hz, $^4J(^{19}F, ^{13}C)$ = 0.5 Hz), 51.4 (t, 1C, $CF_3CF_2CF_2CF_2O\mathbf{C}H_3$, $^3J(^{19}F, ^{13}C)$ = 6.6 Hz) ppm. **$^{13}C\{^{19}F\}$ NMR** (125.8 MHz, $CDCl_3$): δ = 119.0 (s, 2C, $(\mathbf{C}F_3)_2CFCF_2OCH_3$), 118.1 (q, 1C, $(CF_3)_2CF\mathbf{C}F_2OCH_3$, $^3J(^{13}C, ^1H)$ = 3.9 Hz), 117.7 (s, 1C, $\mathbf{C}F_3CF_2CF_2CF_2OCH_3$), 117.2 (q, 1C, $CF_3CF_2CF_2\mathbf{C}F_2OCH_3$, $^3J(^{13}C, ^1H)$ = 3.8 Hz), 108.9 (m, 1C, $CF_3CF_2\mathbf{C}F_2CF_2OCH_3$), 108.4 (m, 1C, $CF_3\mathbf{C}F_2CF_2CF_2OCH_3$), 88.7 (s, 1C, $(CF_3)_2\mathbf{C}FCF_2OCH_3$), 51.8 (q, 1C, $(CF_3)_2CFCF_2O\mathbf{C}H_3$, $^1J(^{13}C, ^1H)$ = 148.9 Hz), 51.7 (q, 1C, $CF_3CF_2CF_2CF_2O\mathbf{C}H_3$, $^1J(^{13}C, ^1H)$ = 148.8 Hz) ppm.

^{17}O NMR (81.4 MHz, $CDCl_3$): δ = 44.8 (s, 1O, $(CF_3)_2CFCF_2\mathbf{O}CH_3$, $CF_3CF_2CF_2CF_2\mathbf{O}CH_3$) ppm.

^{19}F NMR (564.7 MHz, $CDCl_3$): δ = −75.4 (dt, 6F, $(C\mathbf{F}_3)_2CFCF_2OCH_3$, $^3J(^{19}F, ^{19}F)$ = 6.1 Hz, $^4J(^{19}F, ^{19}F)$ = 9.6 Hz), −81.5 (tt, 3F, $C\mathbf{F}_3CF_2CF_2CF_2OCH_3$, $^3J(^{19}F, ^{19}F)$ = 9.3 Hz, $^4J(^{19}F, ^{19}F)$ = 2.4), −82.4 (dsept, 2F, $(CF_3)_2CFC\mathbf{F}_2OCH_3$, $^3J(^{19}F, ^{19}F)$ = 9.6 Hz, $^4J(^{19}F, ^{19}F)$ = 9.5 −88.8 (m, 2F, $(CF_3CF_2CF_2C\mathbf{F}_2OCH_3)$, −126.6 (m, 2F,

$CF_3CF_2CF_2CF_2OCH_3$), −127.0 (m, 2F, $CF_3CF_2CF_2CF_2OCH_3$), −189.0 (septt, 1F, $(CF_3)_2CFCF_2OCH_3$, $^3J(^{19}F, ^{19}F)$ = 7.2 Hz, $^3J(^{19}F, ^{19}F)$ = 5.7 Hz) ppm.

$^{19}F\{^1H\}$ NMR (564.7 MHz, $CDCl_3$): δ = −75.4 (dt, 6F, $(CF_3)_2CFCF_2OCH_3$, $^3J(^{19}F, ^{19}F)$ = 6.1 Hz, $^4J(^{19}F, ^{19}F)$ = 9.6 Hz), −81.5 (tt, 3F, $CF_3CF_2CF_2CF_2OCH_3$, $^3J(^{19}F, ^{19}F)$ = 9.3 Hz, $^4J(^{19}F, ^{19}F)$ = 2.4), −82.4 (dsept, 2F, $(CF_3)_2CFCF_2OCH_3$, $^3J(^{19}F, ^{19}F)$ = 9.6 Hz, $^4J(^{19}F, ^{19}F)$ = 9.5 −88.8 (m, 2F, ($CF_3CF_2CF_2CF_2OCH_3$), −126.6 (m, 2F, $CF_3CF_2CF_2CF_2OCH_3$), −127.0 (m, 2F, $CF_3CF_2CF_2CF_2OCH_3$), −189.0 (septt, 1F, $(CF_3)_2CFCF_2OCH_3$, $^3J(^{19}F, ^{19}F)$ = 7.2 Hz, $^3J(^{19}F, ^{19}F)$ = 5.7 Hz) ppm.**GC-MS** [t = 0.47 min] m/z$^+$: 249 [M]$^+$, 231 [M −F], 219 [M −OCF_3], 181 [M −CF_3], 169 [M −CF_2OCH_3], 150 [M −FCF_2OCH_3], 131 [M −C_2F_5], 119 [M −$C_2F_4OCF_3$], 100 [M −$CF_3CF_2OCH_3$], 81 [M −C_3F_7], 69 [M −$C_3F_6OCH_3$].

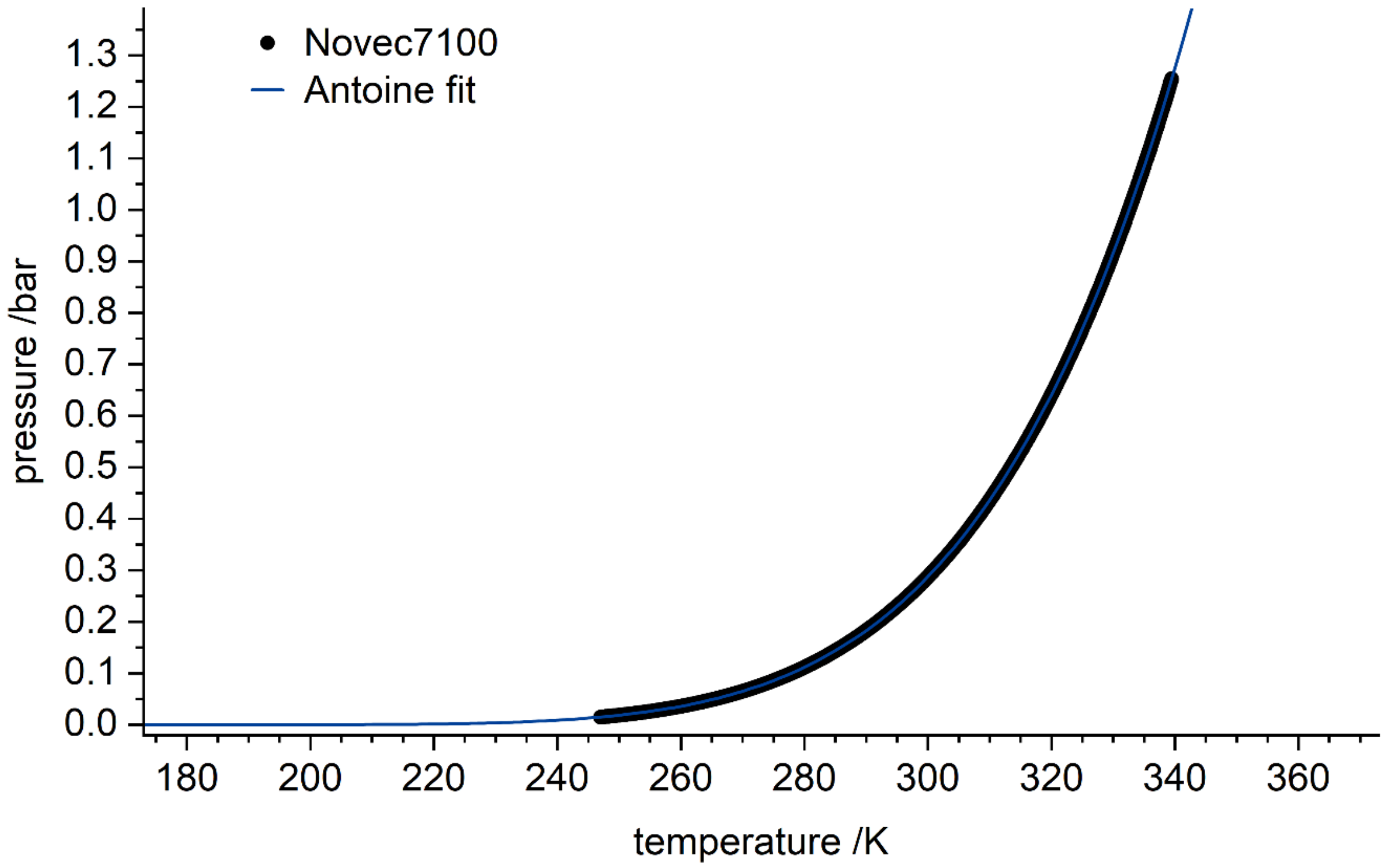

Figure S152. Vapor pressure measurement and Antoine fit of $(CF_3)_2CFCF_2OCH_3/CF_3CF_2CF_2CF_2OCH_3$ (Antoine parameter: A = 4.049; B = 1119.6; C = −56.071 for equation: $p = 10^{(A-B/(T+C))}$).

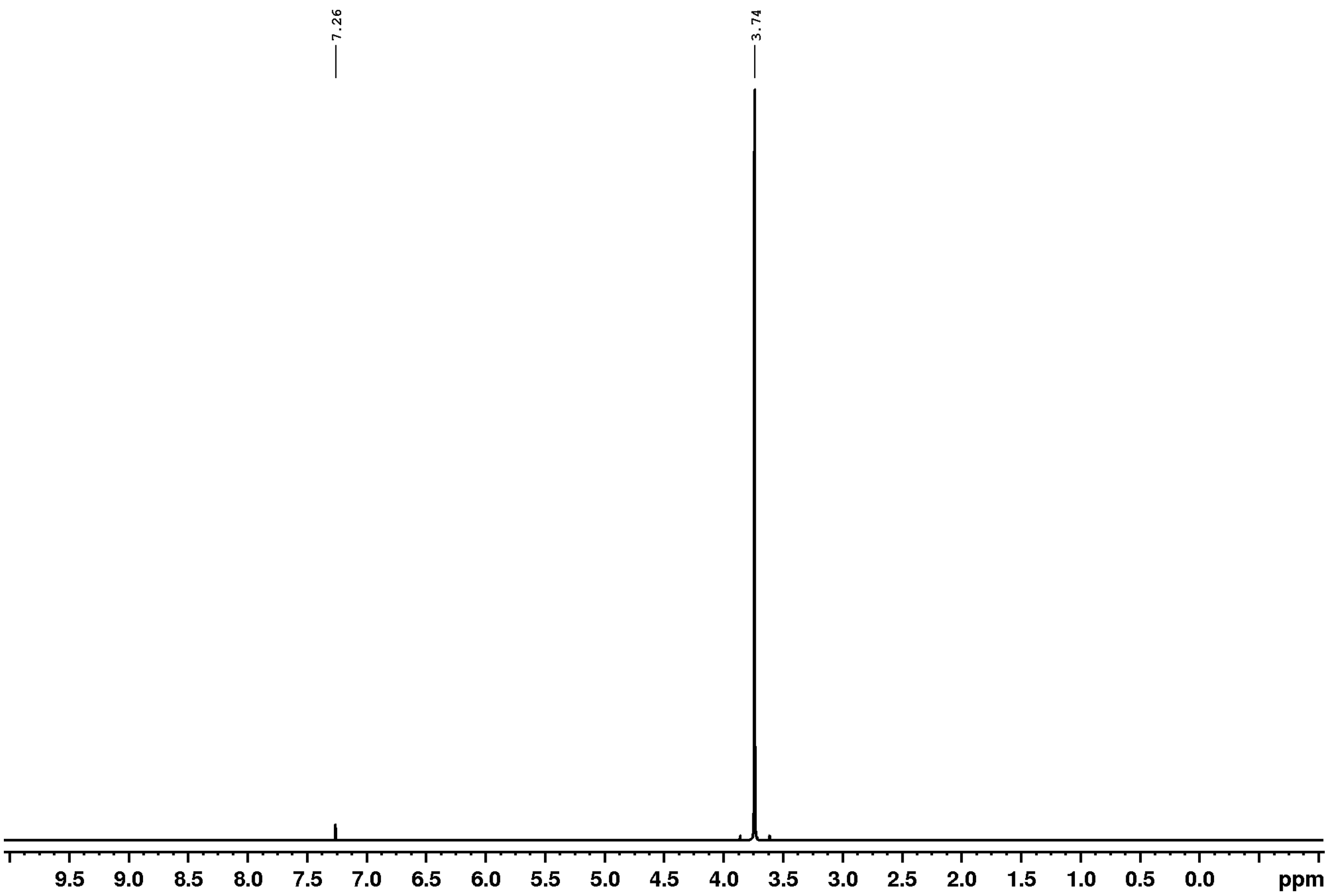

Figure S153. ^1H NMR of $(CF_3)_2CFCF_2OCH_3/CF_3CF_2CF_2CF_2OCH_3$ in $CDCl_3$, integrals not shown as signals overlap.

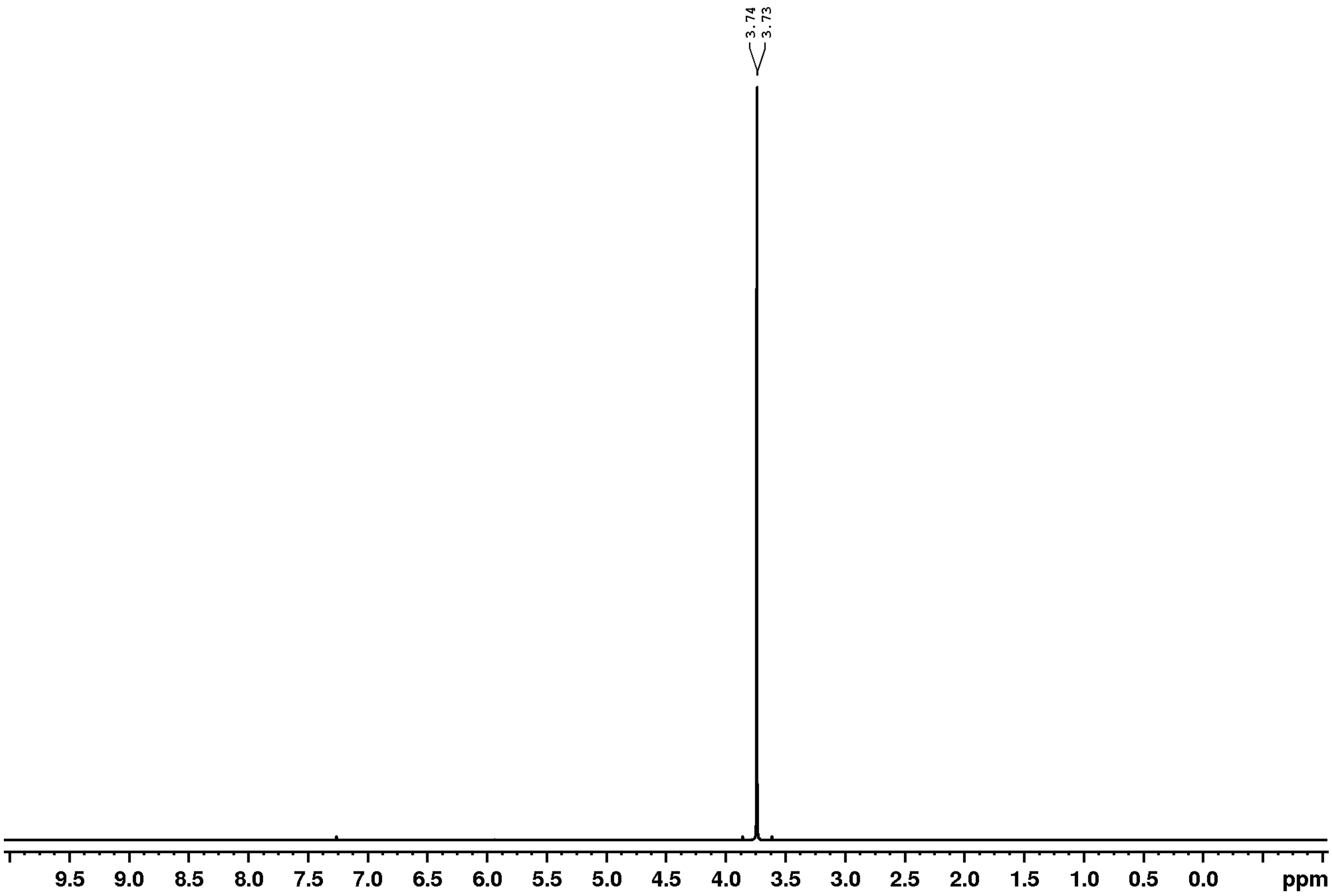

Figure S154. $^{1}H\{^{19}F\}$ NMR of $(CF_3)_2CFCF_2OCH_3/CF_3CF_2CF_2CF_2OCH_3$ in $CDCl_3$.

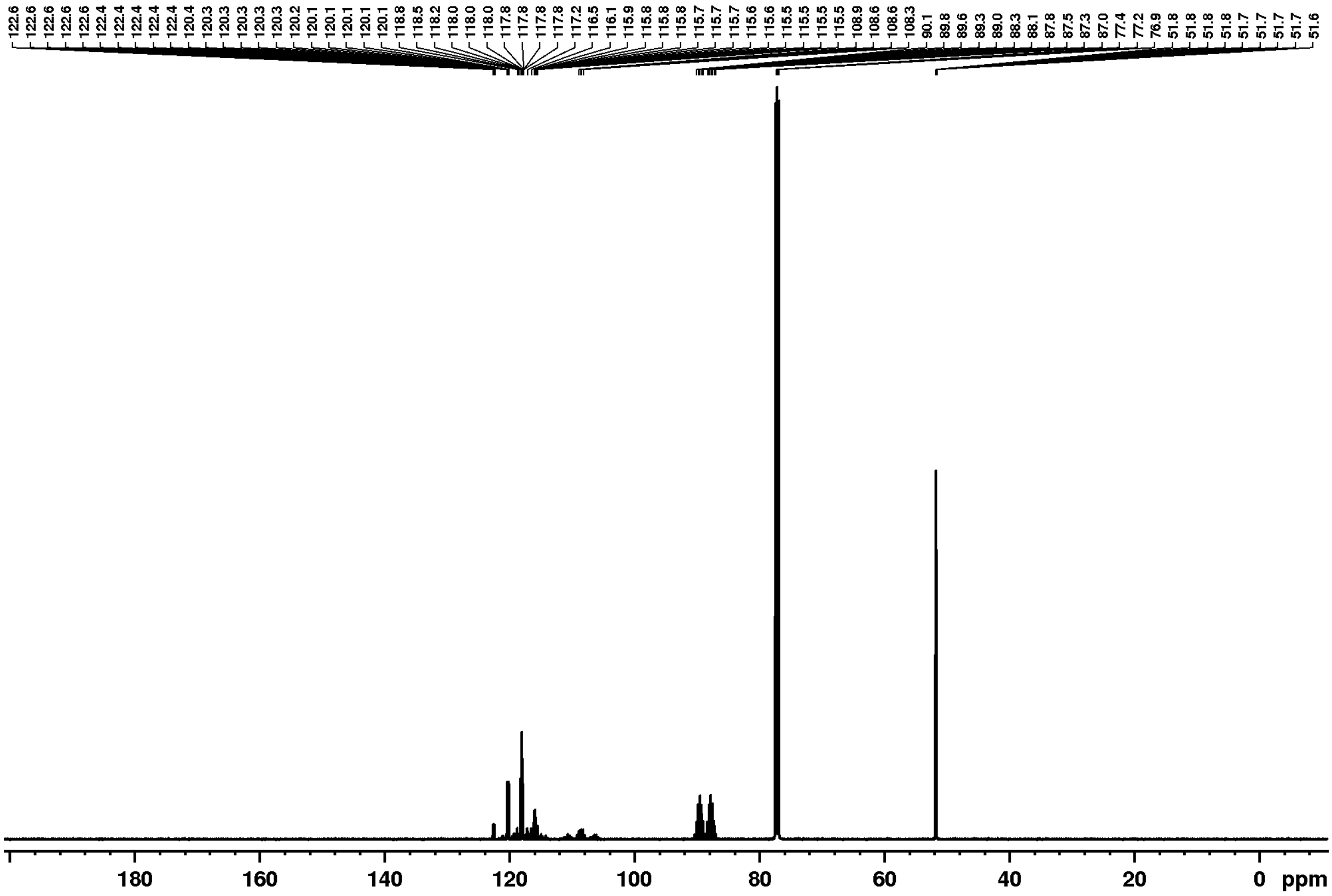

Figure S155. $^{13}C\{^{1}H\}$ NMR of $(CF_3)_2CFCF_2OCH_3/CF_3CF_2CF_2CF_2OCH_3$ in $CDCl_3$.

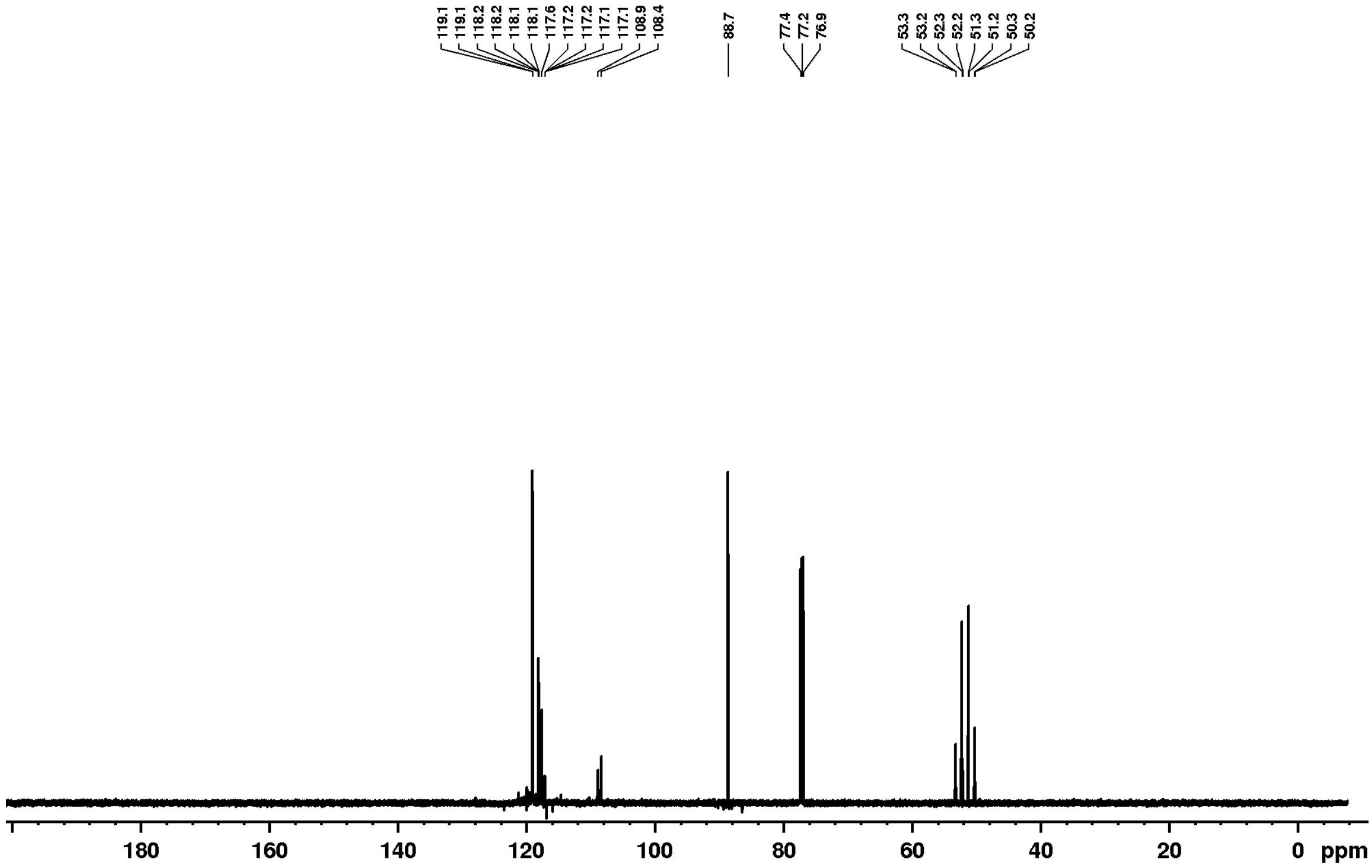

Figure S156. $^{13}C\{^{19}F\}$ NMR of $(CF_3)_2CFCF_2OCH_3/CF_3CF_2CF_2CF_2OCH_3$ in $CDCl_3$.

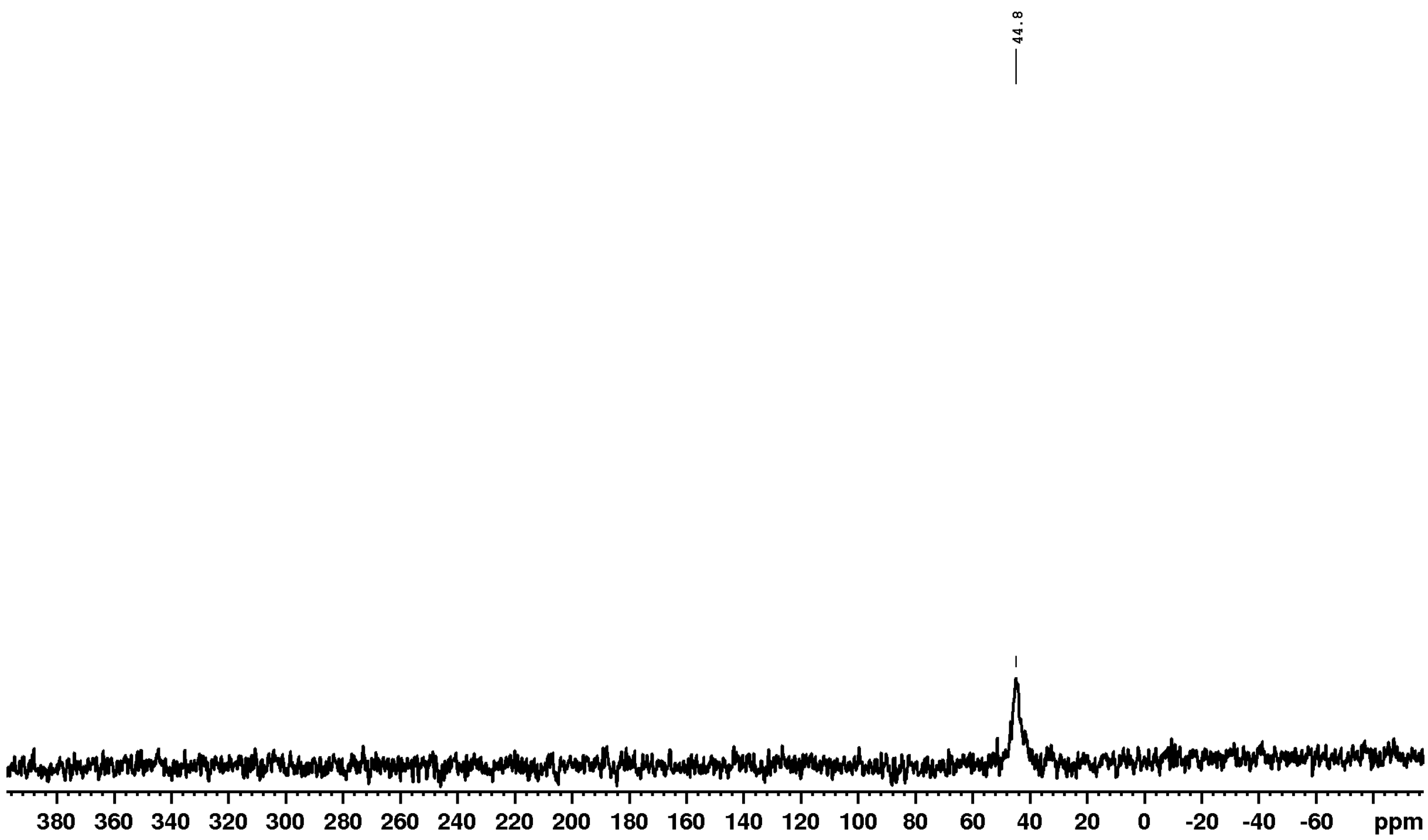

Figure S157. ^{17}O NMR of $(CF_3)_2CFCF_2OCH_3/CF_3CF_2CF_2CF_2OCH_3$ in $CDCl_3$.

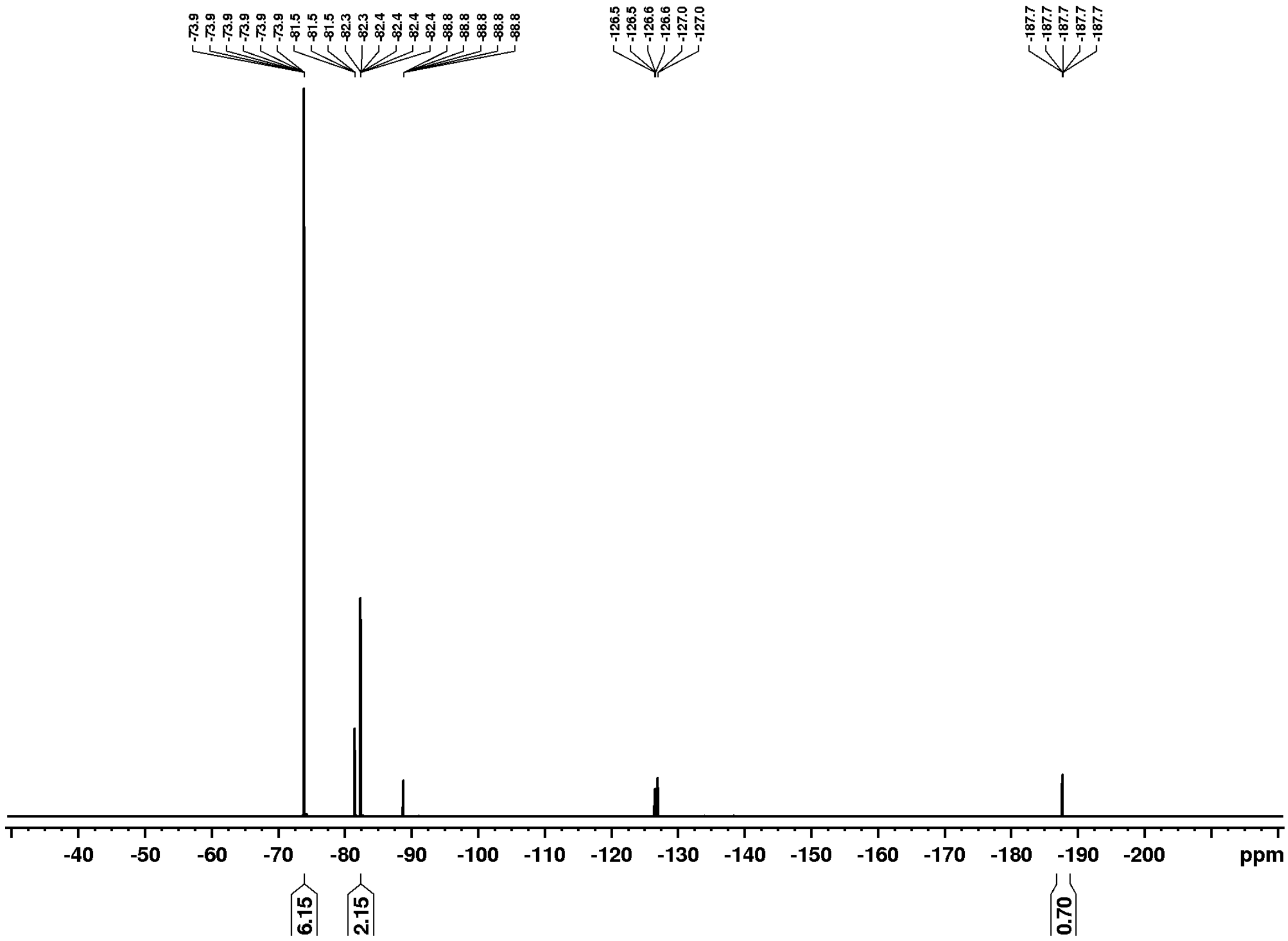

Figure S158. ^{19}F NMR of $(CF_3)_2CFCF_2OCH_3/CF_3CF_2CF_2CF_2OCH_3$ in $CDCl_3$, integrals shown for $(CF_3)_2CFCF_2OCH_3$.

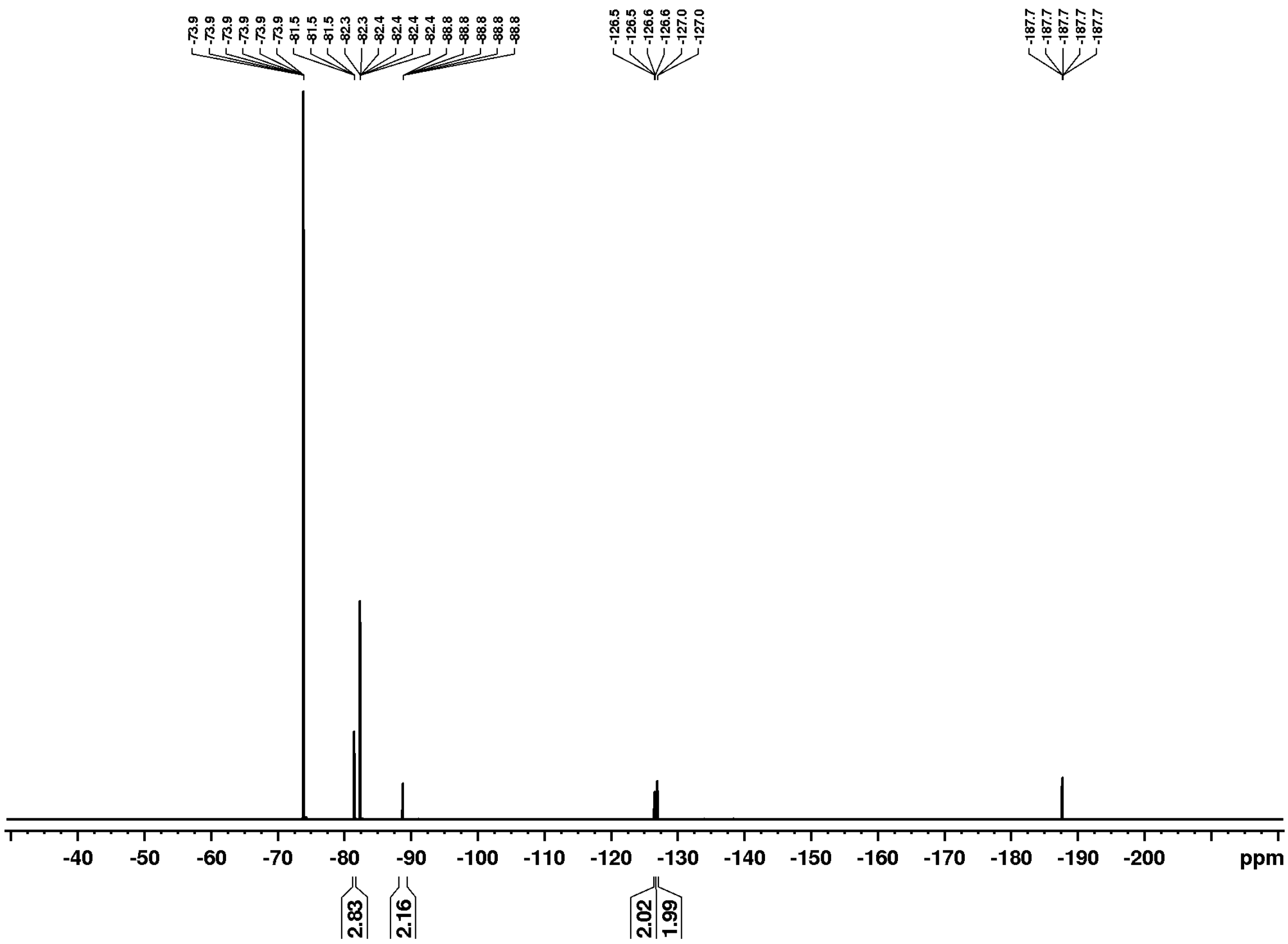

Figure S159. ^{19}F NMR of $(CF_3)_2CFCF_2OCH_3/CF_3CF_2CF_2CF_2OCH_3$ in $CDCl_3$, integrals shown for $CF_3CF_2CF_2CF_2OCH_3$.

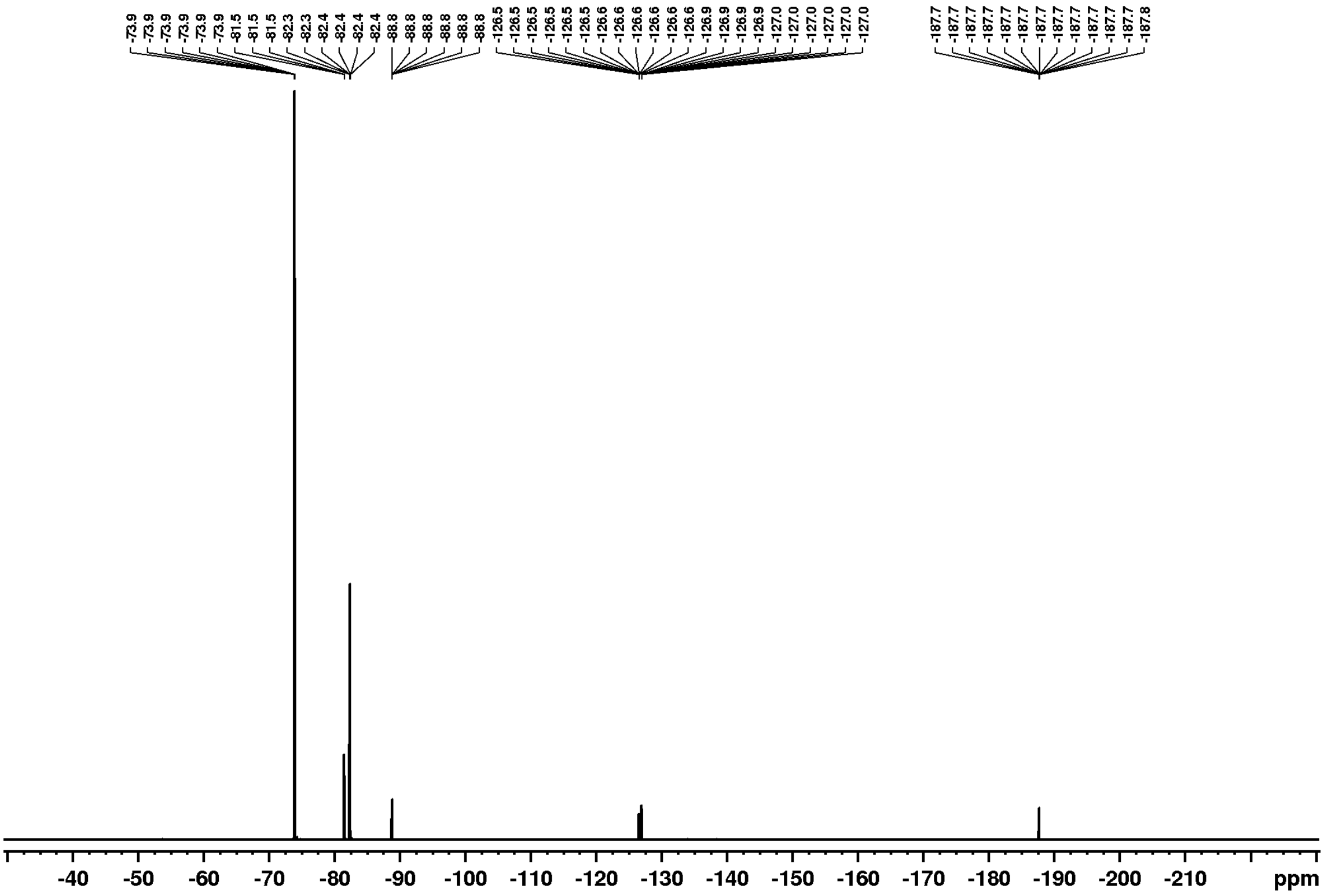

Figure S160. $^{19}F\{^{1}H\}$ NMR of $(CF_3)_2CFCF_2OCH_3/CF_3CF_2CF_2CF_2OCH_3$ in $CDCl_3$.

***i*+*n*-$C_4F_9OCH_2F$** (not isolated, mixture with *i*+*n*-$C_4F_9OCHF_2$ and *i*+*n*-$C_4F_9OCH_3$)

^{1}H NMR (400.3 MHz, $(CH_3)_2CO$): δ = 4.83 (d, 4H, $(CF_3)_2CFCF_2OC\mathbf{H}_2F/CF_3CF_2CF_2CF_2OC\mathbf{H}_2F$, $^2J(^{19}F, ^1H)$ = 50.7 Hz) ppm.

^{1}H{^{19}F} NMR (400.3 MHz, $(CH_3)_2CO$): δ = 4.83 (s, 4H, $(CF_3)_2CFCF_2OC\mathbf{H}_2F/CF_3CF_2CF_2CF_2OC\mathbf{H}_2F$) ppm.

^{19}F NMR (376.6 MHz, $(CH_3)_2CO$): δ = −75.3 (dt, 6F, $(C\mathbf{F}_3)_2CFCF_2OCH_2F$, $^3J(^{19}F, ^{19}F)$ = 5.6 Hz, $^4J(^{19}F, ^{19}F)$ = 9.9 −80.9 (m, 2F, $(CF_3)_2CFC\mathbf{F}_2OCH_2F$), −83.2 (m, 3F, $C\mathbf{F}_3CF_2CF_2CF_2OCH_2F$), −87.7 (m, 2F, $CF_3CF_2CF_2C\mathbf{F}_2OCH_2F$), −128.1(m, 4F, $CF_3C\mathbf{F}_2C\mathbf{F}_2CF_2OCH_2F$), −156.8 (tt, 1F, $CF_3CF_2CF_2CF_2OCH_2\mathbf{F}$, $^2J(^{19}F, ^1H)$ = 50.8 Hz, $^4J(^{19}F, ^{19}F)$ = 9.3 Hz), −157.1 (tt, 1F, $(CF_3)_2CFCF_2OCH_2\mathbf{F}$, $^2J(^{19}F, ^1H)$ = 50.7 Hz, $^4J(^{19}F, ^{19}F)$ = 9.6 Hz), −189.2 (m, 1F, $(CF_3)_2C\mathbf{F}CF_2OCH_2F$) ppm.**^{19}F{^{1}H} NMR** (376.6 MHz, $(CH_3)_2CO$): δ = −75.3 (dt, 6F, $(C\mathbf{F}_3)_2CFCF_2OCH_2F$, $^3J(^{19}F, ^{19}F)$ = 5.6 Hz, $^4J(^{19}F, ^{19}F)$ = 9.9 −80.9 (m, 2F, $(CF_3)_2CFC\mathbf{F}_2OCH_2F$), −83.2 (m, 3F, $C\mathbf{F}_3CF_2CF_2CF_2OCH_2F$), −87.7 (m, 2F, $CF_3CF_2CF_2C\mathbf{F}_2OCH_2F$), −128.1(m, 4F, $CF_3C\mathbf{F}_2C\mathbf{F}_2CF_2OCH_2F$), −156.8 (t, 1F, $CF_3CF_2CF_2CF_2OCH_2\mathbf{F}$, $^4J(^{19}F, ^{19}F)$ = 9.3 Hz), −157.1 (t, 1F, $(CF_3)_2CFCF_2OCH_2\mathbf{F}$, $^4J(^{19}F, ^{19}F)$ = 9.6 Hz), −189.2 (m, 1F, $(CF_3)_2C\mathbf{F}CF_2OCH_2F$) ppm.

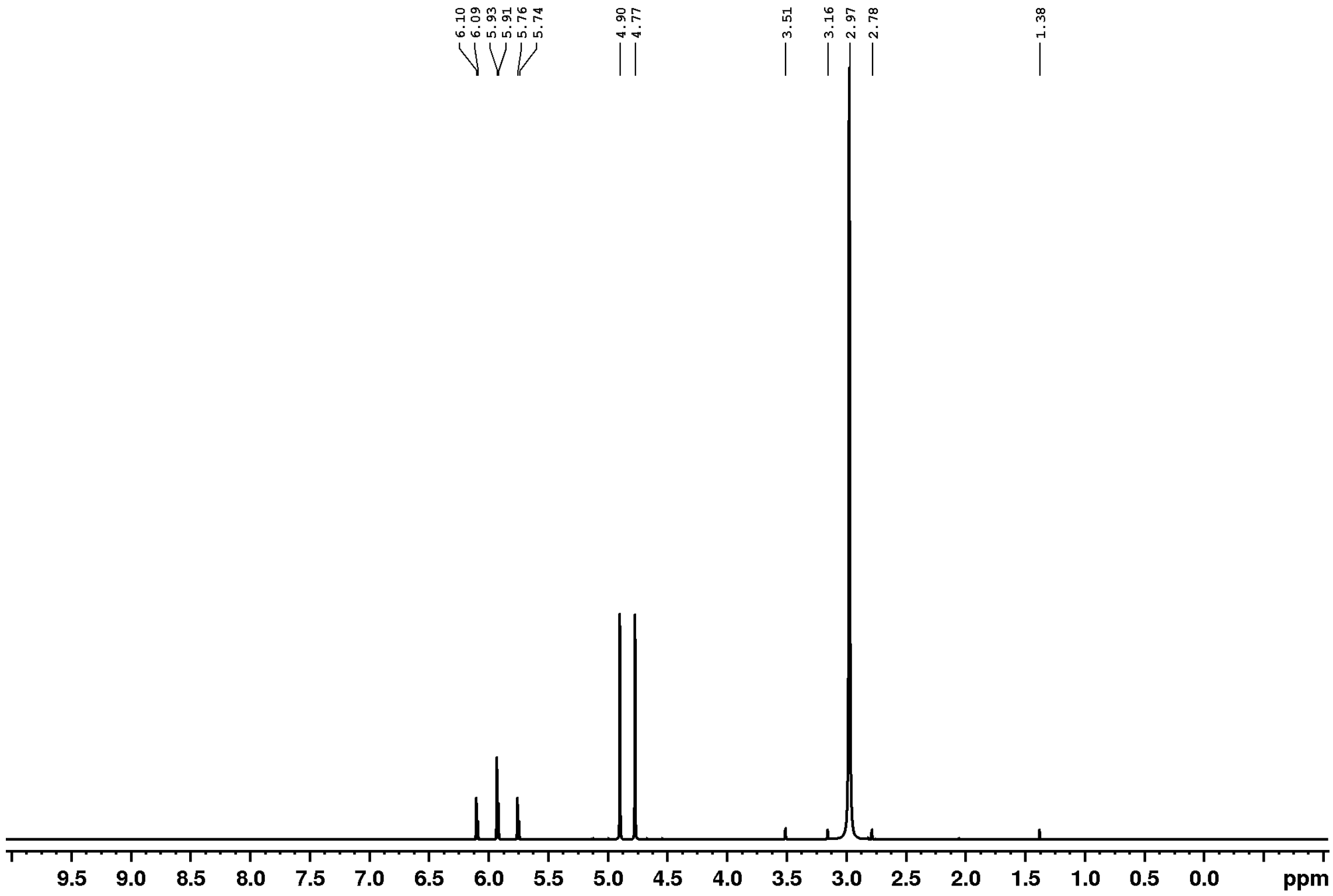

Figure S161. ^1H NMR of $(CF_3)_2CFCF_2OCH_2F/CF_3CF_2CF_2CF_2OCH_2F$ (mixture with *i*+*n*-$C_4F_9OCHF_2$ and *i*+*n*-$C_4F_9OCH_3$) measured as neat substance with acetone-d_6 and CCl_3F as external standard, integrals not shown as signals overlap.

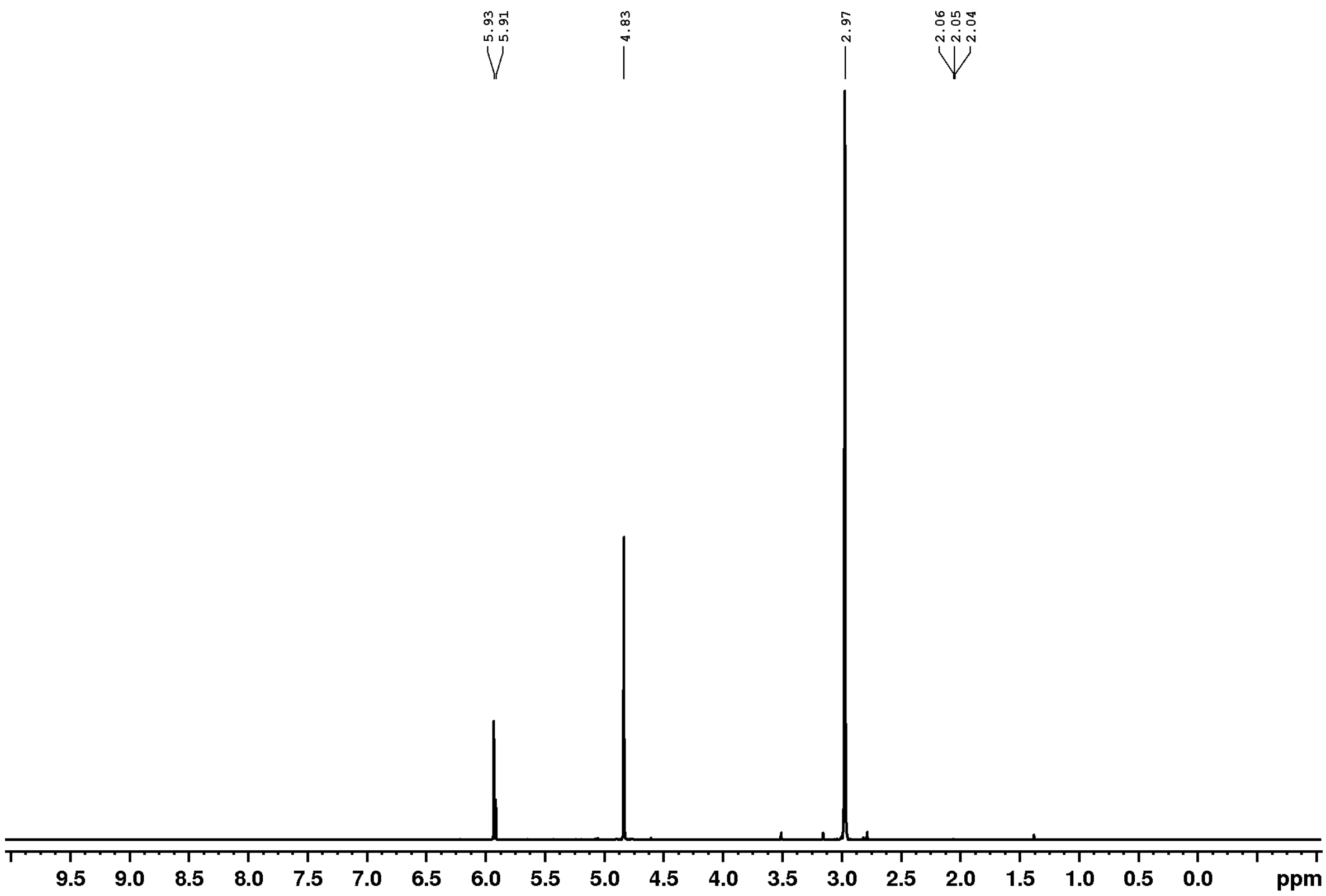

Figure S162. $^1H\{^{19}F\}$ NMR of $(CF_3)_2CFCF_2OCH_2F/CF_3CF_2CF_2CF_2OCH_2F$ (mixture with *i*+*n*-$C_4F_9OCHF_2$ and *i*+*n*-$C_4F_9OCH_3$) measured as neat substance with acetone-d_6 and CCl_3F as external standard.

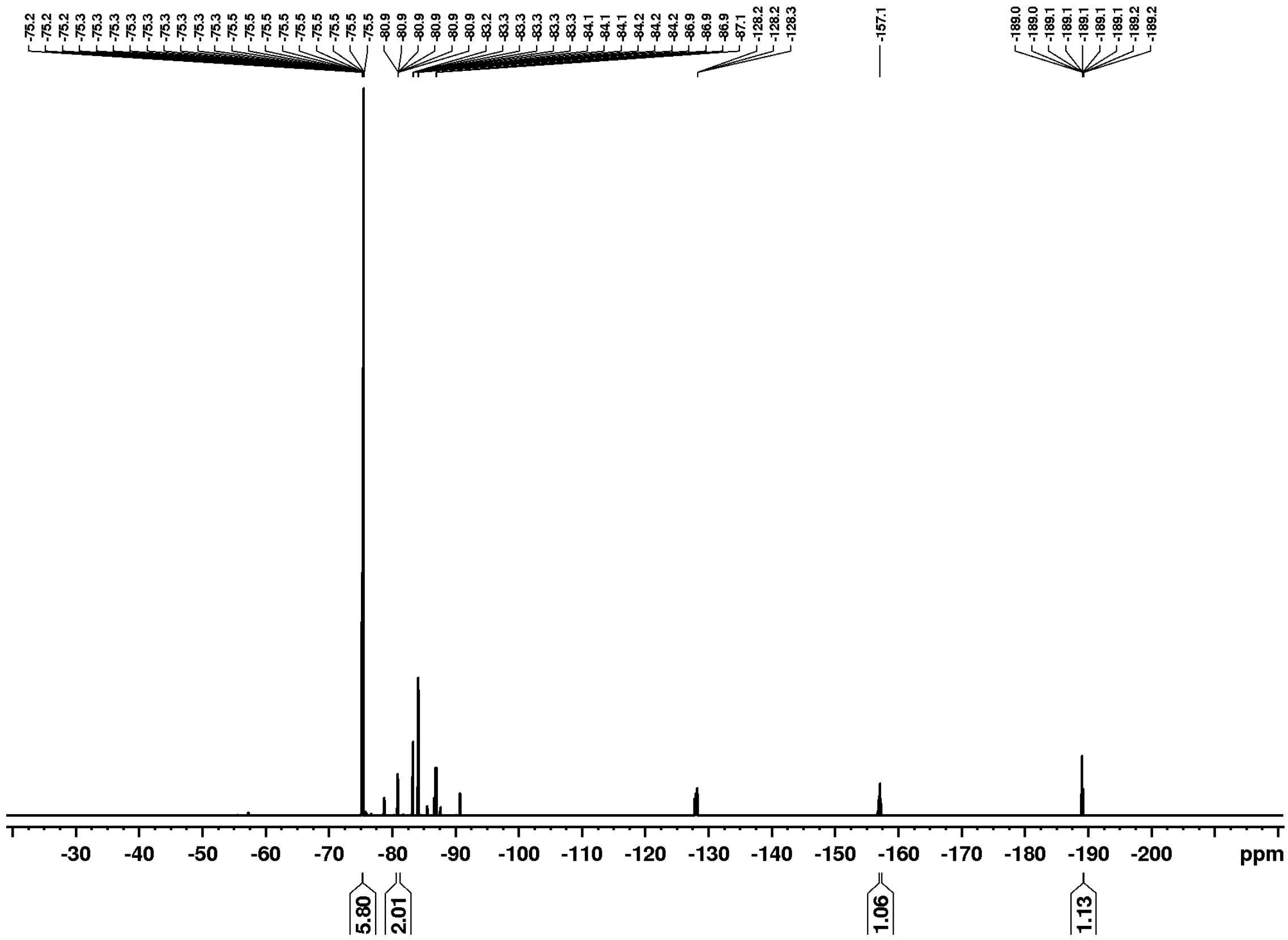

Figure S163. ^{19}F NMR of $(CF_3)_2CFCF_2OCH_2F/CF_3CF_2CF_2CF_2OCH_2F$ (mixture with *i*+*n*-$C_4F_9OCHF_2$ and *i*+*n*-$C_4F_9OCH_3$) measured as neat substance with acetone-d_6 and CCl_3F as external standard, integrals shown for $(CF_3)_2CFCF_2OCH_2F$. Integrals for $CF_3CF_2CF_2CF_2OCH_2F$ not shown as signals overlap.

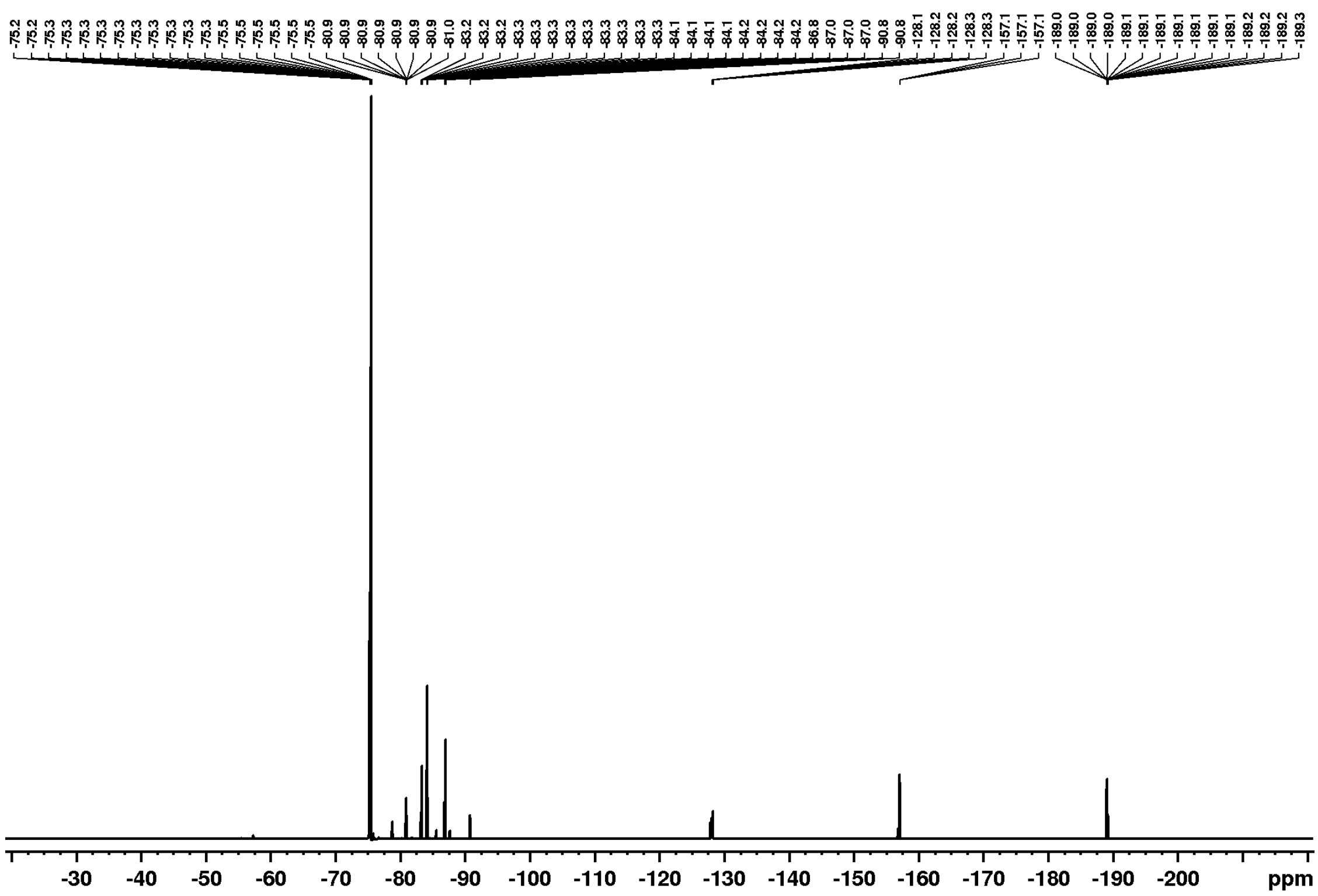

Figure S164. $^{19}F\{^1H\}$ NMR of $(CF_3)_2CFCF_2OCH_2F/CF_3CF_2CF_2CF_2OCH_2F$ (mixture with *i*+*n*-$C_4F_9OCHF_2$ and *i*+*n*-$C_4F_9OCH_3$) measured as neat substance with acetone-d_6 and CCl_3F as external standard.

***i+n*-$C_4F_9OCHF_2$**

Boiling point (distillation): 49.5 °C at 1000 mbar.

Boiling point (vapor pressure): T_{bp} = 49.8 °C at 1013 mbar (Antoine parameter: A = 4.190; B = 1156.5; C = −46.614 for equation: $p = 10^{(A-B/(T+C))}$).

Enthalpy of vaporization (T = 298.15 K): $\Delta_{vap}H$ = 31.1 kJ mol^{-1} (for equation: $\Delta_{vap}H = \ln(10)\cdot R\cdot T^2\cdot(B/(T+C)^2)/1000$).

^{1}H NMR (500.1 MHz, $(CH_3)_2CO$): δ = 5.90 (t, 1H, $(CF_3)_2CFCF_2OC\mathbf{H}F_2$, $^2J(^{19}F, ^1H)$ = 69.1 Hz), 5.88 (t, 1H, $CF_3CF_2CF_2CF_2OC\mathbf{H}F_2$, $^2J(^{19}F, ^1H)$ = 69.2 Hz) ppm.

^{1}H{^{19}F} NMR (400.3 MHz, $(CH_3)_2CO$): δ = 5.89 (s, 1H, $(CF_3)_2CFCF_2OC\mathbf{H}F_2$), 5.88 (s, 1H, $CF_3CF_2CF_2CF_2OC\mathbf{H}F_2$) ppm.

^{13}C{^{1}H} NMR (125.8 MHz, $(CH_3)_2CO$): δ = 118.6 (qdm, 2C, $(\mathbf{C}F_3)_2CFCF_2OCHF_2$, $^1J(^{19}F, ^{13}C)$ = 286.6 Hz, $^2J(^{19}F, ^{13}C)$ = 26.2 Hz), 117.3 (qm, 1C, $\mathbf{C}F_3CF_2CF_2CF_2OCHF_2$, $^1J(^{19}F, ^{13}C)$ = 286.4 Hz), 116.5 (tdt, 1C, $(CF_3)_2CF\mathbf{C}F_2OCHF_2$, $^1J(^{19}F, ^{13}C)$ = 281.1 Hz, $^2J(^{19}F, ^{13}C)$ = 25.6 Hz, $^3J(^{19}F, ^{13}C)$ = 4.3 Hz), 115.2 (tt, 1C, $CF_3CF_2CF_2\mathbf{C}F_2OCHF_2$, $^1J(^{19}F, ^{13}C)$ = 281.4, $^2J(^{19}F, ^{13}C)$ = 30.0 Hz), 112.1 (tt, 1C, $CF_3CF_2CF_2CF_2O\mathbf{C}HF_2$, $^1J(^{19}F, ^{13}C)$ = 264.6 Hz, $^3J(^{19}F, ^{13}C)$ = 6.4 Hz), 112.0 (ttd, 1C, $(CF_3)_2CFCF_2O\mathbf{C}HF_2$, $^1J(^{19}F, ^{13}C)$ = 264.6 Hz, $^3J(^{19}F, ^{13}C)$ = 6.2 Hz, $^4J(^{19}F, ^{13}C)$ = 0.9 Hz), 108.3 (tm, 1C, $CF_3CF_2\mathbf{C}F_2CF_2OCHF_2$, $^1J(^{19}F, ^{13}C)$ = 321.3 Hz), 108.2 (tm, 1C, $CF_3\mathbf{C}F_2CF_2CF_2OCHF_2$, $^1J(^{19}F, ^{13}C)$ = 296.3 Hz), 88.3 (dseptm, 1C, $(CF_3)_2\mathbf{C}FCF_2OCHF_2$, $^1J(^{19}F, ^{13}C)$ = 221.9 Hz, $^2J(^{19}F, ^{13}C)$ = 33.0 Hz) ppm.

^{13}C{^{19}F} NMR (125.8 MHz, $(CH_3)_2CO$): δ = 118.6 (s, 2C, $(\mathbf{C}F_3)_2CFCF_2OCHF_2$), 117.3 (s, 1C, $\mathbf{C}F_3CF_2CF_2CF_2OCHF_2$), 116.5 (d, 1C, $(CF_3)_2CF\mathbf{C}F_2OCHF_2$, $^3J(^{13}C, ^1H)$ = 1.7 Hz), 115.3 (d, 1C, $CF_3CF_2CF_2\mathbf{C}F_2OCHF_2$, $^3J(^{13}C, ^1H)$ = 2.0 Hz), 112.1 (d, 1C, $CF_3CF_2CF_2CF_2O\mathbf{C}HF_2$, $^1J(^{13}C, ^1H)$ = 225.21C, $(CF_3)_2CFCF_2O\mathbf{C}HF_2$, $^1J(^{13}C, ^1H)$ = 225.3 Hz), 108.3 (s, 1C, $CF_3CF_2\mathbf{C}F_2CF_2OCHF_2$), 108.2 (s, 1C, $CF_3\mathbf{C}F_2CF_2CF_2OCHF_2$), 88.3 (d, 1C, $(CF_3)_2\mathbf{C}FCF_2OCHF_2$, $^4J(^{13}C, ^1H)$ = 3.8 Hz) ppm.**^{17}O NMR** (54.3 MHz, $(CH_3)_2CO$): δ = 129.3 (s, 1O, $(CF_3)_2CFCF_2\mathbf{O}CHF_2$, $CF_3CF_2CF_2CF_2\mathbf{O}CHF_2$) ppm.

^{19}F NMR (470.5 MHz, $(CH_3)_2CO$): δ = −76.5 (dt, 6F, $(C\mathbf{F}_3)_2CFCF_2OCHF_2$, $^3J(^{19}F, ^{19}F)$ = 5.7 Hz, $^4J(^{19}F, ^{19}F)$ = 9.9 −80.0 (m, 2F, $(CF_3)_2CFC\mathbf{F}_2OCHF_2$), −84.5 (tm, 3F, $C\mathbf{F}_3CF_2CF_2CF_2OCHF_2$, $^3J(^{19}F, ^{19}F)$ = 9.5 Hz), −86.8 (m, 2F, $CF_3CF_2CF_2C\mathbf{F}_2OCHF_2$), −88.1 (dt, 2F, $CF_3CF_2CF_2CF_2OCH\mathbf{F}_2$, $^2J(^{19}F, ^1H)$ = 69.2 Hz, $^4J(^{19}F, ^{19}F)$ = 4.6 Hz), −88.3 (dt, 2F, $(CF_3)_2CFCF_2OCH\mathbf{F}_2$, $^2J(^{19}F, ^1H)$ = 69.2 Hz, $^4J(^{19}F, ^{19}F)$ = 4.7 Hz), −129.3 (m, 4F, $CF_3C\mathbf{F}_2C\mathbf{F}_2CF_2OCHF_2$), −190.5 (m, 1F, $(CF_3)_2C\mathbf{F}CF_2OCHF_2$) ppm.**^{19}F{^{1}H} NMR** (376.6 MHz, $(CH_3)_2CO$): δ = −76.5 (dt, 6F, $(C\mathbf{F}_3)_2CFCF_2OCHF_2$, $^3J(^{19}F, ^{19}F)$ = 5.7 Hz, $^4J(^{19}F, ^{19}F)$ = 9.9 −80.0 (m, 2F, $(CF_3)_2CFC\mathbf{F}_2OCHF_2$), −84.5 (tm, 3F, $C\mathbf{F}_3CF_2CF_2CF_2OCHF_2$, $^3J(^{19}F, ^{19}F)$ = 9.5 Hz), −86.8 (m, 2F, $CF_3CF_2CF_2C\mathbf{F}_2OCHF_2$), −88.1 (t, 2F, $CF_3CF_2CF_2CF_2OCH\mathbf{F}_2$, $^4J(^{19}F, ^{19}F)$ = 4.6 Hz), −88.3 (t, 2F, $(CF_3)_2CFCF_2OCH\mathbf{F}_2$, $^4J(^{19}F, ^{19}F)$ = 4.7 Hz), −129.3 (m, 2F, $CF_3C\mathbf{F}_2C\mathbf{F}_2CF_2OCHF_2$), −190.5 (m, 1F, $(CF_3)_2C\mathbf{F}CF_2OCHF_2$) ppm.GC-MS [t = 0.44 min] m/z$^+$: 219 [M $-OCF_2H$], 197 [M $-HCF_4$], 169 [M $-CF_2OCHF_2$], 169 [M $-CF_2OCHF_2$], 150 [M $-CF_3OCHF_2$], 131 [M $-CF_4OCHF_2$], 117 [M $-C_3F_7$], 100 [M $-C_2F_5OCHF_2$], 69 [M −, 51 [M $-OC_4F_9$].

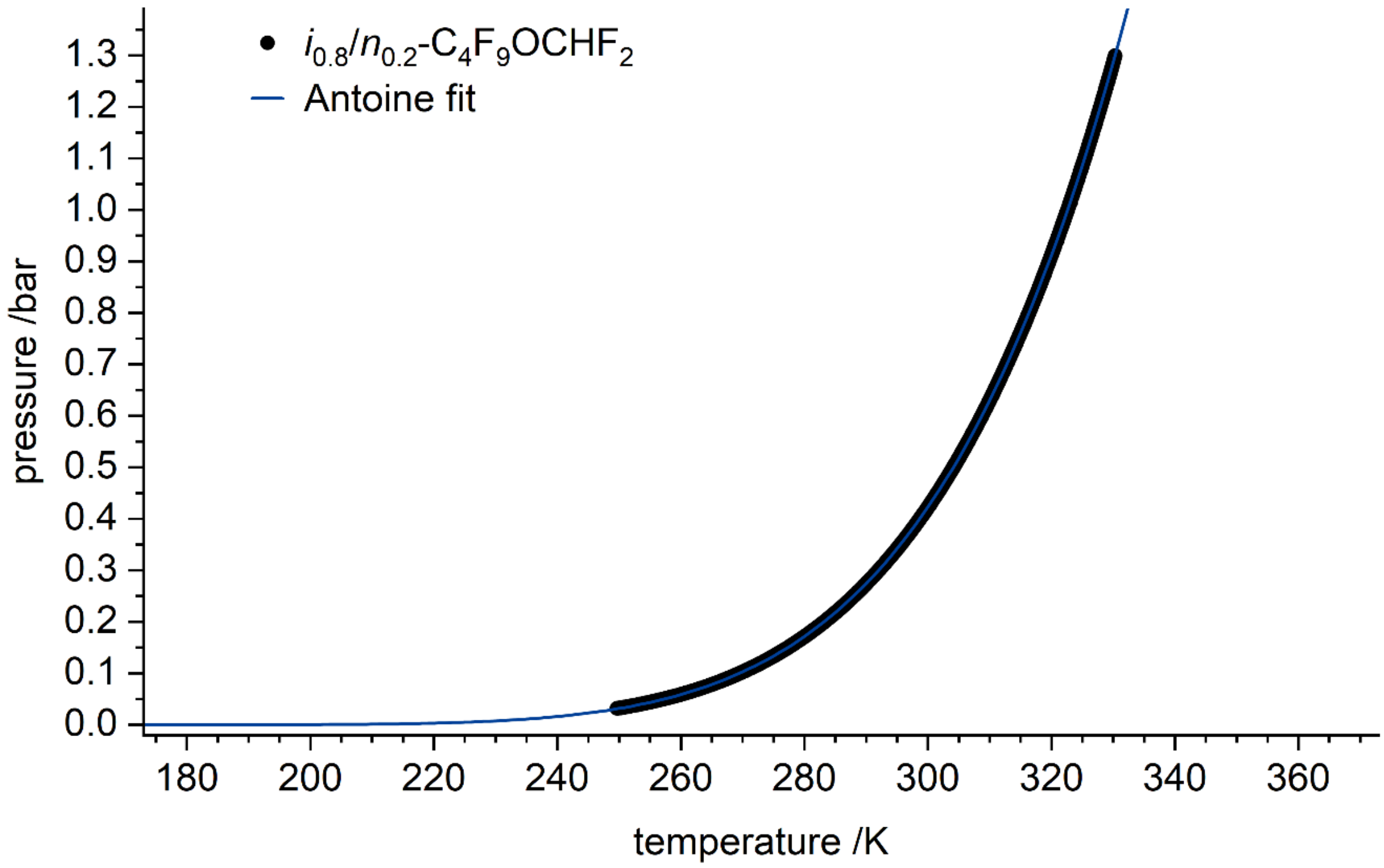

Figure S165. Vapor pressure measurement and Antoine fit of $i_{0.8}/n_{0.2}$-$C_4F_9OCHF_2$ (Antoine parameter: A = 4.190; B = 1156.5; C = −46.614 for equation: $p = 10^{(A-B/(T+C))}$).

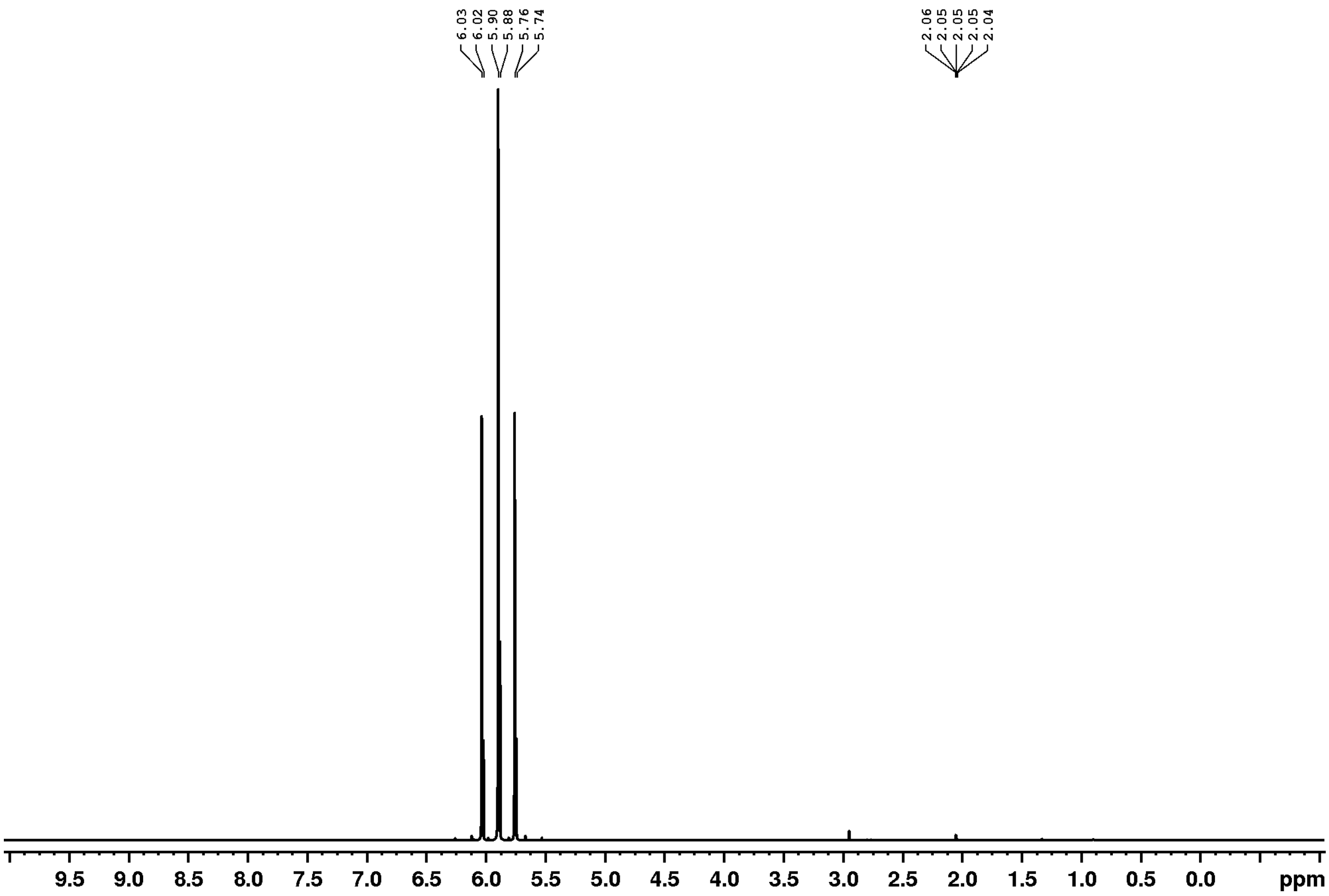

Figure S166. ^{1}H NMR of $(CF_3)_2CFCF_2OCHF_2$/$CF_3CF_2CF_2CF_2OCHF_2$ measured as neat substance with acetone-d_6 and CCl_3F as external standard, integrals not shown as signals overlap.

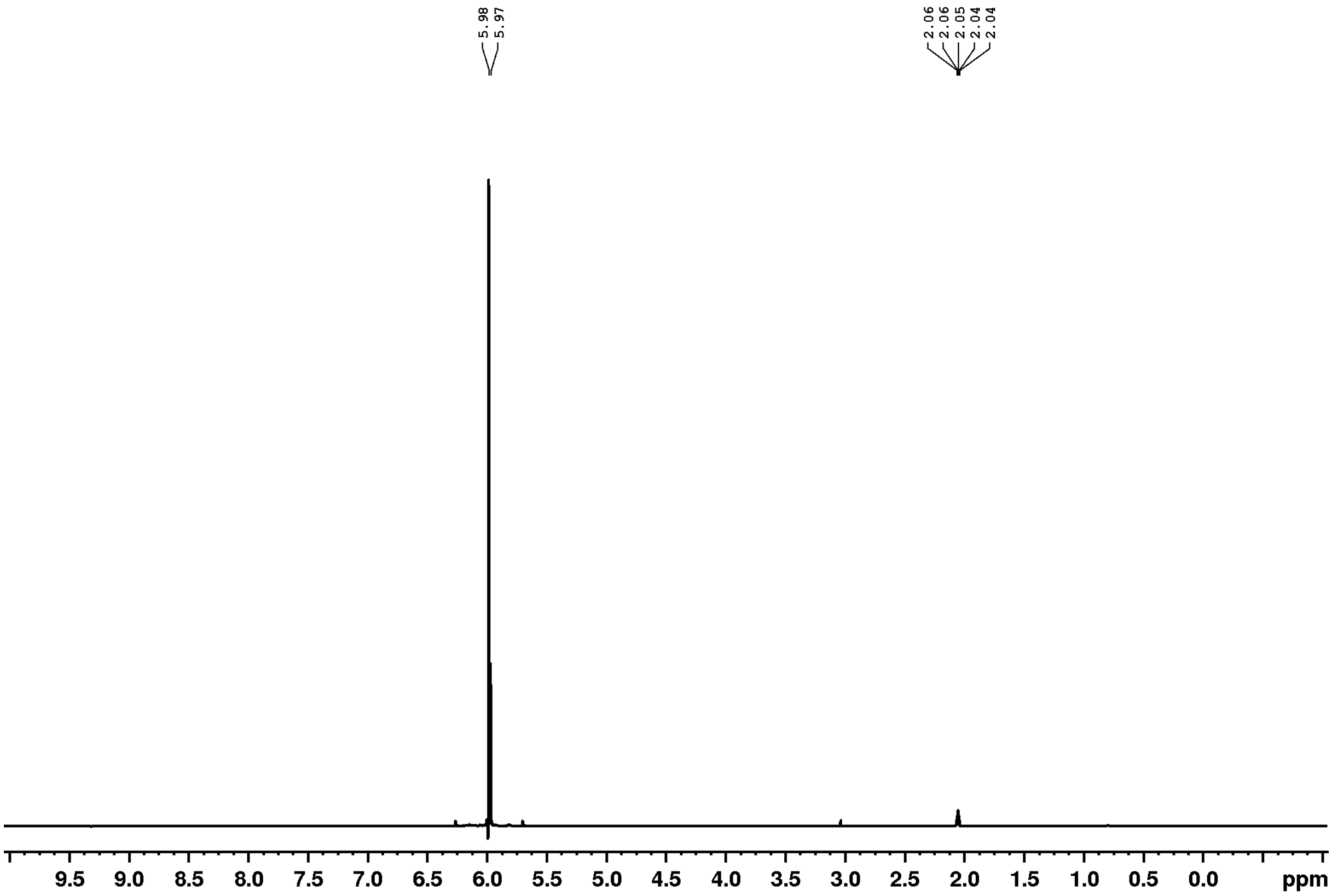

Figure S167. $^{1}H\{^{19}F\}$ NMR of $(CF_3)_2CFCF_2OCHF_2/CF_3CF_2CF_2CF_2OCHF_2$ measured as neat substance with acetone-d_6 and CCl_3F as external standard.

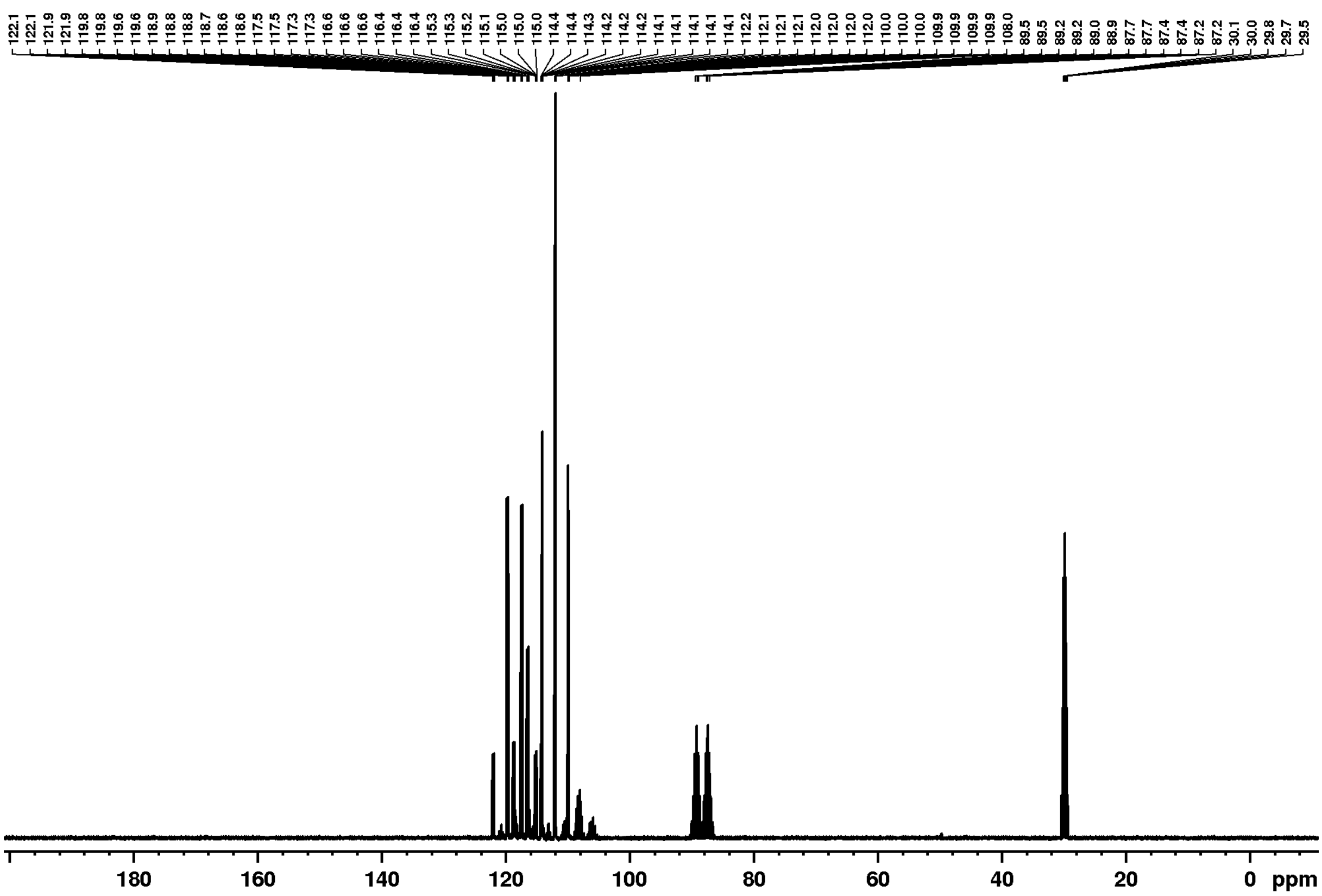

Figure S168. $^{13}C\{^{1}H\}$ NMR of $(CF_3)_2CFCF_2OCHF_2/CF_3CF_2CF_2CF_2OCHF_2$ measured as neat substance with acetone-d_6 and CCl_3F as external standard.

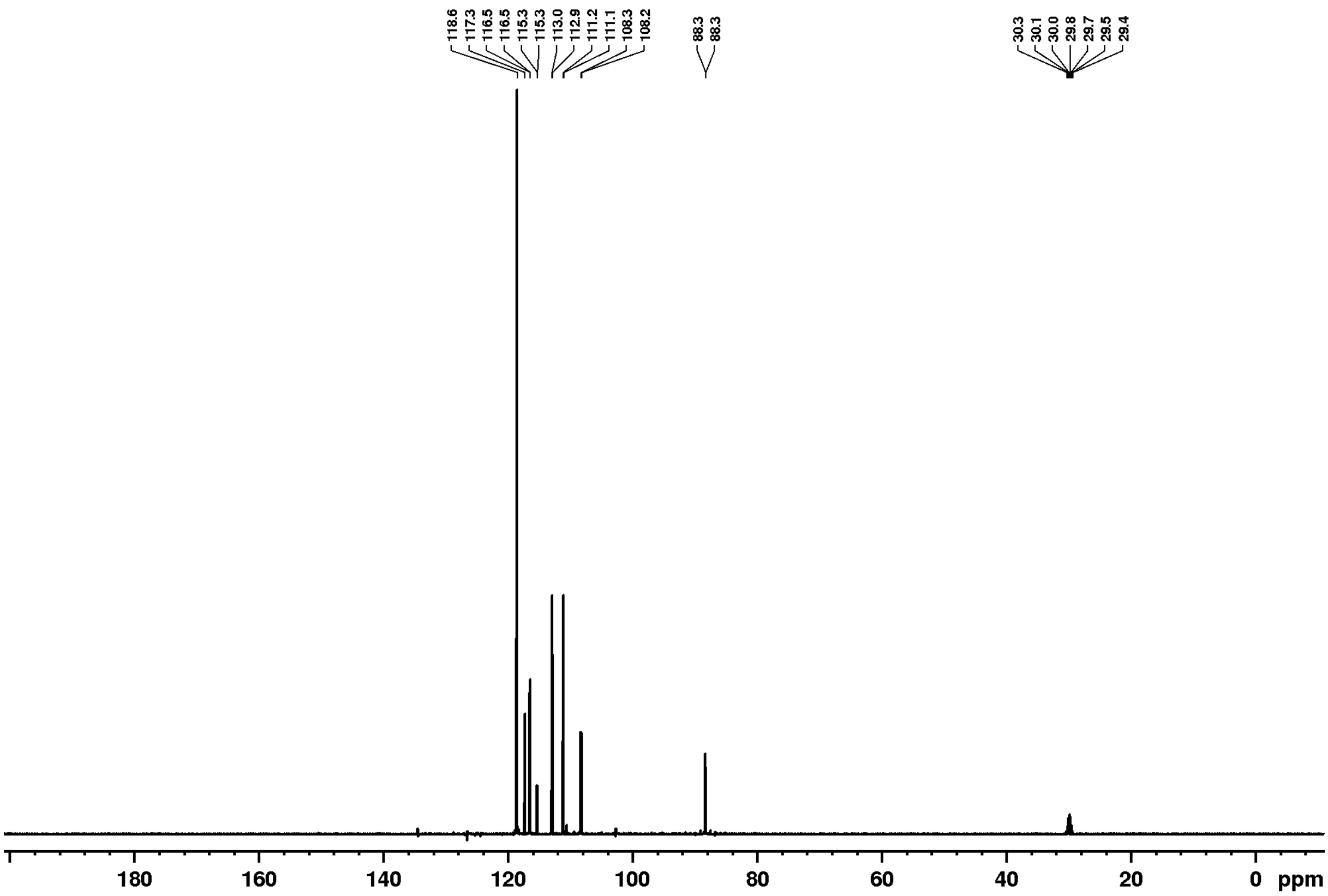

Figure S169. $^{13}C\{^{19}F\}$ NMR of $(CF_3)_2CFCF_2OCHF_2/CF_3CF_2CF_2CF_2OCHF_2$ measured as neat substance with acetone-d_6 and CCl_3F as external standard.

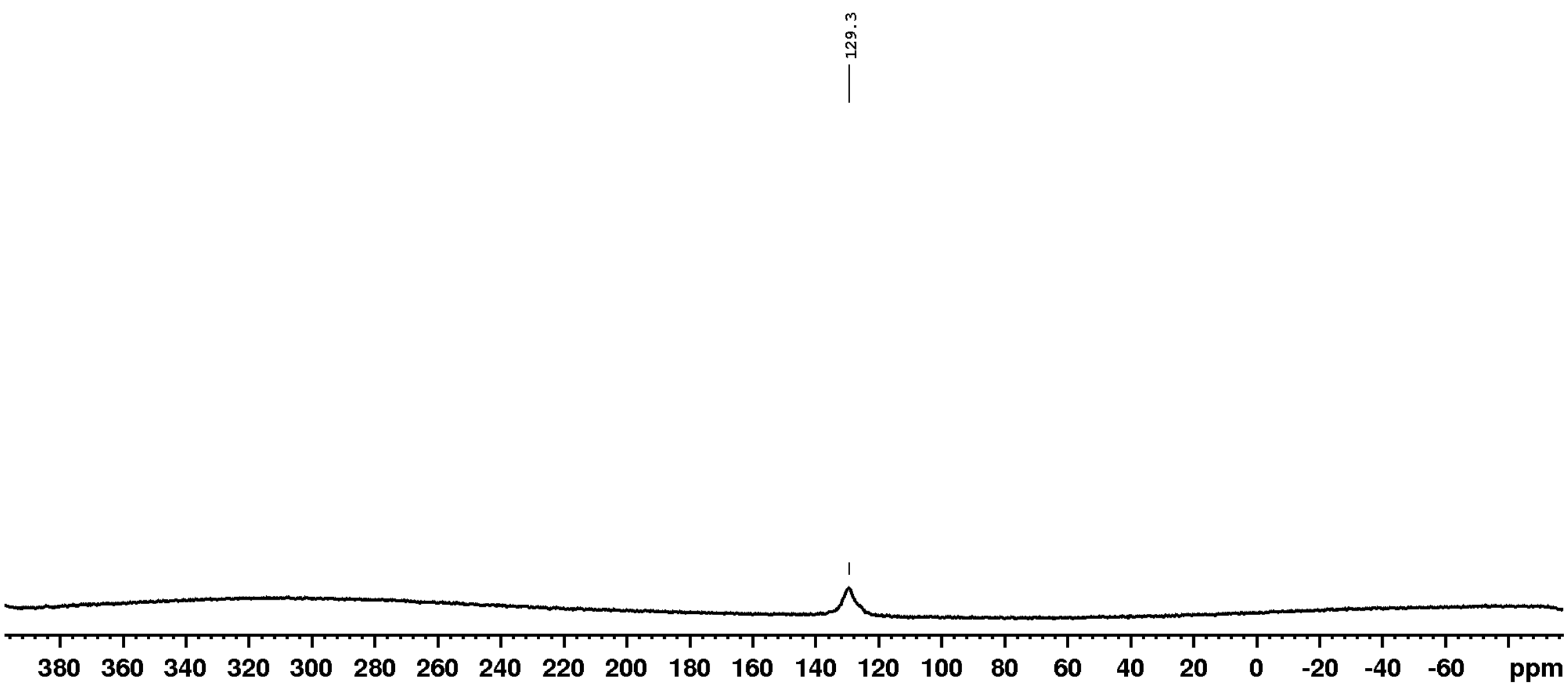

Figure S170. ^{17}O NMR of $(CF_3)_2CFCF_2OCHF_2/CF_3CF_2CF_2CF_2OCHF_2$ measured as neat substance with acetone-d_6 and CCl_3F as external standard.

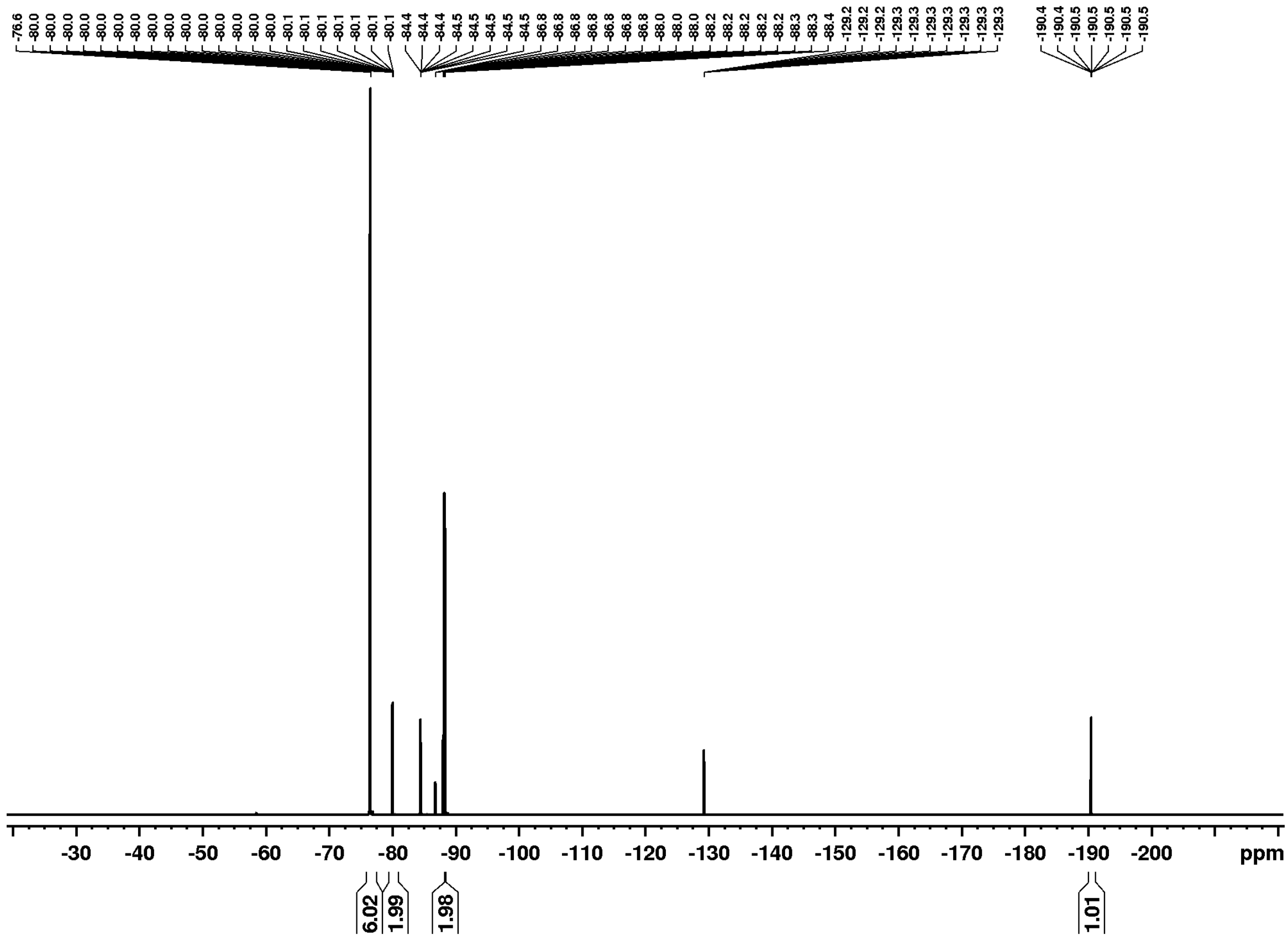

Figure S171. ^{19}F NMR of $(CF_3)_2CFCF_2OCHF_2/CF_3CF_2CF_2CF_2OCHF_2$ measured as neat substance with acetone-d_6 and CCl_3F as external standard, integrals shown for $(CF_3)_2CFCF_2OCHF_2$.

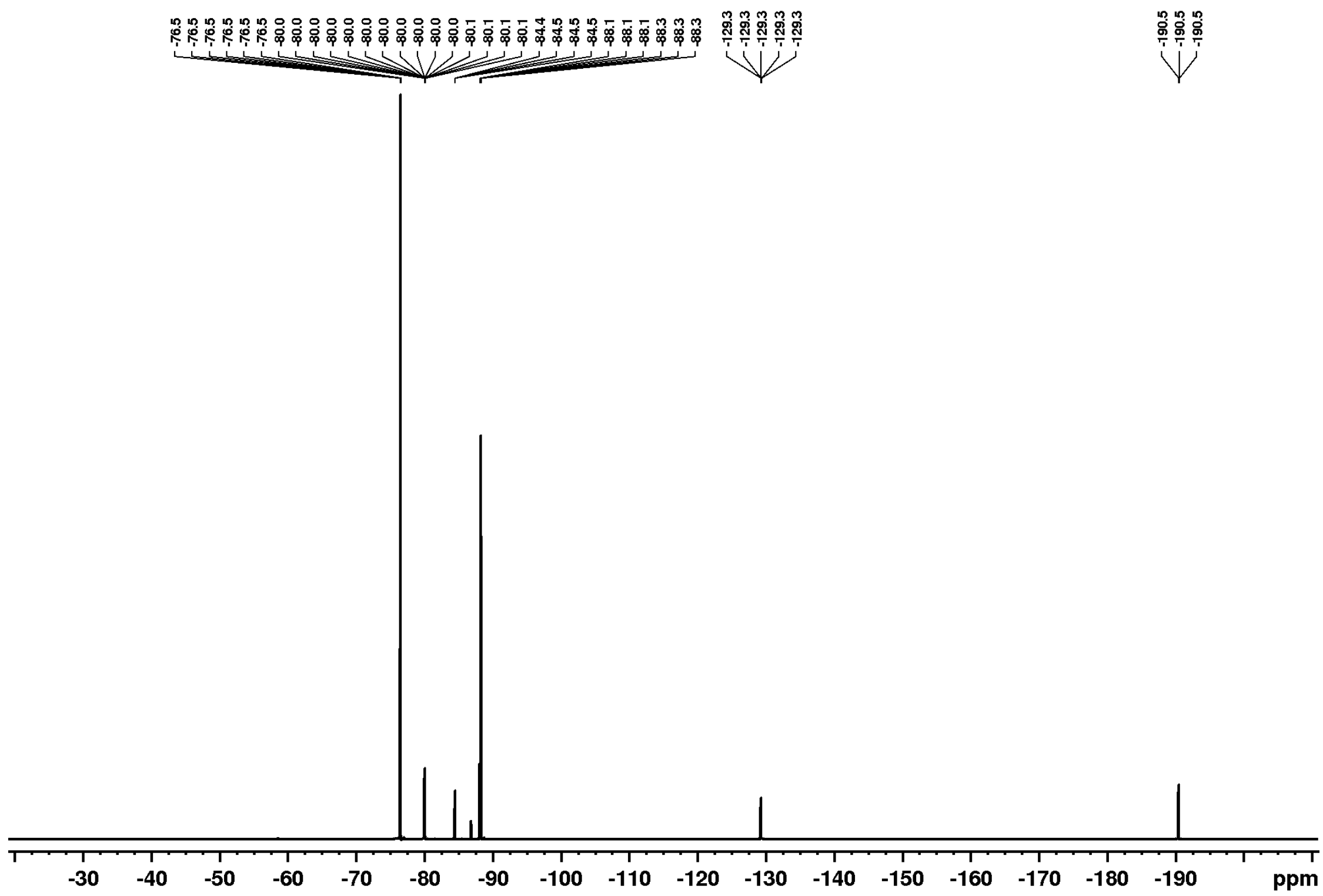

Figure S172. $^{19}F\{^1H\}$ NMR of $(CF_3)_2CFCF_2OCHF_2/CF_3CF_2CF_2CF_2OCHF_2$ measured as neat substance with acetone-d_6 and CCl_3F as external standard.

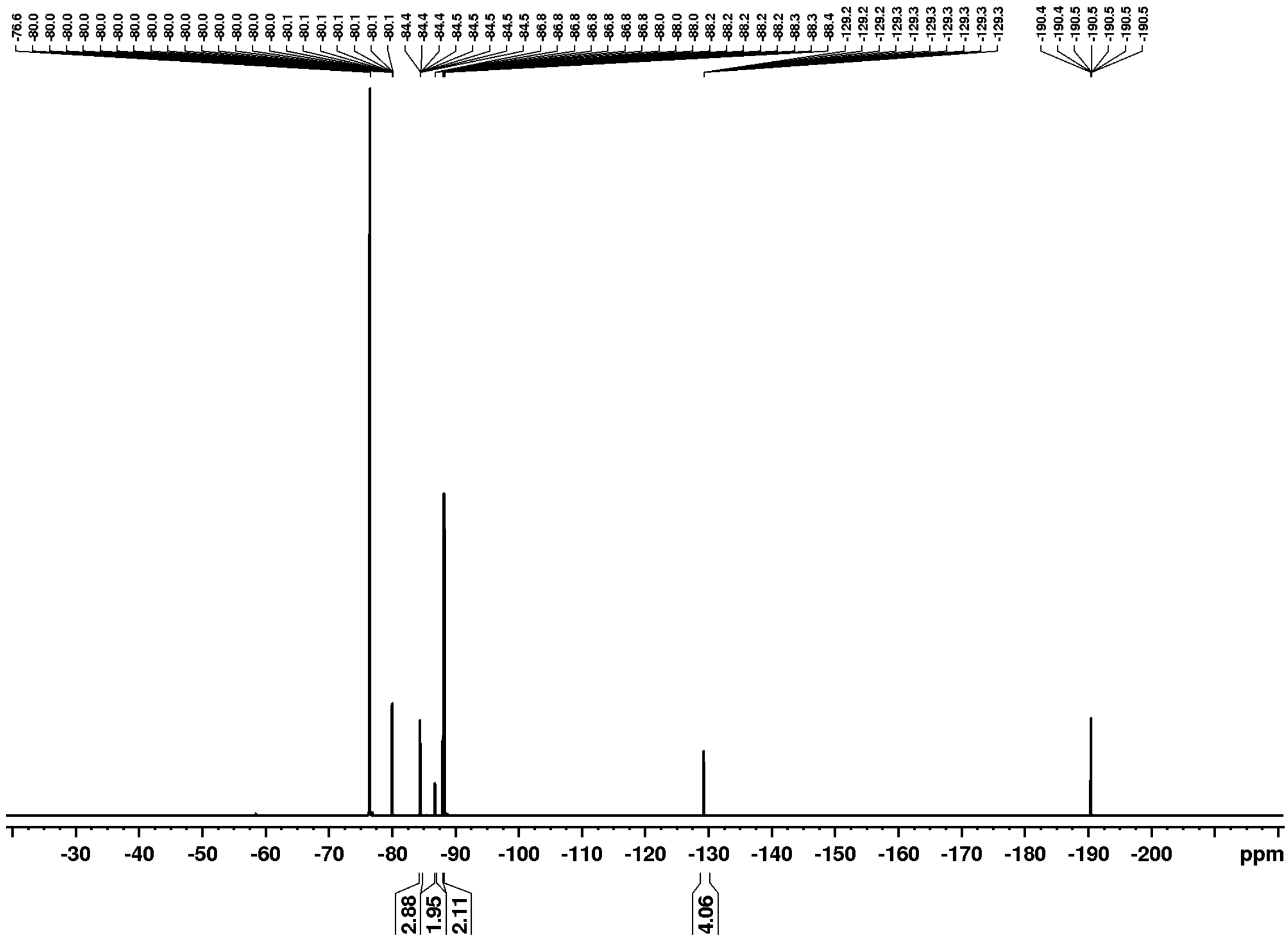

Figure S173. ^{19}F NMR of $(CF_3)_2CFCF_2OCHF_2$/$CF_3CF_2CF_2CF_2OCHF_2$ measured as neat substance with acetone-d_6 and CCl_3F as external standard, integrals shown for $CF_3CF_2CF_2CF_2OCHF_2$.

***i+n*-$C_4F_9OCF_3$**

Boiling point (distillation): T_{bp} = 34.5 °C at 1000 mbar.

Boiling point (vapor pressure): T_{bp} = 35.1 °C at 1013 mbar (Antoine parameter: A = 4.041; B = 1032.2; C = −52.418 for equation: $p = 10^{(A-B/(T+C))}$).

Enthalpy of vaporization (T = 298.15 K): $\Delta_{vap}H$ = 29.1 kJ mol^{-1} (for equation: $\Delta_{vap}H = \ln(10)\cdot R\cdot T^2\cdot(B/(T+C)^2)/1000$).

$^{13}C\{^1H\}$ NMR (100.7 MHz, $(CH_3)_2CO$): δ = 119.3 (q, 1C, $CF_3CF_2CF_2CF_2O\mathbf{C}F_3$, $^1J(^{19}F, ^{13}C)$ = 269.6 Hz), 119.2 (q, 1C, $(CF_3)_2CFCF_2O\mathbf{C}F_3$, $^1J(^{19}F, ^{13}C)$ = 269.9 Hz), 118.5 (qd, 2C, $(\mathbf{C}F_3)_2CFCF_2OCF_3$, $^1J(^{19}F, ^{13}C)$ = 275.2 Hz, $^2J(^{19}F, ^{13}C)$ = 26.1 Hz), 115.9 (td, 1C, $(CF_3)_2CF\mathbf{C}F_2OCF_3$, $^1J(^{19}F, ^{13}C)$ = 289.4 Hz, $^2J(^{19}F, ^{13}C)$ = 25.6 Hz), 114.8 (m, 1C, $CF_3CF_2CF_2\mathbf{C}F_2OCF_3$), 108.4 (tm, 1C, $CF_3CF_2\mathbf{C}F_2CF_2OCF_3$, $^1J(^{19}F, ^{13}C)$ = 269.0 $^1J(^{19}F, ^{13}C)$ = $CF_3\mathbf{C}F_2CF_2CF_2OCF_3$, $^1J(^{19}F, ^{13}C)$ = 269.4 Hz), 88.4 (dsept, 1C, $(CF_3)_2\mathbf{C}FCF_2OCF_3$, $^1J(^{19}F, ^{13}C)$ = 224.5 Hz, $^2J(^{19}F, ^{13}C)$ = 32.5 Hz) ppm. **$^{13}C\{^{19}F\}$ NMR** (125.8 MHz, $(CH_3)_2CO$): δ = 119.3 (s, 1C, $CF_3CF_2CF_2CF_2O\mathbf{C}F_3$), 119.2 (s, 1C, $(CF_3)_2CFCF_2O\mathbf{C}F_3$), 118.5 (s, 2C, $(\mathbf{C}F_3)_2CFCF_2OCF_3$), 117.3 (s, 1C, $(CF_3)_2CF\mathbf{C}F_2OCF_3$), 115.9 (s, 1C, $\mathbf{C}F_3CF_2CF_2CF_2OCF_3$), 114.8 (s, 1C, $CF_3CF_2CF_2\mathbf{C}F_2OCF_3$), 108.2 (s, 1C, $CF_3CF_2\mathbf{C}F_2CF_2OCF_3$), 88.4 (s, 1C, $(CF_3)_2\mathbf{C}FCF_2OCF_3$) ppm.

^{17}O NMR (81.4 MHz, $CDCl_3$): δ = 129.8 (s, 1O, $(CF_3)_2CFCF_2\mathbf{O}CF_3$, $CF_3CF_2CF_2CF_2\mathbf{O}CF_3$) ppm.

^{19}F NMR (470.5 MHz, $(CH_3)_2CO$): δ = −58.8 (t, 3F, $(CF_3)_2CFCF_2OC\mathbf{F}_3$, $^4J(^{19}F, ^{19}F)$ = 9.1 Hz), −58.9 (t, 3F, $CF_3CF_2CF_2CF_2OC\mathbf{F}_3$, $^4J(^{19}F, ^{19}F)$ = 8.5 Hz), −76.7 (dt, 6F, $(C\mathbf{F}_3)_2CFCF_2OCF_3$, $^3J(^{19}F, ^{19}F)$ = 6.2 $^4J(^{19}F, ^{19}F)$ = 9.6 Hz), −81.7 (m, 2F, $(CF_3)_2CFC\mathbf{F}_2OCF_3$), −84.7 (tm, 3F, $C\mathbf{F}_3CF_2CF_2CF_2OCF_3$, $^3J(^{19}F, ^{19}F)$ = 9.1 Hz), −88.4 (tq, 2F, $CF_3CF_2CF_2C\mathbf{F}_2OCF_3$, $^3J(^{19}F, ^{19}F)$ = 14.0 Hz, $^4J(^{19}F, ^{19}F)$ = 9.1 Hz), −129.4 (m, 2F, $CF_3CF_2C\mathbf{F}_2CF_2OCF_3$), −129.4 (m, 2F, $CF_3C\mathbf{F}_2CF_2CF_2OCF_3$), −190.2 (sept, 1F, $(CF_3)_2C\mathbf{F}CF_2OCF_3$, $^3J(^{19}F, ^{19}F)$ = 6.5 Hz) ppm. GC-MS [t = 0.43 min] m/z$^+$: 219 [M −OCF_3], 197 [M −CF_5], 169 [M −CF_2OCF_3], 150 [M −CF_3OCF_3], 135 [M −C_3F_7], 131 [M −CF_4OCF_3], 119 [M −$C_2F_4OCF_3$], 100 [M −$C_2F_5OCF_3$], 69 [M −C_4F_9O].

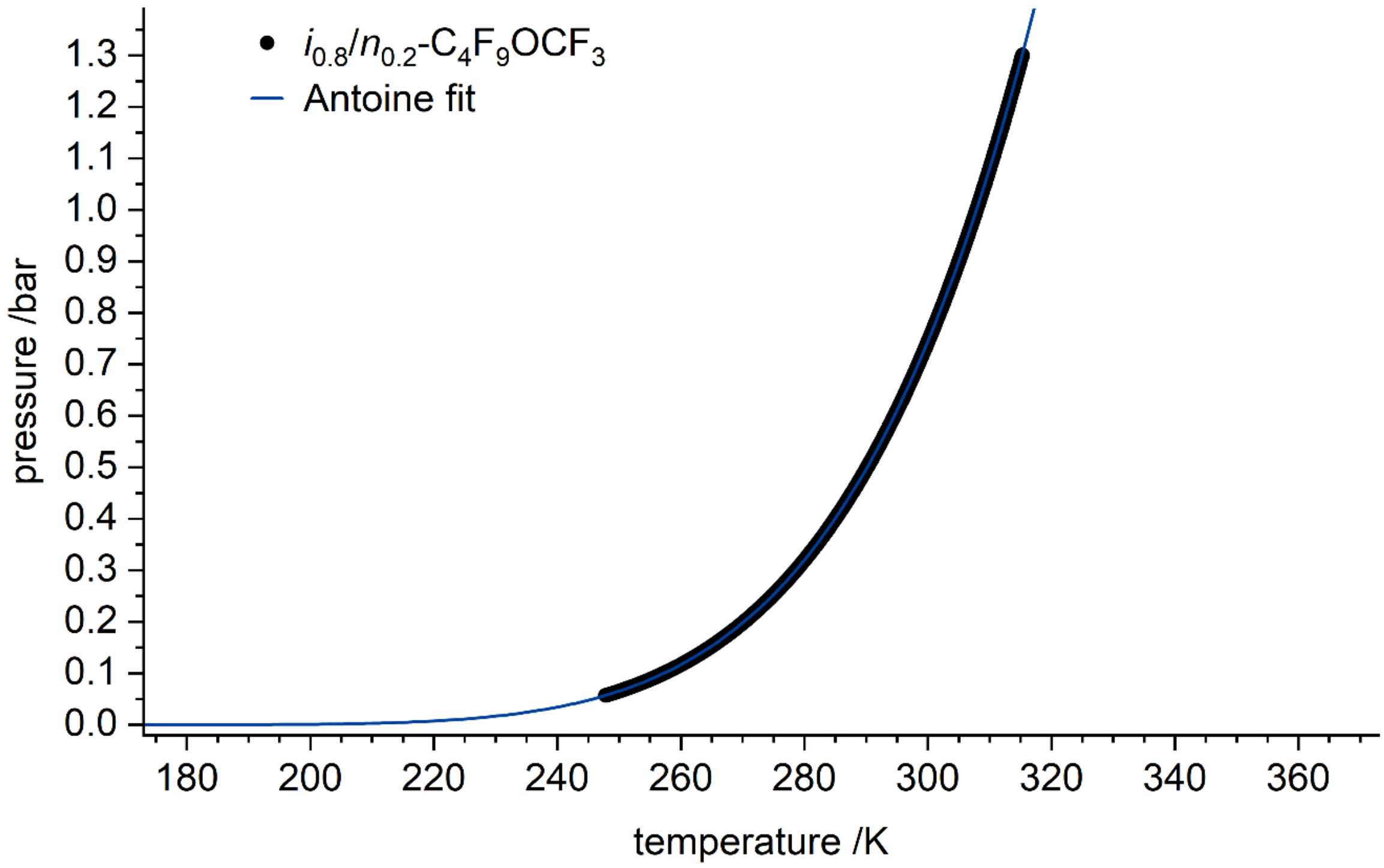

Figure S174. Vapor pressure measurement and Antoine fit of $i_{0.8}/n_{0.2}$-$C_4F_9OCF_3$ (Antoine parameter: A = 4.041; B = 1032.2; C = −52.418 for equation: $p = 10^{(A-B/(T+C))}$).

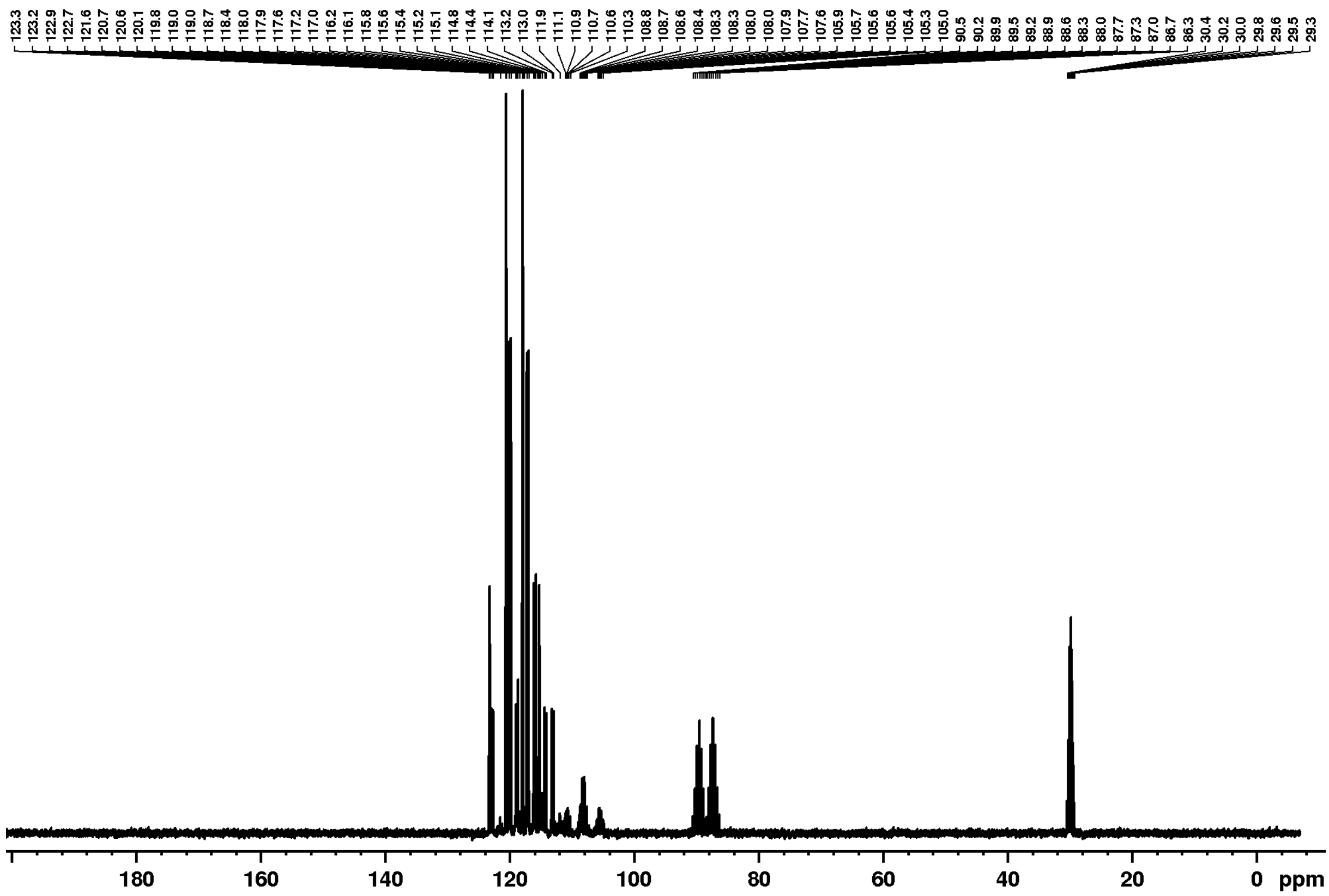

Figure S175. $^{13}C\{^1H\}$ NMR of $(CF_3)_2CFCF_2OCF_3/CF_3CF_2CF_2CF_2OCF_3$ measured as neat substance with acetone-d_6 and CCl_3F as external standard.

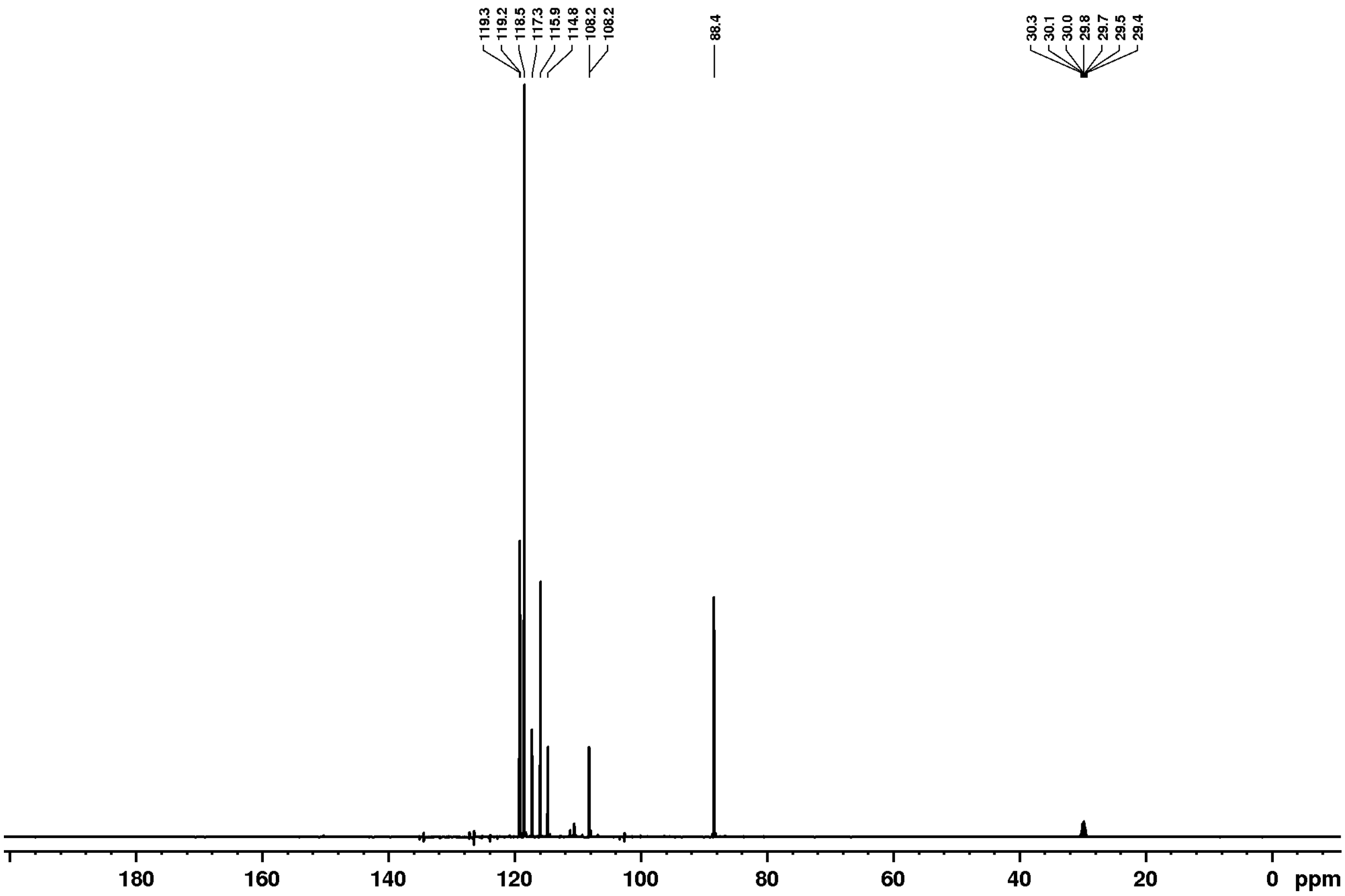

Figure S176. $^{13}C\{^{19}F\}$ NMR of $(CF_3)_2CFCF_2OCF_3/CF_3CF_2CF_2CF_2OCF_3$ measured as neat substance with acetone-d_6 and CCl_3F as external standard.

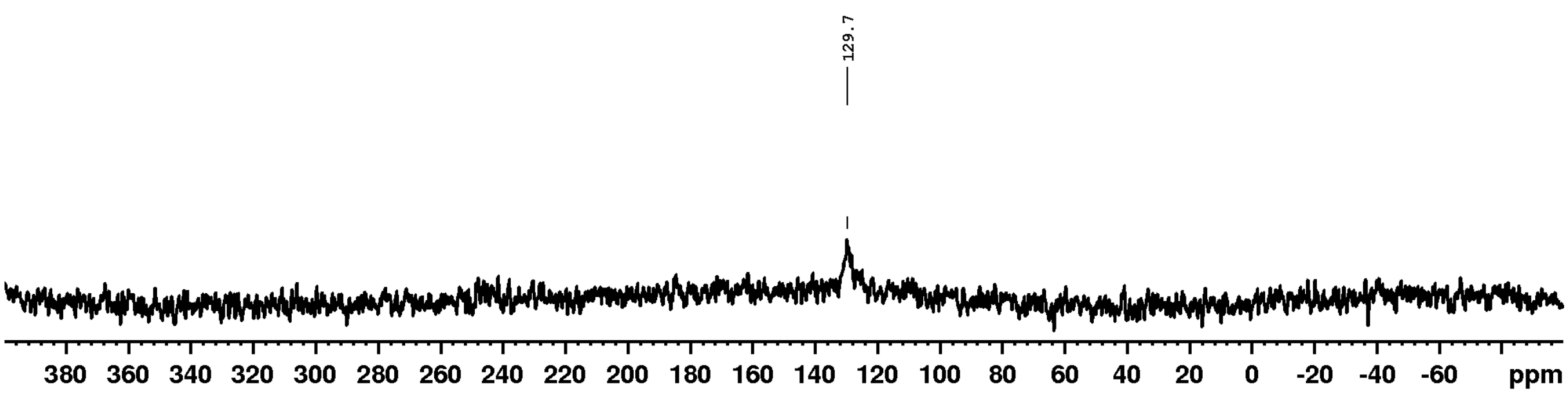

Figure S177. ^{17}O NMR of $(CF_3)_2CFCF_2OCF_3/CF_3CF_2CF_2CF_2OCF_3$ in $CDCl_3$.

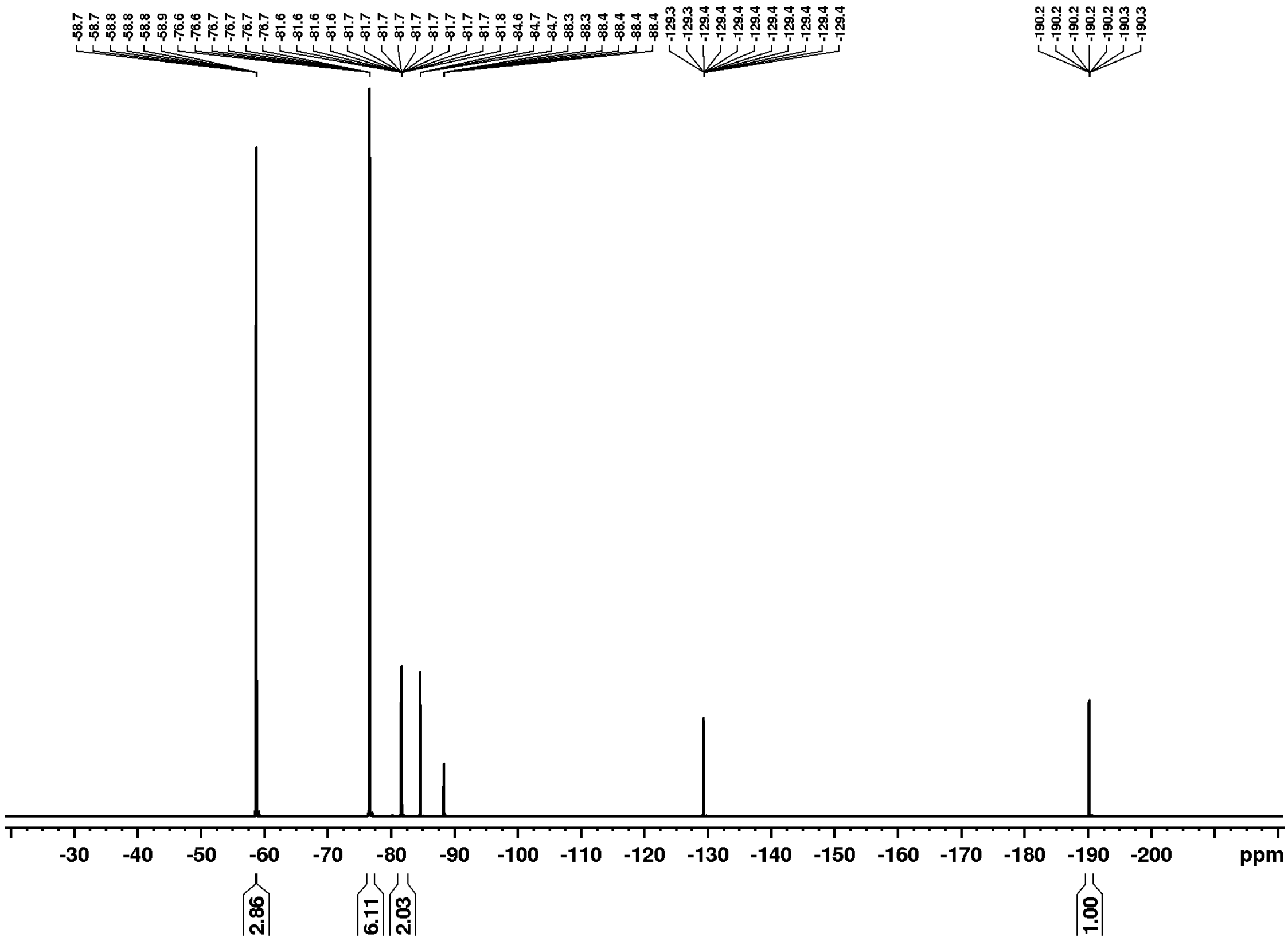

Figure S178. ^{19}F NMR of $(CF_3)_2CFCF_2OCF_3/CF_3CF_2CF_2CF_2OCF_3$ measured as neat substance with acetone-d$_6$ and CCl_3F as external standard, integrals shown for $(CF_3)_2CFCF_2OCF_3$.

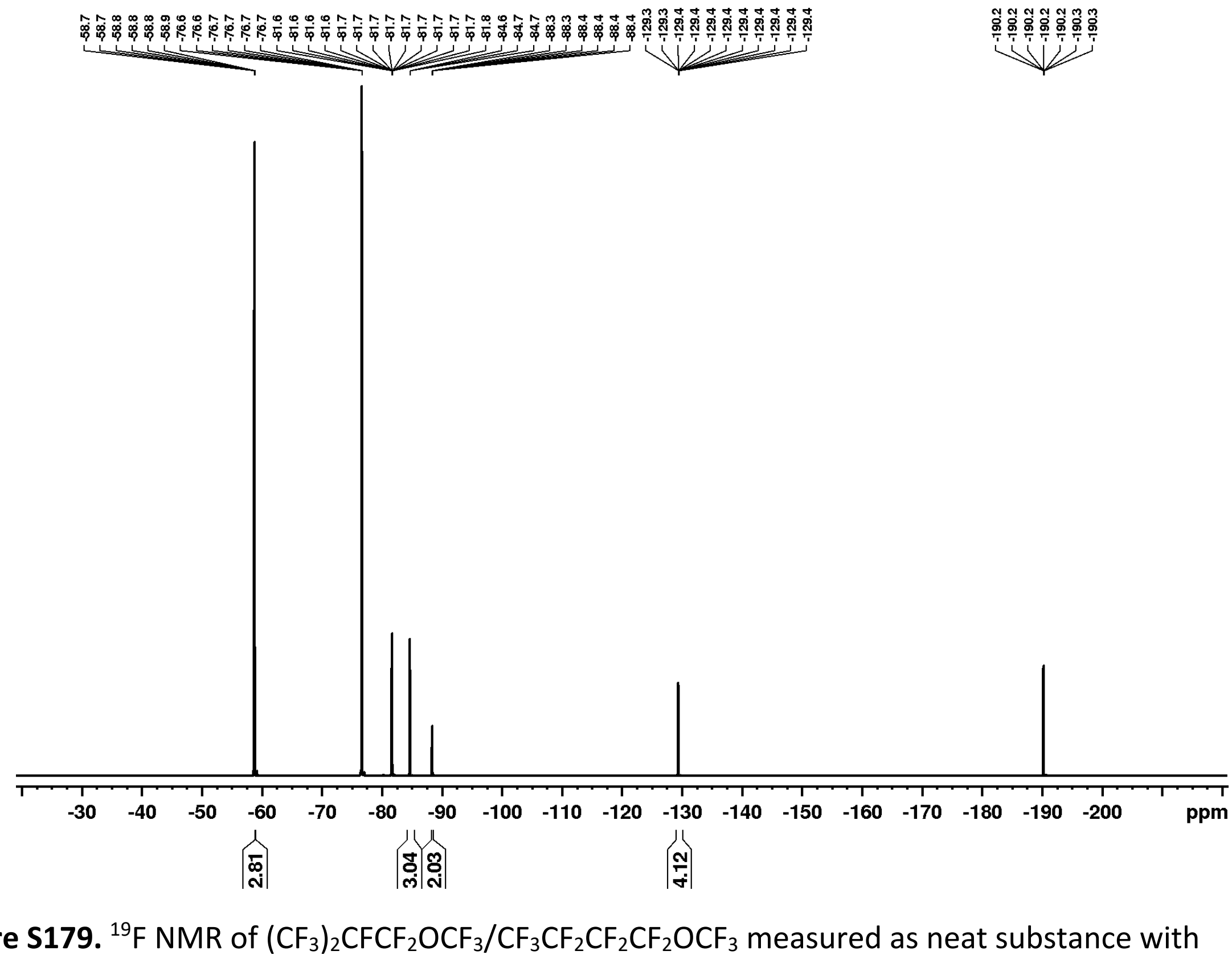

Figure S179. ^{19}F NMR of $(CF_3)_2CFCF_2OCF_3/CF_3CF_2CF_2CF_2OCF_3$ measured as neat substance with acetone-d$_6$ and CCl_3F as external standard, integrals shown for $CF_3CF_2CF_2CF_2OCF_3$.

4. Single crystal X-ray diffraction

Single crystals suitable for X-ray diffraction studies of $(CF_3)_2CHOCH_2F$ (**1**), $(CF_3)_2CHOCHF_2$ (**2**), $(CF_3)_2CHOCF_3$ (**3**), $(CF_3)_2CFOCF_3$ (**4**), $(CF_3)_2CFCF_2OCH_3$ and $(CF_3)_2CHOCH_3$, which are all liquids or gases at room temperature, were grown *in situ* by manual crystallization on the diffractometer. The general procedure of this crystallization process was reported previously [16-18]. Samples of the respective substance were loaded into glass capillaries with 0.28 mm inside diameter. The filled capillaries were cooled on the diffractometer in a nitrogen gas stream from an *Oxford* cryosystem attachment. The following steps until data collection were monitored manually under an optical microscope with polarizer. After repeated freezing and heating of the samples to a polycrystalline state the samples were warmed just below their respective melting points to slow down nucleation. Manual local heating in the next step with a thin copper wire reduced the number of crystalline domains ideally so that only a single seed is left. By slow cooling the crystal slowly grows until only a single specimen fills the entire cooled area. Afterwards it was cooled to the respective temperature of data collection.

Crystal data were collected on a *Rigaku* XtaLAB Synergy-S, dualflex diffractometer with a HyPix 6000 detector, using $Cu_{K\alpha}$ radiation (micro-focus sealed X-ray tube, λ_{Cu} = 1.54184 Å). In the case of $(CF_3)_2CHOCH_3$ $Mo_{K\alpha}$ (micro-focus sealed X-ray tube, λ_{Mo} =0.71073 Å) was used instead of $Cu_{K\alpha}$. The structures were solved by intrinsic phasing methods (SHELXT) and refinement is based on full-matrix least-squares calculations on F^2 (SHELXL) [19]. All nonhydrogen atoms were refined anisotropically. All hydrogens were freely isotropically refined. In the case of $(CF_3)_2CHOCH_3$ the hydrogen atoms were freely and anisotropically refined. The refinement was done using NoSpherA2, which makes use of tailor-made aspherical atomic form factors calculated on-the-fly from a Hirshfeld-partitioned electron density (ED) - not from spherical-atom form factors [20].

Calculations were carried out using the ShelXle graphical interface [21]. Molecular structure diagrams were drawn with the program Diamond [22]. Crystallographic data have been deposited with the Cambridge Crystallographic Data Centre. Experimental details, crystal data, and CCDC numbers are summarized in Table S3. These data can be obtained free of charge from The Cambridge Crystallographic Data Centre via www.ccdc.cam.ac.uk/data_request/cif.

Table S3. Crystal data and structure refinement details of $(CF_3)_2CHOCH_2F$ (**1**), $(CF_3)_2CHOCHF_2$ (**2**), $(CF_3)_2CHOCF_3$ (**3**), $(CF_3)_2CFOCF_3$ (**4**), $(CF_3)_2CFCF_2OCH_3$, and $(CF_3)_2CHOCH_3$.

**compound**	**$(CF_3)_2CHOCH_2F$ (1)**	**$(CF_3)_2CHOCHF_2$ (2)**	**$(CF_3)_2CHOCF_3$ (3)**	**$(CF_3)_2CFOCF_3$ (4)**	**$(CF_3)_2CFCF_2OCF_3$**	**$(CF_3)_2CHOCH_3$**
empirical formula	$C_4H_3F_7O$	$C_4H_2F_8O$	C_4HF_9O	$C_4F_{10}O$	$C_5H_3F_9O$	$C_4H_4F_6O$
M_W [g mol^{-1}]	200.06	218.06	236.05	254.04	250.07	182.07
colour	colourless	colourless	colourless	colourless	colourless	colourless
T [K]	100(2)	152(2)	100(2)	100(2)	100(2)	100(2)
crystal system	monoclinic	monoclinic	orthorhombic	orthorhombic	monoclinic	orthorhombic
space group	$P2_1$	$P2_1/n$	$Pnma$	$Pnma$	$P2_1/n$	$P2_12_12_1$
a [Å]	8.97740(10)	6.92480(10)	8.88460(10)	13.9488(2)	8.67390(10)	6.8132(2)
b [Å]	11.4460(2)	12.0885(2)	10.68260(10)	10.1645(2)	10.28710(10)	8.4453(2)
c [Å]	9.54790(10)	8.92190(10)	7.38210(10)	5.10000(10)	9.30510(10)	11.4059(3)
α [°]	90	90	90	90	90	90
β [°]	91.4100(10)	112.562(2)	90	90	106.1170(10)	90
γ [°]	90	90	90	90	90	90
V [Å^3]	980.80(2)	689.695(19)	700.640(14)	723.09(2)	797.654(15)	656.29(3)
Z	6	4	4	4	4	4
ρ_{calcd} [Mg m^{-3}]	2.032	2.100	2.238	2.334	2.082	1.843
μ [mm^{-1}]	2.540	2.696	2.938	3.122	2.626	0.238
F(000) [e]	588	424	456	488	488	360
θ range [°]	4.633−76.798	6.502−76.972	7.293−77.251	6.346−77.292	6.174−76.628°	3.001−31.085°
Radiation, λ [Å]	$Cu_{K\alpha}$, 1.54184	$Cu_{K\alpha}$, 1.54184	$Cu_{K\alpha}$, 1.54184	$Cu_{K\alpha}$, 1.54184	$Cu_{K\alpha}$, 1.54184	$Mo_{K\alpha}$, 0.71073
reflections collected	25247	17509	19191	5849	20466	21382
independent reflections [$I > 2\sigma(I)$]	3755	1301	775	726	1556	1756
R(int)	0.0990	0.1085	0.0993	0.0969	0.1823	0.0295
data / restraints / parameters	3755 / 1 / 340	1441 / 0 / 124	783 / 0 / 72	792 / 0 / 76	1651 / 0 / 137	1918 / 0 / 112
$R1$ [$I > 2\sigma(I)$]	0.0490	0.0911	0.0633	0.0700	0.0578	0.0272
$wR2$ (all data)	0.1302	0.2428	0.1560	0.1824	0.1679	0.0627
GOF on F^2	1.056	1.129	1.224	1.106	1.123	1.052
largest diff. peak / hole [e Å^{-3}]	0.423 / –0.585	0.793 / –1.028	0.513 / –0.876	0.531 / –0.870	0.428 / –0.567	0.268 / –0.195
CCDC number	2565960	2565961	2565963	2565964	2565962	2565965

5. References

[1] R. K. Harris, E. D. Becker, S. M. Cabral de Menezes, R. Goodfellow, P. Granger, "NMR Nomenclature. Nuclear Spin Properties and Conventions for Chemical Shifts", *Pure and Applied Chemistry* 73 (2001), 1795–1818, https://doi.org/10.1351/pac200173111795.

[2] G. R. Fulmer, A. J. M. Miller, N. H. Sherden, H. E. Gottlieb, A. Nudelman, B. M. Stoltz, J. E. Bercaw, K. I. Goldberg, "NMR Chemical Shifts of Trace Impurities: Common Laboratory Solvents, Organics, and Gases in Deuterated Solvents Relevant to the Organometallic Chemist", *Organometallics* 29 (2010), 2176–2179, https://doi.org/10.1021/om100106e.

[3] W. Kohn, L. J. Sham, "Self-consistent equations including exchange and correlation effects", *Physical Review A* 140 (1965), 1133–1138, https://doi.org/10.1103/PhysRev.140.A1133.

[4] F. Neese, "Software Update: The ORCA Program System—Version 6.0", *WIREs Computational Molecular Science* 15 (2025), e70019, https://doi.org/10.1002/wcms.70019.

[5] F. Neese, "The SHARK integral generation and digestion system", *Journal of Computational Chemistry* 44 (2023), 381–396, https://doi.org/10.1002/jcc.26942.

[6] E. Caldeweyher, C. Bannwarth, S. Grimme, "Extension of the D3 dispersion coefficient model", *The Journal of Chemical Physics* 147 (2017), 034112, https://doi.org/10.1063/1.4993215.

[7] E. Caldeweyher, S. Ehlert, A. Hansen, H. Neugebauer, S. Spicher, C. Bannwarth, S. Grimme, "A generally applicable atomic-charge dependent London dispersion correction", *The Journal of Chemical Physics* 150 (2019), 154122, https://doi.org/10.1063/1.5090222.

[8] J.-D. Chai, M. Head-Gordon, "Long-range corrected hybrid density functionals with damped atom–atom dispersion corrections", *Physical Chemistry Chemical Physics* 10 (2008), 6615–6620, https://doi.org/10.1039/b810189b.

[9] A. Schäfer, C. Huber, R. Ahlrichs, "Fully optimized contracted Gaussian basis sets of triple zeta valence quality for atoms Li to Kr", *Journal of Chemical Physics* 100 (1994), 5829–5835, https://doi.org/10.1063/1.467146.

[10] K. Eichkorn, F. Weigend, O. Treutler, R. Ahlrichs, "Auxiliary basis sets for main row atoms and transition metals and their use to approximate Coulomb potentials", *Theoretical Chemistry Accounts* 97 (1997), 119–124, https://doi.org/10.1007/s002140050244.

[11] K. Eichkorn, O. Treutler, H. Öhm, M. Häser, R. Ahlrichs, "Auxiliary basis sets to approximate Coulomb potentials", *Chemical Physics Letters* 240 (1995), 283–290, https://doi.org/10.1016/0009-2614(95)00838-U.

[12] F. Weigend, R. Ahlrichs, "Balanced basis sets of split valence, triple zeta valence and quadruple zeta valence quality for H to Rn: Design and assessment of accuracy", *Physical Chemistry Chemical Physics* 7 (2005), 3297–3305, https://doi.org/10.1039/B508541a.

[13] M. K. Kesharwani, B. Brauer, J. M. L. Martin, "Frequency and Zero-Point Vibrational Energy Scale Factors for Double-Hybrid Density Functionals (and Other Selected Methods): Can Anharmonic Force Fields Be Avoided?", *Journal of Physical Chemistry A* 119 (2015), 1701–1714, https://doi.org/10.1021/jp508422u.

[14] G. A. Zhurko, "ChemCraft" 1.8 (www.chemcraftprog.com), 2014.

[15] N. Ignat'ev, "Electrochemical Fluorination: A Powerful Tool for the Preparation of Organofluorine Compounds", in "Modern Synthesis Processes and Reactivity of Fluorinated Compounds“ (Eds.: H. Groult, F. Leroux, A. Tressaud), Elsevier Inc., London, UK, (2016), https://doi.org/10.1016/B978-0-12-803740-9.00004-4.

[16] M. Bujak, H.-G. Stammler, S. Blomeyer, N. W. Mitzel, "The nature of interactions of benzene with CF_3I and CF_3CH_2I", *Chemical Communications* 55 (2019), 175–178, https://doi.org/10.1039/c8cc08980a.

[17] M. T. Kirchner, D. Bläser, R. Boese, "Co-crystals with Acetylene: Small Is not Simple!", *Chemistry- A European Journal* 16 (2010), 2131–2146, https://doi.org/10.1002/chem.200901314.

[18] D. A. Bond, "*In situ* co-crystallisation as a tool for low-temperature crystal engineering", *Chemical Communications* (2003), 250–251, https://doi.org/10.1039/B208904A.
[19] G. M. Sheldrick, "Crystal structure refinement with SHELXL", *Acta Crystallographica Section C* 71 (2015), 3–8, https://doi.org/10.1107/S2053229614024218.
[20] F. Kleemiss, O. V. Dolomanov, M. Bodensteiner, N. Peyerimhoff, L. Midgley, L. J. Bourhis, A. Genoni, L. A. Malaspina, D. Jayatilaka, J. L. Spencer, F. White, B. Grundkötter-Stock, S. Steinhauer, D. Lentz, H. Puschmann, S. Grabowsky, "Accurate crystal structures and chemical properties from NoSpherA2", *Chemical Science* 12 (2021), 1675–1692, https://doi.org/10.1039/D0SC05526C.
[21] C. B. Hübschle, G. M. Sheldrick, B. Dittrich, "ShelXle: A Qt Graphical User Interface for SHELXL", *Journal of Applied Crystallography* 44 (2011), 1281–1284, https://doi.org/10.1107/S0021889811043202.
[22] K. Brandenburg, "Diamond 5.1.1", Crystal Impact GbR, 2025.
